# Turkish Guideline for Diagnosis and Treatment of Allergic Rhinitis (ART)

**DOI:** 10.4274/tao.2021.suppl.1

**Published:** 2021-05

**Authors:** Mustafa Cenk Ecevit, Müge Özcan, İlknur Haberal Can, Emel Çadallı Tatar, Serdar Özer, Erkan Esen, Doğan Atan, Sercan Göde, Çağdaş Elsürer, Aylin Eryılmaz, Berna Uslu Coşkun, Zahide Mine Yazıcı, Mehmet Emre Dinç, Fatih Özdoğan, Kıvanç Günhan, Nagihan Bilal, Arzu Yasemin Korkut, Fikret Kasapoğlu, Bilge Türk, Ela Araz Server, Özlem Önerci Çelebi, Tuğçe Şimşek, Rauf Oğuzhan Kum, Mustafa Kemal Adalı, Erdem Eren, Nesibe Gül Yüksel Aslıer, Tuba Bayındır, Aslı Çakır Çetin, Ayşe Enise Göker, Işıl Adadan Güvenç, Sabri Köseoğlu, Gül Soylu Özler, Ethem Şahin, Aslı Şahin Yılmaz, Ceren Güne, Gökçe Aksoy Yıldırım, Bülent Öca, Mehmet Durmuşoğlu, Yunus Kantekin, Süay Özmen, Gözde Orhan Kubat, Serap Köybaşı Şanal, Emine Elif Altuntaş, Adin Selçuk, Haşmet Yazıcı, Deniz Baklacı, Atılay Yaylacı, Deniz Hancı, Sedat Doğan, Vural Fidan, Kemal Uygur, Nesil Keleş, Cemal Cingi, Bülent Topuz, Salih Çanakçıoğlu, Metin Önerci

**Affiliations:** 1 Department of Otorhinolaryngology, Dokuz Eylül University Faculty of Medicine, İzmir; 2 Department of Otorhinolaryngology, University of Health Sciences Turkey Faculty of Medicine, Ankara; 3 Department of Otorhinolaryngology, Yozgat Bozok University Faculty of Medicine, Yozgat; 4 Department of Otorhinolaryngology, Hacettepe University Faculty of Medicine, Ankara; 5 Department of Otorhinolaryngology, Derince Training and Research Hospital, İzmit; 6 Department of Otorhinolaryngology, Lokman Hekim Hospital, Ankara; 7 Department of Otorhinolaryngology, Ege University Faculty of Medicine, İzmir; 8 Department of Otorhinolaryngology, Selçuk University Faculty of Medicine, Konya; 9 Department of Otorhinolaryngology, Adnan Menderes University Faculty of Medicine, Aydın; 10 Department of Otorhinolaryngology, University of Health Sciences Turkey, Şişli Hamidiye Etfal Training and Research Hospital, İstanbul; 11 Department of Otorhinolaryngology, University of Health Sciences Turkey, Faculty of Medicine, İstanbul; 12 Department of Otorhinolaryngology, University of Health Sciences Turkey, Prof. Dr. Cemil Taşçıoğlu City Hospital, İstanbul; 13 Department of Otorhinolaryngology, Celal Bayar University, Manisa; 14 Department of Otorhinolaryngology, Kahramanmaraş Sütçü İmam University Faculty of Medicine, Kahramanmaraş; 15 Department of Otorhinolaryngology, Uludağ University Faculty of Medicine, Bursa; 16 Department of Otorhinolaryngology, University of Health Sciences Turkey, İstanbul Training and Research Hospital, İstanbul; 17 Department of Otorhinolaryngology, Amasya University Sabuncuoğlu Şerefeddin Training and Research Hospital, Amasya; 18 Department of Otorhinolaryngology, Trakya University Faculty of Medicine, Edirne; 19 Department of Otorhinolaryngology, Atatürk Training and Research Hospital, İzmir; 20 Department of Otorhinolaryngology, University of Health Sciences Turkey, Bursa Yüksek İhtisas Training and Research Hospital, Bursa; 21 Department of Otorhinolaryngology, İnönü University Faculty of Medicine, Malatya; 22 Department of Otorhinolaryngology, Bakırçay University Faculty of Medicine, Çiğli Training and Research Hospital, İzmir; 23 Department of Otorhinolaryngology, Sıtkı Koçman University Faculty of Medicine, Muğla; 24 Department of Otorhinolaryngology, Mustafa Kemal University Faculty of Medicine, Hatay; 25 Bayındır Heathcare Group İçerenköy Hospital, İstanbul; 26 Department of Otorhinolaryngology, University of Health Sciences Turkey, Ümraniye Training and Research Hospital, İstanbul; 27 University of Health Sciences Turkey, Bozyaka Training and Research Hospital, Department of Otorhinolaryngology, İzmir; 28 Department of Otorhinolaryngology University of Health Sciences Turkey, Kayseri City Hospital, Kayseri; 29 Department of Otorhinolaryngology, Alanya Alaaddin Keykubat University Faculty of Medicine, Antalya; 30 Department of Otorhinolaryngology, Abant İzzet Baysal University Faculty of Medicine, Bolu; 31 Department of Otorhinolaryngology, Sivas Cumhuriyet University Faculty of Medicine, Sivas; 32 Department of Otorhinolaryngology, Bahçeşehir University Faculty of Medicine, İstanbul; 33 Department of Otorhinolaryngology, Balıkesir University Faculty of Medicine, Balıkesir; 34 Department of Otorhinolaryngology, Bülent Ecevit University Faculty of Medicine, Zonguldak; 35 Department of Otorhinolaryngology, Kocaeli University Faculty of Medicine, Kocaeli; 36 Department of Otorhinolaryngology, Adıyaman University Faculty of Medicine, Adıyaman; 37 Department of Otorhinolaryngology, University of Health Sciences Turkey, Eskişehir City Hospital, Eskişehir; 38 Department of Otorhinolaryngology, Gazi University Faculty of Medicine, Ankara; 39 Department of Otorhinolaryngology, İstanbul University İstanbul Faculty of Medicine, İstanbul; 40 Department of Otorhinolaryngology, Eskişehir Osmangazi University Faculty of Medicine, Eskişehir; 41 Department of Otorhinolaryngology, Pamukkale University Faculty of Medicine, Denizli

**Keywords:** Allergic rhinitis, guideline, rhinitis

## Abstract

**Object:**

To prepare a national guideline for Otorhinolaryngologist who treat allergic rhinitis patients.

**Methods:**

The study was conducted by three authors, namely the writing support team. The support team made the study plan, determined the writing instructions, chose the subgroups including the advisory committee, the advisors for authors and the authors. A workshop was organized at the very beginning to explain the details of the study to the team. Advisors took the chance to meet their coworkers in their subgroups and determined the main headings and subheadings of the guideline, together with the authors. After key words were determined by the authors, literature search was done in various databases. The authors keep in touch with the advisors and the advisors with the advisory committee and the support group at every stage of the study. National and International published articles as well as the abstracts of unpublished studies, imperatively presented in National Congresses, were included in this guideline. Only Guideline and meta-analyses published in last seven years (2013-2017) and randomized controlled studies published in last two years (2015-2017) were included. After all work was completed by the subgroups, support team brought all work together and edited the article.

**Results:**

A detailed guideline about all aspects of allergic rhinitis was created.

**Conclusion:**

The authors believe that this guideline will enable a compact and up-to-date information on allergic rhinitis to healthcare professionals. This guideline is the first in the field of Otolaryngology in Turkey. It should be updated at regular intervals.

## 1. Why have we composed this guide?

Allergic rhinitis (AR) is a frequently seen global upper airway disorder affecting individuals at all ages. The upper airway is in continuum with a number of important regions, and disorders of upper airway cause significant comorbidities. The most frequent comorbidity of AR is asthma. Acute or chronic rhinosinusitis, otitis media with effusion, adenoid hypertrophy and gastroesophageal reflux may accompany AR. AR affects quality of life negatively since it is a frequent disease affecting individuals at all age groups, and may lead to complications.

Although late diagnosis of AR or errors in its treatment do not lead to fatal outcomes in the early phase, they may result in significant morbidity. Errors in diagnosis and treatment result in an economic burden and psychological dysfunction in the affected patients. Therefore, its epidemiology, and the basic principles for avoidance, diagnosis, treatment and alternative treatment must be known.

Physicians in various disciplines come across with AR patients due to high incidence and prevalence of disease in all age groups, and its relation and effect on multiple body systems. Not only allergists and pediatricians, but also otorhinolaryngologists frequently encounter with those patients. In Turkey, there are no Guideline prepared for all medical specialties. This guideline has been prepared to increase awareness of every physician at all disciplines and grades. It intends to give clear and practical messages on epidemiology, clinical picture, complications, and treatment of AR by transferring the experiences of the otorhinolaryngologists in Turkey.

## 2. Definition and pathogenesis of allergic rhinitis

AR was first described by Hansel in 1929, based on its clinical symptoms, namely sneezing, nasal obstruction, and rhinorrhea. Allergic Rhinitis and its Impact on Asthma (ARIA) working group was founded by World Health Organization in 1999. This group has prepared detailed Guideline for clinicians on definition, classification, treatment algorithms using data in the literature, and updated them regularly (1). The ARIA Working Group has defined rhinitis as a nasal mucosal inflammation characterized by nasal symptoms including rhinorrhea, sneezing, nasal obstruction and/or nasal itching. AR has been defined as a clinical form accompanied by immunoglobulin E (IgE)-related immune response.

AR is characterized by a chronic mucosal inflammation induced by an IgE-related type 1 hypersensitivity reaction based on the inflammatory mediators released after the process of the antigen presentation, T cell differentiation, IgE synthesis and mast cell degranulation. It is a hyper-responsive state in which eosinophils and lymphocytes play the principal role due to repetitive stimuli of antigens (2, 3).

### 2.1. IgE sensitization

The allergens contacting mucosa and skin are presented to T cells by antigen presenting cells (APC), they are processed by epitope peptides, and presented to T-helper (Th) lymphocytes together with major histocompatibility (MHC) class II molecules. Activated CD4+ Th2 lymphocytes release cytokines, mainly interleukin (IL)-4 and IL-13, and they communicate with B cells which synthesize allergen-specific IgE (IgE sensitization). IgE releasing memory and plasma cells also develop. Then, the allergen specific IgE binds to the high-affinity IgE receptors on the surface of the mast cells (3).

### 2.2. Early phase response

This phase starts minutes after allergen exposure in sensitized individuals, and lasts for 2-4 hours. Mast cell degranulation is the main component of the early phase response. A vast number of mast cells are present in the epithelial part of the nasal mucosa, and they are easily activated after re-exposure to antigen. IgEs binded to the high-affinity receptors cross-bind to release pre-synthesized and newly synthesized mediators from the mast cells (2). Pre-synthesized mediators are released to extracellular fluid within seconds / minutes. Those mediators include histamine, prostoglandins, leukotriens, proteases, proteoglycans, cytokines and chemokines, which are responsible for edema, increased vascular permeability and rhinorrhea in AR. Histamine is the main mediator. It stimulates the sensory nerve endings of the trigeminal nerve, and causes sneezing, itching, and increased mucosal secretions. It results in nasal congestion acting on vessels together with leukotriens and prostoglandins.

### 2.3. Late phase response

This response appears 4-6 hours after the allergen exposure, and follows the early phase response. It lasts approximately 18-24 hours. Nasal submucosal T lymphocytes, basophils and eosinophils play role in the late phase. They release leukotrien, kinin, histamine, chemokine and cytokines. IL-4, IL- 5, IL-9 and IL-13 that released from mast cells, early lymphocytes, basophils and Th2 cells initiate and maintain the late phase response. IL-4 and IL-13 increase the expression of vascular cell adhesion molecule (VCAM1), and cause eosinophil, Th2 lymphocyte and basophil infiltration into nasal mucosa. RANTES (Regulated on Activation Normal T Cell Expressed and Secreted), eotaxin, monocyte chemoattractant protein (MCP)-4 and Thymus and activation regulated chemokine (TARC) are released, which provide a strong chemotaxis for eosinophil, basophil and T lymphocytes. Granulocyte-macrophage colony-stimulating factor (GM-CSF) increases the survival of eosinophils that have invaded the nasal mucosa. Eosinophilic cationic protein (ECP), thrombocyte activating factor and major basic protein released by eosinophils also play role in the late phase. Late phase response is particularly related to nasal congestion. Both upper and lower airways are affected by the local inflammation of AR, and systemic inflammation appears (4).

Eicosanoid, endopeptidase, cytokine and chemokines released from the nasal mucosa [IL-6, IL-8, IL-25, IL-31, IL- 33, TSLP, GM-CSF, tumor necrosis factor (TNF)-a, RANTES, TARC, eotaxin, stem cell factor (SCF)] result in the allergic inflammation. Matrix metalloproteinase (MMP)-2, MMP-9 and MMP-13 are released from the nasal epithelial cells, and they degrade the extracellular matrix. Human Leukocyte Antigen – DR isotype (HLA-DR) and CD86 expressed by nasal epithelial cells present antigen to T cells. IL-25, IL-33 and Epithelial cell-thymic stromal lymphoprotein (TSLP) are important inducers of AR. IL-4 is produced by natural killer (NK) 1+ T and mast cells, and induces Th2 differentiation. IL-12 is produced by macrophages and NK cells, and causes Th1 differentiation. An increase in IL-25 accentuates Th2-related inflammation. IL-33 enhances Th2 response, and activates type 2 innate lymphoid cells (ILC) that release IL-5, IL-9 and IL-13. These three cytokines contribute augmented Th2 response and tissue eosinophilia by increasing ILC. The allergens tend to destruct the epithelial barrier in AR. Proteolytic enzymatic activity of various allergens directly activates the epithelial cells, cause cytokine-chemokine release, and result in airway inflammation, independent of IgE.

Endothelial cell-derived VCAM-1 increases in the pollen season. RANTES and eotaxin are other important cytokine and chemokine released by the endothelial cells. H1 receptor is also expressed by the endothelial cells. Macrophage and dendritic cells (DC), too, release chemokines and influence Th2 cells as well as tissue fibroblasts. IL-4 induces allergic fibroblast proliferation, and GM-CSF production increases through histamine stimulation (3).

Allergen tolerance may occur by induction of T regulatory (Treg) cells that balance the hyper-activation of the immune system (5). All processes related to T cell subgroups determine the main targets of treatment in allergic diseases. There are two main Treg subgroups. The first one is the innate thymic FOXP3+, CD4+, CD25+Treg cells, and the other one is the inducible Treg cells that may be formed at the periphery under tolerogenic conditions (6).

FOXP3+Treg and IL-10 positive Tr1 cells, which are two subunits of inducible Treg cells, play role in development of allergen tolerance (7). The mutation of FOXP3, the main transcription factor in the development of Treg cells, may lead to allergic and autoimmune disorders. Treg cells influence Th2 cells as well as DCs, mast cells, basophils and eosinophils. Treg cells contribute the negative regulation of allergen specific IgE, increase production of blocking antibodies (IgG4 and IgA), and may inhibit mast cell degranulation directly by OX40-OX40 ligand interaction.

Together with other factors, it is evident that a decrease in Treg cells plays an important role in development of AR. CD4+CD25+Treg cell numbers decrease in vitro in patients with seasonal AR. In patients with persistent AR, the number and the functions of CD4+CD25+Treg cells are normal, however the number of IL-10 releasing Treg cells decrease (8, 9).

### 2.4. The effect of innate immune response on allergic rhinitis

The most important function of innate immune system in the upper airway is detection of the microorganisms. It is the host defense mechanism coded by the host genes. They include epithelium, mucus layer, cilia, soluble proteins, complement, defensin and a number of cytokines and chemokines. The Dcs, macrophages and mast cells in the upper airway contribute the process. There are two types of DCs: myeloid (mDC) and plasmocytoid (pDC). mDCs, are rich in microbial pattern recognizing receptors, which make a subepithelial network. pDCs express toll-like receptor (TLR)-7 and TLR-9, and release interferon alpha; they play a particular role in anti-viral response. Mast cells express complement receptors for TLR1, TLR2, TLR4,TLR6, C3a and C5a. Neutrophils and NK cells are crucial components of this system. First-line defense provided by innate immune system plays an important role in future development of tolerance or chronic inflammation.

Antimicrobial peptides (AMP) kill microbes straight off. Cathelicidin is one of them, and it triggers tissue inflammation. Defensin is an antimicrobial against bacteria, viruses and fungi (10).

### 2.5. Mast cells

Mast cells play a crucial role in the first phase response of AR. They are the main producers of histamine, leukotriens and prostoglandins. They also release cytokines and chemokines that regulate the late phase response. IgE-activated mast cells express vast amounts of high-affinity IgE receptors (FceRI), CD40L, IL-4 and IL-13. They stimulate local IgE synthesis in nasal mucosal B cells. Mast cells auto-activate themselves by IgE or IL-4 mediated FceRI upregulation. In this way, they intensify the ongoing inflammation (2).

Th2 cells play a role in development and progress of cytokine-dependent inflammation. Basophils are present in the nasal lavage fluids of AR patients, and they are thought to be the main sources of histamine in the late phase reaction. Basophils are also important sources of LTC4 (11).

### 2.6. Basophils

They infiltrate the nasal mucosa in AR (12).

### 2.7. Group 2 innate lymphoid cells

Group 2 innate lymphoid cells (ILC2) release Th2 cytokines. They have been shown to be increased in the peripheral blood in cat antigen-related AR. Another study showed increased ILC2 in peripheral blood of the patients with pollen allergy, and their numbers decreased after subcutaneous immunotherapy (13).

### 2.8. Natural killer cells

AR patients produce type 2 cytokines, and they have a high NK cytotoxic capacity (14). Those cells are giant granular lymphocytes. They produce cytokines such as Interferon- gamma, TNF-alpha and GM-CSF. They do not need MHC receptors to identify their target cells.

### 2.9. Eosinophils

They play a crucial role in the nasal mucosa. The number of eosinophils and the amount of ECP increase in parallel with the severity of the symptoms (15).

### 2.10. Antigen presenting cells

The type and the amount of the allergens that come across with APC are important in an immunological reaction. The most significant APCs are the DCs (16). There are three types of DCs in the nasal mucosa: CD11c+ mDCs, CD123+ pDCs and Langerhans cells (CD1a+, CD207+). They trigger inflammation. DCs break antigen into small pieces, and present them to T cells in cooperation with MHC I and MHCII. They regulate Th2-type allergic reaction over Th1, Th17 and T regulatory reactions. The antigens presented by pDC usually induce tolerance, however mature DCs induce inflammation. DCs play role in allergic inflammation and appearance of symptoms (17, 18).

### 2.11. T and B lymphocytes

CD4 Th cells are formed by activation of DCs. These cells activate effector cells including eosinophils and neutrophils, and cause differentiation of B cells into plasma cells, releasing pathogen-specific immunoglobulin. Another specific T cell group, Tregs, inhibit the immune response. IL-10 and TGF- beta expressed by Treg cells inhibit activation of other T and B cells, DCs and mast cells (19, 20). Other T cells inhibit T cell-related activation in presence of Foxp3- CD25 positive Treg cells that do not express IL-10 or TGF-beta. These Treg cells have been reported as a component of symptom suppression mechanism of immunotherapy. Epigenetic research has been going on concerning specific genomic mutations, expression profiles, and epigenetic alterations of the T and B cells in allergic patients. The network of regulatory cells that control the activation of these cells is also a research topic.

### 2.12. Cytokines and chemokines

Cytokines are soluble proteins or peptides that play role as the mediator hormones of the immune system. Their functions may change in relation with the target cell. Chemokines are a subgroup of the cytokines, and they cause migration of leukocytes into the site of inflammation in AR. IL-1 and IL-2 cause B cell activation. IL-33, IL-25 and TSLP are released by nasal mucosal epithelial cells, and mediate uptake of the allergen by antigen presenting DCs. T-cell informing cytokines interact with undifferentiated T helper (CD4+) cells to induce different immune responses. IL-12 and interferon-gamma induce formation of type 1 Th1 cells which fight against bacteria and viruses. IL-4 pioneers Th2 cells that fight against the parasites. Th17 battles with bacterial and fungal infections, and plays role in autoimmune diseases. Treg cells induce release of IL-10 and transforming growth factor (TGF)-b , inhibit migration of the inflammatory cells, and suppress inflammation by reducing Th function (21). Th-effector cytokines mediate activation of the Th cells. Th2 cells modify B cells to express allergen specific IgE, IL-4, IL-13, IL-5 that induce production of eosinophilic granulocyte, and IL-9 and IL-13 that induce nasal mucosal inflammation (2, 22).

Chemokines induce cell chemotaxis. They define the type of migratory inflammatory leukocyte (eosinophil, neutrophil, basophil, T or B cell). Some chemokines induce high concentration of mediator release from leukocytes, and play role in allergic inflammation. The most crucial chemokines in allergic inflammation are eotaxin-1 (CCL11), eotaxin -2 (CCL24) and eotaxin-3 (CCL26). All of them exert their action through CCR3 receptors located on eosinophils, basophils and Th cells. Another crucial Th2 chemokin is RANTES (CCL5) acting through CCR5 receptor.

### 2.13. The role of local and systemic IgE

In a small group of patients, serum specific IgE and skin prick tests are negative, however these patients have typical AR symptoms. Local IgE synthesis in the nasal mucosa has been presumed after identification of IL-4 and epsilon gene transcription in nasal mucosal B cells with in situ hybridization. Local IgE production may explain why some patients develop asthma and eczema and some others develop AR.

Absence of AR symptoms in presence of positive serum specific IgE and skin prick test may be due to lack of local IgE. It has been noted that some of the patients diagnosed with non-allergic or idiopathic rhinitis might in fact have local IgE-dependent rhinitis (23). A nasal provocation test must be performed in those patients. In a Spanish study, triptase, ECP and Th2 cytokines have been isolated in the nasal lavage fluids of these patients following nasal provocation. The local IgE levels were low, however it was supposed that this might be due to dilution in the nasal lavage fluid (24).

### 2.14. Lipid mediators in allergic rhinitis

Arachidonic acid is released from cell membrane phospholipids in cells activated by phospholipase A2. Arachidonic acid is metabolized through 5-lipoxygenase (5-LO) pathway into leukotriene (LT) B4 and cysteinyl leukotrienes (CysLT), namely, LTC4, LTD4 and LTE4. Neutrophils are the main sources of LTB4, on the other hand, mast cells, basophils and eosinophils produce mainly CysLT. CysLT play role in eosinophil migration, stimulation of airway mucus production, and upregulation of inflammatory cytokines. Prostaglandin (PG) E2, PGD2, PGF2alpha, prostacyclin and thromboxane (TXA2) are produced from arachidonic acid through cyclooxygenase (COX) pathway. Mast cells produce mainly PGD2. There are two forms of COX: basal (COX-1) and inducible (COX-2) forms. PGs have inflammatory functions (PGE2, PGD2, PG2alpha, TXA2), however they may act as anti-inflammatory endogenous molecules (PGE2, PGD2). Lipoxin (LX) A4 is produced by leukocytes from arachidonic acid through 15-LO pathway, or LTA4 is produced and metabolized into LXA4 in thrombocytes. Low LTE4 and PGD2 levels have been determined in nasal biopsy of the patients with AR. CysLT, LTB4 and PGD2 increases with nasal allergen provocation. Nasal symptoms improve with CysLT1 receptor antagonist treatment. LTA4 analogs have potential regulatory actions in inflammation of AR (25).

### 2.15. Nasal mucosal epithelial barrier

Upper airway is the first barrier to allergens. The epithelial barrier of the nose and paranasal sinuses is composed of pseudostratified ciliated epithelium. The epithelial barrier contains antimicrobial proteins such as defensin, cathelicidin, lysosome and lactoferrin. S-100 proteins also have antimicrobial activity through innate immunity and Toll-like receptors (18). Tight junctions, constituted by integral membrane proteins, constitute a crucial part of epithelial barrier. Various antigens contacting nasal mucosa are presented to lymphocytes by the epithelial cells. Tight junction cells in the nasal epithelium are influenced by growth factors and cytokines. Epithelial TSLP increases the tight junction proteins in the epithelial barrier, and plays an important role in inflammation (26).

### 2.16. Neuroimmune mechanisms in allergic rhinitis

The nasal epithelium is innervated by unmyelinated type C trigeminal nerve endings. Sympathetic neurons innervate the arteriovenous anastomoses of the venous sinusoids. Histamine stimulates H1 receptors. Nociceptive receptors are depolarized, resulting in itching in patients with AR. Calcitonin gene-related peptide (CGRP) is a potent vasodilator, and it is closely associated with neuromedin B and gastrin releasing peptide (GRP). Tachykinin, neurokinin A and substance P induce glandular exocytosis while glutamate is an excitatory amino acid neurotransmitter. Local CGRP release results in plasma exudation from the membrane vessels. The mediators such as leukotriene B4 and nerve growth factor induce expression of sensory receptors, neurotransmitters and inhibitory autoreceptors. Afferent receptor sensitivity is induced by an increased expression of endothelin and bradykinin receptors, transient receptor potential vanilloid 1 (TRPV1), purinergic P2X receptors and acid-sensing ion channel 3 (ASIC3). Damaged cells release potassium and calcium. The nociceptive neurons travel to pons, turn caudally at the trigeminal spinal pathway, and end at the dorsal horns of the caudal interneurons of the first three cervical segments. Glutamate and N-methyl-D-aspartic acid bind receptors and depolarize interneurons. GRP is the neurotransmitter of the itching neurons. They cross the midline to reach lateral trigeminothalamic tract, and end at the medial thalamus. Axonal branches travel to superior salivatory nucleus, and enrich parasympathetic reflex bilaterally. This reflex stimulates muscarinic M3 receptors, and glandular exocytosis and seromucous rhinorrhea are triggered. This mechanism explains the benefit of the patients from anticholinergic medications. Tertiary thalamic nerves transmit mucosal sensation to interoceptive cortex, situated at the posterior insula. The management of these perceptions is performed by the interactions in the brain, explaining the negative effect of AR on cognitive functions at school and work. Anterior insular efferent pathways activate brainstem sympathetic (right insula) and parasympathetic (left insula) stimulation (27).

Continuance of allergic symptoms despite use of H1 histamine antagonists has led to research on other receptors. H4 histamine receptor plays role in immune regulation, and it is one of the main targets for treatment of AR. Specific H4 antagonists have been investigated by various researchers, however we do not have clear data on their clinical efficacy (28).

### 2.17. Nasal hyper-reactivity

A number of patients report that their symptoms are triggered not only by allergic stimulation, but also with non-specific stimuli including smoke, cold air and perfumes. Increased sensitivity of nasal mucosa to stimuli is called as nasal hyper-reactivity, and may be evident in patients with AR and non-allergic rhinitis. Nasal epithelial damage and increased permeability of the epithelium lead to stimulation of sensory nerve endings, resulting in mediator release from the mast cells. In addition, non-adrenergic non-cholinergic neurotransmitters (neuropeptide Y and vasoactive intestinal peptide) activate the cholinergic system that leads to nasal vasodilatation and increased secretion. Nasal hyper-reactivity may be tested with nasal provocation using cold-dry air (29).

## 3. Classification of allergic rhinitis

AR is a frequent disease affecting both adults and children. It is considered as a significant health problem due to its negative effects on school / work performance and quality of life as well as its high economic burden. The classification of AR is based on the subjective clinical symptoms of the disease. It is classified in relation with the severity (mild/moderate-severe) and duration (intermittent-persistent) of the symptoms.

Apart from its frequency, AR is a significant health problem due to its economic burden, absenteeism and comorbidities, including bronchial asthma. Classification of AR is crucial since it can be confused with other types of rhinitis, its treatment plan is based on symptoms and duration of the disease, and a common language among physicians is needed to determine the benefit from therapy. AR may be classified in accordance with the time of exposure to allergen, and frequency and severity of the symptoms (30, 31). Traditionally, AR may be divided into four subgroups according to time of exposure to the allergen.

### 3.1. Seasonal allergic rhinitis

This term is used for the disease that becomes symptomatic only in specific periods of the year, in presence of allergens in the environment. The responsible allergens are usually pollens. They are released into the air at the same time of year in regions with a moderate climate. Similarly, some mold spores increase in the summer, and cause seasonal symptoms in sensitive patients. The symptoms of some patients increase in cold seasons, and the responsible allergens may be indoor mold spores, house dust mites, and animal allergens, since their concentrations increase indoors when the inside temperature is high and windows are closed.

### 3.2. Perennial allergic rhinitis

Most of the patients have perennial symptoms. The responsible allergens may be animal fur, house dust mites and the spores of the indoor molds. The diagnosis and treatment of these patients is complicated in presence of a non-allergic rhinitis causing chronic nasal congestion.

### 3.3. Episodic allergic rhinitis

In this form of AR, the symptoms appear occasionally. Appearance of symptoms in contact with a cat in an individual with hypersensitivity to cat allergen may be an example. Another example may be becoming symptomatic after housecleaning in case of house dust mite hypersensitivity. A detailed history may help the diagnosis in this form of AR.

### 3.4. Seasonal exacerbation of chronic disease

These patients are sensitive to perennial allergens. Their symptoms exacerbate in relation with the periodical increase in the allergenic load (30, 31).

Traditional classification AR is not practical in many patients since most of the patients have multi-sensitivity to seasonal and perennial allergens. Therefore, ARIA working group of World Health Organization proposed a new classification of AR (1). In this classification, ARIA uses the terms “intermittent” and “persistent” instead of “seasonal” and “perennial”. It must be noted that “intermittent” is not the synonym for “seasonal”, and “persistent” is not the synonym for “perennial”. ARIA classification takes the severity of the disease into consideration, different from the traditional classification. The disease is classified as “intermittent” or “persistent” in relation with the duration (Table 3-1), and as “mild” or “moderate/severe” in relation with the severity of the symptoms (Table 3-2).

### 3.5. Intermittent allergic rhinitis

The term “intermittent rhinitis” indicates duration of the symptoms less than 4 days/week, or less than 4 consecutive weeks/year.

### 3.6. Persistent allergic rhinitis

The term “persistent rhinitis” indicates presence of symptoms more than 4 days/week and more than 4 consecutive weeks/year. These patients usually have symptoms every day of the year.

AR is classified as “mild” or “moderate/severe” in relation with the severity of the symptoms.

### 3.7. Mild disease

In this form of the disease, the patient has mild symptoms not influencing sleep, school or work performance, or sportive or daily activities.

### 3.8. Moderate-severe disease

This is the form of disease in which the symptoms have negative influence on sleep, school/work, leisure, or daily activities.

In the light of aforementioned information, AR may be classified into four groups as “mild intermittent”, moderate/severe intermittent”, “mild persistent” or moderate/severe persistent” in relation with the duration and the severity of the symptoms (1).

### 3.9. Local allergic rhinitis

This term is used for the patients who have classical AR symptoms in absence of systemic atopy, ie. negative skin tests and serum specific IgE (23). Most of the data on local allergic rhinitis (LAR) come from European centers. These data indicate that 47-62.5% of the patients with perennial or seasonal AR symptoms and negative skin tests and specific IgE in serum have LAR. The responsible allergens are house dust mites, grasses and olive tree pollens (32-34). Local IgE production has been claimed to play role in the pathophysiology, and has been detected in 22-35% of the patients (32, 33). LAR seen in the elderly is characterized by pronounced eye symptoms, and responds well to oral antihistamines and nasal corticosteroids (32, 33, 35). Diagnosis is based on presence of nasal specific IgE and/or a positive nasal provocation test in absence of any systemic atopy (36).

## 4. Epidemiology of allergic rhinitis

### 4.1. Global epidemiology of allergic rhinitis

AR is frequent both in adults and children all around the world. It is the 16^th^ more frequently diagnosed disorder in the outpatient clinics in the USA. It ranks as the 5^th^ most frequent chronic disease in the adults, and the first most frequent chronic disease in the children in the USA (37). It has been estimated that AR affects more than 500 million individuals worldwide. AR is most frequently seen in the adolescents, and secondly in the first decade of life (38). AR prevalence has been reported as 10-30% in the adults, and 40% in the children (39).

A study performed on 7398 volunteers (older than the age of 6 years) in the USA revealed presence of AR symptoms in one of three individuals in the previous year, independent of an upper respiratory tract infection. There was hypersensitivity for at least one allergen in 52.7% of the participants. Global prevalence of AR has been estimated as 10-20% (40).

AR prevalence shows regional differences. The prevalence in adults has been reported as 16.3% in the Switzerland while it has been reported as 23.5% in the USA (39). “The International Study of Asthma and Allergies in Childhood” report indicates regional differences in childhood, too: AR prevalence is the smallest in Iran, affecting only 1.5%, and the highest in Nigeria, affecting 39.7% of the children. The prevalence of AR has been estimated as 13-19% in children younger than 14 years of age in the USA (27).

### 4.2. Specification of the epidemiological studies and data in Turkey, and questioning their accuracy

There are only a few studies on AR prevalence in our country, and further studies on larger populations are needed. A multi-center study on 4125 individuals (age range 16-54 years, mean age 30.5 years) from every geographical region of Turkey was conducted in 44 centers. AR prevalence was found as 22.3% in adult men, and as 23.8% in adult women (41). Another study on university students reported AR prevalence as 21.8%, and the diagnosis was based on a physician report in 12.1%. AR prevalence was 17% in males, and 25.2% in females, with a statistically significant difference in between (42). A study that included 12-15-year-old students in Trabzon reported AR prevalence as 14.5%. The prevalence was higher in the girls. In addition, parental smoking, living in an apartment, and presence of a pet in the house increased AR prevalence significantly (43).

Although the data are insufficient, the results of the Turkish studies indicate various differences between Turkish population and the populations of other countries. Further studies on larger populations are needed in Turkey.

### 4.2.1. Comparison of epidemiological data in Turkey with other regions of the world

AR prevalence demonstrates regional differences in the world. The prevalence has been reported as 25% in Europe, however there are differences among the European countries. AR prevalence was reported as 28.5% in Belgium, 24.5% in France, 20.6% in Germany, 16.9% in Italy, and 26% in the United Kingdom (44). A study reported AR prevalence in Japan as 29.8% in 1998, and as 39.4% in 2008 (45). A large Middle-East study including Bahrain, Egypt, Iran, Iraq, Israel, Jordan, Kuwait, Lebanon, Oman, Palestine, Qatar, Saudi Arabia, Syria, United Arab Emirates and Yemen reported AR prevalence as 9-38% in all age groups (46). The data for Turkey are unsatisfactory, however AR prevalence has been estimated as 20-25%, with regional differences (41). The AR prevalence in Turkish adults is similar to the prevalences in other regions of the world.

Pediatric AR prevalence has been reported as 13-19% in the USA (27). A large Korean study reported childhood AR prevalence as 20.8% (47). A study compared prevelances of AR in Turkey in 2002 and 2008. Prevalence of physician-diagnosed AR was reported as 4.3% in 2002, and as 7% in 2008 (48). There are no recent studies that investigated AR prevalence in children in our country. Further studies are needed.

### 4.2.2. Specification of the regional differences in Turkey (diet, seasonal differences)

AR prevalence shows differences in our country in accordance with geographical regions, diet and lifestyle. A study that included 11,483 participants in İstanbul investigated AR prevalence in 6-7 -year-old schoolchildren, and reported once-in-a-lifetime AR prevalence as 44.3%, active AR prevalence as 29.2% and physician-diagnosed AR prevalence as 8.1% (49). A study that investigated prevalences of allergic disorders in Bolu in 30-49-year-olds reported AR prevalence as 16.5%, and noted that the prevalence was higher in individuals with low socioeconomic status (50). Other researchers investigated the influence of diet on AR prevalence in 6-7-year-old children in our country. They reported that AR prevalence was lower in children that ate grains, rice or chocolate more than three times a week. The authors did not find any influence of Mediterranean diet on AR prevalence (51). AR prevalence may show differences in accordance with geographical regions, seasonal factors and diet. Further large-scale studies are needed on this topic both in our country and in the world.

### 4.2.3. Specification of the epidemiological data in relation to age, gender, region, method of diagnosis, occupation, allergens, classification, urban/rural areas, diet (breast milk, lactose, gluten)

A number of factors may affect AR prevalence. A study that investigated AR prevalence in accordance with the age groups designated the age groups as 20-44, 45-64 and 65-84 years, and found the prevalence as 26.2% in females and 28.6% in males in 20-44-year group, as 21.3% in females and 19.8% in males in 45-64-year group, and as 17.8% in females and 17.1% in males in 65- 84-year group. The authors also reported lower AR prevalence in smoking individuals, and higher prevalence as level of education increases and socio-economic status gets better (21). A study from South Korea investigated AR incidence, and grouped the participants into 1-6, 7-12, 13-18, 19-64 and >65-year age groups. The authors found out that AR incidence increased from 2003 to 2011 (52). A meta-analysis on gender and AR epidemiology reported that AR was significantly more frequent in girls younger than 11 years of age, however it was more frequent in boys in 11-18-year-old age group. The prevalence was similar in adult women and men. Those data included the individuals from all continents except Asia (53). AR prevalence changes in accordance with gender and age.

A large-scale study from China reported AR prevalence as 13.5% in rural, and as 19.1% in urban areas. The AR prevalence was significantly higher in the urban areas (54). A study on the geriatric population investigated house dust mite hypersensitivity in the individuals living in urban, semi-urban and rural areas, and reported sensitization rates as 17.2%, 9.8% and 6%, respectively (55). A study from Poland reported prevalence of allergic diseases (bronchial asthma, AR and atopic dermatitis) twice higher in the ones living in the cities compared to the ones living in rural areas (56). A study investigated AR prevalence in 19-25-year-old female university students, and reported higher AR prevalence in the ones with high socioeconomic status. The AR prevalence was higher in individuals that had spent their childhood in urban areas. There was no correlation between estrogen levels and AR prevalence (57).

A total of 304 individuals were tested for house dust mite allergens, and AR was found in 46%, non-allergic rhinitis was found in 50%, and LAR was seen in 4% (58). An Australian study investigated food allergy epidemiology, and reported the prevalence as 11% in children aged 1 year, and as 3.8% in children aged 4 years. Specific food allergy prevalences were as follows in 4-year-old children: Peanut allergy 1.9%, egg allergy 1.2%, and sesame allergy 0.4%. AR and food allergy was simultaneously evident in 8.3% of 4-year-old children (59). A study on European and American women reported that consuming seafood during pregnancy did not increase AR incidence in the offspring. It was reported that consuming seafood during pregnancy did not increase AR prevalence (60). AR prevalence was higher in 1-4-year-olds that consumed cow milk three times a day (61). The effect of dietary habits on AR prevalence was investigated in children. The authors reported that a fat-rich, carbohydrate-poor diet increased AR incidence (62). A study on correlation of obesity with AR prevalence reported that AR prevalence increased in direct proportion to body mass index (63). High omega-3 poly-unsaturated fatty acid level in the colostrum was correlated with high AR prevalence in adolescence, however there was no correlation with high omega-6 content in the colostrum (64). It is evident that dietary habits are correlated with AR prevalence.

The children exposed to air pollution and high carbon monoxide in the city in their first year of life were reported to have higher AR prevalence at 6-7 years of age (65). A study from Sweden reported that smoking increased rhinosinusitis prevalence both in males and females, but decreased AR prevalence in males (66). Another study on AR prevalence and smoking reported that smoking did not affect AR prevalence in smoking individuals, however AR was more frequent among passive smokers (67). Although studies on smoking and AR prevalence are scarce, one may say that smoking does not increase AR prevalence.

It was reported that children with 25-OH levels greater than 75 had lower AR prevalence compared to children that had 25-OH levels lower than 50 (27). A large study from Italy also investigated correlation of vitamin D levels and AR prevalence. Although higher AR rate was present in individuals with low vitamin D levels, the result did not reach statistical significance (68). It may be concluded that vitamin D deficiency increases AR prevalence.

## 5. The influence of allergic rhinitis on quality of life

AR classification is based on clinical subjective symptoms. In ARIA classification, AR is classified as “intermittent” if the duration of the symptoms is less than 4 days/week, or less than 4 consecutive weeks/year, and as “persistent” in presence of symptoms more than 4 days/week and more than 4 consecutive weeks/year. AR is classified as “mild” or “moderate/severe” in relation of the symptoms’ influence on the quality of life (1). Since the clinical definition is based on the patient history, and it is impossible to have epidemiological data in ARIA classification, there is need for standardized questions to ask the patients (38).

Acoustic rhinometry and rhinomanometry that measures nasal obstruction, nasal nitric oxide determination to assess inflammation, and visual analog scale (VAS) that defines symptom severity are used to determine the clinical severity of AR symptoms. It has been claimed that VAS was comparable with the quality of life scales designed for AR for quantitative measurement of severity of AR (69). VAS has been used for a number of disorders. The patients are asked to mark the severity of their symptoms on a line, one end marked with 0, and the other end marked with 10. A number of studies agreed that VAS was successful for quantitating rhinitis symptoms, and it is suggested for quantitative measurement of symptom severity in AR (70-72).

Although quantitative data collection by visualization through VAS helps the clinicians for the analysis of the symptom scores, this inventory is not sufficient to determine the comorbidities of the disease and to convert them into data. In this context, quality of life scales provide a standardized and numerical summary of the symptoms of the patient, and functional and psychosocial results of the disease and its treatment, and epidemiological analysis of the data is made possible. General quality of life scales provide data for an overall functional disability and disturbance, therefore they can be used for all segments of the population, for all diseases and disorders, and for various medical interventions. Disease-specific quality of life scales are specific scales for the disease under research, and may detect small variations (73).

### 5.1. Quality of life scales frequently used for allergic rhinitis

### 5.1.1. Medical Outcome Study, Short-Form 36 (SF-36), Short Form-12 (SF-12), Short Form-20 (SF-20)

This is an overall health questionnaire used for detecting the effects of chronic conditions on functional heath status (74). The overall scale estimates physical and mental wellbeing of the individual. In case of AR, this scale was proven to differentiate healthy individuals from the patients, and it could be used successfully in the follow up of treatment (75). It is the most frequently used general quality of scale in the literature for investigation of AR patients.

### 5.1.2. Glasgow Benefit Inventory

This is a frequently used 18-item questionnaire directed to overall, physical and social benefits of the treatment employed. It is mostly used in studies on hearing surgery and hearing aids (76).

### 5.1.3. Sinonasal Outcome Test 20 and Sinonasal Outcome Test 22 (SNOT-22)

This scale consists of the questions on nasal symptoms. It measures the severity of the symptoms as well as the emotional and mental significance of these symptoms for the patients. Symptom-related comorbidities are also included. SNOT-22 includes additional symptoms, it is proven to be reliable and valid, and it is an easy-to-use, popular scale used frequently in studies on rhinitis symptoms in order to obtain quantitative data (77).

### 5.1.4. Rhinoconjunctivitis Quality of Life Questionnaire

It is the most frequently used rhinitis-specific quality of life scale (78). It measures not only the rhinitis symptoms, but also selection of the activities that rhinitis influences, and the disability regarding those activities. This scale is employed for various rhinitis groups, and it has modifications for different symptoms.

### 5.1.5. Rhinasthma Quality of Life Questionnaire

The target population of this questionnaire is the asthma patients with comorbid AR. It has been proven to be reliable in patients with simple rhinitis and comorbid allergic asthma (79). Since those two disorders co-exist most of the time, Rhinasthma Quality of Life Questionnaire is frequently used in studies on those disorders.

### 5.1.6. General Nasal Patient Inventory

This is a 30-item questionnaire for all rhinology patients. It measures quality of life in patients with any nasal disorder (80).

### 5.1.7. Sinonasal-5 Quality of Life Survey

It is a specific scale for children with persistent sinonasal symptoms. It has been used in treatment and follow up of pediatric sinonasal disorders.

### 5.1.8. Rhinitis Control Assessment Test (RCAT)

This scale is used to follow up the rhinitis symptoms after treatment. Higher scores in follow up has a significant correlation with disease control.

### 5.1.9. Nasal Obstruction Symptom Evaluation Scale

This is a nasal symptom assessment questionnaire used in adults. It is a reliable and valid 5-item nasal symptom scale that assesses nasal obstruction (81). Its validity has been proven in the follow up of the patients after surgery. It may be used in studies on sinusitis and rhinitis.

### 5.1.10. Rhinosinusitis Disability Index

This is a disease-specific questionnaire that measures the impact of the symptoms on daily activities, and their functional and emotional effects. It has been found beneficial in AR patients as well as rhinosinusitis patients (82).

### 5.1.11. Rhinosinusitis Symptom Inventory

This scale includes major and minor symptoms of rhinosinusitis.

Rhinoconjunctivitis quality of life questionnaire has been regarded as the main reliable and valid distinguishing scale for rhinitis-related quality of life (77). Other than this scale, SNOT-22 has been proven to differentiate rhinitis patients from the symptom-free individuals, and it has been suggested for the patients that had surgery. This scale has now been used by many researchers for rhinitis patients since it takes all nasal symptoms into account. It has been suggested that SNOT-22 is the most easy-to-use, specific and reliable scale (83). Turkish validation of SNOT-22 was done in 2015 (84).

The studies on the impact of AR on quality of life usually employ the aforementioned scales. A meta-analysis compared house dust mite-related perennial AR and pollen-related seasonal AR in 2016 (85). Included studies were the ones that measured health-related quality of life with generic indices such as SF-12 and SF-36, or disease-specific indices. It was reported that perennial AR due to house dust mite had a more negative impact on quality of life when compared to seasonal AR due to pollen (85). Another study on 990 AR patients found that nasal congestion and ocular symptoms influenced negatively the quality of life the most, as measured with VAS symptom scores and RQLQ, compared to other symptoms of AR. Nasal airflow measurements before and after treatment showed a significant positive correlation with total nasal symptom scores of RQLQ, even the change was minimal (86). Similarly, SF-36 provided similar results with RQLQ in AR patients after control of the symptoms with treatment (87). Both scales were found useful in the follow up, and to distinguish the patients that were actively treated with fluticasone or administered placebo. SF-36 was claimed to be as specific as RQLQ, which enclosed AR- specific items (87). RCAT, a 6-item easy-to-use scale addressing AR-related symptoms in the previous week and has been created for the follow up of AR patients was also reported to provide sufficient data in the follow up period (88).

## 6. Diagnosis of allergic rhinitis

### 6.1 History

A detailed history is crucial in AR since nasal inflammation may occur in a number of disorders. Rhinitis symptoms are similar in allergic and non-allergic rhinitis. Therefore, the specific points for AR in the history may help the physician in the diagnosis of AR.

### 6.1.1. Symptoms

### 6.1.1.1. Nasal symptoms

Rhinorrhea, nasal itching, sneezing and nasal congestion are the main symptoms of AR. Rhinitis is accompanied by eye, ear and throat symptoms. Rhinorrhea is usually copious and serous in character. Sinusitis may be evident in some patients. In this case, the patients may complain of purulent nasal and postnasal discharge, pressure on face, anosmia, headache and halitosis (89). Nasal itching is usually a characteristic of AR. Paroxysmal sneezing episodes are the most characteristic symptoms of AR, and may be accompanied by nasal itching and irritation.

Most of the AR patients complain of nasal congestion that worsens at night. Rhinorrhea, nasal itching and sneezing are mostly seen in seasonal AR, however nasal congestion is frequently evident in perennial AR. Nasal congestion may result in mouth-breathing and snoring (38). There are a number of disorders causing nasal congestion, therefore simultaneous symptoms should be questioned. The patient should be questioned whether nasal congestion is unilateral or bilateral. Unilateral nasal obstruction or rhinorrhea is suggestive of disorders other than AR. Periodicity and presence of the symptoms only in specific places or circumstances are the characteristics of AR (90).

The patients should also be questioned for the most bothersome symptom since the main symptom is important in treatment planning.

### 6.1.1.2. Non-nasal symptoms

Except for the main symptoms, the patients with AR may have other symptoms due to the systemic effects of the allergic inflammation, or presence of comorbid diseases.

Itching of palate and/or ear, postnasal dripping and dry cough are frequently seen in patients with AR. Smell and taste problems may also be evident (91).

Itching and watering eyes, eye redness and photophobia usually appears in pollen-related AR. Nasolacrimal canal obstruction due to nasal congestion contributes the severity of the ocular symptoms. Compared to non-allergic rhinitis, ocular symptoms are more prominent in AR (1).

It has been claimed that AR is a risk factor for otitis media with effusion. In this case, hearing loss, ear fullness and otalgia may be evident (92).

Paroxysmal dyspnea, wheezing and cough may appear in case of comorbid bronchial asthma (93).

“Oral allergy syndrome” or “pollen-food allergy syndrome” is a kind of food allergy characterized by itching at the mouth and throat, and it is due to cross-reaction of pollens with uncooked fruits and vegetables, various spices and nuts (94).

AR patients may have symptoms including malaise, fatigue and somnolence due to nonspecific systemic effects of the allergic inflammation. These symptoms may also be due to impaired sleep as a result of nasal congestion. Impaired sleep and rhinitis symptoms may lead to impairment of concentration which has a negative impact on school or work performance. Itchy skin may also be a symptom, particularly in the individuals with pollen hypersensitivity (95).

### 6.1.1.3. Symptom characteristics

Appearance of symptoms after getting in contact with the allergen is a characteristic of AR in hypersensitive individuals. Therefore, the patient should be questioned whether his/her symptoms appear at outdoors, home, workplace, or in contact with a pet.

Determining the time of the year when the symptoms arise, and whether they are seasonal or perennial is important both for diagnosis and treatment planning. Seasonal AR usually appears when the pollens are in the air. Perennial symptoms suggest that the responsible allergens are present in indoors, such as house dust mites. The symptoms may change and their severity may fluctuate in perennial AR. The duration of symptoms, and their persistence through the days in a week is important for differential diagnosis. AR symptoms usually persist for hours and days (90).

The age of the patient at the time of onset of the symptoms is also important for the differential diagnosis. In most of the cases, the onset of AR symptoms is at adolescence or young adulthood. Non-allergic etiology should be taken into account if the symptom onset is after 40 years of age (90).

After ascertaining the symptoms, determining and noting the severity of the symptoms is important in the follow up. The symptoms are regarded as mild if they do not have an impact on the quality of life, however in case of a negative impact on the quality of life (increased severity of coexisting asthma, sleep impairment, impaired daily activities, school/work performance) AR is regarded as severe (96).

The patients with AR may have nasal hyper-reactivity similar to bronchial hyper-reactivity, and become symptomatic when exposed to non-allergenic materials including cigarette smoke, perfumes, detergents, various chemicals, air pollution, temperature/humidity alterations and cold air (97).

### 6.1.2. Personal history

### 6.1.2.1. Occupation

The characteristics of the workplace, the equipment used, and the exposed agents should be questioned. A study on different occupational groups in Turkey reported that the allergic disorders mostly affected the ones working in textile, dye and chemistry industries as well as the ones working in an office (98). In another study on occupational allergic disorders, the authors reported that AR incidence was significantly higher among kitchen and health workers when compared to the others (99).

### 6.1.2.2. Medications

The patient should be questioned whether he/she was administered any medications for his/her symptoms, used them properly and regularly, and got any benefit from treatment, since all those are important for supporting the diagnosis and planning the treatment.

The patient should be questioned for use of any medicines for any other medical conditions.

The use of medications that may induce rhinitis (antihypertensives, antidepressants, topical decongestants, etc.) should be questioned by mentioning them individually. The correlation of the time of onset of the symptoms and the time to start the medication should be noted.

### 6.1.2.3. Previous surgery

The history of previous nasal surgery is of importance to appreciate the conditions that may cause similar symptoms.

### 6.1.2.4. Comorbid diseases

The patients should be questioned for presence of any chronic disorders including hypothyroidism (100), asthma, atopic dermatitis (101), urticaria and diabetes (102). Hypothyroidism results in non-allergic rhinitis. History of urticaria or asthma may be a reason for preferring in vitro allergic tests for the diagnosis. Obesity has been claimed as a risk factor for AR (103). Another study found AR incidence higher in children with perianal erythema (104).

### 6.1.2.5. Smoking and air pollution

Some studies reported that passive smoking increased AR risk (67, 105), however some others claimed that smoking during pregnancy, passive smoking in childhood and active smoking did not increase the risk for AR (103, 104).

A number of studies investigated the effect of air pollution on AR development. Some reported that air pollution was correlated with AR development (65, 106-108) while some others claimed absence of any correlation (109).

Exhaust fume may cause atopic sensitization and AR. Diesel motors have been claimed to be more detrimental since they yield more particles (110).

### 6.1.2.6. Place of living

The household characteristics may play role in development of allergies. Living in a slum and use of fossil fuel and biogas have been claimed to increase the risk, however use of wood/coal burning stoves did not (111). It was reported that allergy risk was lower in a household that needs less energy for heating compared to the one that needs more energy (112). Living in a city was not reported as a risk factor for allergy development, however living in a farm decreased the risk (103, 113).

The patient should be questioned for presence of a garden in his/her house. In case of living in an apartment, the floor at which the house located is of importance.

The protective measures at household (air cleaner, acaricide, bed covers, HEPA vacuum cleaner, air conditioning) should be questioned.

### 6.1.2.7. Pets

Having pets is common particularly in the cities. Although the furs of the cats and dogs act as reservoirs for allergens, the allergen sources are their glands, saliva and urine. The allergens may remain in the household for weeks and even for months. A number of researchers investigated the relation of animal allergens and AR, however the results were conflicting. It was reported that early exposure to animals was protective for AR, this was a risk factor for AR, or did not affect AR development (104, 114-117).

### 6.1.2.8. Food allergy

Hypersensitivity to food allergens during pregnancy or early childhood has been correlated with AR development (118). It was claimed that presence of food allergy and atopic dermatitis in early childhood was a risk factor for development of other allergic disorders later in life (101). Consumption of more sugar and small amounts of vegetables had significantly increased AR prevalence (104).

### 6.1.3. Family history

### 6.1.3.1. Number of the siblings

AR prevalence was lower in children with older siblings and the ones living with large families (119).

### 6.1.3.2. Household

The household at infancy has been claimed to affect AR development. Moving into a new house may increase the risk for AR. It was reported that increased risk might be related to new chemicals (120). AR risk is higher in concrete homes with polyvinyl chloride window frames, central heating, and visible molds in the house (121).

The results of the studies on socioeconomic status and AR development are controversial. Although most of the studies claimed that high socioeconomic status was correlated with AR, some others claimed the opposite (122). The data suggest that the children living in families with high socioeconomic status have a higher risk for AR (48, 57, 123-125).

High humidity has been shown to increase AR occurrence (104).

### 6.1.3.3. Childhood history

Some factors during prenatal and postnatal periods and pregnancy may play role in AR development. Excessive exposure to allergens during pregnancy and use of oral contraceptives have been claimed to increase AR risk in the offspring. Use of proton pump inhibitors, H2 receptor blockers, antibiotics and paracetamol may also increase AR risk in the offspring (104, 126, 127). Consumption of coloring material- or sweetener-added beverages extensively during pregnancy may increase the risk for AR (128). The results of the studies that investigated the correlation of mother age and multiple gestations on AR risk yielded conflicting results as well as the ones that investigated the season of the birth and allergenic sensitization. Some authors reported higher AR risk if the baby was born in spring or fall (129).

AR risk was found higher in the ones that were born with a Cesarean section, and it was claimed that the baby was more susceptible to food allergens and aeroallergens since it was not exposed to vaginal flora during birth (119).

Some studies concluded that hospitalization in the neonatal period, neonatal jaundice and phototherapy increased AR risk (130).

Feeding the baby with mother’s milk was reported to avoid AR. Meta-analyses showed that the babies fed with mother’s milk developed AR less frequently later in life (131, 132).

The studies that investigated the correlation of AR with exposure to house dust mites in prenatal period or early childhood reported conflicting results. Most of them did not correlate AR with exposure to mites early in life (116). Most of the studies did not confirm exposure to fungal allergens in prenatal period or early childhood was a predisposing factor for AR (108, 133, 134).

Upper airway infection and sinusitis in childhood were claimed as risk factors for AR (104).

### 6.1.3.4. Family history of atopy and allergic diseases

There is a familial tendency for AR. Approximately 59% of the allergic patients have positive family history. The risk of developing an allergic disease in the child is approximately 47% if both parents are atopic. This risk is 13% if none of the parents are atopic, and approximately 29% if one of the parents is atopic. The risk of developing AR is 4-6 times more if the individual is asthmatic (103, 135). Family history of atopy, food allergy and eczema has been reported to increase the risk for AR (104).

### 6.2. Physical examination

**Keywords:** Rhinitis, Allergic. Physical examination

### 6.2.1. Nasal signs

There are no specific nasal findings for AR. Otoscope, nasal speculum, and rigid and flexible endoscopes may be used for nasal examination (136).

The patients with AR may have mouth breathing, sniffles, hypernasality and allergic salute, i.e. wiping and/or rubbing the nose in an upwards or transverse manner with the palm. A horizontal supratip crease may appear over the nose as a result of allergic salute (137).

Nasal examination may be normal when there is no seasonal allergen exposure. Thin and colorless rhinorrhea, mucosal edema of the turbinates, serous secretion extending between the lower turbinate and nasal septum, purplish or pale nasal mucous membranes, and maceration at the nasal vestibule may be seen on nasal examination (138-140). Nasal endoscopic examination may reveal polypoid lower turbinates and nasal polyps in addition to turbinate hypertrophy, however those findings are not specific to AR (141).

### 6.2.2. Ocular signs

Chronic spasm of the Muller muscle due to venous stasis and hypoxia may head to horizontal lines, called as Dennie–Morgan lines on the lower eyelid. In addition, “allergic shiners” may be evident in the lower eyelid. Allergic shiners refers to hyperpigmentation of the lower eyelid skin, appearing as dark circles. The reason for this finding is disruption of venous blood flow in the periorbital region due to nasal mucosal congestion, pooling of blood around the eyes, capillary leak, and subcutaneous deposition of hemosiderin. Sclera may get thicker due to increased vascularity of the conjunctiva. Increase in ocular secretions, conjunctivitis, thinning of eyelashes, and scaling of the eyelashes may be evident (96, 141).

### 6.2.3. Other signs on otorhinolaryngological examination

Eczematous appearance of the external ear canal, postnasal discharge, hypertrophy of tonsils and lateral pharyngeal bands, posterior pharyngeal erythema and edema, granular oropharyngeal pharyngitis due to irritation of postnasal discharge, and vocal cord edema may be seen (96, 142). Adenoid face, maldevelopment of dental arc and palate, and tooth decays may be evident in children owing to chronic mouth breathing (142).

### 6.2.4. Complications

AR may lead to physical and mental complications particularly in children. Otitis media with effusion may occur in the short term, however chronic rhinosinusitis, asthma, orthodontic malocclusions, nasal polyposis and obstructive sleep apnea syndrome may be evident in the long term as physical complications. Mental complications include impairment of school performance and hyperactivity (143, 144). 

### 6.3. In vivo tests in the diagnosis of allergic rhinitis

An international literature search was performed with the keywords “allergic rhinitis, in vivo testing and diagnostic testing” in Pubmed, Scopus, Google academic and Thomson Reuters databases. Only reviews and meta-analyses were taken into account until 2008 (7 publications). All publications on in vivo testing for AR have been included between 2008 and 2018 (156 publications). The abstracts were reviewed to eliminate the ones that were not directly on in vivo tests or diagnostic tests, and a total of 45 publications were reviewed. At the end, a total of 52 international publications were included in the study. National literature search was done on Ulakbim and Google academic databases with the keywords “alerjik rinit, in vivo testler, tanı testleri”, without any time limit.

### 6.3.1. Introduction

There are three types of in vivo skin tests used in the diagnosis of AR:

1. Skin prick testing (SPT): This is the primary test for the diagnosis of IgE-mediated allergy. It has been frequently employed. Although very low, it may lead to severe complications. It provides valuable information if done and interpreted correctly.

2. Intradermal testing (IDT): Used in the diagnosis of both IgE-mediated and delayed-type hypersensitivity reactions. Its complication rate is higher if used for the diagnosis of immediate, namely, IgE-mediated allergy, therefore its technique and interpretation necessitates expertise.

3. Patch testing: Used for the other forms of delayed hypersensitivity, including contact dermatitis. It is primarily performed by dermatologists and some immunologists. This test will not be discussed herein since its role is limited in the diagnosis of AR.

4. Scratch test: This test is not performed anymore.

SPT provides information on presence of specific IgE against peptide antigens (allergens). It is based on application of a small amount of allergen into epidermis and avascular dermis to enable reaction with the specific IgE binded on the cutaneous mast cells. Histamine and other mediators released from the mast cells give rise to a visible “erythema and induration” skin reaction 15 minutes after the application of allergen.

The quality of the test results depends on some steps, as follows (145):

• The relevance of the used allergen with the investigated allergic condition

• Application of sufficient amount of natural allergen into the skin in the correct manner

• The functional status of the cutaneous mast cells

• Correct interpretation of the result in the context of positive and negative controls

When performed correctly, SPT has high specificity and sensitivity for determination of allergen specific IgE. It may sometimes be more sensitive than in vitro specific IgE testing (146). It does not cause much discomfort in the patients, and the risk of systemic reaction is very low (147).

IDT is primarily used in the diagnosis of venom allergy and IgE-mediated drug allergy, particularly penicillin hypersensitivity, and its use requires more knowledge and expertise. It carries a higher risk for anaphylaxis compared to SPT, and it is usually performed in a hospital (148).

AR diagnosis is based on patient history, clinical examination, and SPT or in vivo testing of serum for specific IgE. In case of any uncertainty in the diagnosis, other tests may be employed taking non-allergic conditions into account (149).

SPT has various advantages since it is a fast and cheap test, and it provides a visual result for the patient. It should be a routine test for atopic individuals in whom the responsible allergen needs to be determined. SPT should not be used in the patients with dermographism or eczema, as well as in the ones who are on histamine-receptor-blocking medications (antihistamines, corticosteroids, tricyclic antidepressants). In this case, in vitro tests should be preferred. Skin tests performed using standardized inhalant allergens are quite safe.

### 6.3.2. Evaluation before diagnostic testing

### 6.3.2.1. Indications for skin prick test

Indications for SPT are listed below:

• Rhinitis / rhinoconjunctivitis / rhinosinusitis / allergic conjunctivitis

• Asthma

• Atopic dermatitis

• Food allergy causing anaphylaxis, urticaria or acute eczema

• Suspicion for latex allergy

• Allergic bronchopulmonary aspergillosis, eosinophilic esophagitis

Selection of the allergens depends on the condition to be diagnosed, and the risks for exposure to potential allergens. SPT is not recommended in conditions in which low-molecular-weight substances are thought to be responsible for allergy. These conditions include allergy for food additives, non-allergic adverse reactions of medicines, airway irritants, and most of the occupational allergies (the details will be discussed later).

### 6.3.2.2. Indications for intradermal test

Indications for IDT are listed below:

• Venom allergy

• Immediate allergic reaction due to beta-lactam antibiotics and medicines with a valid protocol

• Immediate allergy due to vaccines

IDT should be performed by a health professional with sufficient knowledge and expertise. IDT is not indicated for aeroallergens, and contraindicated for routine diagnosis of food allergy (148).

Allergy tests have been shown to improve the accuracy of the diagnosis when the results are anticipated together with patient history (150). They are useful to rule out the conditions that cause symptoms similar to allergic disorders.

Allergy testing makes allergen avoidance, realistic use of medicines, and allergen immunotherapy possible in some cases. SPT is strongly recommended when the physician finds strong evidence for the benefit of allergen avoidance or allergen immunotherapy.

SPT may also be employed for epidemiological research or determination of atopy not related with specific disorders.

### 6.3.2.3. Patient selection for skin prick test

### 6.3.2.3.1. Age

Although there is no age limit for SPT, one should consider that children and elderly have less skin sensitivity, and interpretation of the results is hard in this case. Babies usually have smaller indurations and larger induration reactions. Rarely, systemic side effects may appear in children (as occurs at every age). Experienced specialists should perform SPT to children younger than 2 years of age due to higher risk for complications and interpretation difficulties (150).

### 6.3.2.3.2. Contraindications

Contraindications for SPT are (151):

• Dermatological disorders at the possible sites to be used for SPT (the test should be done on a normal, healthy skin)

• Severe dermographism

• Poor patient compliance

• The patients who cannot stop antihistamines or other medicines that may affect the test results

### 6.3.2.3.3. Relative contraindications

These relative contraindications may be related to physical conditions of the place where test is performed, or the health professional that performs the test (151).:

• Severe persistent or unstable asthma

• Pregnancy (due to the risk of anaphylaxis that causes hypotension and uterine contractions, although rare)

• Babies and infants

• The patients on beta blockers

### 6.3.2.3.4. Medications that affect skin prick test results

A number of medications may decrease skin reactivity. The patient should be questioned for their use before the test, when making the test rendezvous (Appendix 1). First generation antihistamines usually change the skin response for a relatively short time, however second generation antihistamines change it for a longer time. So that the antihistamines should be stopped 10 days before a skin prick test. The suppression of skin test result shows differences among the members of the same class of medicines, and also among the patients. Tricyclic antidepressants, such as doxepin, have antihistamine activity, and should be stopped 1-2 weeks before the test (152).

Phenothiazines also have antihistamine activity. Oral corticosteroids do not affect skin reactivity even after long-term use, however long-term use of intranasal corticosteroids has been shown to decrease skin reactivity (153, 154, 155).

### 6.3.2.3.5. Patient factors that affect skin prick test results

Dermographism may lead to false positive erythema and induration after SPT. An induration may appear at the test site of the negative control. If the induration of the allergen is not bigger than the induration of the negative control, it may be hard to comment on the SPT results. Mild dermographism does not affect SPT results. Some techniques used for performing SPT may trigger dermographism (151).

Some conditions may alter SPT results. Being elderly, performing the test during the menstruation period, the race of the patient, circadian rhythm, the season, and atopic dermatitis (even its presence in another part of the body) are some examples (151).

Presence of some disorders may decrease shin sensitivity: chronic renal failure, cerebrovascular disorders, malignancy, spinal cord injury, diabetic neuropathy, and recent anaphylaxis. The SPT should not be performed on the extremities with lymphedema, paralysis or neurogenic disorders (151).

Some recent studies reported that respiratory syncytial virus infections increased histamine release, and false positive skin test results might be obtained in the patients infected with this virus. Therefore, the test results should be interpreted carefully in presence of an acute viral infection (156).

### 6.3.2.4. Intradermal skin test

The allergens are injected intradermal to produce a small swelling in the skin. The increase in the size of the induration is examined 20 minutes after the injection. The injected allergens should be diluted 100 – 1000 fold compared to the concentration used in SPT. Using a correct injection technique and a proper interpretation of the result are important. The tester should keep systemic reaction risk in mind, including anaphylaxis. The risk is higher than SPT, although rare (157). IDT should be performed by specialists, and if possible, in a hospital.

IDT is contraindicated for the diagnosis of food allergy, and its benefit is limited in case of allergies caused by inhalant allergens, due to its low specificity (158, 159). SPT has been shown to have a greater correlation with the symptoms, compared to IDT (160). IDT is useful in the diagnosis of penicillin hypersensitivity, and it is also used in the diagnosis of other drug hypersensitivities including insulin, opiates, anesthetic agents and muscle relaxants (161). Although its clinical predictive value is not clear, it may be used in the diagnosis of bee venom hypersensitivity (162). IDT has been used in USA in the routine diagnosis of allergies, however its use is limited in Europe and Turkey.

### 6.3.3. Method

### 6.3.3.1 Allergens for skin prick test

### 6.3.3.1.1. Commercial extracts

These allergen extracts are produced specifically for SPT. They are aqueous solutions of the protein extracts obtained from the allergens, and 50% glycerol is added as a preservative. Therefore, they are quite viscous. They are sold in small bottles with a dropper.

The commercial allergen extracts for SPT are not produced in Turkey. There are only a few international producer and retailers. The allergen extracts used in our country are produced by Hollister-Stier (USA), Stallergenes (Europe) and ALK-Abello (Europe and USA).

### 6.3.3.1.2. The contents of skin prick test extracts

The commercial allergen extracts should contain all allergenic proteins labeled on the bottle. However, they should not contain any allergenic proteins that cause a cross-reaction. For example, the allergen extract of one plant pollen should not be contaminated with the pollen of another plant. Some allergen extracts contain a mixture of the allergens, and this is labeled on the bottle. Examples may be the pollens of various grasses in one bottle, the pollens of various trees in one bottle, or different Alternaria allergens in one bottle.

Some allergenic extracts have standardized allergenic potencies, while some others are prepared in regard to the weight of the allergen.

Allergen extracts are complex mixtures. They contain a series of allergen proteins separated by electrophoresis, and visualized by immunoblotting. The extracts of different companies may contain different amounts of major allergen. This is why the products of different companies may produce different SPT results. This is also the main reason for obtaining different results with SPT and serum specific IgE measurement. Before interpreting the results of the studies, one should take whether the investigators used standardized extracts in the study (163).

Allergenic substances contain hundreds of different proteins with unique designs. Only a subgroup of these proteins have allergenic potential. However, individuals may produce IgE for different proteins in the allergenic material. If the protein in the test material does not have the same protein sequence with the allergen that induced IgE in this individual (due to production process or protein instability), the test will give a false negative result although the patient has allergy. The aforementioned entity is a potential reason for a false negative SPT (164).

### 6.3.3.1.3. Cross-reaction

Cross reaction is an important concept while choosing the allergen extracts for SPT and interpreting the test results. Cross reaction refers to reactivity of a specific IgE to a similar allergen, other than its specific allergen. The patient may have not ever been exposed to the second, similar allergen. Cross-reactivity of pollen and other allergens is mostly related to phylogenity, but there are also some biologically unpredictable models of cross-reactivity due to proteins that have conserved their structures across various species (165).

### 6.3.3.1.4. Allergen test panel

The allergen test panel should be relevant to the clinical picture of the patient as well as the allergenic exposure. The number of the allergens in the panel should be kept at minimum, just sufficient for the diagnosis and treatment. Allergen panels with a relatively small number of allergens (8-12 inhalant allergens) are considered as adequate. However, a detailed test with more allergens may be needed if allergen immunotherapy is an option, or allergy for a rare substance is explored. The test panels must be prepared in accordance with the flora and fauna of the region (166). On the other hand, every clinic has its own routine. The number of the test allergens needed for SPT has been reported between 6 and 60 in different studies (167). It is not cost-efficient to use a large allergen panel in a small center with small number of patients.

We suggest you to take pollen map of Turkey into consideration as well as the pollen calendar before planning an inhalant test panel in your center (168).

### 6.3.3.1.5. Food allergens

SPT may be used to test IgE-mediated food allergy, however interpretation of the results is difficult. The test results may be positive, however no clinical correlation may be evident. This may be due to various factors, on the other hand, the test may be negative in presence of positive clinical findings (169). The anaphylaxis risk is higher when compared to SPT performed with aeroallergens (170). IDT is not indicated in case of food allergies (171). There are commercial extracts, but they are not standardized. Sometimes performing the SPT with the fresh food or the food itself gives better results. Food allergy testing should be performed by experienced healthcare professionals due to risk of adverse effects and difficulty of interpretation of the results (172).

### 6.3.3.1.6. Storage of the allergen extracts

The constituents of the allergen extracts should be clearly labeled on the bottle. They are usually sold in a bottle with a dropper. The allergens are proteins in nature, therefore they need a refrigerated transport, and should be stored in the refrigerator. Their expiration date should be checked before use. Precautions should be taken to avoid bacterial contamination of the extracts as well as cross-contamination between the allergen bottles.

The practical measures listed below are recommended:

• Number the test bottles and align them in a row over a shelf

• Open only one bottle when performing SPT. If you place the dropper over another bottle, the allergens cross-contaminate. In this case, the bottle and the dropper should be discarded

• In order to avoid bacterial contamination of the tip of the dropper, cleanse the skin surface with alcohol. Apply the test only on the normal, healthy skin. While dropping the allergen extract on the skin, do not let the tip of the dropper touch the skin; however, the extract drop may touch the skin

### 6.3.3.2. Positive and negative controls

Some patients may have dermographism, and an induration may appear just by pricking the skin, even if an allergen is not used. This may lead to misinterpretation of the test result, and a false positive SPT. The negative and positive controls should be examined very carefully in this case. If the indurations of the negative and positive controls are equal, the test result cannot be interpreted. On the other hand, if the negative control is bigger than the positive control, the allergen results should be interpreted by comparing the allergen’s induration with the negative control. As an example, if negative control’s induration is 3 mm, the allergen indurations bigger than 6 mm should be regarded as positive. Since dermographism response may be inconsistent in different regions of the body, and false positive SPT results may occur, the tester should be careful while interpreting the results. The test should be regarded as “invalid” if the induration of the negative control is bigger than 3 mm. A delicate pricking technique may minimize the nonspecific reaction in patients with dermographism.

The positive control should produce an erythema / induration sizing approximately 7 mm. Use of antihistamines or other medications with antihistamine activity (Appendix 1), or a nonreactive skin should be considered if the erythema / induration size is smaller than 7 mm. SPT is not valid in this case. An erythema / induration ≥4 mm in the positive control is an acceptable result (or an erythema / induration 4 mm larger than that of negative control). The test is regarded as invalid if the induration of positive control is smaller than 4 mm.

Negative control does not contain any allergen (normal saline or 50% glycerol solution). Negative controls of SPT extracts are commercially available. The positive control may contain histamine (usually histamine phosphate 10 mg/mL, it directly induces erythema and induration), or codeine (usually 9% solution, it indirectly degranulates cutaneous mast cells and causes a skin reaction).

### 6.3.3.3. Equipment for skin testing

For SPT the skin is pricked using a sharp lancet through the allergen extract for penetration of the allergen into the epidermis and superficial dermis. The skin may be pricked by a lancet or special applicators. The special applicators are designed to apply 5 or 8 allergens at the same time. The applicator is first submerged into the allergen extract, then applied over the skin to prick it. Lancet technique is used more frequently. In this technique, after the allergen extract is dropped on the skin, then the skin is pricked through the extract with a lancet. The skin should not be over-pricked with the lancet in order to avoid bleeding. A new lancet should be used for each allergen extract in order to avoid mixing of the allergens (173, 174).

### 6.3.3.4. Performing a skin prick test

A summary of the minimal and optimal requirements in SPT, circumstances that requires experience, and contraindications are given in Appendix 6.3.7.4.

### 6.3.3.4.1. Equipment needed for skin prick testing

The equipment needed for SPT is listed below (175);

• Allergen extract

• Positive and negative control solutions

• Sterile lancets for pricking the skin

• A “sharp medical waste container” to dispose the lancets

• A pen to mark the skin

• A ruler to measure the sizes of the skin reactions

• Test report

The patient sits in an armchair. The arms of the patient should be in a suitable position for the tester. The patient is informed about the procedure (an informed consent form may be used). The test region is cleansed.

### 6.3.3.4.2. The area of the body used for skin prick testing

The most suitable and frequently used part of the body is the plantar surface of the forearm, or the dorsal surface of the upper arm. The test region should be 5 cm superior to the wrist, and 3 cm inferior the antecubital fossa (176). Skin pricks should be away from superficial vessels and skin lesions.

### 6.3.3.4.3. Test method

Although skin cleansing with alcohol before SPT is not obligatory, it is recommended (cleansing with alcohol may be contraindicated in case of an extremely dry skin or presence of eczema). The sites of the allergen extract drops are marked with a pen before dropping them. The drops should be at least 2 cm away from each other in order to avoid false positive results and overlapping erythema / induration reactions (176). If a multitest applicator is used, the upper side of the applicator should be marked on the test site. The multitest applicator sites should also be numbered if more than one applicator is used.

### 6.3.3.4.4. Waiting period before reading the results

The skin reaction of positive control, i.e. histamine, reaches its maximum size approximately 10 minutes after application, however the skin reactions for the allergens take 15 minutes to reach their maximum sizes. Therefore, it has been recommended to evaluate SPT results 15 minutes after application of the test material (177).

The skin reactions of some allergens may enlarge up to 20 minutes. If test evaluation is done after 20 minutes, both histamine and allergen skin reactions may diminish, and a re-test may be needed. The test is evaluated and reported if the reaction of the positive control is bigger than the negative control, and/or positive control’s induration is bigger than 3 mm. The positive control’s skin reaction is usually measured as 7-9 mm.

### 6.3.3.4.5. Measuring erythema and induration

The standard and recommended method for evaluation of the SPT reactions is measuring the average diameter of the induration with a transparent ruler, compass or calipers designed for this purpose. Measurement of the diameter is sufficient if the induration is round in shape, however the shortest and the longest axes should be measured and their average should be calculated if its shape is irregular or oval. Erythema is measured with the same method. In case of overlapping skin reactions, only the width of the non-overlapping part should be recorded. The pseudopods are not included in the diameter of the reaction; however, their presence should be noted in the report. Their significance is uncertain, and they have been supposed to appear owing to irregularities in the skin prick. Some authors have recommended measurement of the longest diameter or use of a planimeter to obtain the area of the reaction in mm^2^ (165). On the other hand, measuring the average diameter is easy, and it should be regarded as the standard reporting method (178).

The physician is recommended to see the skin reactions before reporting, if the SPT is performed by a nurse or a technician, in order to increase the quality and determine the need for retesting. As an example, the test should be repeated if skin reactions of the allergens that cross react (D. pteronyssinus and D.farinae, or grass and cereal pollens) show a clear discrepancy.

### 6.3.3.4.6. Recording skin prick test results

The size of the skin reaction should be recorded by the name of the allergen on the report. The reaction size may be noted in millimeters, or graded as +, ++, +++ or ++++. These two methods are widely used in the clinical practice. This subject is detailed in section 6.3.3.5.

### 6.3.3.4.7. Follow up of the patient after skin prick testing

Itching due to allergen reactions may irritate the patient after SPT. The allergen extract should be cleansed with alcohol at the end of the test unless there is a contraindication for use of alcohol (dry skin or a skin disorder in which alcohol use is contraindicated). Itching usually lessens in 15 minutes. Topical crèmes or cold application may decrease itching. Topical corticosteroids are not beneficial (179). Oral antihistamines may be given. There is no evidence for the relative benefits of aforementioned methods. The patients should be warned about late-phase reactions, although those are more frequently seen after intradermal test, and not after SPT.

It is recommended to keep patients under supervision after the test, due to risk of systemic reactions, although rare (170). This supervision may not be necessary if the SPT is negative, the patient is not an asthmatic, and a moderate skin reaction is observed for aeroallergens. On the other hand, the patient should be kept under supervision for at least 40 minutes after the test in case of multiple allergies on SPT, a previous history of anaphylaxis, and in presence of asthma. The risk for a systemic reaction is higher in case of severe asthma, use of beta blockers, pregnancy, an intradermal test is performed, or the patient is tested with latex or food allergens (180).

### 6.3.3.5. Reporting skin prick test results

SPT report should be clear and understandable for other physicians. The SPT report should cover the following:

• The name, address and correspondence of the physician

• The name and date of birth of the patient

• Date

• The body region where SPT is applied (back, forearm, etc.)

• The name of the allergen applied (the name written on the extract bottle should be written, any common or local name should not be mentioned)

• Negative and positive controls

• The size of the reaction for every allergen

The longest diameter of the allergen reaction and the diameter perpendicular to this line are measured, summed up, and the mean of them is written on the SPT report. The induration and erythema diameters are measured with a transparent, flexible ruler.

The SPT results may be reported between 0 and 4+ in accordance with the diameter of the allergic induration:

• < 3 mm: (-)

• 3-5 mm: (+)

• 5-7 mm: (++)

• 7-9 mm: (+++)

• ≥10 mm: (++++)

The reaction size of allergen may be compared to the reaction size of histamine for reporting purposes (181):

• Induration diameter is smaller than half of induration diameter of histamine: (+)

• Induration diameter is equal to half of the diameter of histamine’s induration: (++)

• Induration diameter is equal to the diameter of histamine’s induration: (+++)

• Induration diameter is 1.5 fold of the diameter of histamine’s induration: (++++)

### 6.3.4. Evaluation of skin prick test results

### 6.3.4.1. The significance of positive and negative results

The patient’s life style, diet, and even occupation may need modifications in accordance with the SPT results. A long-term treatment or expensive allergen avoidance measures may be founded on these results. Therefore, the physician should meticulously examine the allergen reactions, take other clinical factors into account, and interpret the SPT carefully.

The SPT results should be interpreted by taking the history, signs, and allergen exposure of the patient into account. In the presence of an allergic disorder (such as the ones listed in section 6.1), the physician may consider the positive allergen in SPT is associated with the symptoms if this allergen is related to the patient’s allergen exposure, and the symptoms flare up with exposure to this allergen, as mentioned in the history of the patient. Any SPT result uncorrelated with patient history should be handled with suspicion.

SPT is a quite accurate and specific test for detection of allergen-specific IgE when performed correctly. On the other hand, presence of an allergen-specific IgE does not prove clinical reactivity of an individual to this specific allergen. Generally speaking, a bigger allergen reaction in SPT predicts a greater reaction to this allergen on exposure, but not the symptom severity (182). It has been shown that an allergen reaction greater than 3 mm is well correlated with clinical allergen reactivity. For example, an induration greater than 6 mm for house dust mite may be more specific for diagnosis of clinical house dust mite allergy compared to an induration size of 3 mm. On the other hand, it should be kept in mind that distinctive allergens and allergen extracts obtained from different companies may yield different results on the same individual. Therefore, every SPT result should be interpreted in the light of the clinical picture of the patient.

A positive SPT indicates presence of specific IgE. On the other hand, exposure to that allergen may not induce symptoms. This is called as “clinically silent hypersensitivity” or “clinically false positive test result” (this individual may still be classified as atopic). The size of the SPT reaction may correlate with probability of the clinical reactivity to that allergen (183). In conclusion, the size of the skin reaction in SPT is usually not correlated with symptom severity.

A positive SPT does not predict the nature of the allergic symptoms. Various individuals with a positive reaction to the same allergen may present with different symptoms on exposure to that allergen.

SPT may be negative due to insufficient allergenic protein content of some allergenic extracts, even if the patient has specific IgE. Negative SPT result does not rule out development of an allergic disorder in the future.

Technical or tester-related mistakes may also lead to false positive and false negative SPTs. In this context, one should keep in mind that a false positive or false negative test result cannot be reproduced in an individual on re-testing.

SPT is not used in the diagnosis of a non-IgE-mediated allergic disorder or intolerance. It is clear in some individuals that the adverse reaction is not mediated by a type 1 (IgE mediated) allergy. Other mechanisms should be considered in case of an adverse reaction in history, and a negative skin test.

### 6.3.4.2. The value of skin prick testing

Every test has its own “performance characteristics” with regard to sensitivity, specificity, and positive and negative predictive values.

The studies that investigated diagnostic value of SPT have shown different evidence levels. Common limitations of those studies bias in choosing the study group, absence of a suitable gold standard method, and lack of blinding. In SPT, allergen reactions and their sizes may vary when different commercial allergen extracts are used, and the test is repeated in the same individual (184).

The specificity and sensitivity of SPT has been reported as 70-95% and 80-97%, respectively, for the diagnosis of inhalant allergy. The positive predictive value of patient history alone is 77% in the diagnosis of persistent allergic rhinitis, however this value increases to 97-99% when a SPT is performed (185). Therefore, positive and negative predictive values of SPT may be considered as 97% and 90%, respectively (186-188).

Optimal evaluation of SPT result is particularly important to avoid unnecessary allergen avoidance, dietary restrictions, use of medications, as well as long-term treatment with immunotherapy.

### 6.3.5. The team

### 6.3.5.1. Physician

The role of physician in allergen SPT:

• Be sure that the conditions of the test room are suitable, and educated personnel, equipment and allergen extracts are ready

• Consider the patient, patient history and physical examination findings, reconsider deferential diagnosis and test indications, and check whether SPT will provide any additional information, or the results will affect the treatment options

• Carefully consider the contraindications for the test and the factors that may influence the test results

• Inform the patient about the benefits and the risks of SPT

• Choose the allergens in the test panel taking the symptoms and allergen exposure of the patient, and the common allergens in your region into account

• Inspect the skin region that will be used for SPT (back, forearm), and prefer forearm

• Be sure that the tester has sufficient information on SPT, and can finish the test safely if the test is to be performed by someone else, other than a physician. Stay close to the test room, be ready to treat complications

• Analyze the clinical importance of positive test results and false negativity in case of negative allergen responses

• Determine the diagnosis and treatment plan

• Share the test results and the treatment plan with your patient

### 6.3.5.2. Other medical staff

Educated and experienced nurses and other medical staff may help the physician while performing SPT, and in some other stages of treatment process. The patient should be informed about the test and its possible consequences, and he/she should be comforted before applying the test. An educated and experienced nurse or health technician may apply the test under supervision of the physician, and helps for documentation. The nurse or technician should be educated and ready for a probable complication. He/she should be capable of educating the patients for allergen avoidance and use of an adrenaline autoinjector (EpiPen or Anapen), if the physician needs such a help.

### 6.3.6. Safety and risks

### 6.3.6.1. The safety and risks of skin prick test

SPT is a safe procedure that does not disturb the patient much.

On the other hand, some adverse reactions may appear, although rare (11). Those may be classified as allergic, allergic but unrelated with the test, and non-specific events. An example for test-related non-allergic adverse reaction may be the risk of infection (although has not been reported up to date, this complication may appear). The examples of non-specific reactions are syncope and headache (189). Vasovagal syncope is relatively frequent, and if the test is performed in sitting position, the test room should have the facility to place the patient in supine position.

The expected skin reaction in SPT is a localized swelling and itching. The localized skin swelling usually appears as an IgE-related late phase response (therefore it usually occurs after IDT) (190). Swelling usually subsides before 36 hours.

Allergens may unintendedly pass into the systemic circulation in SPT. Typical symptoms of anaphylaxis including widespread urticaria, angioedema, bronchospasm and hypotension may appear. These systemic reactions are usually mild, and respond to standard treatment. Although rarely reported in large series, a number of papers have reported systemic allergic reactions (191). In a study including 16,000 patients, the adverse reaction rate was reported as 0.04% after a SPT with eight standard allergens (192). Most of those reactions were syncope and malaise. Systemic allergic reaction rate was reported as 0.033% in a larger series (193).

A few deaths were reported after IDT, and only one death was reported after SPT (the patient had most of the risk factors listed below) (166). Late systemic reactions and late-phase allergic responses were reported particularly in asthmatics. Asthmatics should be followed up closely after SPT, especially if they have large positive skin reactions.

Systemic reactions occurred particularly in children who had atopic dermatitis and younger than 6 months of age, when tested with food allergens (194).

The risk factors of anaphylaxis in SPT (166):

• Infants and young children (may appear at every age)

• Testing with food allergens in the individuals with a previous history of food anaphylaxis

• Testing with fresh food and non-commercial allergen extracts

• Testing with latex allergen

• Presence of asthma (particularly if the disease is active or unstable)

• Widespread atopic dermatitis in children

Since both atopic dermatitis and asthma are frequent disorders and systemic adverse reactions are very rare after SPT, the physician should not be reluctant to perform a SPT on these patients after having sufficient knowledge of these disorders, and taking all necessary precautions.

### 6.3.6.2. Safety rules and equipment

SPT should be performed in a healthcare facility in which a medical team authorized to treat systemic allergic reactions are ready. It is recommended to follow up the patients with positive SPT, the ones with asthma or previous anaphylaxis for at least 20 minutes after completion of SPT (40 minutes after skin pricking) (195, 196).

The minimum standards of emergency equipment and medications (195, 196):

• Oxygen (6 L/min using a mask)

• Intravenous fluids for fast infusion in case of hypotension

• Adrenaline for intramuscular injection

• Salbutamol for use with nebulization or inhalation

Detailed information on treatment of systemic allergic reactions and anaphylaxis is out of scope of this chapter.

### 6.3.6.3. Informed consent form

Most of the studies did not give any information whether their patients filled in an informed consent form before SPT. The informed consent form we recommend is given in Appendix 6.3.7.5 and Appendix 6.3.7.6.

**Keywords: **allergic rhinitis, in vivo testing, diagnostic testing

### 6.4. In vitro tests for diagnosis of allergic rhinitis

In parallel with the recent technological developments, a number of laboratory test methods have been developed and routinely used for diagnosis of AR. Laboratory tests are widely used in the diagnosis of AR. Generally speaking, laboratory tests used in AR diagnosis intend to identify IgE.

In vitro tests have some advantages over in vivo tests in some special circumstances:

• In the elderly with cardiovascular disorders, since in vitro tests do not bring any risk for a systemic allergic reaction

• In patients who have the risk for a severe anaphylactic reaction

• In the patients that cannot stop their medications, since medications do not alter in vitro test results

• In infants (<12 months) that cannot produce a satisfactory skin test response (false negative)

• In post-anaphylactic period

• In patients with severe and extensive atopic dermatitis or dermographism in whom skin tests may be false positive (198).

### 6.4.1. Serum total IgE level

IgE is an antibody produced in response to a threat perceived by the immune system. Its level increases gradually in blood after birth, reaches a plateau in the second decade, then declines. Normal IgE level in adults is considered as 100-150 KU/L (38). Serum IgE may increase in allergic disorders, parasitic infections, inflammatory conditions, malignancy and immune deficiency (199). Since IgE level may increase in various conditions other than AR, the sensitivity of serum total IgE level is low. It is employed to diagnose an allergic response in the body, not for the diagnosis of a specific allergic disorder. Total IgE level determination is not beneficial in the diagnosis of AR, however sometimes it may support the diagnosis when combined with other diagnostic tests (200).

### 6.4.2. Serum allergen-specific IgE

This is the most frequently used in vitro test in the diagnosis of AR. Allergen specific IgE determination in serum is not influenced by medications, or dermatological disorders. It is employed to screen allergy, and to monitor the effectiveness of immunotherapy. Allergen specific IgE levels in serum and SPT results are well correlated (38).

Specific IgE antibody test aims to detect the level of an allergen-specific IgE in serum. Allergen-specific IgE is produced by a type 1 hypersensitivity reaction in the body.

Phadebas radioallergosorbent test (RAST; Pharmacia, Uppsala, Sweden) is the first immunoassay test designed to determine allergen-specific IgE in serum. In this method, the patient’s serum is mixed with known allergens to let allergen-specific IgE in the serum bind these allergens, and produce allergen-antibody complexes. The test material is washed to remove excess, unbound IgE. Then, radiolabeled anti-IgE is added, they bind to patient’s IgE that made allergen-antibody complexes with the specific allergen. Measurement of radioactivity gives the amount of allergen-specific IgE in the serum (198).

Nowadays, enzyme-labeled anti-IgE has been used more frequently for immunosorbent analysis (ELISA). The enzymatic reaction produces a colorful product on adding the substrate for the enzyme. Fluorescent enzyme immunoassay (FEIA) and chemiluminescent immunoassay have been developed in parallel with the advances in ELISA technique, and they are now employed to detect allergen-specific IgE in the serum (198).

ImmunoCAP™ (Phadia/Thermo Fisher Scientific, Uppsala, Sweden) and Immulite™ (Siemens Healthcare Diagnostics, Los Angeles, CA, USA) are highly specific FDA-approved methods for quantitative measurement of allergen-specific IgE levels in serum (38, 201, 202). The results are provided quantitatively (between <0.10 and >100 kU/L), or on a 6-grade scale (1 IU/mL = 1 kU/L = 2.4 ng/mL) (198). A specific IgE level ≥ 0,35 kU/L is regarded as positive in quantitative tests (203, 204). The calibrators used for test methods must comply with the requirements set by the World Health Organization for human IgE (201).

Thanks to advances in genetics, pure natural, recombinant allergens (grass, pollen, mite, mold, etc.) or synthetic peptide panels can be produced for specific IgE tests (205-207). The use of pure allergens instead of raw extracts increased the sensitivity of specific IgE tests(38). However, these alternatives are not possible for all allergens, therefore the test material is enriched with recombinant allergens. This is called as ‘spiking’ (114).

Usually symptom severity is correlated with serum specific IgE levels. It was reported that wheezing and serum specific IgE levels were correlated, however any IgE cutoff level could not be clearly determined for wheezing. The authors concluded that the severity of the symptoms did not only depend on IgE antibodies, but also to the release of mediators and variable responses of the target organ to these mediators (208). On the other hand, serum specific IgE may be undetectable in a patient with allergic symptoms, or may be positive in an asymptomatic individual (209).

### 6.4.3. Determination of serum specific IgE with microarray method

Component-based diagnostic testing (CBDT) is a method based on microarray technology, where recombinant allergens are used. In this way, sensitization to allergens can be determined even in asymptomatic patients, using peptide sequences consisting of pure allergen molecules or allergen sequences. CBDT aims to clearly determine the antigenic epitopes to which the patient’s IgE is bound, and to establish a relationship between the IgE measured specific to the subcomponent of allergens and the severity of allergic disease (210). CBTD is a more sensitive test that has been found effective in identifying the allergen that causes the main symptom. It also reduces the problems caused by cross-reactions. Thanks to these features, it may ensure a more targeted immunotherapy (211, 212).

ImmunoCAP ISAC (Thermo Fisher Scientific, Phadia AB, Uppsala, Sweden) is a commercial example of CBDT. It allows detection of IgE against 112 individual allergens derived from 51 allergen sources (213).

### 6.4.4. Basophil activation test

Basophils and mast cells carry high-affinity IgE receptors on their surfaces which activate on exposure to sufficient amount of allergen. Various techniques have been developed to examine basophil responses to allergens. After the discovery of the activation marker CD63 on the basophils by Sainte-Laudy et al. (214), basophil activation test (BAT) has been used in the diagnosis of type 1 allergy. BAT allows in vitro analysis and quantification of activated basophils by flow cytometry (215, 216, 217).

### 6.4.5. Basophil, histamine and leukotriene release tests

These tests aim to determine histamine and leukotriene C4 (LTC4) released from the basophils after treating human blood with allergen. Since live cells are needed to perform the test, the blood sample should be analyzed within 24 hours. These tests are not used in common (218-220).

### 6.4.6. Nasal allergen-specific IgE

Some patients have classical AR symptoms in absence of systemic atopy, ie. negative skin tests and serum allergen-specific IgE. This has given rise to the concept of local allergic rhinitis (LAR). LAR is diagnosed in absence of a systemic atopy (negative skin tests and serum specific IgE), and positive nasal allergen-specific IgE or nasal provocation test (221).

Various techniques have been used for detection of nasal allergen-specific IgE including Swab method in which specific IgE is analyzed in 5 ml of isotonic nasal lavage fluid, and ImmunoCAP radioallergosorbent technique (UniCAP; Pharmacia, Uppsala, Sweden) (221).

**Keywords: **Allergic rhinitis, in vitro tests, nasal cytology, specific IgE, total IgE.

### 6.5 Other tests used in diagnosis of allergic rhinitis

### 6.5.1. Nasal provocation test

Nasal provocation test is based on appearance of symptoms such as nasal edema, congestion and sneezing, and alterations in objective measurements such as decreased nasal airflow following nasal application of an allergen or a non-specific irritant (222). Nasal provocation tests with specific allergens, histamine or methacholine are employed for the diagnosis of AR as well as for decision of stopping treatment (223, 224). In fact, nasal provocation tests intend to determine the amount of allergen which causes the clinical response in an AR patient (224, 225). Apart from this, nasal provocation test is an important method in determining the effectiveness and safety profile of the AR treatment, and change of the patient’s symptoms after treatment. It may also be used to determine effectiveness of specific allergen immunotherapy (225). Nasal provocation test is used for detection of responsible allergen in occupational AR (226). It is also used in order to determine efficacy of treatment.

It should be noted that there should be at least 7 days between provocation sessions. First, saline is administered into the nose as a control solution, then the provocation agent is applied (227). The most frequently used objective test in evaluating the response during nasal provocation test is rhinomanometry, which measures nasal airflow resistance (222, 223).

### 6.5.2. Nasal cytology

The inflammatory cells obtained by scraping inferior turbinate mucosa on anterior rhinoscopy is examined for eosinophils, and if more than 25% of them are eosinophils, this is supportive for AR. The quality of nasal cytology, thus its use in the diagnosis of AR depends on obtaining a satisfactory sample, as well as proper preparation, staining and interpretation by experienced physicians (228).

### 6.5.3. Visual analogue scoring

There are four parameters in this scoring system; namely VAS 1 (general allergy symptoms), VAS 2 (nasal symptoms), VAS 3 (ocular symptoms) and VAS 4 (asthma symptoms). These parameters provide more accurate diagnosis of AR patients, determine the severity of the disease, and used for making a more effective treatment plan (229).

### 6.5.4. Studies on mucociliary clearance

Chronic airway disorders, nasal infection, sinusitis, otitis media and their co-existence with AR is the main reasons for disturbance of nasal mucociliary clearance (230). Mucociliary clearance is a measurement of the elimination of inhaled or released aerosols, and saccharin test is the most frequently used method. One fourth of a saccharin tablet is placed over the anterior end of the inferior turbinate, and the patient is asked to sit calmly until he/she tastes it. Normal saccharin clearance time is 7-15 minutes, and over 20 minutes is abnormal (230).

### 6.5.5. Rhinomanometry and acoustic rhinometry

Rhinomanometry is a test that allows us to measure the transnasal pressure changes affecting the airflow through the nose (223). The pressure difference between the nostrils and the nasopharynx is measured. Acoustic rhinometry is an objective test for determining the structural abnormalities in the nasal airway. It measures the nasal cavity in cross-sections, and calculates the volume of the internal nasal cavity (231). It has high sensitivity and repeatability index. It particularly evaluates the anterior part of the nasal cavity, and allows identification of nasal geometric changes after nasal provocation tests (232).

### 6.5.6. Smell tests

They are done as psychophysical and electrophysiological tests. Smell sticks are used in psychophysical tests. This test has subtitles such as threshold, discrimination, identification, memory and hedonic scale (233).

### 6.5.7. Nasal nitric oxide measurement

Nitric oxide is the primary defense against microorganisms in the upper respiratory tract owing to its antiviral and bacteriostatic activities. It also has an accelerating effect on ciliary motion in the upper respiratory tract (234). In addition, increased total nitric oxide synthase and inducible nitric oxide synthase activity were detected in nasal biopsy and mucosal swab samples of patients with AR, viral rhinitis and chronic sinusitis (235).

### 6.5.8. Microarray tests

Along with the technological development, increased expression of RNA (lncRNA) and mRNA which do not encode in CD4 T cells has been detected in AR patients. These are measured using microarray tests (236).

### 6.6. Radiological imaging

### 6.6.1. Plain X-ray

Not indicated in the diagnosis of AR (237).

### 6.6.2. Computerized tomography

This is the primary imaging modality in the diagnosis of paranasal sinus disorders (238). On the other hand, its use in AR diagnosis is limited (239). It is particularly useful in the differential diagnosis of conditions that can be confused with AR, in the detection of acute and chronic rhinosinusitis, in the diagnosis of complications of rhinitis, in cases of rhinitis that do not respond to treatment, in cases of unilateral rhinitis, and in presence of nasal polyps in AR patients (240-243). In addition, paranasal sinus problems may occur in AR patients due to edematous nasal mucosa, impaired ciliary function, excessive secretion production and blockage of ostiomeatal complex. These patients must be evaluated with a CT scan (244).

### 6.6.3. Magnetic resonance imaging

Not preferred in the diagnosis of AR. It should be employed in the diagnosis of fungal sinusitis, encephaloceles and tumors (243, 245).

**Keywords: **Allergic rhinitis, diagnosis, radiology

### 6.7. Differential diagnosis of allergic rhinitis

**Method:** A literature search was done on Pubmed, Scopus and Google academic databases with the keywords “non-allergic rhinitis, differential diagnosis”. The meta-analyses were taken into account until 2015. All international studies were reviewed between 2015 and 2018. Older references were reviewed in case of insufficient new data on the topic.

### 6.7.1. Introduction

Rhinitis is defined as the inflammation of the nasal mucosa. A number of factors may trigger this inflammation including infectious agents, allergy, irritants, medications and hormones. Rhinitis is characterized by presence of one or more of the following symptoms: rhinorrhea, nasal congestion, nasal itching and sneezing (246). Headache, facial pain, otalgia, itching of throat and palate, need for frequent throat cleaning and sleep disturbances may also be evident in patients with rhinitis (247). Although rhinitis can sometimes be perceived as an insignificant disease, it is a serious disorder that should not be ignored since it causes significant morbidity including high medical treatment costs and impaired work performance (248). Chronic rhinitis is quite common, and can be encountered together with important comorbid diseases such as sinusitis, otitis and asthma (249). Chronic rhinitis includes a group of disorders with high direct and indirect costs, as the disease impairs quality of life and exacerbates comorbid conditions.

### 6.7.2. Pathophysiology

Some complex pathophysiologic mechanisms give rise to rhinitis symptoms (250). Chronic inflammation and neurogenic mechanisms play important role in the pathophysiology of rhinitis. AR is an IgE-mediated allergic reaction, and this reaction is accompanied by nasal inflammation varying in severity.

IgE does not play a role in the pathophysiology of non-allergic rhinitis (NAR). NAR occurs as a result of nasal hyperactivity against non-immunological stimuli; however, the exact cause of this hyperactivity has not been clearly understood. The blood supply and glandular secretions of the nasal mucosa are under the control of the autonomic nervous system. Stimulation of sympathetic nerves results in release of norepinephrine and neuropeptide Y, causing vasoconstriction of nasal vessels, decreasing mucosal blood flow (250-252). The parasympathetic nerves increase nasal secretions by stimulating the nasal glands through cholinergic neuropeptides and neurotransmitters. Autonomic dysregulation is manifested by decreased sympathetic activity and / or increased parasympathetic activity, and this is thought to play role in the pathophysiology of NAR (253-255).

Ophthalmic and maxillary branches of the trigeminal nerve are primarily responsible for the sensation of the nose, and nociceptive dysfunction is also thought to play a role in the pathophysiology of NAR (253, 254). The nerves that innervate the nose include fast myelinated and slow unmyelinated type C nerve fibers. Type C nerve fibers are the most sensitive fibers to pain and temperature changes, and are thought to play the primary role in NAR pathophysiology. These afferent nerve fibers secrete neuropeptides that increase vascular permeability and activate the submucosal glands on stimulation (251), resulting in rhinorrhea and sneezing.

### 6.7.3. Classification of rhinitis

Chronic rhinitis may be classified into allergic and non-allergic rhinitis. AR is nasal mucosal inflammation triggered by IgE release after exposure to the allergen. The definitive diagnosis is made by allergy tests; either a skin test or an allergen-specific IgE test in serum. In case of similar symptoms and examination findings but a negative allergy test, the patient is diagnosed with NAR. On the other hand, AR and NAR may coexist. Studies on patients with chronic rhinitis showed that 43% of them had AR alone, 23% had NAR alone, and 34% had mixed (allergic and non-allergic) rhinitis (256). These results indicate that a non-allergic component exists in more than half of the patients with chronic rhinitis.

Sometimes it may be difficult to distinguish AR from other types of rhinitis since the diagnostic criteria of different rhinitis types may overlap. The definitive diagnosis is very important since some treatment options that are effective in AR are less effective in other types of rhinitis. On the other hand, it should be kept in mind that NAR may progress to AR over time, and patients with NAR should be re-evaluated at intervals in terms of allergies (250, 257).

It is of great importance to distinguish other diseases that can cause rhinitis-like symptoms from rhinitis; these include anatomical abnormalities such as nasal septum deviation, systemic diseases such as hypothyroidism and diabetes, granulomatous diseases such as Wegener’s granulomatosis, as well as tumors, foreign bodies, cerebrospinal fluid rhinorrhea and nasal polyposis (100). These disorders will be discussed in detail in the relevant parts of this section.

### 6.7.3.1. Allergic rhinitis

AR is a symptomatic nasal disease triggered by IgE-mediated inflammation following allergen exposure. Allergic patients have a genetic tendency to produce an inflammatory response to materials that are normally harmless to the body. These reactions may also be called as “hypersensitivity reactions” since inflammation developing against such non-pathogenic substances (such as pollen) is unnecessary (138). The symptoms are characterized by itching, sneezing, watery rhinorrhea and nasal congestion. Atopy is the genetic predisposition to develop allergic hypersensitivity reactions. Atopic disease usually causes local inflammation in the region of exposure, and is classified accordingly. Examples are allergic conjunctivitis, AR, allergic asthma, atopic dermatitis and food allergies. Atopic individuals who suffer from one of these disorders tend to develop the others. AR is discussed in more detail in the relevant sections of this guideline.

### 6.7.3.2. Non-allergic rhinitis

NAR is diagnosed with the help of negative allergy tests in presence of symptoms similar to AR. It is often difficult to distinguish NAR from AR since both may exhibit similar clinical pictures. The patient has nasal congestion, rhinorrhea, sneezing and / or postnasal drainage, however skin test and / or allergen specific IgE in serum are negative. NAR covers a number of different conditions that cause similar nasal symptoms, therefore it a disease spectrum rather than a single disease.

NAR may be classified into eight groups (Table 6-1) (253, 258, 259). Mixed rhinitis, LAR, rhinosinusitis with or without nasal polyps, anatomical / mechanical abnormalities giving rise to chronic symptoms, and occupational rhinitis are not included in this classification; because these are not always non-allergic or may have different mechanisms other than that of NAR.

### 6.7.3.2.1. Non-allergic rhinopathy (vasomotor rhinitis, idiopathic rhinitis)

The most frequently encountered NAR type is ‘non-allergic rhinopathy’, previously called as ‘vasomotor rhinitis’ or ‘idiopathic rhinitis’. Although it was believed that inflammation triggered by intrinsic nasal vascularization and abnormalities in the nasal glands played role in the pathogenesis of vasomotor rhinitis, it has recently been shown that neurosensory mechanisms play an important role in the pathophysiology, therefore it is more appropriate to call it ‘rhinopathy’ rather than ‘rhinitis’. Since this group of patients do not have allergy by definition, this disorder is more accurately called as ‘non-allergic rhinopathy’ (253, 258, 259).

Non-allergic rhinopathy may occur episodically or perennially; the triggering factors are environmental factors that usually do not cause any symptoms in normal individuals. These factors include strong odors, temperature, humidity and pressure changes, exposure to cold air, alcohol consumption and hormonal changes during the menstrual period (30, 260). The patients may also admit with persistent complaints, without a triggering factor.

Non-allergic rhinopathy is a diagnosis of exclusion, and the patient history is of great importance. Absence of inflammatory cells including eosinophils, plasma cells and mast cells on nasal cytology helps diagnosis.

Unlike AR, patients are older and do not complain of symptoms triggered by exposure to classical allergens, such as pollen and house dust mites. However, as the patients’ complaints may be affected by changes in temperature and humidity, they may state that there are seasonal changes in their symptoms; and this should not be confused with seasonal AR.

In non-allergic rhinopathy, sneezing, nasal itching and ocular complaints are less common than AR. The differential diagnosis includes chronic sinusitis, nasal polyps, non-allergic rhinitis with eosinophilia syndrome, aspirin-exacerbated respiratory disease, infectious rhinitis/rhinosinusitis, anatomical abnormalities, medication-induced rhinitis, cerebrospinal fluid rhinorrhea and pregnancy. Intranasal corticosteroids or intranasal antihistamines are primarily used in treatment (260).

### 6.7.3.2.2. Non-allergic rhinitis with eosinophilia syndrome

Patients with non-allergic rhinitis with eosinophilic syndrome (NARES) present with AR-like symptoms such as nasal congestion, rhinorrhea and nasal itching; however allergy tests are negative. The diagnosis is based on patient history, physical examination, negative SPT or allergen-specific IgE in serum, and nasal eosinophilia on nasal cytology. The nasal turbinates are usually hypertrophic and pale on nasal examination. The risk of developing aspirin-exacerbated respiratory disease later in life is 50%, and 50% of them have bronchial hyper-reactivity (261, 262). These patients respond better to nasal corticosteroids compared to other patient groups with NAR.

### 6.7.3.2.3. Gustatory rhinitis

Gustatory rhinitis is characterized by watery rhinorrhea, which occurs immediately after the first bite of the meal. This occurs particularly with bitter and spicy foods. Gustatory rhinitis is more frequently seen in the elderly, and the vagus nerve is supposed to play role in its pathogenesis (259, 263). Anticholinergic agents administered before meals are usually sufficient for treatment (259, 263).

### 6.7.3.2.4. Medication induced rhinitis (rhinitis medicamentosa)

The term “rhinitis medicamentosa” is mostly used to describe the rebound nasal congestion caused by long-term use of topical alpha adrenergic decongestants / vasoconstrictor agents (such as oxymetazoline and phenylephrine). Use of these agents for 3 consecutive days is safe, however their use more than 5-7 days results in tachphylaxis and rebound nasal congestion. The rebound nasal congestion encourages patient to use the agent more, so that the patient goes into a vicious cycle and becomes addicted to these medications. Although the clear mechanism of the disease has not yet been fully understood, current theories focus on recurrent nasal hypoxia and negative neural feedback, and a reduction in the accompanying alpha-2 receptor response (260).

Medication induced rhinitis may also develop with use of some oral medications, including antihypertensives (such as beta blockers and angiotensin converting enzyme inhibitors), chlorpromazine, antidepressants, and phosphodiesterase-5 inhibitors used in the treatment of benign prostatic hypertrophy (30, 258, 262). Anti-inflammatory agents also increase leukotriene production, leading to rhinitis. They also may pave the way for asthma and bronchial hyper-reactivity.

The nasal mucosa of these patients tends to seem more erythematous and tends to bleed on nasal examination. The first step in the treatment is to stop the medication that causes rhinitis.

### 6.7.3.2.5. Hormonal rhinitis

Hormones act directly on the nasal mucosa, causing hyperactivity of the mucous glands and rhinorrhea. Hormonal disorders such as hypothyroidism and acromegaly, and changes in estrogen and progesterone levels may predispose to rhinitis (30, 262).

Pregnancy is another hormonal condition that prepares the ground for rhinitis symptoms. Pregnancy rhinitis starts in pregnancy, lasts 6 weeks or more, has no other underlying cause and regresses after birth. It has been supposed that increased circulating blood volume, ponding in vessels and relaxation in smooth muscles causes this condition (30, 264, 265).

### 6.7.3.2.6. Atrophic rhinitis

Atrophic rhinitis can be primary (idiopathic) or secondary. Primary atrophic rhinitis is characterized by atrophy of the nasal mucosa and secretory glands, and is usually seen in young adults. It is observed more frequently in developing countries and hot climates (30, 266). Patients present with bad smell, crusting and drying in the nose. Various bacteria (*Staphylococcus aureus, Proteus mirabilis, Escherichia coli)*, and particularly *Klebsiella ozaenae* may cause atrophic rhinitis. However, these microorganisms are also thought to infect the already damaged nasal mucosa secondarily (30, 266). Secondary atrophic rhinitis is most frequently observed after nasal surgery, in which excess nasal mucosa is removed. However, trauma, granulomatous diseases, chronic cocaine use and radiotherapy can also lead to the development of secondary atrophic rhinitis (30, 266).

In atrophic rhinitis, the functional, ciliated respiratory epithelium of nasal mucosa gradually transforms into non-functional ciliated squamous epithelium This leads to disturbance of mucociliary clearance and neurological regulation. Normal nasal airflow is disturbed, causing the sensation of nasal congestion. Although irrigation, moisturizing, topical or systemic antibiotics and various surgical techniques have been recommended for treatment, the results are not satisfactory (247, 259).

### 6.7.3.2.7. Senile rhinitis

Senile rhinitis is a type of rhinitis that is seen in the elderly, causing persistent watery nasal discharge. Patient’s complaints usually increase while eating and with some environmental factors. It can coexist with other types of rhinitis. Since the elderly individuals frequently use medications, medication induced rhinitis may accompany senile rhinitis, and medications may often be the main cause of the condition. This should be kept in mind in the differential diagnosis (30, 259).

### 6.7.3.2.8. Cerebrospinal fluid rhinorrhea

Patients with rhinorrhea should be questioned for craniofacial trauma and previous nasal surgery, and cerebrospinal fluid (CSF) rhinorrhea should be excluded in the differential diagnosis, particularly in case of unilateral rhinorrhea (262, 267). It should be kept in mind that increased intracranial pressure can also cause spontaneous CSF rhinorrhea in absence of any history of trauma or surgery.

### 6.7.4. Differential diagnosis of allergic and non-allergic rhinitis

Allergic and non-allergic rhinitis may present with similar clinical pictures. Sometimes it is not possible to distinguish these two disorders, and sometimes both allergic and non-allergic rhinitis coexist in same patient. This may cause difficulties in differential diagnosis. A number of studies have been conducted to show the similarities and differences of these two rhinitis types, and to increase the accuracy of the diagnoses. These two conditions may be differentiated by some features.

AR usually begins in childhood, and a family history of atopy (asthma, rhinitis, atopic dermatitis) is present; NAR usually occurs later in life, and does not show a familial transition. While NAR is more common in females, no gender predilection is observed in AR (253, 268).

While AR symptoms vary between seasons, this is minimal in NAR. Seasonal changes in NAR symptoms are often due to temperature and humidity changes rather than pollen; this should not be interpreted as seasonal AR (253, 268). AR is triggered by aeroallergens, and skin tests for aeroallergens and blood allergen-specific IgE tests are positive. NAR has many triggering factors such as irritants, and allergy tests are negative. In a study conducted by Di Lorenzo et al. (269) on 1511 patients, it was stated that there were some distinguishing features between allergic and non-allergic rhinitis. In this study, the authors stated that the number of nasal eosinophils was higher in AR, symptoms of sneezing and itching were more intense, recurrent conjunctivitis frequently accompanied rhinitis, and symptoms were more severe, while patients with NAR were older, most of them were females, more common symptoms were nasal congestion and rhinorrhea, and headache and smell disorders were more frequent.

Although carrying out a detailed physical examination is important, the physical examination findings do not contribute much to differential diagnosis. The nasal mucosa is usually pale, edematous and swollen in AR, however the nasal mucosa may be normal, erythematous or atrophic in NAR, and watery rhinorrhea may be observed. Dark circles under the eyes may be observed in AR (allergic shiners) (253, 268). In addition, it should be kept in mind that conjunctivitis is more frequent in patients with AR, and other allergic conditions such as asthma and atopic dermatitis may accompany (253).

**Keywords:** Non-allergic rhinitis, differential diagnosis.

### 6.8. Differential diagnosis of allergic rhinitis and comorbid disorders

**Method:** Pubmed, Science Direct, Google academic databases were searched using the keywords “nonallergic rhinitis, differential diagnosis, symptoms, triggers”. The international studies published between 2008 and 2017 were included in the review. Expert committee reports were taken into consideration for the topics with insufficient data.

### 6.8.1. Differential diagnosis of allergic rhinitis

### 6.8.1.1. Management of non-allergic rhinitis

### 6.8.1.1.1. History and symptoms

Non-allergic rhinitis (NAR) often appears when the patient comes across with non-allergic triggering factors. Examples of these triggering factors are temperature or humidity changes, nonspecific irritant stimuli such as alcohol, cigarette, smoke, powders, automotive emission fumes, chlorine and odors (eg. bleach, perfume or solvents). It is more common in females, and the elderly population has a higher rate of NAR compared to AR (270). Diagnosis is based on clinical history and exclusion of other types of rhinitis. If nasal symptoms (usually rhinorrhea, congestion, postnasal drip, headache, facial pressure sensation, throat cleansing and / or coughing) of the patient worsens or triggered by one or more of the previously known environmental factors, NAR should be considered. The accompanying ocular symptoms are minimal; nasal itching, palatal itching and sneezing are also uncommon. Some patients with NAR have persistent nasal symptoms and a possible causative factor cannot be identified. These patients may or may not have symptoms when exposed to environmental factors that trigger symptoms in other NAR patients. The clinical features of these patients (predominantly female gender, onset at adult age, clinical symptoms, and response to treatment) are similar to those with known triggers of NAR. It has been supposed that those patients have NAR. Unlike AR, NAR is usually an adult-onset disease, and the symptoms do not worsen when exposed to allergens such as pollens, house dust mite, or dog or cat feather. Seasonal symptoms associated with climate changes may be evident in the spring and fall, since NAR symptoms are triggered with changes in humidity, temperature and / or pressure. Therefore, seasonal NAR can be confused with seasonal AR. The diagnosis of NAR is based only on the patient’s symptom history and the symptoms that result from the triggering factors; however, the diagnosis of AR is made by confirmatory allergy tests that include positive skin test results or allergen-specific IgE test results in addition to patient’s history. These two diseases cannot be mutually excluded, and at least 60% of AR patients develop nasal symptoms with non-allergic environmental triggering factors. However, to have pure NAR, the patient’s skin prick test results or in vitro allergen-specific antibody tests must be negative (260).

Hormonal changes in thyroid dysfunction, adolescence, menstruation, menopause and pregnancy have been associated with NAR. Hormonal changes affect nasal homeostasis with disruption of the normal sympathetic / parasympathetic axis. Irritant-induced rhinitis reflects both the effect of chemical irritants and changes in temperature, humidity and pressure on occupational exposures. Cold air changes nasal homeostasis by increasing the release of triptase and other mast cell activation factors. Vasoconstriction or vasodilation in the nose to maintain constant heat and moisture in the nasal cavity may cause edema, obstruction and rhinorrhea in these patients. Exposure to irritant substances at work or at home may initiate an inflammatory response. The responses related to irritant factors may range from spicy food-induced gustatory rhinitis to inhalation of toluene or other industrial products in occupational rhinitis (271). Marked activation and disruption of the olfactory and trigeminal neural system alters nasal homeostasis on exposure to chemicals and some food. Activities like swimming and running may also cause rhinitis (272).

NARES is diagnosed with a significant level of eosinophilia in nasal cytology and negative allergy tests. The triggering factor of the disease is unknown. The patients with NARES are supposed to have an underlying chronic nasal inflammation that causes eosinophilia and rhinitis symptoms. Nasal polyposis and aspirin intolerance rates are high in these patients.

The systemic disorders that present with rhinitis symptoms should also be considered. Churg-Strauss syndrome, Graves’ disease, systemic lupus erythematosus and Wegener’s granulomatosis may be associated with NAR (271).

History is an important component in making the differential diagnosis. Nasal and palate itching, sneezing, symptoms on exposure to allergen, and seasonal symptoms including ocular ones support an allergic disease. Family history also gives an important clue for presence of allergy (273). Recurrent seasonal symptoms suggest allergic triggers such as pollens or mold spores. If symptoms appear at home, house dust mites should come to mind. Occupational allergens should come to mind if symptoms appear at work; for example, flour-sensitive bakers or animal allergy in an animal laboratory. In prolonged periods of absence of allergens (such as holidays), there may be a remission or the symptoms get milder.

Rhinorrhea may be anterior or posterior, may occur as a postnasal discharge, may be due to allergy or not. AR causes bilateral light-colored nasal secretion. Isolated, unilateral light-colored nasal discharge is not common, and CSF leakage should be excluded in this case. CSF leak most often occurs after sinus surgery or trauma, but may also be spontaneous.

Transparent nasal discharge may be associated with allergies. Eosinophils give secretion a yellow color whereas neutrophils give it a green color, and this reminds an infection (274). Nasal crusting may be seen in AR, but it is not frequent. If the primary complaint is nasal crusting and epistaxis, chronic rhinosinusitis, nose picking, Wegener’s granulomatosis, sarcoidosis, other vasculitides, atrophic rhinitis, treatment with noninvasive ventilation, cocaine use and frequent use of nasal decongestants should be considered. Crusting may also be observed in the early postoperative period of nose and sinus surgery. Intranasal corticosteroids may rarely cause epistaxis and crusting, particularly if the spray is not used in the correct manner (262, 274). Unilateral bloody nasal discharge may be related to a tumor, foreign body, nose picking or improper use of nasal sprays. If bilateral, granulomatous diseases, bleeding diatheses, incorrect use of nasal sprays, infections and nose picking should be considered (262).

Nasal congestion is usually bilateral in AR, however it may be unilateral in some frequent nasal abnormalities including nasal septum deviation (274). Structural problems such as septal deviation, septal perforation, cartilage atrophy and rhinophyma result in narrowing of the internal nasal valve, and cause a sensation of nasal fullness, congestion or obstruction (275). Non-allergic causes of nasal congestion such as foreign body, CSF leak, nasal polyps, tumors and infection should be excluded on physical examination (96).

Choanal atresia and encephalocele should also be considered in the differential diagnosis of nasal obstruction in young patients. Subjective nasal congestion is often observed in atrophic rhinitis (dry, wide nasal cavity), or following aggressive inferior turbinate surgery (empty nose syndrome).

A number of medications may cause symptoms of rhinitis. The mechanism of action of medications may be local inflammation, neurogenic action, or idiopathic (276). Neurogenic medications cause rhinitis by making an alteration in the parasympathetic-sympathetic axis, similar to hormones. Inflammatory agents act as chemical irritants that stimulate local inflammation. One form of this phenomenon is known as “rhinitis medicamentosa”, and is often associated with excessive use of nasal decongestants. Although much is not known on specific mechanisms of medication induced rhinitis, the medications of the patient should be carefully assessed for their timewise relationship with NAR symptoms (271). It is also important to question the effectiveness of previous rhinitis treatments, and question whether they were used only for acute symptoms or to prevent daily complaints (274).

**Keywords: **Non-allergic rhinitis, differential diagnosis, symptoms, triggers.

### 6.8.1.1.2. Physical examination

The examination starts with inspection; mouth breathing, frequent sniffing, nasal speech and scratching the nose may be observed. On inspection, clues of AR such as a horizontal line on the nasal dorsum, red and watery eyes supporting allergic conjuncivitis, and dark folds / shadows under eyes known as “allergic shiners” may be seen.

Wegener’s granulomatosis or chronic cocaine use may result in the saddle nose deformity. Nasal polyps may lead to enlargement of nasal framework. Sarcoidosis may cause a purple color on the nasal tip. Severe telangiectasia suggests hereditary hemorrhagic telangiectasia that manifests with epistaxis. Chronic mouth breathing may result from total or near-total nasal obstruction (262, 274).

Endoscopic examination is the preferred method for nasal examination. However, if an endoscope is not available, anterior rhinoscopy can be performed with a headlamp and nasal speculum. Nasal endoscopy is more specific than anterior rhinoscopy, and alters the diagnosis in more than 50% of patients with nasal complaints (274). In AR, the appearance of the nasal mucosa may be normal (particularly when a seasonal AR patient is examined out of the season), or transparent nasal discharge and edematous lower and middle turbinates may be seen. Polyps may be distinguished with their yellow / gray color, localization on the lateral nasal wall, and numbness on palpation. Yellow submucosal nodules with cobblestone appearance suggest sarcoidosis. Crusting and granulation occurs in vasculitides.

Nasal septal perforation may result from septum surgery, chronic vasoconstriction (due to chronic use of cocaine or topical decongestants), Wegener’s granulomatosis, nose picking, and rarely use of nasal corticosteroids (262, 274).

Although the majority of the patients with rhinitis are diagnosed either with AR or NAR, some other rhinitis types worth considering. Polyps, deviation of nasal septum, or traumatic bone fractures causing mechanical alteration in nasal laminar air flow may lead to rhinitis symptoms. Often, these can be identified on anterior rhinoscopy; however, the abnormalities causing occlusion at posterior or superior parts of the nasal cavity may be overlooked on anterior rhinoscopy, and require an endoscopic examination. More importantly, rhinitis may be related to a neoplasm and may cause mechanical obstruction or alter the dynamics in the nasal cavity. Lymphoma has been reported as the most common neoplastic cause of rhinitis (271).

Although nasal septum deviation is frequent, it rarely causes the main symptoms of rhinitis. It may cause unilateral symptoms and cause difficulty of treatment with nasal sprays. Otorhinolarngological examination should cover throat, postnasal region, palate and ears. All patients with rhinitis should have a chest examination including spirometry and peak flow to determine a possible coexisting asthma (274).

**Keywords: **Anterior rhinoscopy, nasal endoscopy.

### 6.8.1.1.3. Diagnostic methods

An underlying allergy is excluded with a negative SPT and/or negative allergen-specific IgE in serum, and the nasal symptoms are categorized in NAR. Infectious diseases, rhinosinusitis and mechanical /anatomical abnormalities are also excluded (30, 260).

SPT and allergen-specific IgE in serum are usually positive in AR. The diameters of induration and erythema are measured 15-20 minutes after pricking in SPT. If the diameter of the allergen induration is ≥ 3 mm or it equals to that of histamine (histamine ≥ 3 mm), the result is considered as positive for that allergen.

A number of method-related factors may affect serum allergen-specific IgE results. In particular, the anti-IgE used in the kit should preferably be a mixture of monoclonal antibodies that are specific to more than one epitope in the Fc fragment (277).

Complete blood count, nasal swab, nasal cytology, blood tests for a possible underlying condition (thyroid hormone, GH, CRP), b2--transferrin in nasal secretion (259), urinalysis for cocaine use, computerized tomography (CT), and magnetic resonance imaging (MRI) may be used in diagnosis (262). CT and MRI may be used to differentiate anatomical problems, however they are expensive. In fact, imaging methods may not correlate with functional obstructions. CT and MRI are expensive, however they may be used in the differential diagnosis to exclude anatomical abnormalities. However, the imaging modality findings may not correlate with functional nasal obstruction (273).

**Keywords:** Laboratory investigations, differential diagnosis, radiology.

### 6.8.1.1.4. Further medical workup

**Nasal inspiratory peak flow: **Nasal inspiratory peak flow rate is a fast and inexpensive test, the device is small, portable and practical, it can be used easily even in children. It can be used for objective measurements in nasal provocation tests. The results are correlated with rhinoscopic examination in rhinitis, but there are no symptom scores (274, 277).

**Anterior rhinomanometry: **Rhinomanometry provides information about the transnasal pressure affecting the nasal airflow by measuring the anterior and posterior nasal pressures. Measurement is made by placing a pressure detector in the anterior and posterior of the nose, or the postnasal region. In AR, rhinomanometry is used in studies investigating the pathogenesis, severity and treatment control. It objectively illustrates the nasal obstruction increasing in parallel with the duration and severity of AR (277).

**Acoustic rhinometry: **The acoustic rhinometry device is occasionally used in nasal provocation tests.

**Nasal provocation tests: **Nasal provocation test is based on appearance of symptoms such as nasal edema, congestion and sneezing, and objective measurements such as slowing of airflow rate after application of an allergen or a nonspecific irritant into the nose. In practice, the allergen is prepared by dilution. The patient’s basal symptoms are recorded before the test, and a baseline rhinomanometry is performed. First normal saline, then allergen in increasing concentrations are applied into the nose. The waiting period between each application is 15 minutes. The test is considered positive and terminated if the patient has symptoms, or the rhinomanometric value decreases by at least 20% (277).

**Nasal exhaled nitric oxide:** Nitric oxide is mainly synthesized in the paranasal sinuses in the upper respiratory tract, and is associated with inflammation of this region. Increased inducible nitric oxide synthase activity in patients with AR is attributed to persistent inflammation of the nasal mucosa. In case of high exhaled nitric oxide levels in adolescents with complaints of rhinitis, the probability of persistent rhinitis four years later is 5.11 times higher compared to those with low exhaled nitric oxide levels (277).

**Keywords: **Rhinomanometry, nasal provocation test, differential diagnosis.

### 6.8.1.1.5. Treatment

Avoiding triggering factors forms the basis of treatment. An alternative medication with fewer side effects should be considered in case of medication induced rhinitis. Nasal irrigation helps restoring normal physiology by providing mechanical cleansing and moisture into the nasal cavity. This treatment is also safe and effective in pregnant women (271, 278). Although intranasal corticosteroids are frequently used for NAR, only fluticasone and beclomethasone have the indication for treatment in patients 4 years and older (279). Azelastine nasal spray has proven to be effective in randomized controlled trials, and may be used in 12 years and older NAR patients (280). Surgery is considered for crusting, nasal airway obstruction, or rhinorrhea that does not respond conservative treatment (271).

**Keywords: **Treatment, non-allergic rhinitis.

### 6.8.1.2. Disorders other than non-allergic rhinitis in differential diagnosis of allergic rhinitis

**Rhinitis Associated with Systemic Diseases: **Various systemic disorders can cause rhinitis. Nasal manifestations are observed in some granulomatous disorders (Wegener’s granulomatosis, sarcoidosis, Churg-Strauss syndrome), autoimmune conditions (lupus erythematosus, Sjogren’s syndrome, pemphigus), cystic fibrosis, tuberculosis and ciliary dyskinesia. Other systemic causes should be considered in patients with symptoms that do not respond maximum medical therapy, or when scarring (pemphigus), intense bleeding and crusting (Wegener’s granulomatosis) or submucosal cobblestone appearance (sarcoidosis) are observed in the nasal mucosa. The findings of granulomatous disorders include persistent inflammation and crusting, ulceration, nasal mass formation or mucosal cobblestone appearance in addition to extranasal findings and systemic symptoms. Autoimmune diseases can cause antigen-antibody interaction in the nose, resulting in mucosal ulceration, dryness, crusting and recurrent infection. In cystic fibrosis, the sinonasal findings vary according to the mutation state. The nasal polyps are evident most frequently in patients with ΔF508 mutation, and are often together with bacterial biofilms of *Staphylococcuc aureus* or *Pseudomonas aeruginosa*. Tuberculosis often involves the nasopharynx, and results in nasal inflammation and rhinorrhea. Nasal symptoms occur in primary ciliary dyskinesia due to inability to clear mucus from nose and paranasal sinuses, and the diagnosis is made with the saccharin test (281).

**Other Conditions in the Differential Diagnosis of Allergic Rhinitis: **Anatomical disorders such as nasal valve narrowing, turbinate hypertrophy, nasal septum deviation, nasal tumors and foreign bodies should be considered in the differential diagnosis (259).

**Local allergic rhinitis (LAR): **It has recently regarded as a subgroup of rhinitis. The allergic reaction of LAR is limited to the nose. Although the local inflammatory response is similar to AR, there is no systemic involvement. Some patients classified as NAR previously are now considered to have LAR. LAR manifests only in the nose, and is characterized by a local inflammation with eosinophilic infiltration, and nasal IgE production in response to aeroallergens. Skin tests performed with aeroallergens and serum allergen-specific IgE levels are negative. LAR is associated with asthma and conjunctivitis, and often begins in childhood (273).

**Keywords: **Non-allergic rhinitis, local allergic rhinitis.

### 6.8.2. Comorbid diseases in allergic rhinitis

### 6.8.2.1. Asthma

AR and asthma are usually perceived as unrelated disorders, and they are diagnosed and treated separately by Otorhinolaryngologists and Chest Diseases physicians. Although different parts of the respiratory tract are affected in AR and asthma, their etiology and pathogenesis, and the pathological changes in the respiratory tract are similar. Therefore, evaluating and treating these two disorders together will make management of both diseases more successful.

There are a number of possible relationships between AR and asthma: (a) AR may be statistically related to asthma; (b) AR may aggravate concomitant asthma; and (c) AR may have a causal role in the pathogenesis of asthma.

The upper and lower airways share the same anatomical, functional, pathogenic, clinical and immunological features, and share the same lymphoid network, thus they activate similar cells when reacting to airborne allergens. These relationships have been extensively studied, and it has been suggested that allergic respiratory disease is a disease in continuum, occurring simultaneously. Several possible mechanisms have been proposed to explain the link between AR and asthma. Braunstahl has shown that an allergen encountered anywhere in the airway may cause a response throughout the airway (282). Failure to perform the functions of the nose such as air cleaning, heating and humidification will increase the reaction in the lower respiratory tract. In addition, allergens may stimulate nasobronchial reflex. The hypothesis of post-nasal discharge (inflammatory cytokines / transport of mediators from the nasopharynx into the lower respiratory tract), which was previously popular, has been largely abandoned (283).

Numerous studies have investigated the association of AR and asthma and their comorbid effects. While the prevalence of asthma is less than 2% in those without AR, its prevalence is 10-40% in those with AR, and it has been reported that 80% of asthma patients may have symptomatic AR (284). Kou et al. (285) performed a meta-analysis, and reported estimated comorbidity of asthma as 35.01%, and the estimated comorbidity of AR as 54.93% in Chinese children with asthma.

Although different rates have been reported in studies for the association of AR and asthma, it is clear that they are comorbid conditions. Recent studies have revealed that environmental factors play important roles in the etiology of AR and the comorbidity of allergic diseases. The prevalence of asthma and AR may also change depending on smoking, exposure to cigarette smoke and air pollution as well as the distribution of allergens that vary in accordance with the geographical features, climate and vegetation (286). The coexistence of AR and asthma differs in childhood and adulthood. Di Cara et al. showed that children with moderate-to-severe persistent AR developed new-onset asthma in the 5-year follow-up, on the other hand, one-third of children with mild AR developed asthma. Therefore, the notion that persistent AR may be related to the progression into asthma has strengthened (287). AR in childhood is not only associated with a predisposition to development of asthma in childhood, but also with an increased risk of asthma in adulthood (96).

Both AR and asthma are chronic inflammatory diseases of the upper and lower respiratory tracts, and similar inflammatory mechanisms and similar cells and mediators play a role in their pathogenesis. The studies clearly showed that the upper and lower airways share common immunopathological mechanisms, and as a result, the term “single airway” or “combined airways” has been used. This term has been based on the evidence that AR is an independent risk factor for the development of asthma (288).

The respiratory mucosa consists of pseudostratified ciliary columnar epithelium and a supporting lamina propria. The airway epithelium acts as a physical barrier against the external environment, is constantly exposed to pollutants, allergens and microbes, and responds directly by regulating adaptive immune responses (289). The lower airway mucosa is the same as that of the upper airway, except for the presence of the airway smooth muscle that extends from the trachea to the terminal bronchioles. The airway mucosa is resistant to environmental factors, and quickly initiates tissue repair after damage. Airway damage causes inflammation, and inflammatory and structural cells release cytotoxic mediators, free oxygen radicals and collagenases. As a result, epithelial cells secrete adhesion molecules, cytokines, and growth factors to induce tissue repair. In healthy individuals, inflammation settlement and tissue restoration are provided at the end of this response.

Tissue remodeling can occur in any organ in response to inflammation or mechanical injury, in order to restore normal tissue. Airway remodeling is considered as the hallmark of asthmatic lung, and is often associated with more severe phenotypes (290). Today, most of our understanding on airway remodeling has been obtained from allergic asthma studies. Structural changes of the lower respiratory tract in asthma include epithelial shedding, goblet cell hyperplasia, basement membrane thickening, mucous gland hypertrophy, subepithelial fibrosis, and angiogenesis. Despite extensive research over the past decade, the precise mechanisms underlying the different aspects of lower respiratory tract remodeling and their clinical effects for allergic diseases are still unclear (288).

AR is mainly driven by Th2 cell-related inflammation. Upper and lower airway samples obtained with bronchoscopy in patients with AR and allergic asthma showed similar Th2 cell-induced inflammation in the nasal and bronchial mucosa (291). Considering that inflammation causes remodeling, permanent structural changes should be the key findings in AR, as in asthma. However, there is contradictory evidence for remodeling in AR. Allergen exposure in individuals with AR leads to rapid activation and proliferation of inflammatory pathways, almost identical to those occurring in asthma. Lim et al. showed that basal membrane thickness increased in the initial nasal biopsies taken after allergen exposure in individuals with AR, however there was no further increase in basement membrane thickness 24 hours after allergen exposure, despite eosinophilic chemotaxis. No change was observed in epithelial thickness or submucosal collagen deposition (292). Eifan et al. (293) observed an increase in submucosal eosinophils, however no significant difference was observed in terms of angiogenesis, lymphangiogenesis, extracellular matrix accumulation, collagen markers, reticular basement membrane thickness or percent of glandular area when compared to normal individuals. Contrary to aforementioned studies, Amin et al. (294) reported the loss of epithelial integrity proportional to the degree of eosinophilia in perennial AR. Comparing whether patients with asthma and AR have more nasal remodeling compared to the ones that have AR alone will provide important information. In addition, variables such as the severity of the disease, allergen type and amount should be taken into account.

A study that analyzed the molecular mechanisms underlying a multi-comorbidity of asthma, eczema, and rhinitis identified a series of proteins and cellular processes that are common in these atopic disorders. In this study, it was observed that asthma and rhinitis shared numerous interrelated proteins. The authors reported that there were 15 pathways including IL4 and GATA3-related pathway in the multi-comorbidity of asthma, eczema, and rhinitis, and a number of proteins were obtained potentially related to this multi-comorbidity processes (295).

The presence of AR causes aggravation of asthma attacks, and lengthens hospital stay in asthmatics. It has been shown that treating AR causes a reduction in asthma costs and hospitalization. ARIA Guideline suggest that asthma patients should be evaluated for AR, and AR patients for asthma (38). Early and aggressive treatment of AR may prevent development of asthma. Therefore, the physicians treating AR should be familiar with the early signs and management of asthma. When evaluating a patient with AR, asthma should also be evaluated, and typical symptoms such as difficulty of breathing, cough, wheezing, and ability to exercise should be analyzed, and the patient should be consulted to a chest diseases physician, if necessary.

AR therapy should be individualized in patients with concomitant asthma. Administration of oral antihistamines and particularly nasal steroids have been shown to reduce bronchial hyper-reactivity and improve asthma control. Lohia et al. (296) conducted a meta-analysis and reported that nasal corticosteroids significantly improved the morning and evening peak expiratory flows. Nasal corticosteroids have been shown to significantly improve asthma-specific outcome measures in both AR and asthma patients. It was determined that oral corticosteroids were not superior to inhaled corticosteroids, and the corticosteroid effect was more pronounced with nasal corticosteroid sprays sniffed into the lungs through the nose.

Although leukotriene receptor antagonists are not used in the primary care of the patients with AR alone, it may be a viable choice in case of simultaneous AR and asthma (297). To date, the only treatment option that eliminates respiratory symptoms as well as allergic / immunological mechanisms at the background is allergen specific immunotherapy. It can change the prognosis of allergic conditions, especially AR, by targeting the underlying etiology; hence, it may be a valuable first-line treatment strategy to prevent asthma. There is evidence that the treatment with immunotherapy may prevent development of asthma and susceptibility to new allergens children with AR (96, 298). Follow up of 205 children treated with immunotherapy up to 10 years after treatment revealed improvement in AR symptoms that continued after end of immunotherapy, and fewer children had asthma in the group treated with immunotherapy (299). Aydıner et al. (300) followed up monosensitized patients with mild persistent asthma with/without rhinitis for subjective and objective asthma and AR parameters for 3 years. Three years later, there was a significant reduction in asthma symptoms in the groups treated with immunotherapy, together with a marked improvement in rhinitis symptoms. In their review, Morjaria et al. (301) concluded that, unlike corticosteroids and other symptomatic treatments, immunotherapy prevented development of other allergic conditions in individuals at risk.

**Keywords:** Allergic rhinitis, asthma.

### 6.8.2.2. Rhinosinusitis

### 6.8.2.2.1. Nasal polyps

As defined in the European Position Paper on Rhinosinusitis and Nasal Polyps (EPOS 2020), rhinosinusitis (RS) is inflammation of the nose and the paranasal sinuses characterized by two or more symptoms, one of which should be either nasal blockage/obstruction/congestion or nasal discharge (anterior/posterior nasal drip), and/or facial pain/pressure and/or reduction or loss of smell. On endoscopy, nasal polyps (NP) and/or mucopurulent discharge mainly from middle meatus and/or edema/mucosal obstruction are evident. On CT, there are mucosal changes in the ostiomeatal complex and/or sinuses. The definition of acute rhinosinusitis (ARS) in children is similar to aforementioned definition, but there is symptom of “cough” instead of “a decrease or loss in smell”. These findings are defined as ARS if they are present for less than 12 weeks, and as chronic rhinosinusitis (CRS) if they are present for a longer period. CRS, on the other hand, is divided into two groups as “with nasal polyps” and “without nasal polyps” (302).

The association of AR and RS has been the subject of many studies. Although the coexistence of AR and RS has been shown in most of the studies, there is no clear definition whether they are risk factors for each other. The results of the studies are also contradictory. Although some studies report that patients with sinusitis have a higher prevalence of AR with a positive prick test compared to the general population, others contradict this finding. Most of the discussions on this issue are due to the fact that most of the studies are old, and that there were no definitions of CRS and AR at that time. The patients included in the study were not classified as ARS, CRS with polyps or CRS without polyps, as stated in EPOS. On the other hand, susceptibility to CRS may differ with regard to the type of AR, since it has been reported that patients with perennial AR were more susceptible to CRS compared to the ones with seasonal AR (303).

The connection between AR and RS may be explained by various mechanisms. One of these is the blockage of the ostiomeatal complex due to mucosal edema caused by inflammation induced by IgE-mediated mechanism of AR, prevention of mucociliary transport of the sinuses, and development of bacterial colonization thereon. Another mechanism is significant eosinophilic inflammation, particularly in the maxillary sinus, in allergic patients during the allergen season. When the ethmoid and nasal polyp tissues of patients with CRS are examined, local T cell infiltration, mediators such as IL-4 and IL-5, and Th2-type cytokine profile dominance were observed in the ethmoid mucosa and nasal polyps of the patients with CRS. These cytokines stimulate local IgE production, cause regional eosinophil infiltration, and prolong eosinophil life. Nasal allergen provocation or natural allergen exposure causes eosinophil migration into the paranasal sinuses (283).

The prevalence studies showing the coexistence of AR and RS reported different rates. İbanez et al. (304) included 1275 AR children in their 217-centered study, and reported rhinosinusitis in 26.1% of them. Hoffman et al. conducted a GA2LEN (The Global Allergy and Asthma European Network) survey on 8347 patients on phone. Among all, 29% of the participants had AR criteria, 18% had ARS criteria, and 16% had CRS criteria. The authors investigated a number of risk factors, and emphasized that presence of AR, ARS and CRS symptoms constituted risk factors for each other, and that these three disorders had common and independent risk factors (305). Ha Yoo et al. (306) studied the cost effectiveness of airway disease in Korea on 999 patients who admitted with respiratory complaints, and reported that AR and RS coexisted in 15.4% of the patients. RS was not classified as acute or chronic in those studies. However, considering the mechanisms mentioned above, AR prevalence with ARS, chronic rhinosinusitis without nasal polyps (CRS w/o NP) and chronic rhinosinusitis with nasal polyps (CRS w/ NP) are different (283).

### 6.8.2.2.2. Relation of acute rhinosinusitis and allergic rhinitis

Inflammation in AR may disturb mucociliary motility, and make the patient prone to ARS. The results of the prevalence studies on simultaneous appearance of AR and ARS are quite diverse. In 1992, Furukawa concluded that allergy was an important factor in sinusitis based on the analysis of AR and ARS studies and a number of summaries (307). Mbarek et al. (308), studied 100 children who admitted with recurrent upper respiratory tract infection and 164 healthy individuals, and reported that there was a significant relationship between allergy and RS. Naclerio et al. (309) and Blair et al. (310) also reported a clinical relation of ARS with AR. Lin et al. (311) followed 69 children between the ages of 3-12 for 1.5 years, and reported that 27 children with AR (39.1%) were more likely to develop ARS compared to nonatopic children. There is controversy on whether the frequency of ARS increases during the allergy seasons in patients with AR. Leo et al. compared 242 children with grass allergy with the control group of 65 in the period from April to June in terms of symptoms and findings of ARS. In the AR group, 17 (7%) children had endoscopically shown ARS, however 3 children (4.6%) in the control group presented ARS findings, and the authors concluded that there was no significant difference between two groups in terms of ARS in the pollen season. The authors stated that grass pollen AR was an insignificant risk factor for ARS (312). In their review of RS, Pant et al. stated that there was insufficient evidence indicating seasonal or perennial AR as a significant predisposing factor for ARS (313).

Melvin et al. (314) investigated the mechanism underlying relationship of AR and ARS. They studied toll-like receptor 9 (TLR9) expression in the nasal epithelia of the patients with AR and / or recurrent ARS. They showed that TLR9 expression was higher in the patients with AR and recurrent ARS. They reported that a congenital disorder in immune gene expression may lead to recurrent ARS in some patients with AR. Vlastos et al. (315) made saccharin test on 125 patients with AR, and showed that 23 patients with AR who had a predisposition to sinusitis had longer mucociliary clearance times compared to 102 AR patients without predisposition to sinusitis.

In the light of all these contradictory findings, one may say that although AR is not considered as a definite risk factor for ARS, it is beneficial to keep AR in mind, to perform tests for the diagnosis of AR in case of clinical necessity, and to add AR treatment in case of sinusitis, particularly in pediatric patients with recurrent ARS findings.

### 6.8.2.2.3. Relation of allergic rhinitis with chronic rhinosinusitis without nasal polyps

Studies on CRS w/o NP showed TNF-alpha, IL-4, IL-5 and IL-8 as mediators. The etiopathogenesis of CRS w/o NP is multifactorial. There are no controlled studies showing the relation of CRS w/o NP with AR. Prevalence studies reported AR prevalence in CRS patients in a wide range, varying between 36.2% and 84%. The patient populations are heterogeneous in most of the studies. The patients with and without NP, and even patients with fungal sinusitis were included in the same study. The reason for widely varying prevalences is the lack of clear distinctions, as stated in EPOS. There are only 3 cross-sectional studies that compared allergic and non-allergic patients with a group of patients with CRS w/o NP, carrying the current diagnostic symptoms for at least 12 weeks. Kirtsreesakul and Ruttanaphol divided the patients that were symptomatic for at least 3 months into two groups by performing a prick test, and compared their plain sinus radiographs and nasal endoscopic examinations. Although they did not find a significant difference between the two groups for endoscopic findings, they detected 2.8 times more abnormalities in the allergic patients compared to non-allergic ones (316). In 1999 Berettini et al. (239) and Ramadan et al. (317) compared allergic and non-allergic patients, and reported more radiological abnormalities in the allergic groups. Contrary to these reports, some studies reported that there was no increase in the incidence of CRS during pollination periods in patients with pollen allergy (302). Gelincik et al. (318) studied 155 patients with persistent rhinitis, and reported that CRS symptom scores and global CRS scores were higher in NAR patients compared to those with AR, and only the rate of nasal purulence observed in nasal endoscopy was high in AR patients. In a 2009 review, Pant et al. (313) stated that there was contradictory evidence for higher prevalence of IgE-mediated allergy in patients with CRS when compared to the ones without CRS, therefore there was no evidence to regard allergy as a direct risk factor for CRS.

In a study by Sedaghat et al. (319) in 2013, it was shown that the degree of atopy in children (such as the number of aeroallergen hypersensitivity or the presence of atopic multi-morbidities) was not associated with progression to CRS. Baroody et al (320) conducted a double-blind, randomized, placebo-controlled study on 20 allergic patients out of the allergy season. Nasal provocation was performed with allergens in one group, and with ringer lactate in the other group. Examination of maxillary sinus lavage fluid revealed that maxillary sinus inflammation was significantly more in the group that had nasal provocation with allergens.

DeYoung et al. (321) conducted a systematic review to analyze the effect of immunotherapy on the clinical findings of CRS. They stated that none of the studies were randomized-controlled trials, and the patients were not divided into CRS w/ NP or CRS w/o NP groups. In two studies, the symptom scores of atopic CRS patients treated with immunotherapy and those who received pharmacotherapy were compared. Both studies showed that symptom scores improved significantly in the immunotherapy groups.

The presence of AR (as determined by a positive RAST or skin test) in CRS patients does not affect the severity of the disease, degree of involvement of sinuses on CT scan, or the possibility of surgical failure when compared to non-allergic CRS patients. Therefore, the effect of AR on CRS is variable, but small. However, patients with CRS should be questioned about the symptoms of AR, and allergy should be tested in case of clinical suspicion. Regarding treatment, it is recommended that anti-allergic therapy be added to the treatment of patients with chronic sinus disease and associated allergies (302).

### 6.8.2.2.4. Relation of allergic rhinitis with chronic rhinosinusitis with nasal polyps

Similar to AR, Th2 pathway is active in CRP w/ NP, and the disease is characterized by high IL-5, IL-13 and IgE levels, and eosinophilic inflammation. Local mucosal IgE production and an increase in serum IgE levels are frequently observed in patients with NP. It has been suggested that when sensitive patients are constantly exposed to inhaled allergens, the polyclonal IgE antibodies contribute to persistent inflammation in CRS w/ NP. Pathophysiologically, allergy and CRS w/ NP overlap (303). However, it has been shown that the level of IgE was independent of the patient’s atopic state that in NPs, whereas the specific level of IgE in the NPs is partially correlated with the positivity of the skin prick test (283). CRS w/ NP can be seen with asthma, and this group of patients is particularly characterized by tissue eosinophilia and high local IgE levels (322). It has been reported that the perennial allergy prevalence is higher in patients with CRS w/ NP, and the AR prevalence varies between 45% and 77.4% (303). In 1999, Pumhirun et al. (323) found positive prick skin tests in 24 (60%) of 40 patients with NP, and in 6 (20%) of 30 control cases. They stated that allergic individuals have 6 times more risk for polyp formation compared to non-allergic individuals (Odd ratio = 6.0). On contrary to this high rate, Settipane and Chafee reported the prevalence of NP as 4.2% among 4986 individuals, as 6.7% in asthmatic patients, and as 2.2% in patients with rhinitis alone. Among 211 NP cases, 71% had asthma and 29% had rhinitis alone (324). Pang et al. (325) reported that food allergy diagnosed with the intradermal test was higher in NP patients (81%) compared to the control group (11%).

Tan et al. (326) performed surgery to NP patients unresponsive to medical therapy, and reported that skin tests were positive in more patents in CRS w/ NP compared to patients with CRS w/o NP. Erbek et al. (327) analyzed allergic and non-allergic patients with CRS w/ NP, and found total serum eosinophil and IgE levels significantly higher in allergic ones. They also reported that neither IgE nor eosinophil levels were correlated with other parameters of disease severity. Görgülü et al. (328) found allergy prevalence as 25% in CRS w/ NP patients, and as 28% in the control group, and stated that allergy was not a significant risk factor for NP as shown in their regression model.

Despite conflicting reports in the literature, the prevalence of allergy is higher in patients with CRS w/ NP compared to the general population and patients with CRS w/o NP. Th2 type inflammation, eosinophilia and increased IgE constitute the major underlying pathophysiology, which resembles to AR pathophysiology. However, clinical evidence supporting the relationship between CRS w/ NP and AR is quite weak.

**Keywords:** Allergic rhinitis, sinusitis, rhinitis.

### 6.8.2.3. Conjunctivitis

Allergic conjunctivitis is a hypersensitivity reaction affecting the eyelids, conjunctiva and / or cornea, causing itching, stinging, redness, edema and watering in the eye (329, 330). Atopic eye disorders include seasonal allergic conjunctivitis, perennial allergic conjunctivitis, vernal keratoconjunctivitis, atopic keratoconjunctivitis and giant papillary conjunctivitis (331). Allergic conjunctivitis accounts for more than 95% of them (332, 333). Papillary conjunctivitis is also frequently seen, and there is evidence of type 1 IgE-mediated hypersensitivity in all aforementioned disorders, except for giant papillary conjunctivitis (331).

Simultaneous AR and allergic conjunctivitis is called as allergic rhinoconjunctivitis (329). AR and conjunctivitis usually coexist. This situation was explained by nasoconjunctival reflex and a pathophysiological type 1 reaction in the nose and eye. Allergic conjunctivitis is the typical conjunctival reaction that occurs after allergen exposure. It affects 15-20% of the population. More than 75% of rhinitis cases with pollen allergy also have conjunctival symptoms (332). Patients with AR should be questioned for allergic conjunctivitis. The most important symptoms are itching, redness and swelling of the eyelids. Conjunctival hyperemia, edema and papillary reaction are evident on physical examination.

Vernal keratoconjunctivitis usually affects children and young adults. It is most frequently observed in temperate and subtropic regions, but may be seen all over the world. Although approximately 50% of the cases have AR, asthma, and atopic dermatitis, any relationship with atopy has not been shown (334). In addition to eye itching, redness, swelling and discharge, the vast majority of patients are photophobic. Giant papillae, seen as ‘paving stone’ in the upper tarsal conjunctiva, are the most characteristic finding. Sticky mucus is seen around giant papillae. The cornea may be affected, and punctate keratitis, which tends to merge in the central cornea, may be seen. There may be conjunctival scarring, and small white spots called ‘Tarantas’ spots may be seen in the upper limbus (335).

Atopic keratoconjunctivitis is associated with eczematous lesions of the eyelids and skin. There is dermatitis on the eyelids, face and trunk. Mild to severe chemosis may be seen. There may be giant papillae and conjunctival scarring, and Tarantas spots can be seen, similar to vernal keratoconjunctivitis. Atopic cataract may also develop (332).

Giant papillary conjunctivitis is not a true allergic disorder; irritation is the main etiologic factor. It is characterized by giant, medium or small papillae in the upper palpebral conjunctiva. Conjunctival appearance resembles vernal keratoconjunctivitis (336). However, there are no corneal lesions. The pathophysiologic mechanism is not allergy but irritants such as contact lenses, ocular prostheses, limbus sutures or dermoids. The allergy rate is not different from the normal population. The disease improves when the irritants are eliminated (332, 336).

**Keywords: **Allergic rhinitis, allergic conjunctivitis.

### 6.8.2.4. Otitis media

Inflammation is the main incident in comorbid diseases associated with AR. AR may coexist with acute sinusitis, acute otitis media, serous otitis media and adenoid hypertrophy (337).

Otitis media is the inflammation of the middle ear cavity. It is the most common disease in childhood following viral upper respiratory infections. Acute otitis media is an infection in which acute signs of infection such as fever and pain occur. On the other hand, otitis media with effusion (OME) is a non-infectious inflammation usually accompanied by Eustachian tube dysfunction, without acute signs of infection, defined by the accumulation of serous fluid in the middle ear. It often causes hearing loss in children.

The correlation of EOM with allergic disorders is still controversial. It has been reported that 24-89% of the children with OME had AR (338). The most important risk factors for EOM are young age, male gender, bottle feeding, passive smoking, allergy, low socioeconomic status, nursery care, winter season, genetic predisposition, immunity, ciliary disorders and craniofacial anomalies. Viral and bacterial infections, Eustachian tube dysfunction, allergy and mucociliary disorders play a role in the etiopathogenesis. Conditions that cause nasal obstruction such as allergy, infection, inflammation and adenoid hypertrophy can cause anatomical or functional impairment of the Eustachian tube, and result in collection of fluid in the middle ear cavity. The Eustachian tube is wide, short and horizontally located in infants, however it gets narrower, longer and becomes more oblique as the child grows up (339).

Examination of the middle ear fluids of AR patients and non-atopic controls collected while inserting tympanostomy tubes revealed significantly higher numbers of eosinophils, T lymphocytes and IL-4 and IL-5 positive cells in children with AR who had at least one allergen positivity in the skin test. T2 cytokines were also shown in torus tubarius and adenoid samples of atopic children with OME. However, IFN-gamma-positive cells were prominent in non-atopic patients with OME (340-342).

In case of positive history and AR symptoms, it is recommended to administer anti-allergy treatment in patients with OME and AR (343). Allergy treatment, pharmacotherapy and surgery are the treatment options for these patients. Treatment of allergy includes allergen avoidance, pharmacotherapy and immunotherapy. Avoidance measures include avoidance of allergens found positive in allergy tests. Decongestants, antihistamines, cromolyn sodium, oral and nasal steroids are among the options for pharmacotherapy. Oral steroids can be used for a short time, such as 7-14 days, however their use is limited due to their potential adverse effects, particularly in children. Therefore, topical nasal steroids are preferred. Immunotherapy may be an option in patients with AR and EOM, resistant to medical therapy. The effectiveness of sublingual immunotherapy is still under investigation. Surgical treatment is an option in cases unresponsive to aforementioned treatment modalities (337, 339).

**Keywords:** Allergic rhinitis, otitis media with effusion.

### 6.8.2.5. Gastroesophageal and laryngopharyngeal reflux

Gastroesophageal reflux (GER) is a chronic disorder characterized by the reflux of stomach contents into the esophagus. Patients with AR frequently complain of chest pain and GER (344). GER may cause symptoms such as anorexia, weight loss, dysphagia, wheezing, cough and hoarseness. One of the proposed mechanisms for increased prevalence of GER in AR patients is increased negative intrathoracic pressure due to AR. Although it has been suggested that the reason for increased negative intrathoracic pressure is the inspiration effort through a congested nose, there is no sufficient data to prove that (345).

Eosinophilic esophagitis should be considered in differential diagnosis in patients with resistant GER symptoms. Studies support that GER and eosinophilic esophagitis may be different clinical presentations of the same disease (346).

The coexistence of allergic disorders such as eczema, asthma and AR, and the importance of an allergic background have been emphasized in patients with GER and eosinophilic esophagitis. Eosinophils are thought to migrate to the esophagus in response to digested and inhaled allergens in eosinophilic esophagitis (347, 348). The esophagitis appears in spring or summer in adults and children with grass pollen allergy, therefore it is seasonal. However, food allergy is more prominent in children; the prevalence of cow’s milk allergy has been reported 18 times more than other food allergies (346). In eosinophilic esophagitis, cough accompanies refusal of food intake (349). Delayed type food hypersensitivity should be considered in patients who diagnosed with food elimination and have negative laboratory tests for Type 1 immune response. Patch test, food-specific IgE and skin test may be performed (350).

Laryngopharyngeal reflux (LPR) should be investigated in patients with cough and hoarseness.

Gastrointestinal symptoms of classical reflux may not be seen in LPR. Endoscopic findings of reflux esophagitis are not evident in 50% of the patients, and the severity of esophagitis and LPR may not be equivalent since the upper airway epithelium is more sensitive to the effects of gastric acid than the esophageal epithelium. Patients may experience postnasal discharge, chronic cough, irritation and need for cleansing the throat, as well as other findings such as chronic sinusitis and otitis (351). It was reported that 88% of chronic rhinosinusitis patients who underwent endoscopic sinus surgery had LPR diagnosed with double channel 24-hour pH monitoring, while this rate was 50% in the controls without sinusitis (352).

Post-infectious, allergic and nonspecific factors cause chronic upper airway inflammation in patients with chronic rhinosinusitis findings including chronic cough, rhinorrhea and nasal obstruction. AR is the most common cause in patients with symptoms of chronic rhinosinusitis, such as cough, sneezing, nasal congestion and discharge. GER should be kept in mind in the differential diagnosis particularly in cases that do not respond anti-allergic treatment, and antireflux therapy should be added to AR treatment.

**Keywords:** Allergic rhinitis, gastroesophageal reflux, laryngopharyngeal reflux.

### 6.8.2.6. Adenoid hypertrophy

Nasal congestion, mouth breathing and snoring are frequent both in patients with adenoid hypertrophy (AH) and AR. AR and AH symptoms overlap particularly in childhood. The relationship between AR and AH has not been yet clearly revealed despite numerous investigations. Since epidemiological studies have been conducted on patients in different age groups, their results are contradictory. The volume of adenoid tissue increases with age starting from birth, and reaches a maximum between the ages of 5-6 years. Then, its size gradually decreases until the age of 8-9, and adenoids are rarely seen in the adults. The larger series on the relation of allergy and AH have been conducted by Evcimik et al. (253). AH was reported in 12.4% of 1222 children with AR while it was seen in only 3% of 100 non-allergic children. The allergic children were divided into two groups with regard to presence of AH, and it was reported that rates of passive smoking and AR were significantly higher in the AH group. Ibanez et al. (304) studied on 1275 children between the ages of 6 and 12 years with AR in 271 centers, and reported AH prevalence as 17.3%. Sait et al. (354) performed a cross-sectional study on 190 patients with AR aged between 5 and 56 years. They reported AH in 88 (40.5%) patients, and noted that most of them were preschool children. Marino-Sanchez et al. (355) performed a non-randomized study on 150 children and adults with AR. The patients were divided into two groups as the responders and non-responders to pharmacotherapy. All patients with AH were in the non-responders group, however there was no significant difference between the groups for the size of the adenoids. The AH prevalence was the smallest in the group older than 12 years of age. On the other hand, the same authors performed otohinolaryngological examination on 130 patients with AR, and investigated the effects of the factors that caused nasal obstruction on the non-responsiveness to treatment. They reported that all abnormalities causing nasal obstruction (nasal septal deviation and turbinate hypertrophy), except for AH, were resistant to medical treatment of AR. They found a decrease in AR severity with medical therapy in patients with AH (356).

In the light of aforementioned findings, one may consider that AR may be important for AH in some age groups, or AH may coexist with AR. However, it is not clear whether AR causes AH, or that AH triggers AR symptoms. Doğru et al. found AH in 118 (21.2%) children among 566 children with AR, and stated that persistent AR was more common in children with AH. Among AR patients, they detected moderate rhinitis in 90 (76.3%) patients with AH, in 274 (62.6%) of those without AH, and reported that AH increased the severity and extended the duration of the disease (357). Ameli et al. (358) investigated adenoid tissue volume and symptom scores on 205 children. They reported that 60.8% of the children with Grade 1 (smallest) and 63.8% of the children with Grade 2 adenoid volumes were mono-sensitized, and found that 60.7% of the children with Grade 4 adenoids (largest) were non-allergic. They found an inverse correlation between AH and atopy.

The size of adenoid may affect the severity of symptoms in children with AR. Nuhoğlu et al. (359) compared the lateral skull X-rays of 52 allergic children and 56 children with NAR for the adenoid volume. The adenoid / nasopharynx ratio was significantly higher in the non-allergic group. Bozkurt et al. (360) performed skin prick test on the patients who had adenoidectomy, and compared the removed adenoid’s volume, VAS scores, and adenoid size on flexible nasopharyngoscopic examination between the prick test positive (32 patients) and negative (52 patients) groups. They reported significantly higher adenoid volume in patients with AR.

Some investigators analyzed immune mediators in the adenoid tissue. They hypothesized that AH might develop due to immune responses in the adenoids. They showed that CD1a + Langerhans cells, eosinophils and IL-4 and IL-5 mRNA positive cells were more in number in the adenoid tissues of allergic children compared to non-atopic ones (361-363). Alaygut et al. (364) studied expression of CD23 in the adenoid tissues of 100 2-3-year-olds who had adenoidectomy or adenoidectomy and tonsillectomy. CD23 expression was significantly lower in patients with pollen allergy.

Some authors claimed that anti-allergic treatment might be beneficial, and be an alternative to surgery in patients with AH. Although there is no sufficient data for antihistamines, a number of studies reported the benefit of nasal corticosteroids. Chohan et al. (365) included 8 randomized controlled studies into their meta-analysis, and reported that mometasone nasal spray decreased adenoid volume and improved adenoid/choana ratio significantly. In another meta-analysis, Chadha et al. (366) reviewed 7 studies (6 randomized controlled studies and one cohort) including 493 patients. They reported that various nasal corticosteroids (mometasone, beclomethasone, flunisolide) reduced symptom scores and adenoid size as measured on fiberoptic nasopharyngeal endoscopy.

Although the exact role of AR in the etiology of AH has not been demonstrated, allergy should be questioned in all children with symptomatic AH, and anti-allergic treatment should be administered in case of a positive history. Adenoid examination should be done particularly in preschool children with AR. On the other hand, double-blind controlled studies are needed to clarify the relationship between AH and AR, and the role of medical therapy in this relationship.

**Keywords:** Allergic rhinitis, adenoid hypertrophy.

### 6.8.2.7. Cough

Cough affects about 10-20% of adults, and it has three main etiological factors: upper airway cough syndrome (postnasal drip syndrome), asthma, and gastroesophageal reflux (GER). Cough may frequently be seen in AR and sinusitis. In these diseases, postnasal secretions stimulate nerve endings in the hypopharynx and larynx. Sensitivity to environmental factors is another cause of cough. Cough becomes more severe when both environmental and endogenous factors come into play. Aspiration of postnasal discharge and nasal secretions may result in cough, and its severity may increase due to underlying disorders such as asthma, cough-variant asthma, eosinophilic bronchitis and GER (367).

Chronic upper respiratory cough syndrome (postnasal drip syndrome) and / or subclinical inflammatory changes in the lower respiratory tract have been mainly blamed for the stimulation of the afferent nerve endings in patients with AR. Some studies reported increased reactivity at these nerve endings. Cough responses to capsaicin were compared in the patients with pollen allergy in the pollen season and out of the pollen season. The responses of allergic and non-allergic patients were also compared out of the pollen season. Cough responses to capsaicin was better in the pollen season in patients with pollen allergy. The allergic patients had better cough response to capsaicin compared to non-allergic patients in out of pollen season (368-370).

**Keywords:** Allergic rhinitis, cough.

### 6.8.2.8. Skin rash

The prevalence of skin rash has been reported higher in patients with AR compared to non-allergic ones. This rate is 10-15% in adults with AR, and 3% non-allergic ones (38, 371). A high coincidence has been shown between atopic dermatitis and AR. Specific IgE response to allergens, and mast cell and eosinophilic infiltration have been demonstrated in both diseases. The mechanisms linking these two conditions are complex and not completely understood. Genetic, epithelial barrier defects and *Staphylococcus aureus* colonization are seen in both conditions (372).

It has been shown that the individuals who have one atopic disease have a higher risk for developing another atopic condition. The risk of infectious skin disease was found significantly higher in a study performed on 15,530 patients with atopic eczema. Similarly, 6835 pediatric patients with AR have been shown to have an increased risk for otorhinolaryngologic symptoms and disorders (373).

**Keywords: **Allergic rhinitis, atopic dermatitis.

### 6.8.2.9. Sleep disorders

Sleep is essential for physical and mental health. Chronic allergic respiratory diseases affect sleep mildly or moderately. Adults usually experience sleep disorders and disturbance of performance due to chronic rhinitis, however decision-making and motor abilities may also be impaired (344). A number of AR patients complain of sleep disturbance (374). Difficulty of falling asleep and frequent awakening at night have also been reported in patients with AR (375, 376).

It has been supposed that nasal congestion is the most important factor for sleep impairment in patients with AR (377). Nasal congestion has a circadian rhythm, changes with position of the patient, and it gets worse at night, on supine position (378). The severity of sleep impairment is directly correlated with the severity of the disease.

A study on 600 patients showed that sleep impairment was more severe in patients with severe AR compared to mild AR (379).

Pittsburgh Sleep Quality Index and Epworth Sleepiness Scale were applied to 2200 patients. In this study, 88% of the patients had moderate to severe, and 12% had mild AR. Poor sleep quality was found in 53%, and excessive daytime sleepiness was found in 21% of the patients. In the logistic regression model, it was shown that moderate to severe rhinitis and nasal congestion accompanied poor sleep quality (380). A systematic review on children reported a significant relationship between AR and sleep disordered breathing, including obstructive sleep apnea and snoring (381). Snoring and atopy have been shown to be strongly correlated in infants (382, 383). Obstructive sleep apnea syndrome should be evaluated in adults and children with chronic rhinitis and sleep disordered breathing.

**Keywords: **Allergic rhinitis, disordered sleep, obstructive sleep apnea.

### 6.8.2.10. Cognitive disorders and learning disability

A direct correlation has been shown between the severity of allergy and decreased productivity and concentration at work. Disturbed concentration leads to errors and a decrease in the ability to cope with the problems at work (384). Impairment of quality of life has also been reported (385).

**Keywords: **Allergic rhinitis, cognitive dysfunction.

### 6.8.2.11. Sexual dysfunction

Women with symptomatic allergic rhinoconjunctivitis have significantly lower Female Sexual Function Index scores compared to the treated patients and the controls. International Index of Erectile Function scores were significantly higher in men with rhinoconjunctivitis compared to the control group and the group treated with antihistamine (386). The mechanism has not yet been determined

Another study reported that sexual activity in patients with AR was negatively affected compared to the control group and patients with NAR. In addition, AR treatment was also shown to influence sexual function (385).

**Keywords: **Allergic rhinitis, sexual dysfunctions, psychological

## 7. Treatment of allergic rhinitis

### 7.1. Environmental control

### 7.1.1. Control of indoors: methods for avoiding indoor allergens

**Keywords:** Allergy, allergic rhinitis, allergic reaction, allergen, fungi, molds, mites, acariside, cockroach, cat, dog, mouse, mice precautions, control

The major allergens of cats and dogs are found in their skin, hair follicles and saliva. Since these allergens are smaller than 10-20 µm, they can suspend in the air for a long time, and easily stick to clothes and surfaces (387). Therefore, they can be transported even over long distances. The allergens are not present only in homes where cats and dogs are fed, but also in other homes, schools and workplaces.

The most effective avoidance measure is removing the pet from the house (388, 389). On the other hand, the individuals hypersensitive to cats and dogs may be exposed to their allergens at outdoors. Although frequent washing of pets decreases the amount of suspended allergen in the air, the allergens do not remain in decreased amounts after cleaning, and the expected benefit cannot be obtained. Washing the pets is not a preferred avoidance measure since washing the dogs and particularly the cats is not practical, and the benefit is small (96, 387, 388).

High-efficiency particle filters provide 30-40% reduction in cat allergens suspended in the air, however there is no significant reduction in pet allergens placed on domestic surfaces, and hence the AR symptoms do not improve (387, 390).

Mice pose a risk in terms of AR and asthma in houses of low-middle income groups, as well as schools, shops, restaurants and animal laboratories in city centers (391). The mice allergens may suspend in the air for a long time since their major allergen excreted by urine, Mus m1, is carried on particles smaller than 10 µm (387). Mice-hypersensitive individuals are recommended for meticulous cleaning, closing cracks and holes in the house, installing traps for mice and using poisons if necessary (390-392). In order to prevent the mice from reaching the food in the home, the food and feed of the pets in the house, such as birds, cats and dogs, should be kept in plastic boxes, out of the reach of the mice. The garbage should not be collected so that the mice do not reach easily, and should be collected and removed from the house frequently. Although it is recommended to feed a cat at home to remove the mice, it must be kept in mind that cats cannot completely destroy the mice, and cat allergy may develop (387, 391).

Cockroaches are one of the major risk factors in childhood allergy and asthma. They live in crowded cities inhabited by people with low socioeconomic levels (392). Studies on this subject mostly included allergic asthma patients. The most effective method of protection against cockroach allergy is to fight home pests professionally (389). Covering holes in the house, use of pesticides and meticulous cleaning reduce cockroach allergens significantly. The use of gel form of fipronil or indoxacarb-containing pesticides reduces the number of cockroaches and relieves allergy-asthma symptoms in patients with asthma due to cockroach hypersensitivity (393). Spray pesticides are not recommended as the sprays themselves may cause allergic reactions. In addition, professional support is available to fight insects (387).

A number of house dust mites have been identified. The most frequent ones are *Dermatophagoides pteronyssinus* and *Dermatophagoides farinae*. Farmers, seed workers, and food industry workers are more often exposed to grain mites. House dust mites live in humid and hot environments. They are densely found on surfaces of sheets, blankets and duvet covers since they feed on the skin and hair debris of humans (389).

Reducing house dust mites did not result in improvement of AR symptoms. Mite-proof duvet covers and high efficiency particulate arresting (HEPA) filters reduce house dust mites (389). Nasal symptoms improve in patients with asthma with use of HEPA filter air purifiers (393). Keeping the humidity between 35-50% in the house decreases the reproduction rate of house dust mites (394).

The use of acaricides may improve AR symptoms (395, 396). Marked side effects of acaricide sprays have not been demonstrated. Clinical benefit of the mite-proof bed covers and HEPA filters has not been proven in AR and asthma patients, although they lead to a significant reduction in the number of house dust mites (389, 397, 398).

Presence of high moisture and molds in the house increases the risk of AR and rhinoconjunctivitis (399). There is a close relationship between mold smell in the house and AR symptoms (399). Methods such as reducing moisture in the home, removing molds and removing contaminated materials from the environment reduce the morbidity of allergic diseases (400, 401). Relocation of asthma employees from moisture-damaged buildings and repairing water leaks are recommended to stop recurrence and progression of the disease (402, 403).

The most efficient and sustainable allergen avoidance may be achieved by educating hypersensitive individuals on measures for indoor allergens, and changing their habits (403).

### 7.1.2. Control of outdoors: methods for avoiding outdoor allergens

Pollen is the first allergens identified by Charles Blackeley in 1860. In order for pollens to cause an allergic reaction, they should be present in the environment in high concentrations, transported by wind, and have antigenic properties. Pollens are small male reproductive units with a diameter of 5-200 microns, containing a large number of cells. Pollen contains a large number of allergic proteins. These proteins cause symptoms in hypersensitive individuals. The pollens small enough to be carried by the wind are significant in terms of allergy. They can be transported to very long distances and enter indoors (404).

The size of the pollen is important for the symptoms. Big pollens are allergens of upper airway and conjunctiva while small ones may reach to lower airways and cause symptoms.

The most frequent allergens causing AR show regional differences. The climate and vegetation are different in all geographical regions of Turkey. Types and numbers of pollens show differences in terms of regional temperature and climate. The pollen concentration in the atmosphere varies depending on the regional vegetation, the amount of precipitation, and the direction and speed of the wind. The pollen calendar is the first step for allergen avoidance. Now, pollen collectors are placed in the city centers, and the pollen calendars are available in almost every country, including ours (405).

The highest amounts of pollens in Turkey originate from Cupressaceae (cypress, juniper), Pinus (pine), and Gramineae (grass), and the pollens are in the air between March and June (168).

The main pollens causing allergy are meadow grass, weed and tree pollens. Tree pollination takes place between February and May, grass pollination occurs in June and July, and weed pollination continues from August to the last months of the year. In addition, some pollens are in the air between March and November. The diameter of pollens usually range between 5-100 microns (406).

People who are allergic to pollens are mostly symptomatic when the weather is dry, hot and windy. The pollen amount in the air is the highest in the morning hours. If possible, the patients should not spend time outdoors or wear a mask during these hours. The bedroom window should not be opened in the morning, and the windows should be closed when going to bed at night in the pollen season. There should be air conditioners with a pollen filter in the house and cars, the car window must be kept close in the morning. Recirculation mode should be turned on in the air conditioner of the car (407).

The patient should take a shower as soon as he/she returns home, or wash his/her face with plenty of water. Sprays may be used for nasal cleaning. Outfits such as coats and vests worn outside should be shaken outside while entering the house, and should not be kept in the bedroom (408). Pollens that are attached to the hair, skin and clothes may be carried to the indoors (409).

The bedroom should be protected from pollens as much as possible, and remain as a safe area. Outerwear should be changed as soon as entering the interiors. Although recommended, it is not always possible to change the place of residence for those who have extensive complaints due to the pollens.

In case of being in the park, garden and green areas at the weekends, the nose and face should be washed with plenty of water, and a nasal douche must be performed.

Mold spores are responsible for both perennial and seasonal allergies. Their size is 2-250 microns. Outdoor molds peak in mid-summer, and their number decrease when the weather gets cold (408). The numbers of Alternaria, Cladosporium and Epicoccum spores increase in dry weather and in the afternoon. Alternaria is usually found in soil, near flowers and tree roots. Cladosporium, is the most common fungus in the temperate regions. Aspergillus is usually together with house dusts, and is found extensively in organic fertilizers and dead plants outdoors. Penicillium is found in soil, in foods such as seeds, and with house dust. All these outdoor molds can live in damp, sunless rooms of buildings, wallpapers, and the inhaled air may increase allergy complaints. Patients who are allergic to molds should not keep flowers in their rooms, and should stay away from forest and soil after rain (409).

Today, especially in Europe, there are centers and mobile applications that track the pollens in the air and make the pollen maps to inform patients (410). Pollen forecasts of Turkey may be followed from web pages www.polleninfo.org and www.medaeronet.net. In addition, pollen counts of some cities are sent to Turkish National Allergy and Clinical Immunology Society on daily basis, and may be found in the web page of this society (www.aid.org.tr).

### 7.2. Pharmacotherapy

### 7.2.1. Corticosteroids in treatment of allergic rhinitis

**Keywords: **Administration oral, administration intranasal, allergic rhinitis, anti-allergic agents, beclomethasone dipropionate, betamethasone, budesonide, ciclesonide, corticosteroids, dexamethasone, flunisolide, fluticasone furoate, fluticasone propionate, methyl prednisolone, mometasone furoate, nasal sprays, prednisolone, safety, triamcinolone acetonide.

### 7.2.1.1. Systemic corticosteroids

Systemic corticosteroids have never been proposed as the first line treatment options in AR Guideline (38, 96). However, it has been stated that they can be used in moderate-severe persistent AR patients that do not respond all other treatment options (411, 412). Today, topical agents and immunotherapy provide benefit in most of the patients, and they are more reliable treatment options with fewer side effects, therefore systemic corticosteroids are almost never needed in AR treatment (38, 413, 414). There is no sufficient data in the literature on the therapeutic index (effect/adverse effect) of systemic corticosteroids in AR. In addition, there is no consensus on the place of systemic steroids in AR treatment due to the lack of controlled studies on the dose-response relationships based on the severity of the disease, complaints of the patient and the findings of physical examination.

The short-acting corticosteroids may be administered through oral route. On the other hand, long-acting corticosteroids may be used parenterally, as depot injections. Oral prednisolone may be started at a dose of 20-40 mg/day (38). Then, the dose is reduced on a daily basis, and it is stopped in 3 weeks at maximum. Some authors prefer 0.5 mg/kg/day oral prednisolone for 5-10 days (415). Methyl prednisolone (40-80 mg), betamethasone (2-10 mg), triamcinolone acetonide (40-80 mg) or dexamethasone (8-18 mg) may be administered intramuscularly, as depot corticosteroids (416), however depot injections are not recommended due to adverse effects including osteoporosis and diabetes (38, 414).

Side effects of systemic steroids include infections, adrenocortical insufficiency, diabetes, peptic ulcer, glaucoma, and moon face development. Systemic corticosteroids should not be preferred in the presence of diabetes mellitus, severe hypertension, severe peptic ulcer, severe osteoporosis, glaucoma, herpetic keratitis, psychotic disorders, tuberculosis and similar chronic infections (45, 412).

Systemic corticosteroids may be used for their systemic anti-inflammatory effects for ophthalmic, nasal and general complaints of allergy. In addition, they may be used in patients refractory to other treatment options, particularly the ones with hyposmia. Short-term systemic corticosteroids may be administered to those who have severe perennial rhinitis, AR accompanying nasal polyps and in case of permanent risk for anosmia (411). More often, short-term oral prednisolone (20-40 mg / day, 4-7 days) can be used in patients if intranasal corticosteroids (INS) are not sufficient for severe nasal obstruction and laryngopharyngeal symptoms (45). Systemic corticosteroids are effective in reducing eosinophil migration and suppressing mediator release during the late phase response of AR (96). In the updated AR diagnosis and treatment Guideline, no recommendation has yet been made on the short-term use of systemic corticosteroids in patients with severe AR (96, 417). On the other hand, systemic steroids did not have superiority over intranasal corticosteroids in the control of AR symptoms (418). In one study, treatments schemes including systemic or nasal corticosteroids were found to be more successful in achieving symptomatic improvement compared to schemes with antihistamines, while no significant difference was found between oral betamethasone and intranasal mometasone for symptomatic improvement (418). Therefore, despite strong anti-inflammatory effects of oral corticosteroids, symptomatic improvement they provide is not much different from nasal corticosteroids, and they are not recommended in the routine treatment of AR due to their possible systemic side effects.

### 7.2.1.2. Nasal corticosteroids

Nasal corticosteroids (NCS) are effective in treatment of AR (38, 45, 96, 415). New generation NCS are found in trace amounts in the systemic circulation, and their long-term use does not result in nasal mucosal atrophy; therefore they are the most frequently used medications in treatment of AR (296, 419-421). NCS are better than systemic corticosteroids for inhibiting inflammatory cell migration into the nasal mucosa (422). They directly modulate AR pathophysiology with their strong anti-inflammatory properties. They do this by suppressing cytokine release in secretions of nasal mucosa, and by inhibiting basophils, eosinophils, neutrophils and mononuclear cells (423). Although recently “corticosteroid resistance” has been proposed for the patients unresponsive to NCS, there is not yet sufficient data regarding the resistance to corticosteroids at the molecular level (424).

It has been shown that NCS are effective on all symptoms of moderate /severe AR, including sneezing, itching, nasal congestion, rhinorrhea and ocular symptoms (38, 96). These agents are effective particularly on nasal congestion, and they significantly improve the quality of life (419). In addition to the reduction of nasal symptoms, NCS are beneficial for ocular symptoms, including itching, redness and swelling of eye (412). Nasal steroids have also been shown to improve hoarseness (426) and sleep quality (96, 427). In addition, their positive effects on smell disorders have been reported in experimental models and clinical studies (428, 429).

All NCS are similar for their efficacy. The onset of action is usually 2-8 hours after application, however the maximum effect is evident 7-14 days later (296). Regular use of NCS is recommended since this is more effective than intermittent use. Absence of an improvement in symptoms in the follow-up visit may suggest inefficacy since it has been known that NCS exert their maximum effect approximately 2 weeks after the onset of treatment (38).

Rhinosinusitis, nasal polyposis, smell disorders, adenoid hypertrophy, lymphoid hyperplasia in the nasopharynx, obstructive sleep apnea, Eustachian tube dysfunction, otitis media with effusion, atopic dermatitis, asthma, allergic conjunctivitis, chronic cough, laryngitis, and accompanying gastroesophageal reflux are the main comorbid conditions of AR (283, 430). Apart from their benefit in comorbid conditions affecting the upper respiratory tract such as nasal polyposis, smell disorders, adenoid hypertrophy, lymphoid hyperplasia in the nasopharynx, Eustachian tube dysfunction, and otitis media with effusion, their benefits are also investigated in disorders of lower airways, including asthma. AR and asthma have similar epidemiological and pathophysiological properties, and this partnership contributes mutually to the treatment approach (431). Corticosteroids have been shown to protect the lower respiratory tract by preventing ascending infections and reactive inflammation in asthmatics (432). In some studies, it has been suggested that the use of NCS improve attack control in asthmatics with simultaneous AR (296, 432).

Hypertrophic adenoids may get smaller and the volume of the nasal cavity may increase with the use of NCS (365, 433-435). Comparative studies showed that NCS are more effective than oral antihistamines in the control of nasal symptoms, while there was no significant difference between these two agents for ocular symptoms. NCS are more effective than leukotriene receptor antagonists in the control of allergic complaints. However, it has been shown that nasal antihistamines are superior to NCS in terms of rapid onset of action (96). In a data pool study, improvement in total nasal symptom scores with mometasone furoate was better in both seasonal and perennial AR groups compared to montelukast, desloratadine, and even immunotherapy (436).

NCS are tolerated well. Crusting, dryness, epistaxis, and burning sensation are rare local adverse effects. Patients may complain of bitter taste and a bad smell (419, 427). It has been reported that adverse effects related to local sensations have been minimized with some aerosol formulations, and the patients tolerated NCS better (427, 437).

Recent studies compared mometasone furoate and fluticasone furoate since they are the most widely used nasal preparations (438). Some authors claimed that the patients preferred fluticasone furoate over mometasone furoate due to less bitter taste and irritation in the nose, and less medication flowed into the throat (439, 440).

The prevalence of epistaxis has been reported in a wide range. There are rare reports in the literature concerning nasal septal perforation due to long-term use of NCS (441). No signs of atrophy were detected in the nasal mucosa samples of patients using long-term NCS due to AR (296, 421, 442). Although local ocular findings such as chorioretinopathy or glaucoma are rarely reported with the use of NCS (441), it has been shown that there is no significant thickening of the coronal and retinal membranes (443) and intraocular pressure does not increase (444).

In order to analyze the effects of NCS on the hypothalamic-pituitary-adrenal axis, 2-4 weeks of kinemometry was performed for their short-term effects, and 12 months of stadiometry was employed for their long-term effects (419, 441, 445, 446). Although some studies have shown that beclomethasone dipropionate (447) and fluticasone furoate (445) sprays lead to growth suppression, mometasone furoate spray does not have any adverse effects on growth rate (448). However, some studies showed that beclometasone dipropionate nasal aerosol did not have any negative effects on the hypothalamic-pituitary-adrenal axis (449). In addition, no statistically significant difference was observed between the growth values measured in children using triamcinolone acetonide nasal spray and placebo (450). Although stadiometric measurements show that there is no decrease in human growth in the long term, since some studies detected a decrease in growth rate by kinemometric measurements in the short term, it would be more rational in clinical practice to use NCS preparations that have been shown to have no negative effect on growth of children (446). The lower age limit of beclomethasone spray in our country is six years, however this limit is two years for other NCS. In addition, it has been shown that mometasone furoate was superior to beclomethasone dipropionate in terms of efficacy and safety in the pediatric age group (451).

Numerous different corticosteroids have been administered intranasally from past to present. A first generation corticosteroid, dexamethasone, is no longer preferred due to its side effects (452). The second generation corticosteroids, including beclomethasone dipropionate (aerosol) (453-460), budesonide (aqueous) (461-463), ciclesonide (aqueous or aerosol) (464-466), flunisolide (aqueous), fluticasone furoate (aqueous) (467-470), fluticasone propionate (aqueous) (471-474), triamcinolone acetonide (aqueous) (475-476) and mometasone furotate (aqueous) (466, 477-482) have been used in nasal spray preparations until today.

Beclometasone dipropionate is a prodrug. Others become less active quickly, and produce fewer side effects with minimal systemic absorption. Water-soluble agents such as budesonide pass into the systemic circulation in higher amounts unlike lipophilic fluticasone and mometasone (412). The lipophilic corticosteroids are absorbed through the cell wall into the cell in higher amounts, and in case of nasal administration, into the nasal mucosa. Furoate or propionate ester chains increase the lipophilic properties of the molecule; therefore the systemic effects of the drug are minimized while its local effects are maximized (483). The systemic bioavailability rates of second generation NCS including mometasone furoate, fluticasone propionate, ciclesonide and fluticasone furoate are less than 1% while systemic bioavailability rates of older molecules including budesonide, beclomethasone dipropionate and triamcinolone acetonide are much higher (34-49%) (412, 484).

Laser aerosol spectrometry was used to study the droplet sizes and distributions in the nasal cavity after use of NCS sprays. Droplet storage was detected in a larger mucosal area with fluticasone furoate containing nasal preparations compared to the ones containing fluticasone propionate and mometasone furoate (485). Triamcinolone acetonide and flunisolide bind to plasma proteins less, and pass into the systemic circulation in smaller amounts. Mometasone furoate has the highest affinity for corticosteriod receptors. This is why preparations containing fluticasone furoate and mometasone furoate were found to be more safer for use in pregnancy (486).

Different molecules used in nasal preparations are comparable in efficacy and treatment compliance. NCS are usually prescribed at a dose of two puffs into each nostril, once or twice a day (96). Recently, new aerosol forms have been developed aiming to increase duration of stay in the nose, and to decrease storage in the nasopharynx and oropharynx (437, 487). This goal can only be achieved with appropriate use of the nasal sprays. The use of contralateral hand is recommended to avoid traumatic epistaxis, and administer an effective dose to lower turbinate, anterior half of caudal septum and middle meatus (488). In addition, the nasal examination findings before administration of NCS are very important (489). For example, in the presence of nasal polyps, the absorption of the NCS from the polyp epithelium is less than its absorption in the nasal mucosa, and the polyps create a barrier for NCS to reach the nasal mucosa. Therefore, the potency of the NCS sprays decreases in presence of nasal polyps (412). In the presence of congestion in the nasal mucosa and turbinates on nasal examination, it must be noted that NCS cannot reach the nasal cavity mucosa easily, and cannot be easily distributed into all structures in the nose. In addition to patient-specific physical examination findings, the use of quality of life questionnaires in the follow-up of patients with AR may increase success in symptom control as well as treatment compliance due to different socio-demographic and personal characteristics of the patients (488, 490). Administration of medical treatment effectively should be the responsibility of parents and, if possible, mothers, in pediatric patients (491). The pharmacist or other relevant worker should also direct the patient correctly in order to increase patient compliance (492).

The American Academy of Otolaryngology and Head and Neck Surgery (AAO-HNS) has prepared AR diagnosis and treatment Guideline. They have strongly recommended use of NCS in patients with AR, particularly when the disease affects quality of life (96) 2016 revision of ARIA guideline recommends to take patient preferences and cost of treatment into account while planning treatment of AR. In this context, NCS have been recommended alone or in combination with oral antihistamines for seasonal AR, and recommended alone for perennial AR. This guideline also recommends NCS alone or in combination with nasal antihistamines in treatment of seasonal and perennial AR, and includes NCS in various combination therapy choices of AR (417).

In conclusion, NCS are well tolerated by the patients, and may be the first treatment option in patients with moderate/severe intermittent and mild persistent AR, as well as in patients with moderate/severe persistent AR.

### 7.2.2. Oral antihistamines

**Keywords:** Histamine, H1 antihistamines, anti-allergic medications, medications, anti-allergic agents, antihistamines, allergic rhinitis, antagonists

### 7.2.2.1. H1 antihistamines

Traditionally, oral antihistamines are the first-line treatment options for AR patients worldwide. First generation H1 antihistamines are lipophilic. In addition to crossing the blood brain barrier and binding to histamine receptors, they block muscarinic, adrenergic (or adreno-ceptors) and dopaminergic receptors, causing sedation and cardiovascular, urinary and gastrointestinal adverse effects (493, 494). New molecules have been investigated owing to these undesirable adverse effects, and second generation antihistamines have been developed which have limited penetration into the central nervous system (CNS) as well as a high selectivity to the H1 receptors (495). The reason for this feature of second generation antihistamines is their high affinity to P-glycoprotein (P-gp) in the brain capillary endothelial cells acting as a pump, and their hydrolysis by an ATPase-dependent mechanism. Therefore, this group of antihistamines are also called as “minimally sedative H1 antihistamines”. The lack of effects on CNS is the most important difference of second generation antihistamines compared to first generation ones (496).

AR is actually a systemic disease. Allergic symptoms begin 6-12 hours after exposure to the allergen, and peak at 12-24 hours. Apart from nasal symptoms, oral antihistamines are effective in ocular itching, irritation and redness, oral and pharyngeal symptoms, and all dermatological symptoms (492, 493). Topical nasal H1 antihistamines are also available, and their effectiveness is similar to that of oral formulations. They have a strong effect by reducing the nasal symptoms strongly within 30 minutes (38).

Second generation antihistamines are the most preferred treatment agents for the treatment of allergic symptoms thanks to their high selectivity for the H1 receptors, as well as their high efficacy and less side effects (497). On the other hand, some second-generation H1 antihistamines have serious side effects, including serious life-threatening cardiotoxicity. For this reason, they are not in use in many countries (493). The change of antihistamine drugs in time is shown in Table 7.2.2.1.1.

In addition to sedation, use of first-generation antihistamines may result in traffic accidents. Diphenhydramine is a well-known molecule in this regard. Aviation accidents were examined, and it was reported that the most commonly used medication in deceased pilots was diphenhydramine. Local airline pilots are approved to use second-generation antihistamines (loratadine, desloratadine and fexofenadine) in the USA (498). In another study, the researches administered 20 or 40 mg/day single dose and 50 mg/day single dose bilastine to the flight crew, and found that bilastine had a similar effect with placebo over the 6-hour study period, but the ability to work significantly decreased in those using hydroxyzine (499).

The most frequently prescribed second generation antihistamines are cetirizine, desloratadine, ebastine, fexofenadine, levocetirizine, loratadine, bilastine and rupatadine. The antihistamines curently in use are listed in Table 7.2.2.1.2, with their trade names. Almost all of these preparations are prescribed when necessary.

Cetirizine is a second-generation antihistamine with a proven efficacy in patients with perennial AR at a dose of 10 mg/day. Although it showed a significant improvement in symptom severity compared to placebo, adverse effects such as headache and performance impairment were also observed (500). Learning and concentration disorders have been reported in children using cetirizine, which are supposed to be due to antihistamine and anticholinergic effects of the molecule. This side effect is a common problem for children, parents and teachers. Sometimes parents and teachers cannot find an underlying cause in children who have problems at school; adverse effects of medications should be taken into account in this case (493).

Levocetirizine has been shown as the best treatment option when compared to other second-generation antihistamines due to its beneficial effect on persistent AR (495, 501). Various clinical trials showed that it caused a significant improvement in quality of life in simultaneous perennial AR and asthma (495).

Although cardiac side effects of second generation antihistamines are extremely rare, a high risk of ventricular arrhythmia was reported with ebastine (502), and its use limited to over 12 years of age (503).

Bilastine is a non-sedative oral antihistamine with proven in vitro and in vivo selectivity for the H1-receptors. It is not metabolized by cytochrome P450 system (504). Ninety-five percent of it is excreted from the body unmetabilized. It not metabolized in the liver, and has high therapeutic efficacy (505). Research on healthy volunteers and patients has shown that this agent does not affect the ability to drive, alertness or cardiac conduction, and does not cause arrhythmias (495, 505). It can be used safely in AR, rhinoconjunctivitis, and urticaria (493, 498).

Rupatadine was launched in 2003 as an antihistamine with a strong H1 receptor blocking activity and anti-PAF effect. It has a wider mechanism of action, used in the treatment of chronic urticaria and AR (506, 507). Rupatadin is a selective, long-acting H1 antihistamine that has both anti-allergic and anti-inflammatory properties (506). It has been used in the treatment of urticaria, rhinorrhea, sneezing, nasal itching, nasal congestion and tearing symptoms at a dose of 10 and 20 mg/day, and significantly improved these symptoms compared to placebo (458, 506). Comparative clinical studies showed that it was as effective as loratadine, cetirizine, desloratadine and ebastine in controlling symptoms in adult and adolescent patients with seasonal and non-seasonal AR (506, 507). It is metabolized mainly by cytochrome P450 (CYP) isoenzyme CYP3A4 in liver, and excreted in bile. Drug interactions are observed with some agents that inhibit CYP3A4 activity (eg. ketoconazole, erythromycin, grapefruit juice), and rupatadine is not recommended to be used together with those (508, 509).

The second generation antihistamines registered in Turkey, and their trade names are presented on Table 7.2.2.1.3 according to their specialties.

### 7.2.2.2. Adverse effects

Adverse effects of H1 antihistamines are due to their binding to receptors other than H1 (510). First generation H1 antihistamines bind cholinergic-muscarinic receptors, and dose-related anticholinergic side effects such as sedation, mental impairment, dry mouth, dry eye, urinary retention and constipation may be seen (504). Drug-drug and drug-food interactions can be seen, since most antihistamines are metabolized by the cytochrome P450 system (CYP) and particularly CYP3A4 in the liver and intestine wall. The relationship of drugs with cytochrome P450 is shown in Table 7.2.2.2.1. Simultaneous use of antihistamines with the agents that inhibit the CYP3A4 isoenzyme leads to an increased concentration of antihistamines in the serum, which leads to an increased risk of cardiac toxic side effects in proportion with the level of potassium channel blockage of the agents (495). Therefore, the use of the antihistamines metabolized by P450 system is not recommended in patients with hepatic disorders, hereditary long QT syndrome, in combination with other drugs that extend the QT interval (macrolides, itraconazole and ketoconazole) and CYP3A4 isoenzyme inhibitors (505, 509, 511).

Research has shown that adverse effects of bilastine were minimal when compared to placebo. Studies on healthy volunteers confirmed that it had minimal effects on psychomotor performance, even when administered up to four times of the recommended dose (512). Studies in terms of cardiac safety have shown no effect on the QTc interval or other electrocardiogram parameters (513).

There are several case reports on hepatic toxicity due to levocetirizine and its active R-enantiomer (514). Cases with skin rash (drug eruption) (515) and interstitial pneumonia (510) have also been reported. Patients with urticaria treated with levocetirizine had more psychomotor disorders compared to those treated with rupatadine. Levocetirizine and rupatadine were compared in the treatment of chronic urticaria, levocetirizine was found superior to the rupatadine, and both agents caused drowsiness in similar rates (516). When deciding on an antihistamine, the tolerability and safety profile of the agent should always be kept in mind. The most common adverse effects of antihistamines are shown in Table 7.2.2.2.2.

International allergy study groups including ARIA and International Primary Care Respiratory Group (IPCRG) emphasized that second generation antihistamines should be used as a first-line treatment in patients with AR, and new generation drugs should be preferred. Therefore, non-sedative antihistamines constitute the backbone of allergy treatment. In addition, symptoms and concomitant diseases as well as severity of the symptoms are important for the selection of the therapeutic agent. Therefore, the authors of this chapter prefer a patient-specific treatment approach and medication selection. Easy tolerability, a good safety profile and price are important factors for drug selection, but it is considered that all second-generation antihistamines have acceptable sedative properties, and do not impair learning.

The second generation antihistamines in Turkey are comparable for their effectiveness to control AR symptoms, therefore the selection of drug depends on its adverse effect profile, particularly CNS suppression. Possible sedative effects, wide therapeutic index, appropriate pharmacokinetics and low-dose administration should be considered for selection of an antihistamine. The profession of the patient is of particular importance for antihistamine selection since even a small sedative effect may have serious consequences in some professions.

### 7.2.3. Nasal antihistamines

Antihistamines have been used for more than seventy years in the treatment of allergic disorders. Although the topical treatment approach has been used extensively in the respiratory tract for centuries, oral route has been preferred for antihistamine treatment in AR. The advantage of topical administration is less systemic absorption and hence smaller risk for systemic adverse effects. Nasal administration of antihistamines has been popularized in the past two decades (517).

Nasal antihistamines also have anti-inflammatory activities in addition to their antihistamine activity (518). Their anti-inflammatory effects include mast cell stabilization, inhibition of chemokine release, and inhibition of inflammatory cell chemotaxis and migration (519, 520). In addition, it has been shown that they suppress release of cytokines such as interleukins and TNF-a as well as suppression of neural inflammation (521-526). Owing to these properties, nasal antihistamines may also be used in treatment of non-allergic rhinitis (527).

Although nasal antihistamines exert their anti-inflammatory effects at their daily recommended doses, oral antihistamines need to be used at much higher doses than their recommended dose to show their anti-inflammatory effects. Therefore, recent Guideline have reported that nasal antihistamines may be used as first-line therapy in AR (30).

Today, there are two approved nasal antihistamines: azelastine and olopatadine (528, 529).

### 7.2.3.1. Azelastine

Azelastine is a phthalazinone derivative, a second-generation antihistamine with a high affinity for H1 receptors. Its affinity for binding to H1 receptors is ten times higher than chlorpheniramine (530). Nasally administered azelastine has a rapid onset of action. Randomized double-blind, placebo-controlled studies investigating the effectiveness of azelastine reported that the activity of azelastine nasal spray started in the first 15 minutes after its application, relieving AR symptoms, and this activity continued for 8 hours (531-534).

The most common side effects expressed by patients using azelastine are bitter taste, headache, drowsiness and nasal burning sensation (534).

### 7.2.3.2. Olopatadine

Olopatadine selectively blocks H1 receptors, and it also inhibits the release of histamine and other pro-inflammatory mediators from the mast cells (535). Randomized double-blind placebo-controlled trials showed that olopatadine was superior to placebo in improving AR symptoms including ocular symptoms, and quality of life (536, 537). It has been determined that the activity of olopatadine starts within the first 30 minutes after application, and continues up to 12 hours (538).

The most frequently expressed side effects are bitter taste, headache, epistaxis, and pharyngeal pain. The prevalence of drowsiness was found in less than 1% of the patients (539).

### 7.2.3.3. Comparison of olopatadine with azelastine

In multicenter placebo-controlled studies comparing the efficacies of olopatadine and azelastine, no significant difference was found between these two agents concerning efficacy. Both agents were well tolerated with low adverse effect profiles. Bitter taste sensation was less with olopatadine (540).

### 7.2.3.4. Comparison of nasal antihistamines with oral histamines

Oral antihistamines have been preferred in treatment of AR despite their side effects such as dizziness, sedation and limited effectiveness in terms of nasal congestion (517).

A number of recent controlled studies reported that azelastine had fewer side effects, and had similar efficacy or superior than loratadine, desloratadine, fexofenadine and cetirizine (531, 541-544). In addition, nasal antihistamines have been shown to be more effective for improving nasal congestion. Based on these studies, it has been stated that azelastine may be used as a first-line therapy in AR treatment in patients in whom oral antihistamines are insufficient to relieve AR symptoms (542).

There are no studies in the literature comparing olopatadine with oral antihistamines.

### 7.2.3.5. Comparison of nasal antihistamines with nasal corticosteroids

In a meta-analysis of nine studies comparing azelastine with NCS, it was found that NCS provided a more effective improvement in the nasal symptoms of AR although no difference was reported for ocular symptoms (545). Three multi-center randomized studies reported that the effectiveness of azelastine nasal spray was similar in patients who could not be effectively treated with oral loratadine or nasal beclomethasone (546).

A study comparing olopatadine and fluticasone reported that there was no clinically significant difference between the two groups in terms of mean two-week symptom reduction, however it was concluded that olopatadine was more effective in the first 3 days of treatment, and its therapeutic effect appeared in a shorter time (547).

### 7.2.3.6. Comparison of onset of action of nasal antihistamines with that of nasal corticosteroids

Comparison of azelastin, mometasone and placebo revealed that the effect of azelastine started in the first 15 to 45 minutes after the application, and the superiority of azelastine continued for the first 8 hours after this application. In the comparison of azelastin with fluticasone spray and oral cetirizine tablet, it was found that the effectiveness of azelastine started within the first 30 minutes, and it was superior to all other agents in the first 24 hours (545).

A study comparing olopatadine and mometasone determined that the effectiveness of olopatadine started in the first 30 minutes and continued for 12 hours. It was observed that mometasone and placebo produced the same effect in the first 150 minutes (538).

In the light of the aforementioned studies, it has been concluded that intranasal antihistamines have a more rapid onset of action compared to all other medications used in allergy treatment.

### 7.2.3.7. Combination of nasal antihistamine and nasal corticosteroid

Patients using azelastine nasal spray, fluticasone nasal spray, and azelastine-fluticasone combination were analyzed for clinical improvement after two weeks, and the improvement rates were reported as 5%, 27% and 37.9% in the groups of azelastine, fluticasone and combination groups, respectively (548). In studies investigating the effectiveness of the combination of NCS with oral antihistamine or leukotriene antagonists did not report such an increase in the efficacy (549, 550).

The combination of NCS and intranasal antihistamines may be a good choice in the treatment of AR due to advantage of topical application and additive effects of the molecules (551).

In conclusion;

Nasal antihistamines are good treatment options in AR treatment due to the advantage of direct application to the nasal mucosa. Both azelastine and olopatadine show a fast onset of action, minutes after nasal administration. Both agents’ efficacies are similar with or superior to oral antihistamines, and they have been found to have superior efficacy for nasal congestion. Nasal antihistamines have similar efficacy with NCS. Combinations of a nasal antihistamine with NCS will be a good option in the treatment of AR due to their local application advantage and additive effects of the molecules. Another advantage of nasal administration is direct delivery of the drug to the target tissue, in a higher concentration, thereby minimizing the side effects seen in systemic administration. The most frequently reported side effects are bitter taste and sleepiness. Nasal antihistamines have been recommended in the recent Guideline as the first-line treatment options due to their efficacy and safety.

### 7.2.4. Antileukotriens

**Keywords** Perennial allergic rhinitis, Seasonal allergic rhinitis, Montelukast, Leukotriene antagonists

Leukotrienes (LT) are inflammatory mediators released from leukocytes. They play role both in the early and late phases of the allergic response (552). Cystenyl LTs, namely LTC4, LTD4, and LTE4, cause contraction of bronchial smooth muscles, mucus formation, edema and increased vascular permeability.

Administration of antileukotrienes are divided into two groups in relation with their mechanism of action:

1. Cystenyl leukotriene receptor antagonists (LTRA) block LT receptors, thus the final organ response. This group includes montelukast, zafirlukast and pranlukast.

2. Leukotriene synthesis inhibitors (5-lipoxygenase inhibitors) block the synthesis of cystenyl LTs and LTB4. Zileuton, ZD-2138, Bay X 1005 and MK-0591 are in this group (552).

Montelukast is the only cystenyl LTRA approved for AR in Turkey. It is indicated for the treatment of asthma and AR. Montelukast has FDA approval for seasonal AR treatment in adults, and children 2 years and older. It has also been approved for treatment of perennial AR in adults and children 6 months and older (553). Its use in pregnant women is category B (96). Short or long term use of montelukast does not affect skin prick test reaction (554).

### 7.2.4.1. Meta-analyses

Montelukast is effective on four cardinal symptoms of AR including nasal congestion, rhinorrhea, itching and sneezing. In addition, it is also beneficial on sleep disturbance due to nasal congestion (377). Lately, four meta-analyzes have been published on the effectiveness of montelukast in AR (555-558). The results of these studies are compatible with each other. Although montelukast is more effective than placebo for improving nasal symptoms and quality of life, it is not as effective as NCS and antihistamines, and should not be recommended as the first-line therapy. The combination of montelukast and antihistamine has a similar effect with NCS on nasal symptoms, however it has been reported that NCS provide more improvement in quality of life (555). In seasonal AR, LTRAs are more effective than placebo on daytime and night nasal and eye symptoms, and they improve the quality of life. LTRAs are as effective as oral H1 antihistamines on rhinitis and ocular symptoms and quality of life scores (556, 557). However, they are less effective on daytime and night nasal symptoms compared to NCS. The combination of LTRA and antihistamines, on the other hand, is more effective only on ocular symptoms when compared to antihistamines alone. The effects of NCS on nasal congestion are more pronounced than the combination of LTRA and antihistamine (556). The results of the last meta-analysis emphasized that, although LTRA and antihistamines have similar effects and side effect profiles in seasonal AR, antihistamines were mostly effective on daytime rhinitis and eye symptoms, and LTRAs were effective on night symptoms (difficulty of sleeping, night awakening, and nasal congestion on awakening) (558).

Montelukast improves both rhinitis and asthma symptoms in patients with simultaneous seasonal AR and asthma (559). LTRAs ere effective on nasal symptoms in patients with chronic sinusitis with nasal polyps, however their effect is similar to that of NCS, and adding a LTRA does not provide additional benefit over NCS (560). LTRAs are more effective than placebo, but less effective than oral antihistamines in treatment of seasonal allergic conjunctivitis in adults (561).

### 7.2.4.2. Other international and national publications

Recent publications reported that combination of montelukast and fluticasone propionate improved symptoms and quality of life scores in patients with moderate-to-severe AR better than fluticasone and placebo (562). A descriptive survey study conducted with another LTRA, pranlukast, reported that pranlukast reduced nasal symptoms and sleep disturbance, and improvement of nasal congestion was correlated with the improvement of sleep disturbance in perennial AR (563).

Erdoğan et al. (564) performed a randomized controlled study on 40 patients with persistent AR, and reported that the combination of desloratadine and montelukast had a positive effect on quality of life, particularly sleep symptoms, compared to desloratadine alone. In their randomized controlled trial, Yarıktaş et al. (565), compared the combination of montelukast and loratadine with montelukast alone, loratadine alone and placebo in patients with seasonal AR, and reported that montelukast or loratadine resulted in similar improvements on AR symptoms, and the combination was more effective than using montelukast or loratadine alone. Karabıçak (566), reported in his randomized controlled study that the combination of levocetirizine or montelukast with NCS was more effective than NCS alone in rhinitis symptom scores and acoustic rhinometry results. In their experimental AR model, Bozkurt et al. (567), reported that montelukast provided a significant reduction in sneezing and itching symptoms as well as IL-4 and CysLT levels.

### 7.2.4.3 ARIA reports

In the 2010 revision of the ARIA report, it was stated that LTRAs were effective in seasonal AR in children and adults, and in perennial AR in preschool children. However, it was recommended that oral H1 antihistamines should be preferred over oral LTRAs since they were less costly (568). ARIA 2016 revision reported that both oral H1-antihistamines and LTRAs may be preferred in seasonal AR treatment. The panel members have agreed that the choice would depend on the patient’s preferences, local availability and cost of the drugs. They also stated that this choice would usually be on the side of an oral antihistamine (417). LTRAs are not recommended in adults with perennial AR, since they do not have any significant clinical effect, and are costly (568). ARIA 2016 revision recommends oral antihistamines over LTRAs in perennial AR (417).

In seasonal AR, it has been recommended to prefer NCS over LTRAs because they are more effective. There are no systematic reviews comparing the efficacies of NCS and LTRAs in perennial AR (568).

LTRAs may be more useful than oral antihistamines in patients with AR and asthma, and particularly in exercise-induced asthma and aspirin-exacerbated respiratory disease (417). Inhaled glucocorticosteroids have been recommended alone for asthma control in patients with AR and asthma, before use of oral LTRAs. Oral LTRAs can be used in patients with simultaneous asthma and AR for the treatment of asthma when the patient does not prefer, or cannot take inhaled glucocorticosteroids (568).

### 7.2.4.4. Adverse effects

Montelukast is usually well-tolerated and does not cause sedation. Its most frequent side effect in children is abdominal pain (0.23%) (569). Although psychiatric adverse effects such as agitation, anxiety, depression, sleep disturbances, hallucinations, suicidal thoughts and suicidal tendency have been reported in the recent years, these effects have been reported rarely in large series. In addition, Churg Strauss Syndrome, anaphylaxis, eosinophilic infiltration, and hepatobiliary, pancreatic and uropoeitic disorders have been rarely reported (570). It caused visual hallucinations in a few patients, which disappeared 48 hours after the drug was stopped (571).

### 7.2.5. Combined preparations

**Keywords: **Allergic rhinitis, anti-allergic agents, Histamine H1 antagonists, corticosteroids, antihistamines, nasal decongestants, nasal sprays, administration oral, administration intranasal, mometasone furoate, desloratadine, montelukast, montelukast sodium, azelastine, loratadine, cetirizine, pheniramine, cromolyn sodium, chlorpheniramine, cholorpheniramine maleate, fluticasone, fluticasone propionate, leukotriene antagonists, azatadine maleat pseudoephedrine sulfate drug combination, carbinoxamine, carbinoxamine maleate, dexbrompheniramine maleate, phenylephrine hydrochloride, pheniramine maleate.

Combined preparations registered in Turkey and other countries (572):

1. Desloratadine + montelukast sodium

2. Desloratadine + pseudoephedrine HCL

3. Desloratadine + pseudoephedrine sulfate

4. Pseudoephedrine HCL + cetirizine HCL

5. Pseudoephedrine HCL + triprolidine HCL

6. Pseudoephedrine HCL + acrivastine

7. Pseudoephedrine HCL + chlorpheniramine maleate

8. Pseudoephedrine sulfate + loratadine

9. Pseudoephedrine sulfate + dexbrompheniramine maleate

10. Pseudoephedrine sulfate + azatadine maleate

11. Levocetirizine dihydrochloride + montelukast sodium

12. Azelastine HCL + fluticasone propionate

13. Cromolyn sodium + phenylpropanolamine

14. Carbinoxamine + phenylephrine

15. Carbinoxamine maleate + phenylephrine HCL

16. Phenylephrine HCL+ chlorobutanol + chlorpheniramine

17. Phenylpropanolamine HCL + pheniramine maleate

### 7.2.5.1. Combination of oral antihistamine and leukotriene receptor antagonist

**Keywords:** Allergic rhinitis, anti-allergic agents, histamine H1 antagonists, antihistamines, administration oral, desloratadine, montelukast, montelukast sodium, azelastine, loratadine, cetirizine, pheniramine, chlorpheniramine, cholorpheniramine maleat, leukotriene antagonists, carbinoxamine, carbinoxamine maleate, dexbrompheniramine maleate, pheniramine maleate

### International literature:

Oral antihistamine + leukotriene receptor antagonist combinations are more effective than oral antihistamines alone on rhinorrhea, nasal itching, ocular symptoms, sneezing and daytime symptoms. However, further studies are needed for night symptoms, nasal congestion, ocular symptoms and quality of life (573).

Montelukast is not the first treatment option in AR treatment due to its weak efficacy compared to both oral antihistamines and NCS. However, when combined with oral antihistamines, it provides significant superiority in nasal symptom scores compared to placebo, and shows treatment efficacy equivalent to NCS. Therefore, it is recommended to be combined with oral antihistamines (555).

From the pharmacological point of view, and compared to use of montelukast alone, it has been shown that the standard preparation, in which desloratadine is combined with the usual montelukast dose, has no effect on the bioavailability of montelukast, and can be used safely (574). Combining montelukast and desloratadine provides an additional benefit to therapy particularly in cases with intermittent or mild persistent AR. This combination increases treatment compliance, and offers a more cost-effective alternative in patients with simultaneous asthma and AR (575). The combination of desloratadine and montelukast improves daytime symptoms more effectively than montelukast alone (575). In addition, this combination both objectively and subjectively affects the quality of life positively in perennial AR (575).

Another combination of oral antihistamine and leukotriene receptor antagonist, montelukast + fexofenadine, has been shown to reduce total nasal symptom scores more than the combination of montelukast + levocetirizine, and is a more cost effective option (576).

### National literature:

Oral antihistamines are effective on sneezing, itching, rhinorrhea and ocular symptoms, while LTRAs are less effective on all nasal and ocular symptoms. On the other hand, both medications are not quite effective on nasal congestion, and therefore the use of combination products is advantageous in some respects (489).

Combination of desloratadine + montelukast improved sleep-related symptom scores more than desloratadine alone in patients with AR, therefore desloratadine + montelukast combination is superior to desloratadine alone, particularly for night symptoms (564).

### 7.2.5.2. Combination of oral antihistamine and decongestant

**Keywords:** Allergic rhinitis, anti-allergic agents, histamine H1 antagonists, administration oral, desloratadine, azelastine, loratadine, cetirizine, pheniramine, chlorpheniramine, chlorpheniramine maleate, azatadine maleate pseudoephedrine sulfate drug combination, carbinoxamine, carbinoxamine maleate, dexbrompheniramine maleate, phenylephirine hydrochloride, pheniramine maleate

### International literature:

The combination of cetirizine and pseudoephedrine (cetirizine 10 mg + pseudoephedrine 120 mg) is superior to cetirizine and pseudoephedrine alone in terms of nasal congestion in the first two hours of pollen exposure, and it is more effective than the use of both agents individually in AR treatment (576).

A review including four studies on the combination of desloratadine and pseudoephedrine has reported that the combination was more effective than desloratadine or pseudoephedrine alone in terms of decongestion at the beginning (the second day) of AR treatment, and may be considered in the treatment of AR patients when nasal congestion was the main symptom (577).

A multicenter, randomized, controlled, double-blind study reported that desloratadine + pseudoephedrine combination has more antihistamine and more decongestant activity compared to individual use of these active ingredients, and it was superior to individual use of these two agents not only in nasal congestion, but in all nasal and non-nasal symptoms of seasonal AR. The authors also stated that the adverse effects of this combination was not more than the side effect of the decongestant alone. Therefore, they recommended combined use of desloratadine and pseudoephedrine rather than their individual use in seasonal AR treatment (578).

### 7.2.5.3. Combination of antihistamine and corticosteroid

**Keywords:** Allergic rhinitis, anti-allergic agents, histamine H1 antagonists, administration oral, desloratadine, azelastine, loratadine, cetirizine, pheniramine, chlorpheniramine, cholorpheniramine maleate, carbinoxamine, carbinoxamine maleate, dexbrompheniramine maleate, pheniramine maleate, mometasone furoate, desloratadine, azelastine, loratadine, cetirizine, pheniramine, chlorpheniramine, cholorpheniramine maleate, fluticasone, fluticasone propionate

### International literature:

A randomized controlled clinical trial comparing the combination of oral desloratadine + prednisolone with dexchlorpheniramine maleate-betamethasone in childhood AR concluded that both combinations provided effective treatment, but the desloratadine + prednisolone combination offered fewer side effects and easier dosing (405).

### 7.2.5.4. Combination of nasal corticosteroid and antihistamine

**Keywords:** Allergic rhinitis, anti-allergic agents, histamine H1 antagonists, corticosteroids, antihistamines, nasal sprays, administration intranasal, mometasone furoate, desloratadine, azelastine, loratadine, cetirizine, pheniramine, chlorpheniramine, cholorpheniramine maleate, fluticasone, fluticasone propionate, azatadine maleate pseudoephedrine sulfate drug combination, carbinoxamine, carbinoxamine maleate, dexbrompheniramine maleate, pheniramine maleate

### International literature:

The combination of NCS with nasal antihistamines provides significant improvement in total nasal symptom scores compared to use of NCS and nasal antihistamines alone (411). The combination of azelastine hydrochloride and fluticasone propionate is significantly superior to azelastine or fluticasone propionate alone in the treatment of all nasal and ocular symptoms of AR (457). This combination does not result in any drug-drug interaction, except for a small clinically insignificant increase in the bioavailability of fluticasone (579), and its short and long term use is safe (580).

The use of azelastine + fluticasone combination provides an additional benefit in moderate/severe (579) and persistent (581) AR in adults and adolescents, and in the treatment of both perennial and seasonal AR (582) in all age groups. It is a more effective fast acting treatment option in 4-12 age group (583) and in all age groups (584) compared to fluticasone propionate alone.

Azelastine + fluticasone combination provides efficacy equivalent to sublingual immunotherapy (585) and other current treatment regimens (464) in AR. This combination is more affordable than other NCS and antihistamine combinations as well as use of these two agents alone in AR treatment and when asthma accompanies AR (586). It improves quality of life and eye symptoms significantly more than placebo (587). However, further detailed efficacy, quality of life studies as well as research on children are needed in order to use azelastine + fluticasone or similar NCS + antihistamine combinations in AR treatment (579).

### 7.2.5.5. Combination of nasal corticosteroid and decongestant

**Keywords:** Allergic rhinitis, anti-allergic agents, histamine H1 antagonists, corticosteroids, nasal decongestants, nasal sprays, administration intranasal, mometasone furoate, fluticasone, fluticasone propionate, phenylephirine hydrochloride

### International literature:

The combination of nasal mometasone furoate and oxymetazoline is more effective than nasal mometasone furoate alone in the first 1-4 hours of treatment in terms of decongestion and superior to nasal oxymetazoline alone for the continuance of decongestion. It is recommended in seasonal AR particularly for ensuring rapid onset of treatment (588).

### 7.2.6. Anti-IgE

**Keywords: **Perennial allergic rhinitis, Seasonal allergic rhinitis, Omalizumab, Anti-IgE

Omalizumab is a subcutaneously administered recombinant human monoclonal anti-IgE antibody. It reduces the level of free IgE and prevents binding of IgE to high affinity IgE receptors by binding to the Fc portion of the free-circulating IgE antibodies, hence, it blocks allergic inflammatory reactions. It also reduces the expression of high affinity IgE receptors (FCeRI) on basophils and mast cells. It also reduces the numbers of eosinophils, lymphocytes and other inflammatory cells in the respiratory tissue (589).

There is a meta-analysis on the use of omalizumab in AR. It reported that omalizumab significantly reduced symptom scores and need for use of other agents, and improved quality of life in patients with moderate-severe AR, whose symptoms could not be controlled with conventional treatment (590). In addition, it was reported that omalizumab used together with immunotherapy reduced symptoms scores and the need to use other medications in patients with seasonal AR (591). Starting omalizumab treatment nine weeks before immunotherapy significantly reduced severe side effects and anaphylactic events due to immunotherapy (592). Omalizumab may help tolerance development during immunotherapy by decreasing free serum IgE levels (593). Administration of omalizumab in the first year of immunotherapy reduces the symptom scores and the need to use other medications, however its effect does not persist in the long term (594). Bozkurt et al. (567) reported in their AR model that omalizumab was effective in controlling allergic symptoms and upper / lower airway inflammation.

Current data indicate that omalizumab is considered as a new treatment agent in moderate-severe AR patients who have allergen-specific antibodies and do not respond to conventional pharmacotherapy. In addition, omalizumab may be useful in patients with simultaneous AR and asthma (593). However, the drug is not FDA approved for AR, and it has been claimed that its price is the most important factor in this regard (593).

Omalizumab is usually well-tolerated except for its few serious adverse effects. Local reactions at the injection site, side effects such as viral infections, sinusitis, headache, pharyngitis and rarely urticaria, anaphylaxis and anaphylactoid reactions, thrombocytopenia and alopecia have been reported. In controlled studies on malignancy potential, no difference was found between groups receiving and not receiving omalizumab therapy (595). Cases with Churg Strauss syndrome associated with omalizumab treatment have also been reported (596).

The ARIA group recommends omalizumab in patients with AR and asthma with an obvious IgE-dependent allergic component, and in asthma patients who cannot be treated despite optimal pharmacological therapy and appropriate allergen avoidance. They do not have any recommendations regarding the use of anti-IgE in patients with AR not accompanied by asthma (568).

### 7.2.7. Cromolyns

**Keywords:** Allergic rhinitis, Perennial allergic rhinitis, Seasonal allergic rhinitis, Cromolyn sodium

Cromolyns are mast cell stabilizers that act in acute phase reaction by preventing mast cell degranulation and histamine release (597). They also have anti-inflammatory properties on mast cells, basophils, eosinophils and T-lymphocytes. They have been shown to be effective both in early and late phase allergic reactions (598). They are also called as “mast cell stabilizers”. Cromolyn sodium (sodium cromoglycate) and nedocromil sodium are included in this class of agents. In addition to nasal spray formulations; ophthalmic, pulmonary and oral preparations are also available. Cromolyn sodium (sodium cromoglycate) 4% is marketed as a nasal preparation in Turkey. Cromolyn sodium 4% nasal spray is an effective, safe and well-tolerated preparation recommended in treatment of seasonal AR (599). Its administration 2-3 weeks before the pollen season has been recommended since it acts like a preventive agent in seasonal AR (599).

The reports on the effects of cromolyns include an evidence-based report and a meta-analysis (600, 601). Except for two among 21 randomized controlled studies on seasonal AR, and 14 randomized controlled studies on perennial AR, cromoglycates were found to be more effective than placebo in nasal AR symptoms (600). They are more effective in seasonal AR compared to perennial AR (600). Their efficacy on nasal congestion is less than their effect on other AR symptoms (600). Its effectiveness increases by increasing the dose or the frequency of administration (600). The meta-analysis reported that NCS were more effective than cromolyns in overall assessment and all nasal symptoms, and nasal antihistamines were more effective than cromolyns in overall assessment (601).

The current literature emphasized that use of intranasal 4% sodium cromoglycate spray 4 times/day for 4 weeks improved symptom scores in patients with mild-to-moderate AR, and reduced nasal neutrophilic aggregation and PAF release in nasal secretions (602). In a randomized study comparing nasal disodium cromoglycate with nasal mometasone furoate and levocabastin in seasonal AR, it was reported that mometasone furoate was more effective than levocabastin and cromoglycate in nasal symptom control, and significantly improved nasal inspiratory flow compared to cromoglycate (603). Cromoglycate and azelastine were found to be more effective than placebo in ocular symptoms of the patients with seasonal allergic conjunctivitis (604).

Nasal cromolyns are safe agents, and they can be used safely in children and pregnant women. Nasal cromolyn may be considered as the first-line treatment for AR-related rhinorrhea, sneezing and itching symptoms in pregnant women (605). It can be used safely in pediatric cases at the age of 2 and over (598). However, the need for administration at least 4 times/day due to its short half-life reduces treatment compliance (597). They do not have major adverse effects. Minor side effects such as nasal irritation, headache and nasal congestion have been reported (600). Deveci et al. (606) reported that prolonged use of sodium cromoglycate in healthy rats resulted in rhinitis medicamentosa-like changes in the nasal mucosa, such as squamous metaplasia, loss of cilia and thinning of the epithelium.

According to ARIA criteria, nasal cromolyns are recommended for AR treatment owing to their excellent safety profiles, however nasal antihistamines are recommended over cromolyns since they are more effective, and have a higher patient compliance. Ocular cromolyns may be administered in mild conjunctivitis seen with AR in children or adults, due to their good safety profiles. However, the need to apply 4 times a day may result in a poor patient compliance (568).

### 7.2.8. Decongestants

**Keywords: **Allergic rhinitis, decongestant, systemic, topical

Sympathomimetic amines (such as phenylephrine, pseudoephedrine and phenylproponolamine) and imidazoline derivatives (such as oxymetazoline, xylomethozoline) used as decongestants bind a- adrenergic receptors, and lead to norepinephrine release and hence vasoconstriction and decreased mucosal edema directly (phenylephrine, oxymetazoline) or indirectly (pseudoephedrine). Sympathomimetic amines usually bind to α-1 receptors, while imidazoline derivatives bind more selectively to α-2 receptors. However, both groups include decongestants (pseudoephedrine and oxymetazoline) that can bind non-selectively to α-1 and α-2 receptors (607).

### 7.2.8.1. Systemic decongestants

Oral decongestants exert a sympathomimetic effect by stimulating a-adrenergic receptors and increasing adrenergic activity. In this way, they cause vasoconstriction in the upper respiratory tract, paranasal sinuses and nasal mucosa, and decrease the volume and mucus secretion of edematous mucosal tissues (30, 607).

Regulation of mucosal vascular network and particularly filling and emptying cycles of the cavernous venous plexus are important in the regulation of air flow in the nasal cavity and the feeling of congestion. Venous plexuses are surrounded by adrenergic nerve endings, which offer binding to α- and β-adrenergic receptors, similar to the arterioles accompanying them. β receptors have vasodilator and a receptors have vasoconstrictor capabilities. Pseudoephedrine and phenylephrine which act on α-adrenergic receptors in the nasal mucosa, eliminate nasal congestion by exhibiting vasoconstrictive effects in the nasal vascular structures (608).

Pseudoephedrine and phenylephrine are the most frequently used systemic decongestants due to their sympathomimetic effects. Pseudoephedrine increases noradrenaline release, and has an indirect agonistic effect on peripheral α-1 and cardiac β-adrenergic receptors. Phenylephrine is more selective for α-1 receptors, and has a weaker agonistic effect on α-2 and β receptors. Phenylephrine exerts most of its agonistic effect on α-adrenergic receptors directly, and a small amount of indirect agonistic effect is achieved with a slight increase in noradrenaline release (609).

Phenylproponolamine, on the other hand, is not preferred today due to its serious systemic side effects (risk of cardiovascular adverse effects and hemorrhagic stroke in women) (610, 611).

Prolonged-release tablets may extend the action of oral decongestants on nasal congestion up to 24 hours. Oral decongestants may be used alone or in combination with oral antihistamines (389).

In a randomized placebo-controlled study on patients with AR, the effect of single-dose pseudoephedrine on reducing nasal congestion was significantly higher in the 6-hour observation period compared to placebo and phenylephrine. It was also reported in this study that there was no significant difference between phenylephrine and placebo groups (612). Another study on 539 patients with seasonal AR showed that different doses (10, 20, 30 and 40 mg) of phenylephrine did not have a superiority over placebo in reducing symptomatic nasal congestion (613).

In conclusion, the results of few studies indicated that nasal congestion could be reduced with pseudoephedrine, while phenylephrine was ineffective in patients with AR (30).

The use of systemic decongestants is restricted due to their systemic side effects (psychotropic and cardiovascular effects). Their main known side effects are insomnia, irritability, anorexia, anxiety, tremor, tachycardia and increased blood pressure. Because of these side effects and the concern for their acceptability, it is recommended that oral decongestants should be used short-term in patients with AR, and should not be used in the elderly and in certain patients (patients with coronary artery disease, cerebrovascular disorders, arrhythmia, hypertension, hyperthyroidism, urinary retention or glaucoma) (389, 592, 597). An investigation on the effects of oral decongestants on blood pressure showed that phenylpropanolamine significantly increased both systolic and diastolic pressures without any impact on the heart rate, while pseudoephedrine might cause an increase in systolic blood pressure and heart rate. However, the use of high-dose of pseudoephedrine or rapid-release tablets has been reported to further increase blood pressure (611, 615).

Although it has been shown that the oral decongestants are effective in reducing nasal congestion in children over 6 years of age, prolonged-release formulations at a dose of 120 mg is not recommended in children under 12 years of age. Children under the age of four are more susceptible to toxicity, and the safe dose range has not been defined. In children under two years of age, the central nervous system stimulator effect can lead to psychosis, ataxia and hallucinations. Therefore, systemic decongestants should be administered to the patients under 6 years of age only after assessing the risks and benefits of therapy (389, 608).

### 7.2.8.2. Nasal decongestants

Nasal decongestants lead to vasoconstriction owing to their α-adrenergic stimulating effect on vascular smooth muscles, they also decrease inflammation. Although they improve nasal congestion in patients with AR, they do not have any effect on other symptoms of AR. It has been known that the effect of nasal administration is superior to systemic administration for improving nasal obstruction. Short-term use of nasal decongestants may be recommended particularly in presence of persistent nasal obstruction in patients with AR (389, 492, 616).

Nasal administration of xylometazoline and oxymetazoline provides temporary but rapid elimination of nasal obstruction owing to their strong vasoconstrictive effects. However, long-term topical use results in an increase in symptomatic nasal congestion (rhinitis medicamentosa). The pathophysiology underlying rhinitis medicamentosa is not clearly known, however it is believed that the amount of endogenous norepinephrine in the presynaptic interval decreases with negative feedback due to long-term use of a nasal decongestant. When decongestant is stopped, it is supposed that sympathetic activity decreases due to insufficient norepinephrine, parasympathetic activity becomes dominant, and as a result, “rebound congestion” occurs as a further increase in nasal secretion and vasodilation. In addition, as long-term use of topical decongestants will cause desensitization in α receptors, the patient will need to increase the dose of medication to achieve the same effect (617).

The duration of decongestant use leading to the development of rhinitis medicamentosa is controversial. While some studies have shown that prolonged use up to 8 weeks does not cause rebound nasal congestion, other studies have shown that even 3-day use may result in rhinitis medicamentosa. Largely, it has been accepted that the risk of rhinitis medicamentosa increases significantly when a decongestant is used more than 10 days. The recommended period of administration is less than 3 days (45, 389, 597, 617).

Known side effects of nasal decongestants are nasal burning and tingling sensation, mucosal ulcerations, epistaxis and dryness. Although nasal decongestants have a strong decongestant effect, these side effects may occur due to the rebound nasal congestion and their negative effects on mucociliary activity (389,597).

Some studies showed that the side effects of nasal decongestants (oxymetazoline) such as tachyphylaxis and rebound congestion were reversible when they were combined with NCS. They also claimed that combined nasal decongestant and NCS preparations were more effective than use of NCS alone in the relief of nasal symptoms (586).

### 7.2.8.3. Use of decongestants during pregnancy and in the elderly

The use of decongestants during pregnancy, particularly in the first trimester, may lead to anomalies such as gastroschisis, endocardial cushion defect, ear anomalies and pyloric stenosis. Therefore, their use is not recommended during pregnancy (618).

The use of decongestants is not recommended in the elderly due to their adverse effects on cardiovascular, urinary, central nervous and endocrine systems. They should not be used particularly in those with a history of glaucoma or disorders of cardiovascular, urinary tract, and vascular systems (389, 597, 618).

According to the ARIA Guideline updated in 2016, the American Academy of Otolaryngology – Head and Neck Surgery (AAO-HNS) Guideline, and the International Consensus Report published in 2018, use of systemic pseudoephedrine is a “recommendation”, and the use of phenylephrine is a “counter-recommendation”. The use of intranasal decongestants is reported as “optional / preferential” (96, 389, 417).

### 7.2.9. Anticholinergics

**Keywords:** Allergic rhinitis, Perennial allergic rhinitis, Seasonal allergic rhinitis, Ipratropium.

Anticholinergic agents decrease the parasympathetic stimulation by preventing acetylcholine binding to muscarinic receptors. Nasal anticholinergics prevent secretion of the nasal mucous glands, and provide a reduction in aqueous rhinorrhea. The only nasal spray preparation with anticholinergics contain ipratropium bromide, which is effective in controlling rhinorrhea in both AR and non-allergic rhinitis (619). The nasal form of ipratropium bromide is not marketed in Turkey although nebulized and inhaler forms are in the market.

There are no meta-analyses in the literature on the use of ipratropium bromide in AR. ARIA 2010 revision recommended use of nasal ipratropium bromide for rhinorrhea in perennial AR patients (568). It was reported that combination of nasal beclomethasone and ipratropium was more effective than uncombined use of these agents in the control of rhinorrhea in perennial AR (620). Efficacy of ipratropium bromide was reported to be comparable to the efficacy of nasal beclomethasone for rhinorrhea in school-age children with perennial AR and non-allergic rhinitis. It was reported that ipratropium bromide was also effective in relieving nasal congestion (621). Administration of 42 or 84 micrograms into each nostril, three times a day was found to be easy, safe and beneficial for rhinorrhea related to AR or flu in children between 2-5 years of age (622).

Nasal ipratropium bromide has a fast onset of action, and it is recommended to use it three times a day for maximum effect. It is not effective on nasal congestion, sneezing or itching. Its local side effects include dry nose, irritation, burning, epistaxis, dry mouth, and headache, however systemic anticholinergic adverse effects are rare. On the other hand, it should be used with caution in patients with benign prostatic hypertrophy and narrow-angle glaucoma due to risk of systemic adverse effects (597).

### 7.3. Immunotherapy

**Keywords:** Immunotherapy, mechanism of action, history, indication, contraindication.

### 7.3.1. Introduction

Allergen specific immunotherapy (SIT) is a long-term therapy aiming to reduce symptoms that develop due to allergic AR, allergic conjunctivitis, allergic asthma and insect stings, and it results in permanent relief of symptoms by allergen desensitization after the end of the treatment (623).

Allergens are proteins or glycoproteins that can bind to IgE. Most allergens are natural substances present in the nature, such as pollens, animal hair, molds, insects and food. Immunotherapy regulates the immune system to increase host defense against microorganisms. SIT is a treatment method that alters the IgE-mediated immune response by long-term administration of the allergen extract in subclinical and increasing doses, and it aims to improve the symptoms appearing on exposure to that allergen. SIT aims to increase the quality of life of the patient and prevents the progression of the allergic disease in the long term by providing clinical and immunological tolerance (624). Yılmaz et al. (625) compared the groups treated with SIT and pharmacotherapy, and showed that SIT improved the quality of life and reduced the cost of treatment.

Noon used SIT first in 1911 to vaccinate himself for the pollens that he defined as “aerogenic toxins”. The first randomized controlled study on SIT was performed by Frankland and Agustin in 1954. The serious and even fatal side effects of subcutaneous immunotherapy (SCIT) have led researchers to seek a safer way. Sublingual immunotherapy (SLIT) was developed as a new alternative to SCIT, and was accepted as an alternative method to SCIT in 1998 by the World Health Organization (626).

The patients should provide an informed consent form for SIT due to medicolegal issues. Immunotherapy should be interrupted in the pollen season in patients with seasonal AR. SIT should be discontinued immediately if anaphylaxis develops during treatment (627). The indications of SIT are listed in Table 7.3.1.1. (38), and SIT contraindications are listed in Table 7.3.1.2 (628).

A late phase allergic reaction consisting of eosinophils, basophils, mast cells, T cells and macrophage infiltration occurs 6-12 hours after exposure to the allergen. The inflammatory process continues with the release of inflammatory cytokines and mediators from these cells (625). SIT reduces the hypersensitivity of the end organ by altering the humoral and cellular response to the allergen. It prevents early and late phase allergic reactions. As the SIT continues, the immune response slides from Th2 to Th1. Specific IgE levels increase at the beginning of SIT, but then they gradually decrease. The levels of specific IgG1, G4 and IgA antibodies increase, but these increases are not proportional to clinical improvement. The affinity of the IgG to the allergen rather than the level of IgG is more correlated with clinical improvement (629).

The patient compliance is the most important problem in the selection of patients for SIT.

### 7.3.2. Informing the patient

### 7.3.2.1. Treatment process

As shown in the recent systematic reviews, the nasal and ocular symptoms improved, and the need for medication decreased with SIT (630-634).

Current Guideline on AR and asthma have reported that SIT is particularly indicated for the treatment of moderate to severe intermittent or persistent AR symptoms, which respond poorly to pharmacotherapy. The allergen extracts for SIT include pollens of grasses, trees and weeds; house dust mites, molds and animal skins. However, given the effectiveness and reliability of SIT directly depends on the quality of the extracts, the use of standardized extracts is an important point in clinical practice (423, 568, 635).

There are different methods for the treatment of allergic asthma and AR / conjunctivitis. In fact, each of the three main treatment methods, namely SIT, avoidance of allergen and pharmacotherapy, have their own benefits, risks, and costs. The severity, duration, and need for medical treatment of symptoms should be taken into account. In addition, the treatment must be tailored individually for each patient, considering his/her preferences. The severity of the disease and the response to previous treatments are also important in this planning (629).

In addition, appearance of the side effects of pharmacotherapy is an indication for SIT, as well as the patients who want to reduce or discontinue long-term treatment (629).

Treatment is recommended to be started early, as the effectiveness of SIT against asthma is higher in children and young adults. Moreover, unlike pharmacotherapy, in which treatment is continued uninterruptedly in order to have a symptomatic well-being, the fact that the clinical benefit continues for 3-5 years after SIT is terminated makes this treatment more advantageous in young patients (636-638). Appropriate allergen extracts must be chosen by an educated and experienced physician in the light of the history of allergen exposure and symptoms of the patients.

The initial dose of immunotherapy, target maintenance dose and immunotherapy schedule should be determined by the physician. SIT treatment may be divided into two phases, as the initial phase and the maintenance phase. In the initial phase of treatment, increasing amounts of allergen extract are given in the first 8-28 weeks of treatment. In traditional SIT calendars, one dose increase is made for each visit, and the frequency of the visits varies 1-3 per week.

SIT injections can cause local and systemic reactions. Severe reactions often develop within 30 minutes after injection. In addition, systemic reactions associated with immunotherapy may occur later than 30 minutes. Patients should be informed about early and late systemic reactions and how to proceed when they develop before beginning SIT.

Local reactions can be managed with local treatments such as cold application or topical corticosteroids, or with systemic antihistamines. Systemic reactions can be moderate or severe. Epinephrine is the first treatment option in patients with anaphylaxis. Antihistamines or systemic corticosteroids are the secondary medications for controlling systemic reactions, and they can never replace epinephrine in the treatment of anaphylaxis. In severe cases, intravenous fluids and oxygen supplementation may be necessary. The immunotherapy dose, schedule, and risks / benefits of continuing treatment should be reassessed in cases who had systemic reactions.

### 7.3.2.2. The results of treatment

Different tests and bioparameters are used to evaluate the clinical efficacy of SIT. Two criteria are taken into consideration when analyzing the clinical results, namely the scoring done by the patient and the scoring done by the physician (639, 640).

There are primary and secondary outcome parameters to evaluate the clinical outcomes of SIT treatment. The severity of symptoms and the need for simultaneous medical treatment are the primary outcome parameters. Specific and general quality of life scores and cost effectiveness are the secondary outcome parameters. A number of additional evaluation methods including cytokine analysis, and cell activation or proliferation markers have been used to comprehend the immunological mechanism of SIT or to demonstrate its therapeutic efficacy.

The World Allergy Organization (WAO) stated that both symptom scoring and scoring for the need of medical treatment should be done (641).

Each symptom should be specified daily on a 4-point scale: 0: No symptoms, 1: Mild symptoms (slightly aware of symptoms, they are easily tolerated) 2: Moderate symptoms (symptoms are clearly noticed, they are very disturbing but tolerable) 3: Severe (symptoms are difficult to tolerate, affecting daily activities and sleep). This scoring method has been approved by authorized institutions in the USA (FDA) and Europe (EMA).

### 7.3.2.3. Cessation of treatment

Although there are different opinions in the literature regarding the duration of treatment, it is usually accepted that the treatment should be continued for at least 3-5 years. However, further extension of this period provides a longer symptomatic control (642-644). Despite known clinical benefits of SIT treatment, the obstacles encountered in the treatment process are classified under three main headings: The first is the risks brought by the treatment, the second is the cost of the treatment, and finally the patient’s incompliance with therapy.

### 7.3.2.4. Immunotherapy in the future

Considering the known side effects of SIT, allergens have been developed to produce an immune response without triggering the pathways that cause allergic symptoms or classical side effects of SIT. Peptide immunotherapy uses soluble synthetic peptides that are recognized by T cells, and unlike conventional allergen extracts, they are standardized. Peptide immunotherapy has shown promising results in the treatment of cat, bee venom, house dust mite and grass pollen allergy. Peptide immunotherapy performed for 3 months with synthetic peptide T cell epitopes of cat allergen reduced the symptoms of rhinoconjunctivitis in 2 years following the termination of treatment (645, 646).

Recombinant allergens are commercially produced purified proteins, and they have high safety and efficacy. These genetically modified or natural phenotype allergens reduce IgE-related side effects of SIT, and can provide long-term immunity (647).

Immunostimulant adjuvants are therapeutic agents that aim to increase the immunogenic properties of certain specific allergens without pharmacologically activating them. They have been used in vaccines for many years, however the idea of using them in SIT was introduced later.

In genetic vaccines, plasmid DNA or mRNAs that carry the genetic information of the allergen is used instead of the allergen itself. The genetic material is taken up by local host cells located in the dermis, and converted into protein in vivo. These allergenic proteins produce a T1-mediated antigen-specific CD4 and CD8 T cell response. Toll-like receptor-mediated innate immune response is generated by the genetic material itself (648).

Allergens are coated with recyclable nanoparticles. They allow allergen release in vivo in a delayed-continuous or pulsatile form. They can modulate the immune response with fewer side effects. It was demonstrated that recombinant birch pollen coated with PLGA (polylactic-co-glycolic acid) nanoparticles regulated the ongoing T2 cell response after a single dose (649, 650).

**Keywords:** Allergen immunotherapy, treatment effectiveness, treatment outcome, treatment, withholding, treatment cessation

### 7.3.3. Subcutaneous immunotherapy

### 7.3.3.1. Initiation of treatment and the dose scheme

### International publications

The widely accepted approach in immunotherapy is subcutaneous injection.

SCIT is regarded as the gold standard in studies. SCIT is recommended for patients who have positive skin tests and do not get sufficient benefit despite maximal medical therapy, or cannot tolerate pharmacotherapy as well as for patients with AR and asthma, who do not want to use pharmacotherapy for a long time (651).

The effective treatment dose or maintenance dose is the maximum tolerable dose not causing severe local or systemic side effects. SIT is continued in the form of regular injections for 3-5 years after reaching the maximum tolerable maintenance dose. The dosage schedule differs according to conventional, cluster, rapid protocols and the allergens (651).

After starting treatment in conventional SCIT, the maximum concentration is reached with weekly injections, then the maintenance dose is administered at 4-8 week intervals. The maintenance dose may be achieved with 8 injections in 3 days (652).

Every company in Europe has its own standards. The standard is determined by the reactivity in the skin test. The maximum tolerable maintenance dose is determined based on the major allergen content of the vaccine (652).

In conventional immunotherapy, subcutaneous injections in weekly increasing doses reaches to a maximum level in 3-4 months (50000-100000 SQ-U / ml), then injections are done monthly. Tables 7.3.3.1.1. and 7.3.3.1.2. show the conventional treatment scheme.

Vaccines should be stored in the refrigerator; as high temperatures may reduce the activity of the allergen. They must be transported in a cold chain (652).

It is necessary to make sure that the names and doses of the allergen extracts contained in the bottles are clearly and correctly labeled. The label on each bottle should contain the name, surname, date of birth and identification number of the patient.

### National publications

SIT is indicated for AR, asthma, and venom, pollen, house dust mite, and cat-dog allergies (653).

Pollen (weed, tree, grass), house dust mites (*Dermatophagoides pteronyssinus, Dermatophagoides farinae*), bee venom, cat, dog and mold allergen extracts are used in SCIT. The extracts may contain only one allergen, or multiple allergens. However, attention should be paid to the interaction of allergens with each other. Some allergens may reduce the effect of others (654).

Immunotherapy has an initial phase and a maintenance phase. In initial phase, the first dose is injected and the dose is increased at certain intervals. The injections are performed at a certain dose for 3-5 years in the maintenance phase (655).

Various companies have allergen extracts for immunotherapy. Their initial and maintenance doses differ. Table 7.3.3.1.3. shows the names and treatment doses of some companies (656).

Allergens can be aqueous (aqueous), depot (aluminum, calcium), allergoid, polymerized, with glycerin or lyophilized. The preparation method of extracts affects the efficacy and reliability of SCIT. Side effects are less in treatment with standardized allergen extracts (654).

Allergoid extracts are created by modifying allergens with gluteraldehyde or formaldehyde, and allow administration of high allergen doses. Allergovit (Allergopharma KG, Reinbek, Germany) is used in a scientific research project by Mısırlıgil et al. (647) in 2012. It was emphasized that fewer injections were required compared to conventional SCIT.

Vaccines should be stored between +2 and +8 degrees, on the door of the refrigerator (657). In the presence of active, febrile disease, the injection should be postponed.

**Key words: **Rhinitis, allergic, subcutaneous, injection, immunotherapy.

### 7.3.3.2. Maintenance and the dose scheme

### International publications

The maintenance dose is the dose injected at the maximum concentration that the patient can tolerate. Maintenance doses used by different companies are shown in Table 7.3.3.2.1. (658).

In SCIT’s cluster scheme, weekly or biweekly injections are performed.

### When is the treatment schedule changed?

The scheme is not changed for delays up to 7 days. For delays 8- 13 days, the previous dose is repeated.

For delays 14 -21 days, the previous dose is reduced by 25%.

For delays 21-28 days, the previous dose is reduced by 50%. Then, the dose increase is continued according to the previous scheme at each visit until the ideal dose is reached.

It has been stated that injections were made on the 7^th^, 14^th^ and 21^st^ days after the maintenance dose is reached. This dose scheme was not based either on retrostpecitve or prospective studies, and a patient-based approach may be an option to be followed (629).

The maintainance dose is reached after months in conventional subcutaneous immunotherapy protocol, in days in rush protocol, and in hours in ultra-rush protocol. Paşaoğlu et al. (659) reported that they achieved the maintainance dose in seven days in their rush subcutaneous immunotherapy protocol. The authors started injection of aqueous venom immunotherapy extract (VIT) at a dose of 100 standard quality units (SQ-U)/ml, without any premedication. They preferred a low initial dose (10 SQ-U/ml ) in high-risk patients, and increased dose to 100 000 SQ-U/ml with injections made at 30-minute intervals. It is evident that 14 injections were performed (ALK-Lyophilisate Aqueous SQ 801 and 802, Abelló). Repeat injections were made on days 7, 14 and 21 after reaching the maintainance dose. This study has proven the efficacy of subcutaneous immunotherapy.

### National publications

Maintenance therapy is the highest tolerable therapeutic dose which provides high clinical efficacy with few side effects, and it is continued for 3-5 years.

Conventional (classical), cluster, rush or ultrarush protocols may be employed for subcutaneous immunotherapy. It takes 4-6 months (1-2 injections per week) in the classical protocol, 1 month (2-3 injections per visit, 1-2 visits per week) in cluster protocol, days in rush protocol and 3-4 hours in the ultrarush protocol to reach the maintainance dose.

Maintenance dose schedule varies in different protocols and studies. Low dose is ineffective, and it is not recommended as it may increase the hypersensitivity of the patient (655).

Dose adjustment should be made when switching to a new concentration and bottle, or if patients with a high sensitivity are symptomatic during the pollen season, or if a reaction develops after injection. In these cases, the injections are interrupted (655).

Keleş et al. (660) studied the efficacy of classical subcutaneous immunotherapy on allergic rhinitis. They injected grass pollen and Dermatophagoides pteronyssinus extracts, and induction period was 18 weeks, starting with weekly injections followed by injections every 2 weeks. After reaching the maintainance dose, the patients were injected monthly for 12 months. This study shows that subcutaneous immunotherapy is effective on patients with allergic rhinitis, if the patients are selected in accordance with the correct indication of the treatment.

Polosa et al. (661) used a subcutaneous cluster induction protocol and later monthly maintenance injections with P. Judaica extract (Alutard SQ, ALK-Abello, Milan, Italy) to perform a prospective randomized placebo-controlled study. In this study, the placebo injections contained 0.01 mg/ml histamine in an allergen solution. It was reported that 80 000 standard quality units (SQ-U) were given to the study group as monthly maintenance doses between December 1997 and February 1998, and until 2000, and 80 000 SQ-U was equivalent to 8000 biological units and contained 4.8 µg of allergens. This study demonstrated a significant difference in symptom scores of cluster subcutaneous immunotherapy versus placebo.

In classical subcutaneous immunotherapy, the allergen is injected in increasing concentrations over 8-16 weeks and is continued for 3-5 years, which is the conventional duration for immunotherapy. Since SIT requires frequent and regular injections and the injections are recommended to be performed in a hospital, it is troublesome for the patients. The scientific research project by Mısırlıgil et al. has shown that the use of allergoids (depot preparations modified with aluminum hydroxide or other adjuvants) allows a reduction in the frequency of injections, and does not require a maintenance dose schedule. In this study, the meadow pollen allergoid and placebo groups were compared. Treatment started with pre-seasonal injections and the maintenance dose was reached in 7 weeks, the induction phase was shorter, dose increase was done in accordance with the individual tolerance of the patient, and maintenance doses were not administered (657).

### Adverse effects

### 7.3.3.3. Adverse effects

### International publications

Side effects related to injections are classified into local and systemic reactions. Indurations larger than 5 cm are considered as a local reaction. Systemic reactions are divided into 4 grades (662).

Common skin lesions are seen in grade 1, including redness, itching and urticaria. In grade 2, in addition to the grade 1 reactions, the mucosa is affected; rhinoconjunctivitis, itching in mouth and angioedema are seen. In grade 3, mild or moderate asthma occurs in addition to the symptoms in grade 2. In grade 4, severe urticaria and asthma, hypotension, weakness, dizziness, abdominal pain, nausea and vomiting are observed. When classified in relation with the occurrence time of the reaction, the reaction is called as an “early reaction” if it occurs within the first 30 minutes after injection, and as a “late reaction” if it occurs later than 30 minutes. There is also a difference in relation with the protocols applied.

Side effects may vary depending on the type of allergen extract, injection schedule, allergen preparation and dosage (663).

The patients must be questioned for use of beta blockers or ACE inhibitors, history of asthma attacks before SCIT injections, and their lung capacity should be evaluated with a respiratory function test before starting treatment. Accompanying uncontrolled asthma or cardiovascular diseases (unstable angina history, recent myocardial infraction, arrhythmia and uncontrolled hypertension) are contraindications for SCIT. Therefore, every patient should be evaluated in terms of asthma before SCIT. The use of beta blockers or ACE inhibitors poses a risk to patients. SCIT is contraindicated in patients who had severe systemic reactions (632).

Given that the vast majority of systemic reactions develop within the first 30 minutes following injection, patients should be observed for at least 30 minutes (632).

Injections should be performed by trained personnel, after the necessary equipment is provided, due to the risk of systemic reactions and anaphylaxis. Side effects should be well monitored, and physical examination results and vital signs should be recorded regularly.

In the international evaluation report, a death related to SCIT injection has been reported. The mortality rate was calculated as 1-2 in approximately 2.5 million SCIT injections. The presence of uncontrolled and symptomatic asthma and the presence of a big positive skin reaction on prick test have been blamed for the appearance of systemic reactions. Increasing the dose in the pollen season also increases the risk for systemic reactions. Controlling asthma symptoms before injection largely reduces deaths (664).

Polysensitized patients are at higher risk than monosensitized ones. Systemic reactions mostly occur within the first 30 minutes after injection. However, polysensitized asthmatic patients are more likely to have late reactions. Therefore, these patients should be monitored for more than 30 minutes, for at least 1 hour (665).

### National publications

Local and systemic side effects may be seen after SCIT. According to the Ring and Messmer classification, side effects are divided into four grades. Due to the risk of fatal side effects, SCIT has been recommended to be applied and followed up by experts in centers with emergency intervention opportunities. Incorrect dose adjustment, accompanying signs of uncontrolled asthma, and use of beta blockers and ACE inhibitors increases the frequency of systemic reactions. Therefore, the current medications of the patient should be questioned before the injection, asthma findings should be examined, and respiratory functions should be evaluated with PEF or FEV1 (655).

The most common side effects are local reactions. Studies have shown that small-sized local reactions are asymptomatic, and do not interfere with continuing treatment. It has been emphasized that the dose should not be increased when a local reaction sizing 2.5-3 cm occurs, and the dose should be reduced when a 3-5 cm or larger local reaction is seen.

In light of this information, it is concluded that SCIT can be used safely in both adults and children.

**Key words:** Immunotherapy, allergic rhinitis, elderly, child, systemic reaction, safety.

### 7.3.4. Sublingual immunotherapy

SIT is effective in improving symptoms, reduces the use of medications for symptom control, and is the only intervention that can change the course of the allergic process (666). SIT has been used for many years. Subcutaneous immunotherapy (SCIT) and sublingual immunotherapy (SLIT) are used most frequently today (667). Although the effectiveness of both methods is similar, there are differences in route of administration, frequency and dosage. The route of SIT must be decided by the physician and the patient together, considering the patient’s characteristics. Immunotherapy is applied in two phases, as initial and maintenance phases (668).

SIT has been practiced for over a hundred years. However, upon coming across with the serious side effects and even death, safer administration routes have been sought in SIT, including oral, nasal, bronchial, lymphatic and sublingual routes. SLIT was proposed as an alternative to SCIT in the 1980s, and significant progress has been achieved over the past 30 years (669). The first randomized, double-blind, placebo-controlled study with SLIT was published in 1986, followed by many other reports that confirmed its effectiveness, albeit in small patient populations (670). SLIT was first published by the World Health Organization in 1998, and later published in the Guideline of ARIA in 2001 and 2008, and “World Allergy Organization” Guideline in 2009 and 2013 (671).

SLIT comprises ingestion of allergen-containing extracts after keeping the drop or tablet form under the tongue for a few minutes. In the initial phase, allergens are administered at certain intervals in a solution, at doses that are too small to produce an allergic response. The dose of allergen is gradually increased until switching to the maintenance dose, once a week for several months in SCIT. The maintenance phase is reached when the highest and most effective dose is reached; this is the dose that the patient can tolerate, and does not cause any systemic reactions. The treatment period is minimum 3, and maximum 5 years. In SLIT, while the allergen is applied initially every day, it is applied 3 days a week after passing into the maintenance phase. SCIT requires monthly doctor visits, but not daily dosing. In SLIT, the patient can administer treatment at home after the initial dose. SLIT is generally considered to have a better safety profile than SCIT. The main difference between the two routes of administration is that the dose in SLIT contains at least 50-100 times more allergens compared to SCIT, because low doses are generally ineffective in SLIT to achieve a similar level of efficacy with SCIT (672-674). The advantages of SLIT are ease of application, few side effects, no fatal complications, no need for injections and no need for hospital visits for taking the treatment.

Constant exposure of the oral mucosa to microorganisms or foreign bodies does not result in any infection or inflammation, and this was the starting point of SLIT administration (675-677). Handling and presentation of allergen is very important in the T cell response in SIT (678). It has been supposed that Langerhans-like dendritic cells, which are densely situated under the tongue, carry the allergen into the regional lymph nodes, and lead to development of the immunological response by stimulating the type of T cells that suppress the allergic response (677-679). In the early period of treatment, sublingual dendritic cells secrete IL-10 that inhibits the inflammatory response and induces T cells (675, 680).

With continued treatment, there is a decrease in mast cell sensitivity, decreased IgE secretion from mucosal B cells, and an increase in antigen-specific IgG. Sublingual Langerhans cells transform allergen-specific Th2 cells into Th1 cells, increase lymphocyte tolerance to the allergen, thereby creating immunomodulation (681).

SLIT may be a treatment option in serum IgE positive, prick test positive patients with a clinical response to the allergen, the patients with moderate or severe symptoms, the patients with symptoms that cannot be controlled with pharmacotherapy, the patients who cannot receive medical treatment due to their side effects, the patients who do not want SCIT, and the patients who do not want to use medical treatment for a long time.

SLIT is contraindicated in patients with serious cardiovascular or immunological disorders, in the presence of uncontrolled asthma, malignancies, beta-blocker use, during pregnancy (for beginning the treatment), chronic oral mucous diseases and acute infection (675, 682). Immunotherapy is not recommended for patients with compliance problems, particularly the children under 5 years of age (683).

### Application

Sublingual immunotherapy applications consist of initiation (dose increase) (10 IR / mg) and maintenance phases (300 IR / mg).

### 7.3.4.1. Initiation of treatment and the dose scheme

SLIT commercially is available in drop or tablet forms. There are ready-to-use starter and maintenance sets in their special boxes. In the initial treatment set, there are 3 or 4 allergen extracts with increasing concentrations in each bottle. These are called bottles 1, 2, 3 and 4, respectively. In some products, the starter set starts with number 0. Each numbered bottle is a 1:10 diluted version of the next bottle (eg. number 1 is 1:10 diluted form of number 2). The application starts as 1 drop from the bottle 1, containing the smallest concentration of allergens in the treatment set. The patient should drip the drop under the tongue, wait a few minutes, and then swallow it. Treatment is continued by increasing 1 drop each day, and 28 drops are reached on the 28th day. The next day, treatment is continued from the bottle no. 2, at a dose of 1 drop. Again, 28 drops are reached by increasing the number of drops each day. This is repeated for bottles 3 and 4. The patient reaches the maximum dose at the end of the starter set, and this is the maintenance dose (684).

### 7.3.4.2. Maintenance and the dose scheme

The maintenance treatment set contains the bottle with the highest concentration, bottle 3 or 4, used in the initial treatment. After reaching the maximum dose, the dose is not increased in the maintenance phase, it is kept the same, and is applied 3 days a week.

Treatment should be planned for at least 3 years. The success of the treatment depends on well-informing the patient, a good follow up, and the patient’s compliance. The patient should be scheduled for regular follow-ups. Although there may be individual differences, the symptoms usually disappear completely at the end of the treatment, while there may be little response or even no benefit from the treatment in some patients. It may take 6 months to 1 year to have a clinical response. SLIT should be stopped if there is no reduction in symptoms at the end of the first year. Investigations have shown that 3-5 years of immunotherapy provides long-term efficacy in patients with AR. Immunotherapy has been shown to be protective for the development of asthma in the future in patients with AR.

### 7.3.4.3. Adverse effects

Side effects often occur at the beginning of treatment. Local side effects occur in the oral mucosa, and they constitute the majority of side effects (75%). Itching or swelling, sore throat, and burning in the throat may be seen. Systemic reactions include gastrointestinal symptoms such as nausea, abdominal pain, diarrhea, rhinoconjunctivitis, very rarely generalized urticaria, and anaphylaxis. No deaths have been reported due to SLIT (685-687). Some anaphylaxis cases due to SLIT have been reported in the literature (688-690). The first dose must be administered in the hospital.

**Key words:** Allergic rhinitis, allergic rhinitis treatment, sublingual immunotherapy, immunotherapy.

### 7.3.5. Oral immunotherapy

One of the important developments in AR treatment in recent years is the introduction of oral SLIT tablets. These tablets, which are used daily, contain allergens that dissolve rapidly under the tongue.

Two SLIT tablet brands have been approved for grass allergy in our country, Europe and USA. These are meadow grass (timothy grass) SLIT tablet (GRASTEK ® / GRAZAX ® , Merck & Co., Inc., Kenilworth, NJ, USA / ALK, Hørsholm, Denmark), and 5 grass pollen SLIT tablets (ORALAIR ® , Stallergenes SA, Antony, France / Greer Laboratories, Inc., Lenoir, NC, USA). Other SLIT tablets, namely ragweed SLIT tablet (RAGWITEK ®, Merck & Co., Inc., Kenilworth, NJ, USA / ALK) and house dust mite SLIT tablets (ODACTRA ® / ACARIZAX ® / MITICUR to ® , Merck & Co., Inc., Kenilworth, NJ, USA / ALK / Torii Pharmaceutical Co., Ltd., Tokyo, Japan) still has no approval for use in our country.

### 7.3.5.1. Initiation of treatment and the dose scheme

Five grass pollen SLIT tablets are available in two forms: 150 and 300 IR. Adults use 300 IR tablets once a day, for the specified period (691) (Table 7.3.5.1.1).

For meadow and ragwort SLIT tablets, it is recommended to start treatment at least 12 weeks before the beginning of the pollen season. This period is 4 months for five grass pollen SLIT tablets. House dust mite SLIT tablets can be started at any time of the year. If pollen SLIT tablets are started during the pollen season, the patient should be informed that symptoms may increase and medical treatment may not be sufficient for symptom control (692).

### 7.3.5.1. Maintenance treatment

Disease modification effects of SLIT tablets were investigated in two long-term studies. In the first of them, a significant improvement was observed in the AR symptom scores after 3 years of use of the meadow grass SLIT tablet, and in 2 years after the cessation of therapy. With this study, meadow grass SLIT tablet was approved for long-term use in America and Europe (693). Similar results were obtained in another study with the same duration of use and monitoring of five grass pollen SLIT tablets. However, this study failed to meet the criteria for approval for long-term use (694).

### 7.3.5.3. Adverse effects

The most common side effect of grass pollen SLIT tablets are mild to moderate oral reactions (mainly itching in the mouth, mucosal swelling, irritation of the throat). These reactions usually appear with the first dose, and regress within 14 days and do not require treatment. Similar side effects have been observed with other SLIT tablets (695). Mild gastrointestinal symptoms are also frequent (696). In case of severe and persistent gastrointestinal complaints, treatment should be discontinued, since eosinophilic esophagitis cases associated with SLIT use have been reported (697). Severe, uncontrolled and unstable asthma is another contraindication (698).

**Keywords:** Immunotherapy, sublingual immunotherapy, tablets, side effects, dosage.

### 7.3.6. Informed consent form for immunotherapy

**PATIENT’S NAME - SURNAME:**

**GENDER:**

**DATE OF BIRTH:**

**REGISTRATION NO**

**ID NUMBER**

Dear Sir or Madam,

This written form has been prepared to explain the basic information about allergy vaccination treatment and its complications.

This form contains the written form of the information that is verbally explained to you, and will be stored in the hospital archives FOR USE IF A LEGAL REQUIREMENT OCCURS.

As a result of the examination, it was decided that you / your patient should receive vaccine treatment. Your physician will give you the information written in this document before vaccination treatment, and you will finally make your decision on vaccine treatment on your FREE WILL.

It is a LEGAL REQUIREMENT to sign EVERY PAGE of this form, by you and a relative of yours.

**1. Information about your disease:**

You have been diagnosed with allergic rhinitis after your examinations and tests. The vaccine treatment that will be applied to you can be in the form of injection into your arm, or in the form of drops or tablets taken by mouth.

**2. Who should be given the vaccine treatment, where, how and what should be considered:**

If vaccine treatment is administered in the form of injections, it will be administered to you by your physician or a nurse trained on allergy vaccines. Following the application of allergy vaccine, one or more of the following reactions can be seen; itching in the eyes, nose or throat, nasal congestion, runny nose, difficulty of breathing, cough, wheezing, dizziness, nausea, vomiting, rash, itching and shock. These reactions may rarely be severe or even fatal.

If the vaccine is administered orally as a drop or tablet, mild itching or discomfort may be felt in the mouth. This complaint will usually go away spontaneously without treatment. Very rarely itching in the eyes, nose or throat, nasal congestion, runny nose, difficulty breathing, cough, wheezing, dizziness, nausea, vomiting, rash, itching and shock may appear. These reactions may rarely be severe or even fatal.

After applying the allergy vaccine to you, you should wait in the hospital for at least 30 minutes. If you are under the age of 17, your parent or an adult with your legal responsibility should wait with you.

**APPROVAL OF THE PATIENT, PARENTS OR GUARDIAN**

I am in my right mind and I believe I have the competency to make a decision. My doctor has made the necessary explanations about my condition. My doctor answered all my questions in a way that I can understand. I was informed about the side effects that may occur during vaccine treatment. In the event of a reaction during my treatment, I allow the healthcare personnel who apply my treatment to make any intervention.

**PATIENT, PARENTS OR GUARDIAN**

**Name Surname:**

**Identification number:**

**Signature:**

**Date:**

**PATIENT’S RELATIVE or GUARDIAN’S RELATIVE**

**Name Surname:**

**Identification number:**

**Signature:**

**Date:**

**THE PHYSICIAN WHO OBTAINED THE INFORMED CONSENT**

**Stamp:**

**Signature:**

**Date:**

### 7.3.7. Comparison of immunotherapy methods

SIT is the only treatment method that can change the course of AR. When SIT was first used, it was administered subcutaneously (636). However, the rare but fatal systemic side effects of SCIT have led to the development of the SLIT method (699). Later, the SLIT method took its place in the official documents and Guideline (700). Preparations of SLIT include liquid extracts, sublingual tablets and oral mucosal immunotherapy (OMIT). SLIT delivers the allergenic protein into the sublingual space. In OMIT, allergic protein extracts are delivered to the gingival, vestibular, buccal and sublingual mucosa with a toothpaste (701). It has been determined that OMIT is as effective as SLIT, and they are similar in terms of their side effect profiles (701).

It was concluded that it is highly effective in seasonal AR treatment and reasonably effective in the treatment of perennial AR due to mite hypersensitivity (702).

It has been determined that uninterrupted long-term SCIT and SLIT provide clinical benefit. Both methods were found to be effective in seasonal and perennial AR treatment in reducing AR symptoms, however symptom control is better in seasonal AR. Its efficacy has been shown to be better in adults compared to children (693, 703).

**Key words: **Allergen specific immunotherapy, subcutaneous, sublingual, seasonal, perineal, allergic rhinitis.

### 7.3.8. Algorithm

1. Immunotherapy is effective in the treatment of AR patients.

2. Prick test is preferred. In some cases, it is useful to test specific IgE.

3. Immunotherapy should not be given to those with negative specific IgE tests.

4. The patient should be evaluated in terms of different treatment options, and the treatment of the patient should be individualized.

5. An informed consent should be obtained in terms of risks, benefits and cost of treatment, and the initial dose should be planned.

6. Immunotherapy should be applied in places where its possible side effects can be treated. Treatment may be terminated if local or systemic side effects or complications appear.

7. Maintenance therapy should be planned.

8. Remission of disease is indicated by: No symptoms in the previous year, allergen prick test results as well as histamine reaction gets smaller by 50% compared to pretreatment, decrease in specific IgE levels, and a specific IgG level between class 3-5.

### 7.3.9. Conclusion

Otorhinolaryngologists are responsible for the diagnosis, medical treatment and immunotherapy of AR.

Immunotherapy methods are applied to selected patients who have clinically compatible AR symptoms and have Type 1 hypersensitivity reaction proven with the positive prick test and / or specific IgE.

Otorhinolaryngologists are not responsible for the diagnosis of allergic conjunctivitis, asthma, dermatitis, which may be comorbid with AR. Again, they are not responsible for the treatment of these diseases with immunotherapy methods.

## 8. Special conditions in treatment of allergic rhinitis

### 8.1. Treatment of allergic rhinitis in special conditions

### 8.1.1. Treatment of allergic rhinitis in children

**Method: **A** l**iterature search was conducted in Pubmed, Scopus, Google academic databases with the keywords “children, childhood, allergic rhinitis, treatment”. The meta-analyses were primarily reviewed until 2015. All international publications between 2015-2018 were included in the study. Older publications were used when sufficient data were not available in that period. Priority was given to meta-analyses and randomized controlled trials while making propositions. Other studies, expert committee reports and opinions of respected authorities have been used in the topics without any sufficient data in the literature.

**Keywords: **Rhinitis, allergy, pediatric, treatment

### 8.1.1.1. Epidemiology and risk factors

AR is one of the most frequent chronic diseases in the childhood. Although the prevalence of AR varies in relation with the country and age, AR prevalence was found as 8.5% in children aged 6-7 years, and as 14.6% in children aged 13-14 years in the International Study of Asthma and Allergies in Childhood (ISAAC) study. In that study, the prevalence of AR was reported higher in Pacific Coast countries such as Australia, New Zealand, Korea, while lower prevalences were reported in Eastern Europe, and Central and South Asia (704). The epidemiological data on AR is not satisfactory in our country, and it has been reported as 3-44%. The possible cause for this wide range may be the regional differences in different geographical regions of Turkey as well as the diverse diagnostic methods used in the studies (questionnaire, doctor’s examination, allergy test etc.). AR prevalence was reported higher in the Western regions and in the city centers in our country (653).

Allergic rhinitis in childhood is a step of allergic march. Classically, atopic march begins with atopic dermatitis, and progresses to IgE-mediated food allergy, asthma, and AR (705). On the contrary of this classical knowledge, it has been known that all patients with atopic dermatitis do not develop asthma later in life, or all patients with asthma did not have atopic dermatitis in the past. In their retrospective cohort analysis, Belgra et al. (706) reported that 10.5% of their patients had the classical pattern of the allergic march, and atopic dermatitis persisted and any other allergic disorders did not develop in 15.5% of their patients. Asthma symptoms were observed in 5.7%, and AR symptoms appeared in 9.6% of the patients without any history of atopic dermatitis.

Although AR may be seen at any age, it is very rare to encounter AR in children under two years of age, since two or more seasons are required to develop hypersensitivity to aeroallergens. AR is usually evident after 3 years of age in children, and its prevalence increases with increasing age. In a prospective study on this subject, the prevalence of AR was found as 5% in children 4 years of age, and 14% when children got 8 years old. In fact, it has been shown that symptoms occur before the age of 20 in 80% of patients with AR (707).

Established risk factors for AR are positive family history, male gender, being the first child of the family, early systemic antibiotic use, maternal smoking, exposure to indoor allergens, a serum IgE level above 100 IU / mL before 6 years of age, and presence of allergen-specific IgE. The children with food allergy or atopic dermatitis in early childhood have a high incidence of AR and asthma in the older ages. In addition, food allergy in infancy is an independent risk factor for development of AR and asthma (30, 105, 708). Alduraywish et al. (118). performed a meta-analysis, and stated that children with food allergy in the first two years of life had a high risk for AR and asthma in later years. This relationship is quite evident for major food allergens including peanuts, milk and eggs. In addition, it was stated that the risk of asthma and allergic disorders were higher in children who develop hypersensitivity to more than one food compared to children who develop hypersensitivity to a single food (705).

Epidemiological studies performed in the previous decades revealed an increase in the prevalence and severity of allergic disorders. The results of these investigations paved way for the studies on the possible roles of predisposing factors on increased prevalence, such as diet, hygiene, infectious diseases, allergens, air pollution and other environmental factors. Whether the maternal food intake during pregnancy is related to the development of allergies in the child is a subject that is widely discussed in the literature. Beckhaus et al. (709) made a meta-analysis on this subject. The authors investigated the publications on the effects of vitamins (A, B, C, D, E), zinc, magnesium, manganese and selenium administered during pregnancy as well as maternal dietary habits including fish, vegetables and fruits, meat, fatty acids, sugary drinks and Mediterranean diet, in order to answer whether those factors had any effect on the development of allergic diseases in the children of these mothers. They reported that vitamin D, vitamin E and zinc might have protective effects against childhood respiratory problems, however there was no evidence that they prevented development of asthma or other atopic disorders.

Some researchers claimed that eating fish during pregnancy or in the neonatal period could prevent allergic diseases in the children, based on the assumption that polyunsaturated fatty acids can prevent the development of allergic diseases due to their anti-inflammatory effects. In their meta-analysis, Zhang et al. (710) reported that a fish-rich diet during pregnancy did not prevent the development of childhood allergic disease in the baby. However, the authors stated that the maternal fish-rich diet in the neonatal period might decrease eczema and AR development in the child. However, there are no sufficient randomized controlled trials to make a definitive comment on this subject.

A number of studies investigated whether childhood vitamin D levels had any effect on AR development. Kim et al. (711) stated in their meta-analysis that vitamin D levels were lower in children with AR when compared to healthy children, however there was no evidence that vitamin D replacement could prevent development of allergic disorders. In contrast, Aryan et al. (27) reported that children with serum 25 (OH) D levels below 50 nmol / L were more likely to develop hypersensitization to aeroallergens compared to children with 25 (OH) D levels above 75 nmol / L, and this was gender-dependent. The authors claimed that vitamin D deficiency might be a risk factor for AR, particularly in boys. In conclusion, more detailed studies are required to define the relationship between vitamin D levels and AR development.

Epidemiological studies have revealed a relationship between childhood antibiotic use and asthma and other allergic disorders. On experimental animal models, the effects of pathological or commensal bacteria on the immune system were examined, and it was shown that exposure to antibiotics directed the immune response towards type 2 inflammation, by altering the intestinal flora. Ahmadizar et al. (712) included 34 articles and 340,428 patients in their meta-analysis, and demonstrated that children that received antibiotics up to 2 years of age had an increased risk of developing AR, eczema and food allergies. However, the authors could not find any relationship of antibiotic use with positive skin tests and allergen-specific IgE levels in the older ages.

### 8.1.1.2. Clinical course and diagnosis of allergic rhinitis in children

The clinical pictures of AR may be different in children and in adults. Classical symptoms, such as recurrent sneezing, nasal congestion and rhinorrhea, which are more pronounced in adults, may be subtler in children. Children with AR often seek medical attention with complaints of recurrent sore throat and upper respiratory tract infections. Frequent sniffing, nasal discharge, itching in the eyes, nose and palate, postnasal discharge, chronic cough, weakness, fatigue and decreased appetite are other frequent symptoms (38). Facial anomalies, dental anomalies and snoring are more pronounced in children with AR due to chronic mouth breathing, resulting from nasal congestion (383, 713).

AR has a number of comorbid conditions including asthma, allergic conjunctivitis, sinusitis, otitis media with effusion, frequent upper respiratory tract infections, dental disorders, obstructive sleep apnea, laryngitis and gastro-esophageal reflux.

The coexistence of AR and asthma is frequent. AR and asthma progress like two phenotypes of a common basic condition. The presence of AR should be considered as a risk factor for asthma. Approximately 50% of patients with AR have asthma. On the other hand, up to 85% of asthmatics have AR. Treating AR symptoms in patients with asthma is important for asthma control. Children with AR should definitely be evaluated in terms of asthma due to this close relationship between asthma and AR (417, 714).

Allergic conjunctivitis is characterized by itching, burning and watering in both eyes. Palpebral edema, chemosis and hyperemia are typical, and appear due to decreased venous return. Approximately 53% of the children with AR have concomitant conjunctivitis, and there is a correlation of the severity and duration of AR symptoms with the ocular symptoms in these patients (304). Since allergic conjunctivitis is often related to outdoor allergens, this rate increases up to 75% in studies conducted on patients with pollen hypersensitivity. The families usually ignore conjunctivitis symptoms. However, allergic conjunctivitis was indicated as the most frequent comorbidity of AR in a number of studies conducted on children (715, 716).

Nasal inflammation of AR may affect the ostiomeatal complex, and increase tendency for acute and chronic bacterial sinusitis. Positive skin tests have been shown in 54% of patients with chronic sinusitis symptoms. In another study, the frequency of AR diagnosed with skin tests was reported as 50-84% in patients who had sinus surgery. Although some authors find it reasonable that inflammation of AR triggers sinusitis, some authors do not agree. Taken together, epidemiological data show a relationship between sinusitis and allergy, but the role of allergy in sinusitis pathogenesis remains uncertain (717).

The symptoms of AR and sinusitis are quite similar in pediatric patients. The classical symptoms of bacterial sinusitis are mucopurulent discharge, nasal congestion and cough. No symptom alone is sufficient to make the distinction between AR and sinusitis in pediatric patients. However, mucopurulent discharge and cough are the most useful symptoms for diagnosing sinusitis in children with AR (718).

The relationship between otitis media with effusion (OME) and AR has been investigated in detail. It has been known that Eustachian tube dysfunction, inflammation and atopy play roles in OME etiology. Some studies indicated a higher OME prevalence in children with AR (92). It has been supposed that nasal mucosal inflammation of AR impairs Eustachian tube function, and causes OME.

AR has important consequences on children’s cognitive functions and quality of life. Chronic nasal congestion and associated sleep disorders lead to symptoms such as irritability, anxiety disorder, poor school performance and depression in children. Various studies suggested a relationship between attention deficit hyperactivity disorder (ADHD) and chronic rhinitis (719-721). In addition, the side effects of the medications used in AR treatment, conductive hearing loss and Eustachian dysfunction due to chronic nasal obstruction impair the school success and communication skills of these children (722, 723). Schans et al.(724) included 28 studies in their meta-analysis, and investigated the relationship between atopic diseases (asthma, eczema and AR) and ADHD. They found that there was a strong relationship between ADHD and asthma, eczema, and AR, and the atopic patients’ risk of developing ADHD in the older ages was 30-50% higher. Although the relationship between cognitive functions and atopy has been revealed by clinical research, the mechanism of this relationship is not yet clear. Different opinions have been proposed on the subject. It has been supposed that allergic sensitization and environmental stimulation might play a role in the development of ADHD. Another possible mechanism is the negative effects of inflammatory cytokines or allergic diseases on brain development. Some authors claimed that sleep disorders seen in allergic diseases might indirectly affect brain development in a negative way. On the other hand, Trikolaj et al. (725) claimed that ADHD developed independently of sleep disorders, and there was a relationship between increased IgE levels and poor cognitive functions. They suggested that cytokines responsible for allergic inflammation might cross the blood-brain barrier, and play a role in the maturation of cognitive functions. Melamed et al. (726) stated that the combination of methylphenidate and cetirizine was more effective in the treatment of ADHD in children with AR and ADHD. They also claimed that nerve growth factor might be regulating the relationship between the immune and the neurological systems. Cheng et al. (727) investigated the social and psychological effects of AR detected until the age of 7 in a cohort study including 5780 patients. They found that there was a relationship between AR and mood balance and self-discipline. In a meta-analysis, Miyazaki et al. (728) investigated the relationship between allergic diseases and ADHD, and they stated that children with ADHD were more likely to have AR, asthma, allergic conjunctivitis and atopic dermatitis compared to their peers, but such a relationship could not be established with food allergy. In conclusion, although there is a relationship between allergic diseases and ADHD, more detailed studies on pathogenesis are needed to fully explain the biological basis of this relation.

The diagnosis of AR is based on typical patient history, risk factors, classical symptoms and signs of the disease, and clinical testing of allergen-specific IgE. Although seasonal AR may often be diagnosed with the typical patient history, the history may not be sufficient alone in the diagnosis of perennial AR. The correlation between the responsible allergen and the symptoms may not be put forward in all patients, or children may not be able to express themselves adequately. Since the symptoms can be confused with recurrent upper respiratory tract infections in these children, patients should be evaluated for specific clinical signs of AR and comorbidities on physical examination. Mucosal edema, pale, bluish-nasal mucosa and clear rhinorrhea are seen on nasal examinations of allergic children, if they admit in the symptomatic period. A cobblestone appearance may be observed in the posterior pharyngeal wall and palate, which develops due to hyperplastic lymphoid tissue. In addition, some characteristic facial changes observed in pediatric patients with AR may help in the diagnosis. A transverse line may be seen in the supratip region of the nose, due to the fact that children repetitively rub their nose, which is called as “allergic salute”. The edema and color changes due to accumulation of hemosiderin (allergic shiners) as a result of venous ponding around the lower eyelid, and Dennie-Morgan lines developing due to the contraction of the Muller muscle are characteristic physical examination findings observed in children with AR. In addition, facial anomalies and dental malocclusion due to chronic mouth breathing can be frequently encountered in children with AR (716, 718).

Examination methods other than history, physical examination, skin tests and serum allergen-specific IgE are needed when the diagnosis is uncertain or when investigating the children for the comorbidities. Nasal mucociliary clearance and nitrous oxide measurement may be necessary in the differential diagnosis of primary ciliary dyskinesia. Nasal endoscopy and CT may be needed in case of nasal polyps, anatomical disorders and chronic sinusitis, and lateral radiographs can be used to diagnose adenoid hypertrophy (716).

### 8.1.1.3. Treatment of allergic rhinitis in children

AR treatment in children includes environmental control measures, pharmacotherapy and immunotherapy, as in adults. However, factors such as patient and family education, patient compliance, medication side effects, and family concerns about adverse effects should be considered in this group of patients (716, 718).

### 8.1.1.3.1. Environmental control and allergen avoidance

Avoiding allergens and symptomatic triggers is the first step in AR treatment. However, it is seldom possible to avoid allergens completely. Avoiding outdoor allergens is particularly difficult in children, and restriction of physical activities during the pollen season may lead to psychological problems. The studies investigated the effectiveness of avoiding pet and house dust mites failed to provide sufficient evidence on the effectiveness of avoiding these allergens. In their meta-analysis, Aroyeva et al. (729) concluded that the use of covers to protect from house dust mites as the primary treatment or a supplementary method did not contribute to protection from allergic disorders or prevented allergic symptoms. Nevertheless, many authors emphasized the importance of explaining environmental control measures to families, and avoiding nonspecific respiratory irritants such as cigarette smoke and strong perfumes (395, 397, 716).

### 8.1.1.3.2. Pharmacotherapy

Pharmacotherapeutic principles of AR are similar in adults and children. However, the physician should be careful regarding the side effects of the medications in children. In addition, it is wise to choose the medications administered once or twice a day in order to improve treatment compliance in school-age children.

Due to the limited benefit of environmental control measures, pharmacotherapeutics are usually necessary in children with moderate / severe or persistent AR. Treatment of AR with nasal corticosteroids, antihistamines or leukotriene modifiers is effective in reducing or controlling asthma symptoms (568).

### 8.1.1.3.2.1. Corticosteroids

Systemic corticosteroids should be rarely used in children with AR due to their side effects, and presence of safer and more effective alternatives. Nasal corticosteroids (NCS) are highly effective in pediatric AR (568). They provide significant relief in inflammatory symptoms such as rhinorrhea and nasal congestion. In addition, they improve asthma and bronchial hypersensitivity, ocular symptoms, sleep disorders and quality of life (730, 731). It has been reported that the effect of NCS begins within the first 12 hours after administration. However, it may take several weeks before their maximum effect can be seen (732).

The potential effects of these drugs on growth and development should be taken into account when using NCS in children. Many authors have claimed that systemic absorption is needed for any effect on the hypothalamic-pituitary-adrenal axis and growth, and NCS have limited or no influence on the hypothalamic-pituitary-adrenal axis at the recommended doses (733-735). The new generation of NCS (fluticasone propionate, fluticasone furoate, mometasone furoate and ciclesonide) have lower bioavailabilities and side effect risks compared to older NCS. However, caution should be exercised for the side effects in case of concomitant use of corticosteroids through different routes (such as nasal and inhaler corticosteroids).

In their meta-analysis, Mener et al. (446) analyzed the effects of NCS on growth and development in pediatric patients. The data of 755 patients from 8 papers were examined. All those studies evaluated growth rate by objective methods (kinemometer, stadiometer) in patients aged 3-12 years who were on fluticasone, mometasone, triamcinolone or budenoside treatment. The results indicated that NCS used in AR treatment might affect the growth rate negatively in the early period of use. However, the data were insufficient for the long term effects.

In conclusion, NCS should be administered to children in the smallest effective dose, the need for NCS should be re-evaluated periodically, and the children who use NCS for a long time should be followed-up for growth and development (446).

The irritative side effects of NCS such as crusting, dryness and epistaxis may be minimized with the correct use of the medication and education of the parents.

### 8.1.1.3.2.2. Oral and nasal antihistamines

Histamine is one of the most important mediators of allergic reactions. Therefore, antihistamines are the first choice options in AR treatment. Antihistamines provide improvement in early phase reaction symptoms such as nasal itching, sneezing, rhinorrhea. The effect on nasal congestion is limited. First generation antihistamines (ketotifen, chlorpheniramine, hydroxyzine, promethazine) may cross the blood brain barrier owing to their lipophilic structures; leading to sedation, attention deficit, and even seizures. First generation antihistamines should no longer be used in AR treatment in children due to the risk of sedation and their other negative effects (736, 737). Central nervous system side effects of new generation antihistamines are less because they do not cross the blood brain barrier. New generation antihistamines such as loratadine, desloratadine, cetirizine and fexofenadine should be preferred in the pediatric patients whose academic success and physical skills are restricted due to AR. Although new generation antihistamines have sedative effects in some children, this effect is very small compared to first generation antihistamines (30, 38, 737). Cetirizine, loratadine and levocetirizine may be used in children by 2 years of age. Although it was stated that desloratadine might be administered by 1 year of age, various authors have shown that it is safe for children after 6 months of age (738). Fexofenadine can be used after 6 years of age, and sedation has not been observed even with high doses. However, there are studies indicating that it is safe after 6 months of age (739).

In recent years, nasal antihistamines have been introduced. The nasal antihistamines in the market are azelastine and levocobastin. The efficacy and safety of azelastine has been demonstrated by multicenter studies. Grosman et al. (740) compared azelastine and placebo on 199 patients, and found that azelastine improved symptoms significantly compared to placebo. A number of authors compared the effectiveness of nasal and oral antihistamines. They found no difference between nasal and oral antihistamines for efficacy, however nasal antihistamines were found superior in terms of negative side effects (741). Rapid onset of action of nasal antihistamines is an important advantage. However, the difficulty of nasal administration and side effects such as bitter taste lead many families to prefer oral antihistamines in their children (716). It was shown that NCS are more effective in improving rhinitis symptoms (742). Recently, it was suggested that faster and effective symptomatic control could be achieved with combined azelastine and NCS nasal spray (584).

### 8.1.1.3.2.3. Leukotriene modifiers

Montelukast has been shown to be effective in pediatric patients with seasonal and persistent AR (743, 744). Neuropsychiatric adverse effects have been reported in some individuals, however studies have shown similar adverse effects with placebo (745, 746).

### 8.1.1.3.2.4. Nasal cromolyn

Cromolyn sodium is a well-tolerated topical mast cell stabilizer with a weak effectiveness on nasal symptoms. It needs to be administered 4-6 times a day to exert its effect. It is also recommended to be used prophylactically before exposure to the allergen. Its disadvantages are its limited effectiveness compared to other classes of medications, and difficulty of administration 4-6 times a day in children. However, since they do not have serious side effects, and they can be used safely in children (568).

### 8.1.1.3.2.5. Nasal anticholinergics

Ipratropium bromide nasal spray is effective on rhinorrhea, but it is not effective on other symptoms of rhinitis including itching, sneezing and nasal congestion. Its side effects due to systemic anticholinergic effect are very rare. Local side effects may be seen including dry nose, dry mouth, and headache. It can be used in children when rhinorrhea is the main complaint (622). Nasal pharmaceutical form of ipratropium bromide is not available in our country.

### 8.1.1.3.2.6. Decongestants

They reduce nasal edema by creating vasoconstriction and decreasing mucosal blood flow due to their sympathomimetic effects. Nasal decongestants are more effective than oral forms for relieving nasal congestion, and they have fewer systemic side effects. Vasoconstrictor decongestant sprays may be used for a short period to relieve severe nasal congestion in children, but long-term use may lead to rhinitis medicamentosa, characterized by rebound nasal congestion (716).

### 8.1.1.3.2.7. Saline irrigation

It has been shown that nasal saline irrigation may be used as an adjunctive therapy in AR, and helps controlling symptoms by removing nasal secretions, allergens, and irritants from the nose (747). It contributed to improvement of symptoms in AR, and reduced the need for antihistamines (748). Chen et al. (749) randomized 61 patients aged 2-15 years into nasal saline irrigation, nasal corticosteroid, and nasal saline irrigation + nasal corticosteroid groups, and reported that nasal corticosteroid was more effective than irrigation with saline in reducing AR symptoms, however nasal steroid + saline irrigation group needed a lower dose of nasal corticosteroid for an effective treatment.

Hermelingmeier et al. (750) performed a meta-analysis, and stated that nasal saline irrigation decreased the symptoms of AR by 27.66%, reduced medication use by 66%, improved mucociliary clearance and quality of life by 31.19% and 27.88%, respectively. Therefore, studies have shown that nasal saline irrigation may contribute to the treatment when used together with other medications, although it cannot provide adequate symptom control when used alone in the treatment of children with AR.

### 8.1.1.3.2.8. Anti-IgE

Omalizumab is a recombinant human monoclonal anti-IgE antibody that binds to circulating IgE to prevent them from binding to surface receptors of mast cells and basophils. Kamin et al. (751) showed that omalizumab increased the effectiveness and reduced side effects of immunotherapy in children with seasonal AR. In their meta-analysis, Tsabouri et al. (590) reported that omalizumab provided a significant improvement in the symptoms and quality of life of patients with moderate to severe AR, and reduced the need for medication in patients whose symptoms could not be controlled with pharmacotherapy. In addition, anti-IgE has been found effective in seasonal AR patients with simultaneous asthma who receive immunotherapy (594).

### 8.1.1.3.3. Immunotherapy

SIT is based on the principle of administering allergens that cause symptoms in increasing doses to reduce the hypersensitivity to the allergen. Immunotherapy is indicated in children with AR who cannot be adequately treated with appropriate medical treatment methods (38). It is considered as the only treatment method that can change the natural course of the disease, and provide long-term clinical improvement (718). Although some studies have shown that SIT may be applied in children under 5 years of age, it is recommended to be applied in children over 5 years of age due to difficulty of differential diagnosis in this age group, and higher risk of systemic adverse reactions in younger ages (752, 753). It is recommended to start immunotherapy early to obtain maximum benefit from SIT. Immunotherapy has been shown to reduce the development rate of asthma in patients with allergic rhinoconjunctivitis, when it is started early in life (300). However, it is not yet clear whether immunotherapy will prevent development of asthma or other allergic diseases in allergic children. In the meta-analysis conducted by Kristiansen et al. (754), it was stated that there was no clear evidence for prevention of a secondary allergic disease in the short or long term in patients treated with SIT. On the other hand, it has been reported that there is a short-term decrease in the risk of asthma development in patients with AR, but there is insufficient evidence for the long term effect.

SIT is traditionally administered by subcutaneous injections (SCIT). Different administration routes have been investigated due to difficulty of repetitive injections in children in children due to agitation as well as the risk of systemic allergic reactions. Allergens are administered into the sublingual tissues or the oral mucosa in SLIT, in the form of oral solutions or rapidly dissolving tablets.

Larenas-Linnemann et al. (755) reported in their meta-analysis that grass pollen SLIT was effective in children with seasonal AR over 5 years of age, however it could be administered to children over 4 years of age. They stated that grass and house dust mite SLIT could be effective in children with simultaneous asthma and AR, but SLIT should not be used as monotherapy in symptomatic asthmatics. The authors also stated that there was no sufficient evidence on the effectiveness of SLIT in mold allergy, the evidence for the effectiveness in milk and peanut allergy was limited, and the dose of SLIT should be increased slowly if SLIT was applied for these allergens. Feng et al. (756) performed a meta-analysis reviewing 26 articles and 2261 randomized patients, and reported that SLIT provided significant clinical improvement and decreased the need for pharmacotherapy.

Khinchi et el. (757) compared the effects of SLIT and SCIT in their double-blind and placebo-controlled trial. They showed that both treatment methods were effective compared to placebo. They reported that SCIT provided better treatment results compared to SLIT, however the difference was not statistically significant. When the two methods were compared in terms of side effects, no serious systemic reactions were observed in the SLIT group. The meta-analyzes and the studies comparing these two methods has shown that SLIT has a close efficacy with SCIT. Therefore, the decision for SLIT or SCIT should be made by considering the patient’s preference and accessibility of the extracts (758).

In conclusion, SLIT is a promising treatment method in the pediatric age group. However, randomized controlled clinical trials on larger series are needed due to the limited number of cases in the previous studies, and presence of data mostly on grass SLIT (759).

### 8.1.2. Treatment of allergic rhinitis in the elderly

**Method: **A total of 24 meta-analyzes were found in Pubmed database using the keywords ‘Allergic Rhinitis and aged and treatment’ and ‘Allergic Rhinitis and elderly and treatment’. Examination revealed that none of those were about the elderly population. A search with the keywords “Allergic rhinitis and geriatric and treatment” did not yield any meta-analyses either. Using the keywords ‘allergic rhinitis AND elder’, only 3 clinical studies were found between 2013 and 2018, however those were not performed on the elderly. A total of 15 studies were available between 2013 and 2018 with the keywords ‘(allergic rhinitis) AND geriatric’.

**Keywords:** Rhinitis, allergy, old age, geriatric, treatment.

We included the full text articles specifically investigating the treatment of AR in the elderly. Bousquet et al.(760) used a mobile phone application called as “allergy diary” developed for healthy and active aging, and planned to investigate the usability of technological communication tools in rhinitis and asthma in individuals over and under 65 years of age, and to obtain more detailed information from the patients with rhinitis and asthma. The authors have been investigating the elderly-adult population for the allergy symptoms, nasal and ocular morbidities, how these morbidities affect patients, how they apply treatment, the personal assessment of the benefits they receive from the treatment, the 1-year Visual Analogue Scale (VAS) score, and the follow-up of the workforce evaluations. The authors also aimed to evaluate the health systems and preventive medicine mechanisms in countries, and to make an effective use of sources through cost-benefit analysis. This study is still going on. The data obtained from this study will enable more accurate management of the elderly and others with AR. Similarly, Calderon el al. (761) have launched a study to identify the effects, reliability, cost-benefit analysis and comorbidities of allergen immunotherapy in the elderly for a healthy and active aging.

In parallel with medical and technological developments, the life expectancy and hence the elderly population is increasing. It has been supposed for a long time that skin prick test positivity is rare, there are changes in T/B lymphocyte ratios, decreased number of stem cells due to fatty degeneration in the bone marrow, lymph node regression, and decreased lymphocyte, macrophage and dentricitic cell functions in the elderly (762). It was thought that aforementioned factors decreased frequency of allergic disorders, or in other words, the allergies were diagnosed less in the elderly population due to those factors, and the allergic etiology was generally neglected in the elderly. However in recent years it has been suggested that the frequency of allergic disorders may increase in the elderly, due to multiple medication use, increased exposure to particulate matter as a result of environmental and air pollution (global warming, greenhouse gas effect), and use of additives in food production besides the effects of aging process and personal factors such as immune dysfunctions, inflammatory response (inflammaging) and degenerative changes in the body (763, 764). Recent studies suggested that there was no significant change in the Th2 pathway (765), the nasal mucosa cytology was not different (766) in the elderly when compared to the young people, and the local nasal allergen-specific IgE response was also present. The authors claimed that the diagnosis of the elderly patients was missed by 40% in the previous studies, and all of them were misdiagnosed in the previous examinations.

Contrary to the traditional belief that allergic diseases are seen less frequently in the elderly population, it has been reported in recent years that the prevalence of allergies in the elderly population is 5-10% (767), and the frequency of allergic sensitization accompanied by the clinical findings is up to 50% (768). Bozek et al. (769) performed a cross-sectional study on 2000 elderly individuals and showed that 13% had seasonal and 17% had perennial AR.

Different and more complex clinical pictures may be seen on evaluation of elderly patients with allergic complaints due to the fact that these patients are not a homogeneous group, there are changes in the physiology with aging, and accompanying comorbidities may be present (chronic diseases, multi-medication use, insufficient compensatory mechanisms, etc.). For example, rhinorrhea in an elderly patient may be due physiological alterations in the elderly (increased cholinergic activity, hormonal changes), old man’s drip as a result of decreased testosterone, mucosal atrophy, decreased mucociliary clearance, dehydration-mucous thickening, changes in external and internal nasal structures (columella retraction, etc.) (770, 771), as well as use of acetyl-cholinesterase inhibitors, aspirin or other non-steroidal anti-inflammatory agents (772), occupational and chemical agents, physical and emotional factors, or the viral infections (769). Considering rhinorrhea and nasal congestion as a result of AR without questioning the wide range of the aforementioned etiological factors, and prescribing medications may lead to other problems such as drug interactions and unnecessary use of medications, rather than solving the complaints of the patients.

In summary, diagnosis is the most important step of AR treatment in the elderly patients. However, due to the effects of the physiological changes mentioned above, some problems may be experienced in both in vitro and in vivo tests, and diagnosis can be difficult. For example, atrophy of the skin, altered vascular structure, sun damage, and cellular defects in allergenic responses may decrease test reliability, and prevent detection of atopy in skin prick tests (763, 764). Karabulut et al. (773) compared the skin prick test results of 32 patients over 65 years with 37 people between the ages of 40 and 45 years, and found positive prick tests in 50% and 75.7% in these groups, respectively, with a statistically significant difference in between. The authors suggested that skin tests should be performed in order to establish the correct diagnosis and to initiate the correct treatment, due to the difficulties and conflicts in the step of diagnosis. In addition to this proposition of the authors, it is important to note that the results of the skin prick test alone may not be sufficient to rule out the diagnosis of AR, and it may be necessary to measure allergen-specific IgE or examine possible local immunological responses for correct diagnosis or exclusion of AR. Besides higher frequencies of vasomotor rhinitis, atrophic rhinitis, and geriatric rhinitis, nonallergic rhinitis (NAR) as well as simultaneous NAR and AR should be taken into consideration in the elderly (774). The possibility of NAR coexisting with AR has been suggested by showing no change in the symptoms in long-term follow-up of patients who have been diagnosed with allergies despite decreased skin allergen positivity and IgE levels (770, 774). Considering this situation, Di Lorenzo et al. (269) tried to reveal the differences between two groups, to differentiate AR and NAR in presence of age-related changes in the diagnostic tests. The authors showed that NAR patients were older, had milder and less sneezing, nasal itching and conjunctivitis complaints, benefited less from antihistamines, had lower VAS scores, lower peak nasal inspiratory flows, and less nasal eosinophilia on nasal cytology. They stated that a more accurate diagnosis and treatment of AR could be made by using these criteria as supportive or exclusionary parameters in conjunction with the skin test results.

AR treatment in the elderly aims to reliably and effectively relieve the patient’s symptoms and improve quality of life with three main methods, including allergen avoidance, pharmacotherapy (corticosteroids, antihistamines, leukotriene antagonists, anti IgE, ipratopium bromide nasal spray) and immunotherapy. However, as mentioned above, the major problems in treatment of the elderly are the changes in drug metabolism, drug interactions, side effects of medications, comorbidities (liver, kidney failure, etc.), difficulties in drug selection and insufficient data on these issues.

### 8.1.2.1. Allergen avoidance

In terms of the elderly population, patients are more exposed to indoor allergens due to restriction in activities and more time spent at home. Therefore, frequent cleaning of the house, changing used items, keeping animals away from home, cleaning carpets regularly, ventilating home by taking pollen changes throughout the year and during the day into account have been suggested. The patients with pollen allergy should not go out between 11 AM and 15 PM, and use HEPA filters in the living spaces. In addition, nasal moisturizing and nasal irrigation are also recommended due to dehydration, decreased mucociliary clearance and decreased nasal blood flow with aging. However, the evidence is insufficient in terms of cost effectiveness and applicability of these measures (763).

### 8.1.2.2. Pharmacotherapy

### 8.1.2.2.1. Antihistamines

One of the first-line treatments for AR in the elderly population is antihistamines due to their good oral absorption, and reaching an effective plasma level in as short as three hours. However, it has been known that the first generation antihistamines can easily cross the blood brain barrier due to their lipophilic structures, and cause sedation, confusion, anxiety and impaired cognitive functions. It has been also known that they can cause peripheral vasodilation and hypotension, urinary retention and constipation due to non-selective H1 receptor blockage, and they often interact with other medications. Therefore, they should be used very carefully in the elderly population (776). It is recommended to use safer and more effective new generation antihistamines that make selective receptor blockage and do not cross blood brain barrier in the elderly (70). Desloratadine, levocetirizine, bilastine and ebastine have been reported to be safe in the elderly due to their good selective H1 receptor blockage, lack of anticholinergic and alpha adrenergic receptor antagonist activities, and their ability to inhibit proinflammatory cytokine release (772, 777). Another factor that will shape the drug preference for the elderly population is the need for regulation of drug doses in presence of kidney and liver diseases. Dose reduction is frequently required in the elderly population due to decreased renal excretion and changes in liver enzymes. Dose reduction is recommended for cetrizine, ebastin, levocetrizine and loratadine in patients with hepatic impairment or dysfunction, while dose reduction is recommended for cetrizine, ebastine, fexofenadine and levocetirizine in patients with renal impairment. Desloratadine does not need any dose regulation in the elderly who do not have any systemic disorders (778, 779). Jáuregui et al. (780) and Bousquet et al. (504) reported that a new molecule, bilastine, does not affect psychomotor performance or driving abilities, does not cause cardiovascular side effects, and does not require dose restriction in case of liver or renal failure in the elderly since it does not interact with cytochrome P450. It reduced symptoms, improved quality of life and did not cause any anticholinergic effect in healthy volunteers. It is suitable with European Academy of Allergy and Clinical Immunology (EAACI) / allergic rhinitis and its impact on asthma (ARIA) criteria.

The new generation antihistamines are safe except for terfenadine and astemizole in presence of cardiac diseases, which is another comorbidity frequently encountered in the elderly (781). However, caution should be exercised in terms of cardiac arrhythmias that may occur in patients using drugs such as ketoconazole, macrolides, quinolones and cimetidine, which inhibit liver microsomal enzymes.

Nasal azelastine may be used in seasonal AR treatment when oral medications cannot be used or response to treatment is inadequate since it has low risk of systemic side effects. Studies have shown that azelastine had equal efficacy to oral antihistamines such as ebastine, cetirizine, loratadine and terfenadine, it was easily tolerated in the elderly population, and effective on nasal congestion (530). In addition to its antihistamine effect, azelastine was shown to inhibit ICAM 1, it had anti-inflammatory effects by decreasing leukotriene synthesis and the reduction of inflammatory cytokines, and its combinations with corticosteroids was more effective (774, 782). However, adverse effects such as metallic taste, headache and burning sensation in the nose may cause patient incompliance (530).

In conclusion, oral or topical antihistamines can be used effectively and safely in the elderly, after evaluating liver and kidney functions, particularly in patients over 75 years of age. Dose adjustment may be necessary.

### 8.1.2.2.2. Corticosteroids

Corticosteroids are recommended for seasonal and perennial AR in elderly patients, due to their anti-inflammatory properties and beneficial effects on all symptoms of rhinitis including itching, rhinorrhea and congestion (772). Small number of studies reported that they were well tolerated, and had similar side effects (epistaxis, dry nose and burning sensation) in young people and the elderly (783). Most of these side effects can be prevented by giving patients detailed information about the use of nasal steroids, and prescribing new liquid-based and odorless formulas. The high rates of bleeding and dryness that have previously been frequently encountered in the elderly may be reduced by this way (784). Complications such as osteoporosis, diabetes, and cataracts are very rare, as the rate of absorption into systemic circulation is small due to the chemical properties of nasal steroids. Another question mark about nasal steroids is the fear of more disturbance in the nasal mucociliary clearance, which has already decreased with ageing. Studies have shown that the mucociliary clearance was not usually disturbed with use of nasal corticosteroids, and even improvement was reported with mometasone (784). Therefore, in the light of the aforementioned data, we may suggest use of the mometasone and ciclesonide in the elderly since they have the smallest systemic bioavailability (456, 764).

### 8.1.2.2.3. Decongestants

Decongestants are effective in relieving nasal congestion, but they do not have any effect on sneezing, rhinorrhea and itching symptoms. Oral decongestants may cause problems such as palpitations, insomnia, hypertension and urination problems in the elderly population, and they are not recommended in presence of coronary artery disease, diabetes, hypertension, narrow-angle glaucoma and obstructive urinary disorders (785).

The greatest risk of long-term use of nasal decongestant administration is the possibility of developing rebound vasodilation, nasal dryness, and rhinitis medicamentosa (784, 786). Therefore, decongestants are not recommended as the first choice agents, as monotherapy or for long-term treatment of allergic symptoms in the elderly (777).

### 8.1.2.2.4. Leukotriene receptor antagonists

Leukotrienes play a key role in allergic mechanisms, and their receptor-level antagonists are preferred in the treatment of allergic diseases since they reduce inflammation, congestion, sneezing and rhinorrhea, and improve quality of life. Montelukast used as a monotherapy was shown to be less effective than nasal fluticasone propionate, but had similar efficacy with loratadine. However, combination of leukotriene receptor antagonists with antihistamines and nasal steroids provided much more control in seasonal and perennial AR compared to monotherapy (787). Leukotriene receptor antagonists are also effective in the treatment of lower airway inflammation in asthma patients, therefore they are considered as advantageous treatment options for their synergistic effect in the elderly population. They are thought to be easily tolerated in elderly although data on long-term use is insufficient (783, 788). However, they should be used in the elderly population with caution due to the decrease in the clearance mechanisms in the elderly, and their potential to interact with drugs affecting the CYP3A4 and 2C9 enzyme systems (784).

### 8.1.2.2.5. Nasal cromolyn sodium

Cromolyn sodium provides mast cell stabilization as well as macrophage, eosinophil, monocyte and platelet inhibition, preventing release of inflammatory mediators and formation of both early and late phase allergic responses. However, it should be administered 4 times a day, and for about 3 weeks to show its beneficial effects. Therefore, it has no place in the treatment of acute attacks, however due to its safety and good tolerance, it is recommended for prevention of the symptoms in elderly patients who cannot tolerate antihistamines or other medications (598, 763).

### 8.1.2.2.6. Nasal anticholinergics

Although ipratropium nasal spray is used more frequently in nonallergic rhinitis, it can be considered as a treatment option in nasal discharge in the elderly population, refractory to other treatment options. It is not effective in other symptoms of rhinitis, and it has a good tolerance (789). It should be used with caution in patients with prostatic hypertrophy and glaucoma (784).

### 8.1.2.2.7. Anti IgE

Anti IgE treatment reduces inflammatory response and is used as a promising treatment option. However, there is insufficient data for its use in the elderly population.

### 8.1.2.3. Immunotherapy

It has been known that allergen specific immunotherapy (SIT) is the only treatment method that has the a longest-lasting effect in AR, and unlike other methods, may change the natural course of the disease. This treatment modality consists of administration of allergen in increasing doses until the maximal response is obtained, and maintaining this dose for 3-5 years. SIT was first administered through subcutaneous route (SCIT), then oral, sublingual (SLIT), nasal and bronchial routes have been used. SLIT has become popular and is frequently used in recent years due to ease of application and safety (758). SIT aims to reshape the immune response by decreasing the production of specific IgE by modulating immune response. Immune modulation includes release of different mediators (IFN-γ, IL-10, IL-12) by stimulation of T regulatory cells, and an increase in Th1 / Th2 ratio and allergen-specific IgG4, reduction of IL-4-5-13 (764).

However, SIT has been mostly neglected in the elderly population, and patients aged 65 years or older were not included in the study group even in the randomized trials. Different recommendations are available for the elderly population in terms of SIT. The basis of these recommendations is the belief that allergy is rarely seen in elderly, changes in immune functions, and accompanying comorbidities in these patients, and SIT is neglected in their treatment plan. Although the studies are insufficient, some authors claimed that SIT was effective in the elderly as much as in the young people.

In their review on management of allergic disorders in the elderly population, Ventura et al. (762) emphasized the importance of prevalence, diagnosis, concomitant comorbidities, and multiple medication use in the diagnosis and treatment of AR in the elderly, and stated that both SCIT and SLIT were effective for prevention of asthma progression, and tolerated well in patients older than 65 years of age.

Bozek et al. (790) performed a double-blind, randomized, placebo-controlled study to investigate the efficacy and safety of SLIT. They followed up 78 patients with grass pollen allergy for 3 years, and determined 64% decrease in the total nasal symptom scores of the patients while this rate was 7% in the placebo group. The authors reported that they did not encounter any systemic side effects.

Bozek et al. (791) used SCIT for 65-75-year- old patients with seasonal AR due to grass pollen allergy in their double-blind placebo-controlled study. They reported that the combined symptom and medication scores (41%), symptom scores (55%) and medication usage scores (64%) decreased significantly in the active treatment group when compared to placebo. Rhinoconjunctivitis Quality of Life Questionnaire (RQLQ) scores decreased significantly in the treatment group compared to placebo group. The authors stated that no systemic anaphylactic reactions developed in the treatment group during the study. Bozek et al. (791) also stated that the results of this study were in line with the results of the young age groups, and SCIT had similar immunomodulatory effects in the elderly and young people. In their study, they did not find a significant difference between the study and placebo groups for allergen-specific IgE and total IgE values, and suggested that these parameters were not suitable for evaluating efficacy of immunotherapy, and stated that IgE / IgG4 ratio could be more valuable in terms of laboratory efficacy of SCIT since this value was significantly different between treatment and placebo groups.

In another placebo-controlled double-blind randomized study, Bozek et al. (792) investigated the efficacy and safety of SCIT in the treatment of house dust mite allergy in elderly patients. They determined that there were significant improvements in the symptom and medication scores in the SCIT group compared to placebo group after 2 years of treatment. They showed a significant improvement in RQLQ scores compared to placebo group. First degree mild systemic anaphylaxis was observed in two patients in the SCIT group. The local reactions in the SCIT group included <5 cm erythema in 4%, and > 5 cm erythema in 1% of the patients. The allergen-specific IgE levels ​​decreased in the treatment group, while specific IgG4 values ​​increased significantly. With these results, the authors suggested that the immunomodulatory effect might be directed in elderly patients by using SCIT safely and effectively in treatment, and the pharmacotherapeutics may not be needed or their dose may be reduced.

Another point to be considered in the application of immunotherapy in the elderly population is the applications in presence of comorbid conditions. In the literature, a number of conditions that are frequent in the elderly population have been identified as clinical contraindications of immunotherapy. Regarding this issue, Pitsios et al. (793) made a new and comprehensive literature review on the use of immunotherapy in conditions that have been clinically regarded as contraindications, including asthma, autoimmune disorders, malignant diseases, cardiovascular disorders, chronic conditions (chronic infections, mental disorders, need for immunosuppressive therapy, incompliance with treatment), HIV positivity and use of beta blockers, angiotensin converting enzyme (ACE) inhibitors, and monoamine oxidase inhibitors. In that review, the authors reported that in asthmatic patients: SCIT (level of evidence: Ib) and SLIT (level of evidence: IV) administration were not expected to have a negative effect on the course of the disease, however application of SCIT in uncontrolled asthma patients might result in more frequent and more serious side effects (level of evidence: Ib), but SLIT applications did not cause more frequent and serious side effects (level of evidence: IV). They concluded that SCIT (level of evidence: Ib) and SLIT (level of evidence: IV) applications would have less efficacy in severe / uncontrolled asthma. Based on these results, they reported that immunotherapy application was definitely contraindicated in uncontrolled asthma, relatively contraindicated in controlled asthma, and there were no contraindications in well-controlled asthma (SCIT grade of recommendation: A, SLIT: grade of recommendation: D).

Although some case reports described a link between SCIT application and autoimmune disorders, the authors did not find any evidence for that (level of evidence: III). There was no evidence that immunotherapy administration caused more frequent and serious side effects in cases with autoimmune disorders (level of evidence: IV), and there was no evidence that immunotherapy would be less effective in individuals with autoimmune disorders (level of evidence: IV). In line with these results, it has been stated that immunotherapy is considered to be relatively contraindicated in the remission period, and definitely contraindicated in the active period of an autoimmune disease, and immunotherapy should be used carefully in presence of autoimmune disorders since the data are insufficient (grade of recommendation: D).

It has been stated that there is no information on the effect of immunotherapy on the course of the malignant diseases (NR), and that immunotherapy application is not expected to cause more frequent and more serious side effects (NR). It has been stated that immunotherapy is not expected to be less effective in presence of a malignant disease (NR). In line with these results, it has been reported that the application of immunotherapy in malignant diseases is considered as a definite contraindication (grade of recommendation: D).

It has been reported that use of beta blockers is a relative contraindication for immunotherapy since they prevent performing an effective treatment in case of anaphylaxis due to inhibition of the effect of epinephrine on beta receptors, and unmet alpha adrenergic effect (level of evidence: III). More serious side effects were observed in those using beta blockers, however an increase was not expected in the frequency of side effects (level of evidence: III). There is no evidence showing that immunotherapy is less effective when the patient is on beta blocker treatment (level of evidence: III). Therefore, the authors pointed out that administration of immunotherapy in individuals using beta blockers must be decided on a profit-loss account, and SIT was considered as relatively contraindicated in the ones that use beta blockers (grade of recommendation: C).

In case of use of ACE inhibitors, the authors stated that vasodilation caused due to their effects on the renin-angiotensin system might affect the response in anaphylaxis. Although it has been found that side effects may not occur more frequently, more serious side effects may occur in case of ACE inhibitor use (level of evidence: III). It was stated that there was no evidence for the effectiveness of immunotherapy (level of evidence: IV). In line with these results, the authors reported that there was no contraindication for immunotherapy in the ones that use ACE inhibitors (grade of recommendation: C).

It has been known that epinephrine, which is used in case of an emergency situation can cause serious hypertension and cardiac arrhythmias in patients using monoamine oxidase inhibitors. However, there is no evidence that immunotherapy causes more serious and more frequent side effects in the use of monoamine oxidase inhibitors (level of evidence: IV) or that immunotherapy is less effective (level of evidence: IV). Therefore, immunotherapy is not contraindicated in patients using monoamine oxidase inhibitors. However, caution should be exercised while using epinephrine in these patients (grade of recommendation: D).

There is no evidence that immunotherapy has a negative effect on the course of cardiovascular diseases (level of evidence: IV). There is no evidence that immunotherapy causes more frequent and more serious side effects, or immunotherapy is less effective (level of evidence: IV), however treatment of the side effects may be more difficult in these patients (level of evidence: IV). In the light of these results, the authors stated that the immunotherapy was relatively contraindicated in patients with cardiovascular disorders (grade of recommendation: D), and the decision for SIT should be based on obtaining expert opinion on cardiovascular disorder, careful evaluation of the disease and its treatment, anaphylaxis risk, and profit and loss assessment.

There is insufficient evidence regarding immunotherapy in HIV positive states; the present data are based on the patients without serious symptoms. Immunotherapy has no negative effect on the course of the disease (IV), more frequent and more serious side effects are not expected (level of evidence: IV), and it is not considered that immunotherapy will be less effective (IV), however these remarks cannot be excluded (level of evidence: IV). Therefore, immunotherapy application is accepted as a relative contraindication in HIV positive patients, and it has been stated that the decision should be individually based (grade of recommendation: D).

In general, immunotherapy has been reported to have a negative effect on the course of the disease in patients with immune deficiency or in need of an immunosuppressive therapy (NR), and theoretically it may increase the risk of more serious side effects (NR). In addition, immunotherapy is thought to be less effective in patients with adaptation problems and in patients with an impaired immune system (level of evidence: IV).

There are no reported contraindications for immunotherapy in hepatitis B-C positive conditions. Since nodule formation is observed in patients with sarcoidosis after SCIT application, SLIT application is considered as a good alternative. Primary immunodeficiency states are considered as contraindications.

In the light of these data, although the evidence level and the number of studies are insufficient, immunotherapy can be recommended as a useful, safe and successful method in the elderly population. However, additional care should be taken in terms of old age physiology, chronic diseases, multiple medication use and compliance of patients, and immunotherapy decision should be made on the individual basis (793).

In conclusion, in order to provide effective treatment for allergic conditions that are thought to be increasing in the elderly, first the diagnosis should be made and the comorbidities (vitamin deficiencies, genetic factors, concomitant diseases, multiple medication use, changes in drug bioavailability, hormone disorders, lifestyle, inflammaging and immune system changes) should be evaluated. Treatment methods applied in the elderly population, the success rates and reliability of these treatments are generally similar to the young population, but the most important point in planning treatment is the adaptation of patient-based treatment protocols and close follow-up of these patients.

### 8.1.3. Treatment of allergic rhinitis during pregnancy and lactation

**Method:** Pubmed and Google Scholar databases were searched with the keywords “Pregnancy, Allergic rhinitis, Rhinitis, Diagnosis, Treatment, Medications, Safety, Drug effects, Perinatal outcomes, Breastfeeding, Lactation, H1-antihistamines, Corticosteroids, Leukotriene receptor antagonists, Decongestants”, in order to find the relevant papers on “treatment of allergic rhinitis during pregnancy and lactation”. The papers published between 1970-2017 were included in this review.

**Keywords:** Pregnancy, allergic rhinitis, diagnosis, treatment, drug side effects, safety

### 8.1.3.1. Treatment of allergic rhinitis during pregnancy

Allergic diseases occur in approximately 20-40% of women of childbearing age, and 10-30% of pregnant women complain of AR and asthma (794). AR is usually present before pregnancy, however sometimes it may be evident for the first time during pregnancy (795). The allergic symptoms may be exacerbated, remain the same or decrease during pregnancy (796). Other possible causes of rhinitis during pregnancy are rhinitis medicamentosa, sinusitis and gestational rhinitis (797). Gestational rhinitis is defined as nasal congestion in the last six or more weeks of pregnancy in absence of any findings of respiratory tract infection or allergy (264). It is seen in 20-30% of pregnant women (605). Gestational rhinitis has been associated with hormonal changes, including placental growth hormone (798). It disappears completely within two weeks after delivery (264). Nasal saline irrigation is effective in reducing symptoms (799). Nasal decongestants, nasal steroids and nasal anticholinergics may be used (800).

The diagnosis of AR in pregnant women is made with a detailed medical history and symptom evaluation. If allergy testing has not been carried out in the past, in vitro tests such as allergen-specific IgE can be performed during pregnancy when necessary. Skin tests should not be performed during pregnancy, and postponed after delivery due to the risk of anaphylaxis (800).

Treating AR in pregnant women is important for the health of the mother and fetus. Otherwise, impairment of nutrition, sleep and emotional well-being of the pregnant woman may have negative effects on the fetus (801).

Balancing safety and efficacy of treatment is of paramount importance in the pregnant women (802). Almost all pharmaceuticals can cross the placenta. Malformation in the fetus is the most frightening situation, and this risk is the highest in the first trimester (803).

Prescribing drugs to pregnant women is difficult and troublesome for clinicians due to the lack of evidence-based information. FDA (Food and Drug Administration, USA) risk categories should be taken into consideration in prescription of medications during pregnancy (Table 8.1.3.1.1.) Category A and B medications are safe during pregnancy, however category D and X agents should be avoided. Unfortunately, none of the medications used in AR treatment meets pregnancy category A requirements, and many are in category B or C. (Table 8.1.3.1.2.) Therefore, the physician should make individual-based decisions, particularly when prescribing medications in category C. The patient should be informed about the negative outcomes of the disease process itself, if left without treatment, then should be informed about possible maternal and fetal side effects of the medication (795). The agents that have been considered safe during pregnancy should be preferred over new agents with unclear biological activity. The dose of the medication should initially be at the lower limit of the therapeutic range, and dose adjustment should be made as needed to optimize the outcome (804).

The general principles of AR treatment in pregnant women are not different from the treatment of non-pregnant women. Avoidance of allergens and irritants is the first step, before pharmacotherapy (797). Nasal saline irrigation has been shown to be beneficial and harmless (278). A stepwise pharmacological treatment should be planned if the symptoms cannot be controlled with these methods, and pharmacotherapy should be combined with non-pharmacological methods (805). Usually nasal corticosteroids and antihistamines are preferred in the pharmacological treatment of AR (806).

### 8.1.3.1.1. Nasal corticosteroids

Nasal corticosteroids (NCS) are the drugs of choice for AR treatment due to their good efficacy and pharmacokinetic properties (618). The only placebo-controlled randomized trial of NCS in pregnancy was conducted by Ellegard et al.(807), using fluticasone propionate in patients with gestational rhinitis. The results indicated that fluticasone propionate did not have any significant negative effect on maternal cortisol level, fetal growth or pregnancy outcome. In a case-control study conducted by Kallen et al. (808), it was found that the use of budesonide in pregnancy was not associated with cardiovascular defects in the fetus. In a recent prospective cohort study, Berard et al. (809) reported that there was no major congenital malformations with the use of triamcinolone, mometasone, fluticasone propionate / furoate, budesonide, or beclomethasone during pregnancy, including the first trimester.

NCS have been recommended as the first choice agents in AR treatment during pregnancy, especially after the first trimester (30, 795, 810). Since all NCS are similar in terms of efficiency and safety, continuing the preparation that adequately controlled the patient’s symptoms before pregnancy has been recommended (30). If NCS is to be administered for the first time during pregnancy, it has been recommended to choose budesonide, the only category B agent among NCS (810). Alhussien et al. (494) recommended the administration of fluticasone furoate, mometasone or budesonide in pregnancy owing to their low systemic bioavailabilities.

### 8.1.3.1.2. Systemic corticosteroids

Systemic corticosteroids are not usually necessary in AR treatment in pregnant women (618). The risk of cleft palate increases with systemic steroid use in the first trimester (811). In addition, systemic corticosteroids have been found to cause preeclampsia, preterm birth and low birth weight (812). On the other hand, it was reported in a recent review that there was a small increase in the risk of developing cleft lip after systemic corticosteroid use in the first trimester, however there was not sufficient evidence to support any relationship with preterm delivery, low birth weight, or preeclampsia (813). Use of systemic corticosteroids is recommended in the first trimester only if a severe disease responds only to systemic corticosteroids, and the risk of their use outweighs the possible fetal risks (814). Prednisolone or prednisone may be preferred since they can be oxidized by the placenta to their inactive forms (795).

### 8.1.3.1.3. Antihistamines

A number of studies have shown safety of both first and second generation antihistamines during pregnancy, including the first trimester (30). A recent meta-analysis showed that there was no relationship between the use of antihistamines in the first trimester of pregnancy and major malformations or other undesired pregnancy outcomes (spontaneous abortions, prematurity, stillbirth and low birth weight), and antihistamines could be used safely during pregnancy (815).

Most pregnant women with indication for antihistamines for AR are properly treated with second generation antihistamines since these agents cause sedation less, and they have less cholinergic side effects compared to first-generation agents (797). Among the second generation antihistamines, loratadine and cetirizine have been recommended based on their excellent safety data and the recommendations in the Guideline (794). Desloratadine is the main metabolite of loratadine; therefore, it may be assumed that it has a similar safety profile as loratadine (816). In addition, cetirizine can relieve nausea and vomiting during pregnancy (817).

### 8.1.3.1.4. Nasal antihistamines

Azelastine is not recommended during pregnancy as minor fetal side effects are observed in animals, and data on its safety are not available in humans (794).

### 8.1.3.1.5. Combination of nasal second generation antihistamine and corticosteroid

A new combination of fluticasone propionate and azelastine has been marketted. There are no studies in the literature regarding its use in pregnant women. It may be advisable to consider the measures applied for both components.

### 8.1.3.1.6. Nasal cromones

Due to its excellent safety profile during pregnancy, nasal cromolyn sodium may be considered as a first-line treatment in mild AR (806). It is the safest drug recommended in the first trimester of pregnancy (816, 818). However, it is not preferred much today since nasal corticosteroids have a similar safety profile. Nasal cromolyn may be a good alternative for patients who cannot use corticosteroids (795).

### 8.1.3.1.7. Decongestants

Higher risks for gastroschisis (819-822) (abdominal wall defect), small intestinal atresia (821, 823), endocardial cushion defect (824) and ear defects (825) have been reported with the use of oral decongestants in the first trimester of pregnancy (826). However, in the case-control study of Kallen et al.(826), no teratogenic effect was observed due to the use of oral decongestants during pregnancy. A recent case-control study by Yau et al. (827) supported previously reported endocardial cushion defect with phenylephrine, as well as ear defect and pyloric stenosis with phenylpropanolamine. The authors also observed increased risk of pyloric stenosis with the use of nasal decongestants in the first trimester, for the first time in the literature.

Since the safety data for systemic decongestants during pregnancy are insufficient, they are not recommended particularly in the first trimester (30). After the first trimester, they may be administered with caution (<3 days) (30, 794, 814). Topical decongestants can be used in conditions such as sleep disturbance due to nasal congestion, at the minimum dose and for a minimum time (preferably after the first trimester), which is sufficient to temporarily alleviate nasal congestion (806).

### 8.1.3.1.8. Nasal anticholinergics

Although side effects of topical ipratropium are rare, there are no studies in the literature regarding nasal use during pregnancy.

### 8.1.3.1.9. Leukotriene receptor antagonists

The available data for use of montelukast in pregnancy has mostly been obtained from studies on pregnant women with asthma (828-831). The use of montelukast for AR during pregnancy is not recommended as there are alternative treatments with equal or higher efficacy with more data on safety (814). Leukotriene receptor antagonist therapy should not be initiated during pregnancy. However, treatment may be continued if the patient has already been using them, and the benefit of treatment outweighs the risk of side effects (eg, severe asthma patients who benefit significantly from montelukast) (805).

### 8.1.3.1.10. Specific immunotherapy

The safety of allergen specific immunotherapy in pregnancy has been demonstrated by many authors. The first study on SCIT during pregnancy dates back to 1978. In this study, Metzger et al. (832) studied on pregnant women having immunotherapy, and the majority of patients started treatment before pregnancy. The authors did not find any difference between normal population and the pregnant women for the incidence of fetal and maternal complications. Shaikh et al. (833) reported that the incidence of complications in pregnant women treated with immunotherapy was not higher than the general population. This study did not only show that immunotherapy was safe during pregnancy, but it was also observed that there was less incidence of abortion, toxemia, and prematurity compared to the group of pregnant women who refused immunotherapy. In a prospective study, pregnant women who had SLIT were shown to have a smaller incidence of complications compared to pregnant women receiving pharmacotherapy and the normal population (834). In the light of these data, allergen immunotherapy in pregnancy is considered safe for the mother and fetus (814).

The maintenance therapy can be continued during pregnancy if the patient gets pregnant during immunotherapy, but the dose should not be increased (793). Termination of immunotherapy may be considered if pregnancy occurs during the dose-increasing phase, and the patient is receiving a dose that is unlikely to be therapeutic (629). Immunotherapy should not be initiated during pregnancy due to the risk of anaphylaxis (629, 293).

Some authors have suggested that immunotherapy can prevent allergic sensitization of the child as well as improving the allergic condition in pregnant women (835, 836), but more data is needed.

### 8.1.3.2. Treatment of allergic rhinitis during lactation

Almost all medications pass into breast milk with passive diffusion. The higher the plasma level, the greater the transition into breast milk. However, this amount is usually less than 2% of the dose taken by the mother. In addition, many agents cannot be absorbed from the gastrointestinal tracts of the infants, and clinically effective levels are rarely achieved (804, 837). It is considered that any medication that can be used in newborns can also be considered safe for lactating mothers (804).

Similar to pregnancy, it will be appropriate to use the lowest drug dose that is effective for controlling rhinitis symptoms, and for the shortest time during breastfeeding. Topical medications have the advantage of low systemic bioavailability, and are less likely to pass into the breast milk. The medication should be taken immediately after breastfeeding in order to decrease the dose reaching the baby whith breastmilk. In addition, it should be advised that the mother should be informed about the toxicity symptoms of the medication in the baby. For example, irritability can be seen in the baby with a decongestant taken from the breast milk (804).

Many drugs used in the treatment of AR [(montelukast (838), systemic corticosteroids (839), antihistamines (840)] have been reported to be safe during lactation, and unlikely to harm the baby. Hilbert et al. (841) reported that loratadine passed into the breast milk in very small amounts, and suggested that it could be preferred in lactating women. There are no specific data on the use of decongestants during lactation (804). It has been determined that pseudoephedrine decreases the amount of milk in lactating mothers (842).

In conclusion, although AR is not a life-threatening disease, it can have negative effects during pregnancy. AR treatment in pregnant women aims is to minimize the side effects in the mother and fetus while controlling the symptoms. Avoidance of allergens and non-pharmacological therapy should be the first option. If the disease cannot be adequately controlled with this approach, pharmacotherapy should be considered. Patients should be informed about the benefits and risks of pharmacotherapy.

NCS are recommended as the primary pharmacotherapeutics during pregnancy owing to their efficacy, little transition into maternal circulation, and no reported adverse effects. Cetirizine and loratadine have good safety and tolerability profiles in pregnancy. Oral decongestants should not be used as much as possible during pregnancy, particularly in the first trimester. Finally, pregnancy is not considered as a contraindication for the continuation of immunotherapy. However, immunotherapy should not be initiated during pregnancy.

Many drugs used in AR treatment may be used safely during lactation; however, it is recommended that the mother should observe the baby for drug toxicity.

**Keywords:** Allergic rhinitis, treatment, breastfeeding, drug safety.

### 8.1.4. Allergic rhinitis and its treatment in athletes

**Method:** The keywords “allergic rhinitis, sport, athlete” were used for searching Pubmed database, and 13 reviews were found between 2000 and 2015. Four of these articles were on AR and its treatment in athletes. There were no meta-analyses on this subject. A search with the aforementioned keywords yielded 22 clinical studies.

**Keywords:** Allergic rhinitis, sports, athlete.

### 8.1.4.1. Allergic rhinitis in athletes

The incidence of AR in professional athletes is higher than the general population. This rate is approximately 30-40%, and some authors have reported the prevalence as 60% (38, 843, 844). A study on professional athletes in Switzerland has shown that 16.8% of athletes have AR, and 59% of them need medical treatment during the pollen season (845). Katelaris et al. (844) studied on approximately 900 athletes performing on 34 different sports branches, 37% of the athletes met the AR diagnostic criteria, and 24% had seasonal allergic rhinoconjunctivitis.

The peak incidence of AR is between the ages of 6-25 years, and most of the professional athletes are in this age group (International Consensus Report On The Diagnosis and Management Of Rhinitis, 1994). This explains the high incidence of AR in athletes compared to the general population. Factors that increase the incidence of AR in athletes are the immunomodulatory role of physical activity on the immune system, great amount of allergens the athlete encounters during exercise, and the activities that athletes perform in different environmental conditions (834).

The athletes with AR may experience impaired sleep and quality of life, difficulty in concentration, and restriction in physical activities, and those affect the athletic performance negatively (847). A study on this subject showed deficiencies in the diagnosis and treatment of AR in Olympic athletes, and the negative effects of this on the athletic performance (847, 848). Athletes treated for AR have shown a marked improvement in their quality of life (849). Diagnosis and treatment of AR is of great importance in terms of athlete’s health, especially in the ones who have long-term, frequent and intensive training programs such as Olympic athletes, expected to be at the top of their physical performance.

Physical exercises have different modulating effects on the immune system. It has been suggested that increased physical activity may trigger AR and autoimmune diseases in healthy and young people (850). It is thought that light and moderate, short-term exercises have positive effects on immunity, however intense and heavy exercises cause a decrease in neutrophil function and NK cell count, inadequate IgA and IgM production of T and B lymphocytes, and an increase in proinflammatory cytokines, suppressing immunity (851). It has been supposed that intense and long-term exercise has suppressive effects on the immune system, causes the Th lymphocytes to shift towards the Th2 phenotype, and thus may increase the incidence of diseases such as AR in humans (850, 852-854).

Another factor that determines the effect of exercise on immunity is the kind of sports the athlete does. In a study, changes in the body after exercise were compared in running and cycling athletes, and muscle damage, pain, and systemic inflammation responses were found to be significantly higher in those running (855). Therefore, the response of the immune system to physical activity is thought to be related to the intensity, duration and type of the activity.

The glands in the nasal mucosa and the sinusoidal veins situated in the inferior turbinate play roles in production of nasal secretions, nasal resistance, as well as moistening, filtration and heating of the air entering the nose. These structures are innervated by the autonomous nervous system (856). Activation of the sympathetic system results in vasoconstriction in the venous sinuses. As a result, the turbinates get smaller, and the nasal resistance decreases. During dynamic exercises, nasal resistance decreases by approximately 50% due an increase in the sympathetic tone, while nasal breathing also increases (857). This is particularly important for athletes such as sprinters, who perform a short-term and explosive performance. Nasal congestion and related decrease in nasal breathing may directly affect athletic performance (858). Activation of the parasympathetic system causes vasodilation in the venous sinusoids, and an increase in the secretions of the submucosal glands leading to appearance of the symptoms of rhinitis including nasal congestion, itching and rhinorrhea.

The ventilation volume of the athletes may increase up to 200 liters per minute during the exercise. This may last for a short time in athletes needing speed and power, however lasts a longer in endurance athletes, such as long distance runners and swimmers (859). With the increase in ventilation volume, the amount of air and allergen that gets in touch with the nasal mucosa increases. Mouth breathing during exercise leads to contact of dry air with upper respiratory tract mucous membranes of the athlete, with high amount of allergens in unfiltered, poorly humidified air, increasing the susceptibility to AR. Although some athletes may experience reduction in symptoms of rhinitis during exercise, conditions such as exposure to outdoor and indoor allergens, inhalation or contact of irritant substances (ozone, sulfur, chlorine, etc.) often increase symptoms of rhinitis in athletes (860).

Since many sports are performed outdoors, athletes are exposed to high amounts of airway allergens during their activities. This is an important factor that may negatively affect the performance of athletes who have AR, and compete outdoors. The fact that Olympic sports are usually held during or immediately after the pollen season, increases the allergen exposure of athletes, and causes an increase in rhinitis symptoms of athletes with AR (848). It was shown that the amount of allergen in the air during Sydney Olympics was intense enough to cause symptoms in people with pollen hypersensitivity (847). This is why the aeroallergens have been monitored and air quality has been controlled recently in the cities where the Olympics are held in order to enable athletes to perform their sportive activities safely and healthfully (861).

The riskiest group of athletes in terms of AR are the ones engaged in outdoor sports, competing or training in cold and dry climatic conditions. Athletes who perform in the outdoor environment are exposed to more allergens than the indoor athletes. Long distance runners such as marathon runners, swimmers, ski and snowboard athletes, ice hockey players and deep diving athletes are the ones who show the symptoms of rhinitis most frequently. The characteristics and pathophysiological mechanisms of rhinitis seen in these athletes may vary (860). Allergic predisposition is higher in sportsmen making water sports when compared to the athletes who do not perform water sports. Less allergic predisposition was found in equestrian athletes compared to other athletes, and it was thought that this might be due to natural selection mechanisms (848).

The volume of ventilation per minute is higher in professional athletes as well as the amount of air passing through the nose. This causes a more intense exposure of the nasal mucosa to airborne allergens. This increases symptoms in athletes with AR. On the other hand, intense and long-term allergenic exposure may result in hypersensitivity to the allergen exposed. Dry and cold weather is an important factor causing rhinitis symptoms in individuals performing winter sports (skiing, snowboarding, ice hockey, etc.). Short-term contact of cold and dry air with the nasal mucosa causes an increase in both nasal congestion and the amount of nasal secretions, through neural reflex mechanisms, however prolonged contact may trigger epithelial damage and inflammatory reactions (862). Swimmers are the group of athletes that have rhinitis symptoms and allergic predisposition most frequently (848, 863). These athletes have been shown to have hypersensitivity to airway allergens on the water surface (848, 864). The frequency of rhinitis is significantly higher in athletes who perform sports in pools disinfected by chlorine gas or hypochlorite compared to the general population (865). It has been supposed that swimmers’ rhinitis is an irritation rhinitis that develops mostly due to the contact with chlorine. Examination of nasal mucosa samples of the swimmers with chlorine contact revealed rhinitis was accompanied by neutrophil infiltration. Prevention of contact with chlorine resulted in regression of rhinitis symptoms (866). The prevalence of AR in these athletes is also higher than the general population. Epithelial damage caused by prolonged contact with chlorine, and resulting inflammatory mediators may cause upper respiratory sensitivity in swimmers (867).

### 8.1.4.2. Treatment of allergic rhinitis in athletes

There are a number of medical treatment options in athletes.

### 8.1.4.2.1. Decongestants

Nasal decongestants act on alpha adrenergic receptors, reducing nasal resistance. Their fast onset of action cause rapid regression of the symptoms in the acute period, however they may cause rhinitis medicamentosa if used for more than 5 days (568). The use of oral pseudoephedrine, chlorpheniramine and phenylephrine is restricted or prohibited in professional athletes (847). The maximum urine concentration of pseudoephedrine should be 150 micrograms per milliliter in professional athletes (868).

### 8.1.4.2.2. Antihistamines

Antihistamines are effective on all symptoms of AR except for nasal congestion. They show their effects by blocking H1 receptors. First generation antihistamines are not preferred due to their side effects such as psychomotor impairment and sedation. On the other hand, new generation antihistamines (rupatadine, ebastine, azelastine, levocetirizine, desloratadine, etc.) can be used safely in symptomatic athletes at standard doses. Azelastine and levocabastine may be used nasally. Nasal preparations are easy to use. They do not cause sedation, and their effectiveness is similar with oral antihistamines. Due to their rapid onset of action, they are very effective in acute treatment (847).

### 8.1.4.2.3. Nasal anticholinergics

Nasal use of ipratropium bromide inhibits parasympathetic activity, reduces rhinorrhea, but has no effect on other symptoms of rhinitis. Therefore, it may be used to treat rhinorrhea in athletes performing winter sports (847).

### 8.1.4.2.4. Nasal cromones

Disodium cromoglycate and sodium nedocromil inhibit the release of leukotrienes from the mast cells. Both molecules are available for nasal and ocular use. They can be used safely in athletes, but they are less efficacious than antihistamines (568). They are used in prophylactic treatment of AR (38).

### 8.1.4.2.5. Nasal and systemic corticosteroids

Corticosteroids possess strong anti-inflammatory effects. The most effective drugs in the treatment of AR are nasal corticosteroids (545, 568, 869). A meta-analysis showed that topical corticosteroids were more effective than antihistamines in reducing AR symptoms (869). Although their effects start within a few hours, they show their maximum effect after two weeks of use. A study on professional athletes with AR reported that nasal steroids provided significant improvements in nasal complaints as well as quality of life and athletic performance (870).

### 8.1.4.2.6. Specific immunotherapy

Immunotherapy is the treatment option when there is no response to pharmacotherapy. In order to apply immunotherapy to patients with AR, it is necessary to demonstrate IgE-mediated allergy, a positive skin test, and unresponsiveness to other treatment options (30). Although small, the risk of anaphylaxis necessitates immunotherapy to be applied by experienced physicians where emergency medical intervention can be performed if necessary. Treatment should be started at least 3 months before the allergy season (871). Athletes should be warned not to exercise heavily after immunotherapy injections. Immunotherapy is a long-acting treatment modality. Therefore, it should be used in athletes if there is no response to other treatment modalities, it is impossible to avoid allergens, and exercise worsens the symptoms.

In conclusion, AR and its symptoms may result in sleep disorders, difficulty of motivation and poor physical performance in athletes. These may affect both the quality of life and success of the athletes negatively. Diagnosis and treatment of AR is of great importance for an athlete who is expected to do the best in training and competition. An effective treatment improves the athlete’s race performance; however, it is unrealistic to expect an untreated athlete to perform at his maximum. In the literature, there are insufficient data on the diagnosis and treatment of AR in professional athletes. This negatively affects both community and athlete health. It seems that some athletes refuse to receive treatment due to concern of doping, or that the treatment is given incompletely or incorrectly. This situation can only be prevented by informing the athletes, the clubs of the athletes and the sports federations by the health authorities about the diagnosis and treatment of AR. In this way, adversities such as refusing and abandoning treatment due to doping concerns may be prevented. This is an important step to be taken in terms of athlete’s health in our country.

### 8.1.5. Treatment of allergic rhinitis in patient with comorbid endocrine disorders

**Method: **The international literature was searched on Pubmed, Scopus, Google academic and Thomson Reuters databases with the keywords “Diabetes and allergic rhinitis, nasal steroid - diabetes, treatment of allergic rhinitis in diabetes, drugs used in the treatment of allergic rhinitis and diabetes, thyroid disorders and allergic rhinitis, Hashimoto-allergic rhinitis, Cushing’s syndrome- allergic rhinitis”. All international publications were included in this review between 2012 and 2018. An article in 1993 was included in the study as it was related to this subject. There were 21 articles published in the international literature between 2012 and 2018. After reviewing the abstracts of the articles, the articles thought to be not directly related to the topic were eliminated, 8 research articles and 2 meta-analyzes were evaluated. As a result, 2 meta-analysis and 8 research articles were included in this report.

Although there are no major treatment differences in AR treatment in presence of endocrine disorders, there are points to be considered.

AR treatment in patients with diabetes, thyroid gland disorders and Cushing syndrome, which are the most frequent endocrine disorders, has been discussed below.

### 8.1.5.1. Diabetes

Co-autoimmunity is more prominent in type 1 diabetes. There is an increase in Th1 / Th2 lymphocyte ratio in the favor of Th1. Th1 cells provide protection against intracellular bacteria, as well as protection against autoimmune diseases. Th2 cells are involved in allergen-specific sensitization in atopic patients, and work against extracellular bacteria (872).

In atopic diseases, Th1 / Th2 ratio is in favor of Th2. The studies conducted in patients with type 1 diabetes reported the rate of allergic diseases higher than the normal population. However, no significant correlation was found between total IgE levels and prevalence of autoimmune diseases (873).

Since AR and diabetes are frequent disorders in the community, their coexistence is also frequent. Avoiding allergens is the first step in AR treatment.

Studies with nasal steroids (NCS) have shown that these drugs are safe. They have no effects on serum glucose and hemoglobin A1c levels (874).

Depot steroids are among the drugs that should not be preferred due to the risk of negative effects on blood glucose levels. It has been investigated whether the depot corticosteroids used in AR caused diabetes or osteoporosis. Patients who received depot steroids 1-2 times a year were screened retrospectively, however no significant increase was reported in the risk of diabetes or osteoporosis in these patients (414).

Antihistamines, decongestants and anticholinergics may be used in presence of diabetes, but diabetic patients who already have dry mouth may experience an increase in this complaint (875).

Surgical interventions for AR should be done after blood glucose regulation in diabetics.

### 8.1.5.2. Thyroid disorders

The studies investigated allergic disorders in patients with autoimmune disorders of the thyroid gland, such as Graves’ disease and Hashimoto’s thyroiditis, found significantly higher prevalence for allergic conditions, and it was advocated that patients with autoimmune thyroid disorders should be under closer control in terms of allergic diseases (876).

It has been shown that Th1 cytokines play a role in Hashimoto thyroiditis while Th2 cells play role in Graves’ disease. Thyroid function tests were examined in patients with AR, and it was reported that the prevalence of Hashimoto thyroiditis was much higher than the normal population. This is supposed to be related to the impact of AR on lymphocyte ratios in these patients. Patients with AR should be followed up more closely for hypothyroidism (876).

A study on hyperthyroid patients showed that Graves’ attacks appear together with AR attacks and an increase of eosinophils in serum. AR treatment should be planned as soon as possible and the symptoms should be controlled even if the Graves’ disease is in remission (877).

It has been shown that AR in hypothyroid patients is more easily controlled with antiallergic therapy if administered with hormone replacement (878).

There is no difference in terms of pharmacotherapy of AR in patients with thyroid disorders.

**Keywords: **Allergic rhinitis, thyroid, Hashimoto thyroiditis, hypothyroidism, hyperthyroidism.

### 8.1.5.3. Cushing’s syndrome

Iatrogenic Cushing’s syndrome arises due to use of steroid hormones (879). The prevalence of AR may be expected to be lower in patients with Cushing’s syndrome, but there are no data on this subject. Depot steroid injections for AR in a patient with Cushing syndrome will increase the findings of this syndrome. In the past, cases of Cushing’s syndrome related to use of nasal corticosteroids were published (880).

In conclusion, the correct diagnosis of AR and the comorbidities (diabetes, thyroid disorders and other hormonal disorders) that may be present should be evaluated for an effective treatment of allergic conditions in patients with AR and simultaneous endocrine disorders. The comorbid disorders should be recognized, and the features of the concomitant diseases should be taken into account while planning the pharmacotherapy for AR.

### 8.1.6. Special occupations (heavy and dangerous jobs)

**Method: **A search in the Pubmed database with the keywords ‘Allergic Rhinitis, occupation, heavy work and treatment’ did not reveal any meta-analyses until 2015. Examination of national and international publications and theses did not reveal any publications either. There were no publications in the literature with the keywords ‘Allergic rhinitis and dangerous occupations, heavy work’. When the publications to date were examined, it was seen that there were clinical studies on ‘occupational allergic rhinitis’, however they did not specifically focus on heavy and dangerous jobs. Therefore, the relevant sections of the publications on “occupational rhinitis” were used when writing this chapter.

The prevalence of AR is 8-65% higher in the individuals who work in several regions and occupations (881-883). Occupational rhinitis is evident in 10-60% of healthcare professionals (884). The wide range in prevalence of AR in different occupations may be due to getting information through different methods (such as self-reported symptoms or the diagnosis of rhinitis by a doctor) (882).

Use of antihistamines may negatively affect people whose jobs need attention and high concentration, due to sedation side effects. The side effects of antihistamines do not just impair the occupations in need of driving, but also the jobs requiring writing or tracking. Non-sedating medications should be preferred in individuals whose jobs require attention and constant concentration (885, 886).

**Keywords:** Allergic rhinitis, dangerous occupations.

### 8.1.7. Treatment of allergic rhinitis in patients with other chronic conditions

There is not sufficient information on treatment of AR in presence of chronic diseases (glaucoma, hypertension, chronic kidney failure, chronic liver failure, etc.) in the ARIA guideline.

Medications used in the treatment of AR are nasal corticosteroids, nasal and oral antihistamines, leukotriene antagonists, anticholinergic agents, and nasal cromolyn (30, 246).

The main problem in presence of chronic disorders is drug interactions, especially in case of chronic liver and kidney failure (887).

Nasal corticosteroids are the most useful agents in AR treatment owing to their anti-inflammatory effects. There is no information about their effects on diabetes in long-term use. Mometasone and ciclesonide are the safest agents in presence of chronic organ failures since their bioavailability rates are small (888).

The use of oral corticosteroids is not recommended as this may aggravate chronic diseases such as diabetes and hypertension (889).

Antihistamines are the standard treatment for AR (890). Since the first generation antihistamines can cause confusion, sedation, arrhythmias, urinary disorders and hypotension, they are not recommended for treatment of AR anymore (891, 892).

Fexofenadine, cetirizine, loratadine, levocetirizine, desloratadine, bilastine and ebastine are the second generation antihistamines used frequently. Their drug interaction rates are small. However, they are not recommended in case of liver failure since most of them are metabolized by cytochrome P450 (893). Cetirizine, azelastine, ebastine and desloratadine should be used with caution in renal failure (894, 895).

Nasal and oral decongestants relieve nasal congestion, and they are frequently used in AR. These agents may aggravate hypertension and glaucoma, therefore should not be used in these circumstances (890).

Antileukotrienes are effective in all nasal symptoms, and are well tolerated (772).

**Keywords: **Allergic rhinitis, chronic disease.

### 8.2. Surgery in allergic rhinitis

**Method:** The keywords “allergic rhinitis, surgery, turbinate” were used to search Pubmed database, and 1 systematic review, 1 meta-analysis and 15 review articles were found between 2000 and 2015. It was seen that 5 of these articles were about inferior turbinate surgery in patients with AR. Apart from these studies, 27 clinical studies were found. Ten of them were on turbinate surgery in patients with AR.

Pubmed database was also searched with the keywords “allergic rhinitis, surgery, septum” in the same time period. There were no meta-analyses, while 10 review articles were found. It was observed that 3 of these articles were on nasal septum surgery in patients with AR. Apart from these studies, 7 clinical studies were found, only one of these studies was on nasal septum surgery in patients with AR.

### 8.2.1. Inferior turbinate surgery and septoplasty in patients with allergic rhinitis

Nasal congestion is the most common reason for admittance of AR patients to otorhinolaryngologists (896). The main cause of nasal obstruction is inferior turbinate hypertrophy in these patients. The inferior turbinate is a dynamic structure that has vascular and neural structures designed to respond reactively to various stimuli, including allergens, irritants and changing environmental conditions (897). The nasal septum is a stable structure and has no potential for generating a reactive response.

Primary treatment of AR is allergen avoidance and pharmacotherapy. Immunotherapy may be applied to patients who do not benefit from pharmacotherapy. Surgical treatment comes to the fore when no response is obtained to any of these treatment modalities, or in cases where administration of medications is contraindicated (898-901). If surgical treatment is planned in the treatment of a disease accompanied by inflammation such as AR, surgery should be directed primarily to the inferior turbinates. However, septoplasty should also be performed if turbinate hypertrophy is accompanied by nasal septum deviation (902).

Inferior turbinates are bony structures covered by nasal mucosa. They play role in adjustment of the temperature of the breathing air, mucociliary transport and regulation of nasal resistance. The first contact with the allergen occurs at the anterior mucosa of the inferior turbinate. Under this mucosa are the mucous glands, goblet cells, nerve fibers and vascular network (897). Patients with AR develop hypertrophy in the glandular structures situated in the submucosa of the turbinates, and congestion in the cavernous veins. This is the main cause of rhinorrhea and nasal congestion in patients with AR. The main aim of turbinate surgery in AR is to decrease the volume of the turbinate and hence nasal resistance, and to relieve nasal obstruction. Turbinate surgery may be directed to the hypertrophic mucosa, turbinate bone, or both (903). Partial or total turbinectomy, turbinate lateralization, electrocauterization, cryosurgery, submucosal resection, microdebrider turbinoplasty, laser vaporization, radiofrequency or coblator ablation are the most frequently employed surgical techniques (896, 903, 904). Apart from these, some agents may be injected into the turbinates. Steroids or sclerosing agents may be injected into the turbinates. Sodium marrhuate 5% was used as sclerosing substance in the past, but this method is not used today. The effect of intramucosal corticosteroid injections lasts 6 weeks. Although rare, it has complications such as retinal artery vasospasm and embolism that result in blindness (905).

Today, the functions of the turbinates are more clearly understood. Therefore, radical turbinate resections have been abandoned due to complications such as atrophic rhinitis or empty nose syndrome (906).

Turbinate lateralization was first described by Killian as an alternative to radical turbinate resection (907). With this technique, it is very unlikely to damage the turbinate mucosa or the nasolacrimal system. Turbinate lateralization is widely used owing to its simplicity, and low risks of bleeding and synechiae formation. Although nasal passage widens in the early period, there is a risk of reappearance of symptoms due to medialization of the turbinate over time (907, 908).

Electrocauterization technique involves high-energy coagulation of the medial side of the inferior turbinate. Electrocauterization is performed from several points, starting from the posterior end of the turbinate. A nasal pack should be placed between the turbinate and septum at the end of the procedure to prevent formation of synechiae. The beneficial effect of this application on nasal congestion is short-lived, and it has complications such as synechiae, crusting and thermal injury (907). In submucosal electrocoagulation, needle cauterization of the submucosa is performed. This application has fewer complications compared to classical electrocoagulation due to the preservation of the medial mucosa of the turbinate, however care should be taken not to coagulate the turbinate bone.

A cryoablation probe working with nitric oxide is placed over the medial surface of the inferior turbinate in cryosurgery. The effect of cold creates necrosis in the goblet cells located in the submucosa. Its effect is short-lived, and complications such as synechiae, crusting and bleeding may be seen (907).

Submucosal resection has been developed in order to protect mucosa covering the turbinate as well as its physiological functions while shrinking the turbinate mass. It may be preferred particularly in patients with a hypertrophic turbinate bone (907). In the classical technique, the inferior turbinate bone is dissected from the surrounding mucosa, and removed. Protection of the medial turbinate mucosa protects the functional mucociliary transport. Protection of the medial mucosa is important in terms of preventing complications such as synechiae, crusting and bleeding, however there is a risk for osteitis in cases where the turbinate bone is exposed due to mucosal loss (909). There are various modifications of submucosal resection technique. In these techniques, the submucosal cavernous system is excised with forceps or a microdebrider. It has been shown that the autonomic and sensory nerve fibers located in the turbinate are damaged with submucosal resection, and allergic symptoms decrease in patients with AR (910). In microdebrider turbinoplasty technique, submucosal tunnels are created, and the cavernous system is excised with the microdebrider. The turbinate bone is not excised. In a study involving 160 patients with AR, it was shown that microdebrider turbinoplasty combined with inferior turbinate lateralization had similar effects with submucosal resection in terms of decreasing allergic symptoms (911). Microdebrider turbinoplasty was found superior to submucosal resection in terms of blood loss and duration of surgery (912).

Laser vaporization triggers submucosal fibrosis, reducing the turbinate volume and mucosal surface area. The advantages of this technique are minor bleeding and postoperative pain, and fast recovery. However, its effect is short-lived (907). There are rare complications such as synechiae formation, crusting and bony exposure. Various laser types such as carbon dioxide, diode, Nd:YAG, KTP, argon and Ho: YAG have been used in the treatment of turbinate hypertrophy. The tissue penetration depths of these lasers differ. The carbon dioxide laser has been shown to cause a marked decrease in the number of submucosal seromucous glands, and is highly effective on rhinorrhea (913). Diode laser was used for inferior turbinate hypertrophy in 40 patients with seasonal or perennial AR, and significant improvements were observed in obstruction, rhinorrhea and itching in both groups. The patients with seasonal rhinitis improved more (912). The easy use of diode lasers in the office has made this method popular (914).

The radiofrequency ablation technique creates coagulation necrosis in the tissues in the early period as a result of the increased temperature caused by the transfer of low-frequency energy to the submucosal region. During this process, 350 joules of energy is transferred into the tissues from several points. The tissue temperature rises to about 75 °C. Afterwards, the turbinate shrinks due to contraction and fibrosis (909). This procedure can be performed with local anesthesia. Patients may experience some pain during the procedure, but it is often tolerable. The long-term effects of radiofrequency were investigated in a study involving 101 patients with AR. The authors reported the response rates as 77.3% and 60.5% at the postoperative 6th month and 5^th^ year, respectively. The AR symptoms (nasal obstruction, discharge, itching, sneezing, and ocular tearing) improved significantly. The benefit of treatment on eye symptoms suggested that radiofrequency might have suppressed local immune response or naso-ocular reflex (915). A study was conducted on 45 patients with inferior turbinate hypertrophy, unresponsive to medical treatment that was administered for at least 3 months. A single session radiofrequency ablation was applied to the inferior turbinates of the patients from 3 points, and significant improvement was obtained in nasal obstruction in 2nd and 6th months after the procedure (916).

Coblator ablation creates molecular ionization in the tissues at lower temperatures compared to the radiofrequency technique. In this way, thermal damage to the surrounding tissues and resulting pain are minimized (917). There was a significant increase in the nasal volumes of the children with AR after coblator ablation of the turbinates. These patients showed a marked improvement in AR symptoms for 6 months (917).

Approximately 80% of the people have various degrees of nasal septum deviation. Septum surgery has a high success rate when performed with a correct indication (918). However, septoplasty should not be considered as the primary treatment option in patients with AR (919). Due to the low success rate of septoplasty in patients with AR in the past, it was suggested that patients should be carefully evaluated before planning surgery, and septoplasty should not be performed in absence of a definite indication (920). The obstruction recovery scores of patients who had AR and underwent septoplasty were found to be lower than those who did not have AR and had septoplasty (919, 921). However, the authors of these studies did not mention whether they performed any intervention on the inferior turbinates, or there was inferior turbinate hypertrophy.

Interruption of integrity of the nasal septal mucosa in patients with AR may be a risk factor for the development of septal perforation after septoplasty (294). In addition, risk of septum perforation due to chronic nasal corticosteroid use is higher in these patients (922). In a study on this subject, the patients with and without AR were compared in terms of occurrence of septal perforation after septoplasty, and no significant difference was found between two groups (923).

Today, septoplasty is performed in the presence of an apparent anatomical nasal septal deformity causing obstruction in AR patients. In this way, both obstruction is relieved, and performance of any additional procedures (turbinate surgery, FESS) is facilitated. The improvement in obstruction symptom was significantly higher in patients with AR who underwent septoplasty and turbinate surgery compared to the group that had turbinate surgery alone (902).

In conclusion, inferior turbinate hypertrophy is quite frequent in patients with AR. The nasal airway may be enlarged with inferior turbinate reduction in patients with inadequate response to pharmacotherapy, or incompliant to treatment. Today, conservative methods are used to protect the inferior turbinate function instead of radical procedures. On the other hand, in case of nasal septal deviation in an AR patient, septoplasty is the appropriate treatment in terms of widening nasal airway. Septoplasty is also useful to increase the effectiveness of nasal topical agents. Septoplasty should be planned at the time when the patient has the least allergic symptoms, and is under medical treatment, if necessary. However, it should be kept in mind that patients with AR may benefit less from surgical procedures directed to nasal septum and inferior turbinate when compared to the patients without AR.

**Keywords:** Allergic rhinitis, treatment, surgery, turbinate, septum.

### 8.2.2. Vidian neurectomy in allergic rhinitis

**Method:** In the Pubmed database, 1 meta-analysis was found in the search conducted by the keywords ‘Allergic rhinitis, vidian neurectomy’ until 2015. When national and international publications and theses were analyzed, 1 publication was found related to vidian neurectomy in allergic rhinitis. The analysis of the publications up to present revealed 47 publications. The section was written by making use of the publications reached by using keywords “vidian neurectomy in allergic rhinitis” and “vidian neurectomy” alone.

Vidian neurectomy is not the first choice treatment of AR. Conservative treatment options (avoiding allergens, pharmacotherapy, etc.) have priority. If all conservative treatments fail, a vidian neurectomy may be performed (924).

After the description of Golging-Wood (925) in 1960, vidian neurectomy has been performed with various methods. Vidian neurectomy is effective for improving symptoms of AR and vasomotor rhinitis (926). With the introduction of endoscopy in paranasal sinus surgery in the 1980s, Kamel and Zaher (927) first benefited from this method for intervention to the vidian nerve.

The use of transnasal endoscopy in vidian neurectomy by El Shazly (928), El-Guindy (929), and Robinson – Wormald (930, 931) has started a new era.

The vidian nerve carries parasympathetic fibers from the facial nerve to the sphenopalatine ganglion. The parasympathetic fibers synapse and divide into three branches in the sphenopalatine ganglion. These nerves innervate the lacrimal gland, palate and nasal mucosa. Computerized tomography is the best imaging modality for evaluating the vidian canal. Lee et al. (931) classified the vidian canal into three types based on CT findings: the vidian canal is completely within the sphenoid sinus (type 1); vidian canal is on the sphenoid sinus floor or partially protruding into the sphenoid sinus (type 2); and the vidian canal completely embedded in the sphenoid body (type 3).

Although different vidian neurectomy techniques have been described until today, vidian neurectomy is performed through the intrasphenoidal approach if CT shows intrasphenoidal protrusion of the vidian canal, and transsphenoidal approach is used if the vidian canal is buried in the sphenoid body (931). Liu et al. (932) described a similar technique in 2010.

Robinson and Wormald (930) showed improvement of nasal congestion and rhinorrhea after vidian neurectomy, however there was no significant benefit for sneezing or postnasal dripping (928). Jang et al. (933) reported similar results.

Lee et al. (931) published the largest retrospective series on 178 patients. Over a mean follow-up period of 1.5 years, more than 90% of patients reported that they were satisfied with the surgical intervention. The incidence of postoperative dry eye was reported as 23% in this series.

Dry eye is the most common problem, and may be seen in 12-30% of the patients. Dry eye occurs due to the loss of postganglionic secrotomotor fibers innervating the lacrimal gland. The risk of a serious complication such as vision loss may be minimized with a good preoperative imaging, assessment, and employment of the endoscopic approach. Temporary cheek and tooth numbness (due to maxillary nerve damage), as well as nasal crusting and dryness are also frequent complications after surgery. Another potential complication of vidian neurectomy is sphenopalatine artery bleeding. Bleeding may be controlled with cauterization, and other complications may be prevented (931).

**Keywords:** Allergy, vidian neurectomy.

### 8.2. Other treatment methods in allergic rhinitis

### 8.2.1. Acupuncture

**Method: **International literature was searched with the keywords “allergic rhinitis, acupuncture” in Pubmed, Scopus, Google academic and Thomson Reuters databases. Until 2015, three meta-analyzes were identified and used in this chapter. Between 1961 and 2018, 182 international publications were analyzed. There were 127 articles published in the international literature between 2008 and 2018. After reviewing the abstracts of the articles and eliminating the ones that were not related to “acupuncture in allergic rhinitis”, 23 research articles and three meta-analyzes were taken into consideration, and at the end, three meta-analyzes and 23 international publications were included in this review. Ulakbim and Google academic databases were searched without any date restriction with the keywords “allerjik rinitte akupunktur” for national publications, however no clinical research papers were identified.

### 8.2.1.1. The basics of acupuncture

Acupuncture is based on the relationships among 14 different energy channels passing under the skin, and the resistance points related to organs on these channels. Human being is the part of the energy in the nature, and directly affected by climatic or other energies such as cold, hot, humid, dry and so on. Acupuncture method assumes that there is an energy network that envelops the entire surface of the body. This network is resembled to life energy. There are control points that reduce, increase, deflect or direct this energy, and even direct it towards a certain point. This is referred to as “motion wakeup” or “reflex wakeup” in medicine. In this method, a needle pricked in a certain point of body is expected to create a reaction in some other part of the body. In this way, disease prevention or control is expected.

Acupuncture has a polygenetic origin, and has been known as a method of therapy for centuries. It has been traditionally practiced in East Asian countries. It has found wide application areas in Europe in the recent years. Today, acupuncture is one of the most important parts of modern complementary medicine. It is performed in similar ways in adults and children.

### 8.2.1.2. Mechanism of action of acupuncture

The majority of acupuncture points (up to 80%) represent anatomical holes in the superficial body fascia, where blood vessels and nerve bundles pass into the skin from loose subcutaneous connective tissue. These points are rich in receptors, and it has been shown that most of them (up to 71%) represent myofascial trigger points. These points located on the skin surface have 10 to 100 times less skin resistance, and a higher electrical capacity (934). Acupuncture has clinically been proven to be effective, and is used particularly for treatment of pain and musculoskeletal disorders (935-937). However, this method may also be effective on chronic or acute phases of other conditions. Its effect on the immune system has been shown in several papers (938). Acupuncture has been shown to modulate the activity of natural killer cells, lymphocyte proliferation, chemotaxis and phagocytosis (939-941). In addition, reduction of eosinophils in blood and nasal secretion has been observed (942).

### 8.2.1.3. The effect of acupuncture on pathogenesis of allergy

Acupuncture probably affects the cytokine profile. Its modulating effect on the cytokine profile was reported in several studies on bronchial asthma (943-945) and AR (946, 947), in both humans and animal models, and improvement of symptoms have been reported. It is worth noting that the production of all cytokines has not been affected by acupuncture.

IL-10, IL-2 and IFN-γ are particularly influenced by acupuncture (943-946). Beyond cytokine modulation, some researchers have reported decreased IgE levels in blood (948, 949). Changes in cytokine production was accompanied by reduction of symptoms in those studies despite lack of evidence on a direct relationship between cytokine alterations and reduction of the symptoms.

### 8.2.1.4. Acupuncture research on allergic rhinitis

The effects of acupuncture on allergic symptoms and quality of life have been studied. Acupuncture was reported to reduce nasal and conjunctival signs and symptoms, and improved quality of life (946, 950-952). It seems that not only the classical sinonasal and ocular symptoms, but also pruritus due to atopic dermatitis were improved (953).

Lee et al. (954) selected only 7 studies out of 115 randomized clinical trials for their meta-analysis. Evidence was diverse for the effectiveness of acupuncture in symptomatic treatment and in the prevention of AR. Specific effects of acupuncture could not be demonstrated in seasonal AR. There was clear evidence for the effectiveness of acupuncture in perennial AR.

A second meta-analysis by Roberts included only seven studies that met his quality criteria (955). The results of this analysis did not show any evidence on the effect of acupuncture in the treatment of allergies.

Two multicenter, randomized controlled trials have been launched recently in order to bridge this gap. In the study carried out by ACUSAR (acupuncture in seasonal AR), a multicenter study on acupuncture was performed on 422 patients with seasonal AR in Germany. In this study, acupuncture was compared with classical antihistamine treatment and “fake” acupuncture, for reducing symptoms and improving quality of life. The results revealed a statistically significant improvement in quality of life in the “real” acupuncture patients (956). A second study with a similar design has currently being conducted on 238 patients in Korea and China, and the effects of acupuncture on perennial AR is being investigated (957). Significant improvements were observed in rhinitis symptoms and quality of life (958).

### 8.2.1.5. Conclusions

Integration of the principles of acupuncture into modern European medical knowledge may only be done to a small degree. Many of the proven therapeutic effects of acupuncture are controversial for modern science, and further research is needed. The effectiveness of acupuncture in AR and other allergic conditions such as asthma and allergic eczema depends on its effect on Th1 / Th2 cells, cytokine profile regulation, and particularly the expression of IL-10, IL-2 and IFN-γ. However, further studies are needed to confirm this hypothesis. The effects of acupuncture have been shown in a number of clinical trials.

Multi-center, controlled studies are currently on the way to reveal the complementary role of acupuncture in AR treatment.

Acupuncture has an effect comparable to pharmacotherapy in moderate to severe AR, and it is a safe method without serious side effects.

**Acupuncture: **Clinicians may recommend acupuncture, or patients with AR who are interested in non-pharmacological treatment can contact a clinician who can offer acupuncture treatment. Option based on randomized controlled trials with limitations, observational studies with consistent effects, and benefit over harm.

### Action Statement Profile (959).

• Quality improvement opportunity: Increased awareness of acupuncture as a treatment option for allergic rhinitis

• Aggregate evidence quality: Grade B, based on randomized controlled trials with limitations, observational studies with consistent effects

• Level of confidence in evidence: Low; the randomized trials did not show comparison to traditional medical therapy for allergic rhinitis and had methodological flaws

• Benefits: Effective alternative to medical therapies, reduction of symptoms, may more closely align with patient values, improved quality of life, avoidance of medication use and potential side effects

• Risks, harms, costs: Logistics of multiple treatments, need for multiple needle sticks, cost of treatment, rare infections

• Benefit-harm assessment: Equilibrium of benefit and harm

• Value judgments: Panel members varied in their preconceived bias for or against acupuncture

• Intentional vagueness: None

• Role of patient preferences: Limited—potential for shared decision making

• Exclusions: None

• Policy level: Option

• Differences of opinions: None

**Keywords:** Allergic rhinitis, acupuncture.

### 8.3.2. Probiotic treatment in allergic rhinitis

**Method: **Pubmed database was searched with the keywords, and three meta-analyzes were found before 2015. There were four meta-analyzes, 24 reviews and 14 clinical studies between 2013 and 2018. In the search done without any date limitation, a total of 157 publications were found on allergic rhinitis and probiotics in the literature.

**Keywords:** Allergic rhinitis, Probiotics

Probiotics have been described as living microorganisms, and they provide health benefits to the person when ingested in proper quantities. Probiotics are naturally found in foods such as yogurt, kefir, pickles, vinegar and dark chocolate, and recent research has shown their beneficial effects in prevention and treatment of infections and inflammatory conditions (960). Based on the hygiene theory in the pathogenesis of allergy, it has been suggested that the immune system is shaped for Th1/Th2 imbalance with the effects of environmental factors, particularly in the developmental phase of the children’s immunity. The effect of mucosal allergy on immunity has recently been shown, and the probiotics have been used in allergic diseases for their immune regulating effects and their effects on intestinal permeability (961). Studies on the relationship of intestinal flora with allergy have shown that children living in the developed countries where allergy prevalence is high have fewer Lactobacilli and Bifidobacteria and more *Staphylococcus aureus* and Clostridia in their intestinal flora compared to the children living in developing countries (962, 963). Penders et al. (964) showed a link between *E. coli *colonization in the intestinal mucosa and development of atopy, however bifidobacteria, *B. fragilis* and Lactobacilli colonization did not have any relationship with the development of recurrent wheezing, eczema or atopic dermatitis.

Some authors proposed use of probiotics to modify the intestinal mucosal and systemic immune responses in treatment of atopic children. Although the results of the studies are conflicting, various authors showed that probiotics interacted with enterocytes and stimulated Th1 response in dendritic cells, increased IFN gamma level, suppressed Th2 response, and decreased IL-4 and specific IgE levels (965). Some other studies reported that interaction of probiotics with intestinal flora could increase TGF beta and T regulatory cells, and decrease IL-4-10 (966).

In the light of aforementioned information, Pubmed database was searched with the keywords, and three meta-analyzes were found before 2015. There were four meta-analyzes, 24 reviews and 14 clinical studies between 2013 and 2018. In the search done without any date limitation, a total of 157 publications were found on allergic rhinitis and probiotics in the literature.

When we examined the meta-analyzes published before 2015, we excluded the study conducted by Batchelor et al. (967) in 2010 since they analyzed the systemic reviews published in 2008-2009 and the innovations in atopic eczema, there was no information about the use of probiotics in AR treatment. Zajac et al. (968) searched Medline, EMBASE, and Cochrane databases in 2015, and reviewed 23 studies on 1919 patients that investigated the effects of probiotic use on AR, using Rhinitis Quality of Life Questionnaire (RQLQ), Rhinitis Total Symptom Score (RTSS), and total/specific IgE values. The results of their meta-analysis showed that the use of probiotics caused a significant improvement in RQLQ scores compared to placebo, but did not cause any significant changes in RTSS, total IgE or specific IgE (968).

In 2015, Zuccotti et al. (961) analyzed 17 studies on 4755 children in their meta-analysis. They investigated the effect of probiotic use during pregnancy and early infancy on the prevention of allergic diseases, and reported that the risk for eczema decreased significantly in infants using probiotics, however there was no significant effect for preventing asthma, wheezing or rhinoconjunctivitis.

We analyzed the meta-analyzes published after 2015, and found two meta-analyzes, and one of them was omitted since it was not specific to treatment. In 2016, Güvenç et al. (969) included 22 randomized, double-blind, placebo-controlled studies in their meta-analysis and investigated the effects of probiotics on AR treatment in terms of total nasal and ocular symptom scores, and quality of life questionnaires, personal nasal symptom scores and immunological parameters. They reported that probiotics led to significant improvements in total quality of life and total nasal and ocular symptom scores in both seasonal and perennial AR when compared to placebo. They analyzed personal nasal symptom scores as secondary outcomes, and showed significant improvements in nasal congestion, rhinorrhea, and nasal itching scores in the probiotic group when compared to placebo. No significant differences were found between the groups for total IgE levels or eosinophil counts. In addition, although it was not an expected result, their results indicated a decrease in the Th1/Th2 ratio for the first time in the literature, with use of probiotics.

We analyzed the clinical studies conducted in the previous 5 years. There were 14 randomized controlled clinical studies. One of them was about symbiotics, one of them was related to symbiotics and inflammatory nonallergic rhinitis, two of them were related to atopic eczema, and one study did not give specific data on AR, therefore these studies were not taken into consideration.

Miraglia Del Giudice et al. (970) performed a randomized placebo-controlled double-blind study, and investigated the effects of Bifidobacterium mixture administration [B longum BB536 (3x109 CFU), B infantis M-63 (1x10^9^ CFU), B breve M-16V (1x10^9^ CFU)] on children with seasonal AR due to parietaria pollen, and intermittent asthma. They investigated relief of nasal symptoms and the impact on quality of life. The active treatment group was administered Bifidobacterium mixture, mixed in a small amount of water or milk, once a day for 8 weeks. Cetirizine syrup and salbutamol inhaler were given as rescue therapy. The patients were asked to note the doses and the days when they used rescue medications. The patients’ total nasal symptom scores (TNSS) and Mini Rhinoconjunctivitis Quality of Life Questionnaire (Mini-RQLQ) scores were recorded at the beginning and end of the treatment. It was found that TNSS decreased significantly in the treatment group, and increased significantly in the placebo group. In terms of quality of life, it was reported that probiotic use significantly improved symptoms compared to placebo. Both groups used similar amounts of rescue medications. There was no difference between the two groups in terms of treatment compliance and side effects.

In a randomized placebo-controlled double-blind study, Dennis-Wall et al. (971) investigated the effects of Lactobacillus gasseri KS-13, B. bifidum G9-1, Bifidobacterium bifidum G9-1, and Bifidobacterium longum MM-2 (2 capsules / day, total 1.5 x 10^9^ colony-forming units / capsule) administration for 8 weeks in the allergy season. They employed Mini-RQLQ, and measured total IgE and Treg cells in 173 patients with seasonal AR. They found significant improvements in the global scores as well as subgroups of activity, nasal, and other symptoms in the probiotic group when compared to placebo group, however there was no difference between the groups for ocular symptoms. There was less constipation in the probiotic group, however the difference between two groups reached statistical significance in 3^rd^, 4^th^, 6^th^, and 7^th^ weeks. The total IgE levels and Treg values ​​were measured at the baseline and in 6^th^ week, there was an increase in both levels in 6th week compared to baseline ​​in the probiotic group, however the difference between two groups was not significant.

A new area for use of probiotics is immunotherapy. In recent studies, combination with biological agents has been attempted in order to increase the success of immunotherapy, and successful results have been obtained. Tang et al. (972) were the first authors that combined Lactabacillus rhamnosus CGMCC 1.3724 probiotic, which was shown to stimulate Treg and Th1 cytokine response, with peanut oral immunotherapy in a double-blind, randomized, placebo-controlled study conducted on children with peanut allergy. They showed a decrease in peanut-specific IgE levels and an increase in peanut-specific IgG4 levels as well as decreased prick test reactions for peanut. However, the authors did not compare the effects of immunotherapy combined with prebiotics and allergen immunotherapy alone in peanut allergy. Similarly, Jerzynska et al. (973) investigated the effect of probiotic (Lactobacillus rhamnosus GG) and vitamin D as adjuvants on efficacy of sublingual immunotherapy. They investigated symptom medication scores (the score calculated by combining rhinitis, conjunctivitis, and respiratory symptom scores and by scores of salbutamol puff use, and seasonal cumulative pollen concentration), lung functions, respiratory nitric oxide levels and immunological parameters including CD4 + CD25 + Foxp3 + (forkhead box P3) cells, Toll-like receptor (TLR) 4, IL-1-6, tumor necrosis factor alpha, IL-10, and transforming growth factor b-1. They included 100 patients diagnosed with grass allergy in the study, and divided them into groups of 25 individuals as follows: SLIT + probiotic (Lactobacillus rhamnosus GG 3x10^9^ CFU), SLIT + Vitamin D (1000 IU), SLIT + placebo (0.3 mg Lactose) and allergic rhinoconjunctivitis patients that were not administered SLIT. The authors followed up patients for 5 months. SLIT + placebo group did not show any differences from the control group except for symptom-medication scores and FEV1%VC values. In SLIT + Vitamin D group, FEV1% VC as well as CD4+ CD25+ Foxp3 +, TLR values increased, and symptom-medication scores decreased significantly compared to the control group, independent of serum vitamin D levels. In SLIT + probiotic group, CD4 + CD25 + Foxp3 +, FEV1% VC, serum vitamin D levels increased, TLR positive cells, respiratory nitric oxide levels and symptom medication scores decreased more than the Vitamin D group. There was no significant difference between pre- and post-treatment measurements of other investigated immunological parameters. The authors claimed that adjuvant Vitamin D and probiotics might have directly induced Fox3P3 cells and enhanced the immunological effects of SLIT, and the results of their study provided a direct evidence for complementing SLIT with probiotics and vitamin D to application as might be recommended.

Simpson et al. (974) administered perinatal probiotics between 36^th^ week of gestation and 3 months postpartum to 415 pregnant women, followed their children for 6 years, and studied development of atopic dermatitis, asthma, and allergic rhinoconjunctivitis in their children in a prospective randomized placebo-controlled double-blind trial. The authors aimed to show the preventive effect of early use probiotics in childhood on atopic dermatitis development, which was shown in previous studies, and also to investigate the effect of perinatal probiotic use on the formation of allergic disorders in the general population, rather than the atopic population, which was not shown before. The pregnant women included in the study were administered daily 5 × 10^10^ colony-forming units (CFUs) Lactobacillus rhamnosus GG (LGG), Bifidobacterium animalis sub sp. lactis Bb-12 (Bb-12) and 5 × 10^9^ CFU L. Acidophilus La-5 (La-5) in 250 mL of low-fat fermented milk. Control patients were administered probiotic-free milk with a similar taste, and the children were not given any probiotic supplements. Clinical follow-up of the patients was made at the ages of 1, 2 and 6 years, presence of disorders were recorded, and skin prick test was performed and allergen-specific IgE levels were measured. At the end of the study, the authors concluded that the use of perinatal probiotics did not cause any change in the incidence of cumulative allergic rhinoconjutivitis, asthma and atopic sensitization prevalence at the end of the 6^th^ year.

Costa et al. (975) made a randomized placebo-controlled double-blind trial to study the effectiveness and safety of Lactobacillus paracasei (LP-33) (2.0x109 CFU) administered for 5 weeks as an adjuvant to loratadine in 18-60-year-old patients with AR related to grass pollens. They included 425 participants into their study, and used Rhinitis Quality of Life (RQLQ) global score as the primary outcome measure, and visual analog scale, nasal and ocular symptom scores (RTSS) (personal and total) and the first time for need of a rescue medication as secondary outcome measures. Although there were significant improvements in scores of both the placebo and probiotic groups in the follow-up, probiotic use improved significantly RQLQ total scores and ocular symptom scores compared to the placebo (p = 0.0255, p = 0.0029), however RQLQ nasal, RTSS nasal, RTSS ocular scores as well as VAS scores and time to need a rescue medication were similar between groups (p = 0.1288).

Lin et al. (976) examined the relationship of quality of life and the mediators with Lactobacillus paracasei (LP-HF.A00232) administered as an adjuvant to levocetirizine in 60 patients with perennial AR at 6-13 years of age. In their randomized placebo-controlled double-blind study, they administered 8 weeks of levocetirizine + probiotic or placebo, then they discontinued levocetirizine, and used placebo and probiotics for 4 more weeks. Pediatric RQLQ (RPQLQ), nasal, throat and ocular total symptom scores, IL-4-10, interferon gamma and TGF-b levels were recorded at baseline, and on 8^th^ and 12^th^ weeks in all participants. The use of levocetirizine as a rescue medication between 8^th^ and 12^th^ weeks significantly decreased in both groups. Although nasal, throat, ocular and total symptom scores decreased significantly in both groups during the follow-up period, no significant differences were found between two groups in terms of these values. There were significant improvements in general RQLQ scores in both groups during follow-up, but there was no statistically significant difference between the groups. Detailed analysis of symptom scores revealed a significant decrease in the symptom scores in the 5-8 and 9-12 weeks in the probiotic group, however this decrease was not evident in the placebo group. The analysis of RPQLQ subgroup scores between baseline and 12^th^ week showed significant improvements in the probiotic group compared to placebo only in the nasal itching, sneezing and swelling of the eyes domains. There was no difference between the groups in terms of cytokine values.

Ivory et al. (977) investigated the effect of an oral probiotic [Lactobacillus casei Shirota (LcS)] on nasal mucosal response after local allergen provocation test in seasonal AR patients in their randomized placebo-controlled double-blind study. Primary outcome measure was nasal total symptom score (TNSS), and secondary outcome measures were peak nasal inspiratory flow and local and systemic immunological response markers (eotaxin, IL-13, IL-1b, IL-4, IL-5, MIP-1 alpha and RANTES in the nasal lavage, soluble cytokine receptors sCD30, sIL-1RI, sIL-4R, sIL-1RII and sTNFR1, CD86, CD252 and intracellular cytokeratin in nasal swab, and IL-4, IL-5, IL-8, IL-10, IL-12p70, IL-13, IFN-gamma, TNF-alpha, eotaxin, MIP-1alpha and RANTES, soluble CD23, pollen-specific IgG, IgG4 and IgE in peripheral blood cell cultures). The patients were administered 6.5x10^9^ CFU LcS or placebo for 4 weeks, and the baseline and outcome values ​​were compared. There was no difference between the baseline and post-nasal allergen provocation test in TNSS, nasal peak flow, asthma or spirometry measurements values in the patients. Nasal lavage analysis did not reveal any difference in terms of eotaxin, IL-13, IL-4, IL-5, MIP-1α or RANTES. No significant change was detected in sIL-1RI, sTNFR1, sCD30 or sIL-4R in nasal cultures. There was a significant change between pre- and post-provocation IL-1b levels in the control group, but not in the probiotic group. In contrast to these findings, there was a significant difference in the probiotic group in terms of sIL-1RII, however there was no difference in the control group. Nasal cell cultures showed less CD86 and CD86 + CD252 + expression after allergen challenge in the control group compared to the treatment group. In peripheral blood cultures, no significant difference was found regarding IL-4, IL-5, IL-10, IL-12, IL-13, MIP-1 alpha eotaxin, RANTES or TNF-alpha. The authors reported that IFN gamma increased in the treatment group after provocation, and TGF beta, which was initially high in the control group, was not high anymore following provocation. The examination of peripheral blood cells in terms of systemic response showed no difference in terms of sCD23 after nasal provocation, whereas significant sCD23 release was detected in the control group after in vitro pollen application. There were no significant differences in terms of IgG, IgG4 and IgE. In summary, the authors showed changes in some immunological parameters, however this was not correlated with any change in the clinical parameters.

Dölle et al. (978) performed a randomized double-blind placebo-controlled study to investigate the toleration and clinical effect of *Escherichia coli *strain Nissle 1917 (EcN) (2.5–25 x10^9^ CFU) on 30 patients aged 18-65 years with grass allergy, starting administration two months before the allergy season, and going on for 6 months. The patients were examined clinically with symptom medication score as the primary outcome, and skin prick test, conjunctival provocation test, RQLQ and compliance with treatment as the secondary outcomes, however no significant differences were found between placebo and probiotic groups. It was found that compliance with treatment was good. Total and grass-specific IgE values were examined as immunological parameters, and no significant differences were found between the groups. It was shown that specific IgA increased significantly in the treatment group.

The use of probiotics in treatment of allergic rhinitis is shown on Table 8.3.2.1.

A literature search in the Turkish Medical Database did not yield any publications.

In conclusion, probiotic use has been considered a promising treatment method. However, the use of different probiotic formulations, the problems in designs of the studies, and the difference in evaluation criteria make it difficult to make a clear evidence-based interpretation on the use of probiotics. Therefore, routine use of probiotics is not recommended for AR treatment, since the available evidence is derived from insufficient and heterogeneous studies.

### 8.3.3. Phototherapy in allergic rhinitis

**Method:** A literature search was conducted in Pubmed, Scopus, Google academic and Thomson Reuters databases with the keywords “phototherapy, allergic rhinitis, rhinophototherapy, endonasal phototherapy”. Only meta-analyses were taken into consideration among the papers published before 2015. All international publications were included in this review between 2015 and 2018. There were 20 articles published in the international literature between 2013 and 2018. After reviewing the summaries of the articles, the articles that were not directly related to the keywords of phototherapy in AR were eliminated, and 10 research articles and one meta-analysis remained. Since four of these research articles were analyzed in the meta-analysis, the tables and graphics of this study were not included in the tables and graphics again. At the end, one meta-analysis and 6 international publications were included in this review. National literature was searched through Ulakbim and Google academic databases with the keywords “ fototerapi, alerjik rinit, rinofototerapi” without any date restrictions, and, three clinical research articles were identified.

Ancient Egyptians and Romans benefited of the therapeutic effects of sunlight thousands of years ago. With the advancements in modern medicine, phototherapy devices producing ultraviolet (UV) light have been used in order to benefit from the immunosuppressive and anti-inflammatory effects of different wavelengths of UV light, particularly for treatment of the dermatological conditions. Recently, rhinophototherapy devices have been developed for intranasal use. Rhinophototherapy devices have been launched in a number of countries, and they appear as an emerging alternative treatment method in the treatment of patients with AR.

Cho et al. (979) conducted a meta-analysis on the effectiveness of phototherapy in AR. The authors analyzed pre- and post- treatment nasal symptom scores, the effect of phototherapy on the quality of life, and the results of the studies that compared phototherapy with placebo or antihistamines, after grouping the studies. That meta-analysis included 13 clinical trials and 679 patients. The results of the studies were analyzed in three groups, as the effect of phototherapy on nasal symptom scores and quality of life, the effects on endoscopic findings, and comparison of phototherapy with a control group (placebo or antihistamine). It was reported that phototherapy provided statistically significant improvements in the total symptom scores, and sneezing, nasal congestion and rhinorrhea symptoms of the patients with AR. It was also found that phototherapy provided a statistically significant improvement on the quality of life. When the symptom scores and quality of life of patients with seasonal AR were compared with patients with perennial AR, a statistically significant difference was found. It was determined that phototherapy did not improve nose itching and sneezing symptoms of the patients with perennial AR. These results reveal that AR subtype (seasonal / perennial) is an important factor for benefiting from phototherapy, and phototherapy is more effective in patients with seasonal AR. Analysis of the effects of phototherapy on endoscopic findings revealed a statistically significant improvement in rhinorrhea and turbinate hypertrophy. When studies comparing the effectiveness of phototherapy with placebo or antihistamines were examined, phototherapy was found to be more effective than placebo in all symptom scores. Comparison with antihistamine showed that phototherapy was statistically superior to antihistamine in improving nasal congestion, rhinorrhea and itching, however the difference was not statistically strong. In addition, there was no statistically significant difference between phototherapy and antihistamine for total nasal symptom scores and sneezing. The authors stated that further studies are needed for comparison of phototherapy with antihistamines due to the weak statistical difference and high heterogeneity among studies in sneezing symptom scores.

Bella et al. (980) investigated the effectiveness of phototherapy in persistent AR patients in a randomized, double-blind placebo-controlled, study. In that study, besides nasal symptom scores, patients were evaluated with objective methods such as nasal mucociliary clearance measurement, objective smell tests, nasal inspiratory peak flow rate values, and ICAM-1 expression in nasal epithelial cells. The authors stated that phototherapy provided statistically significant improvements in nasal congestion, rhinorrhea, sneezing and itching complaints and nasal inspiratory peak flow values ​​compared to the placebo in patients with perennial AR. The smell thresholds were measured before and after treatment with objective tests, and no statistical difference was found. Phototherapy was also shown to have no adverse effects on nasal mucociliary clearance. Although improvements in olfactory function and mucociliary clearance were expected together with improvement of the symptoms, phototherapy had no positive effect on these parameters. Although phototherapy decreased nasal epithelial ICAM-1 expression significantly, no statistically significant difference was found between phototherapy and placebo groups. Phototherapy did not cause any significant adverse effects. Mild nasal dryness that resolved in a few days with topical moisturizers was seen only in three patients.

Alyasin et al. (981) conducted a prospective, randomized, single-blind study to investigate the effectiveness of phototherapy in 62 patients with moderate/severe persistent AR, who were unresponsive to topical and systemic medications. Patients included in the study were divided into two groups (n = 31), and visible light was used as placebo. After baseline evaluation, the patients were re-evaluated in the first, second and third months in terms of total nasal symptom scores, severity of the disease (global severity index) and quality of life. In the group treated with phototherapy, a statistically significant improvements were seen in total nasal symptom scores, disease severity and quality of life.

Tatar et al. (982) included 65 patients with persistent AR in their prospective, randomized study to investigate the effectiveness of rhinophototherapy. The patients were divided into two groups. The first group (n = 33) was treated with topical mometasone furoate 200 mcg / day and levocetirizine 5 mg / day for one month. The same medical treatment was administered to the patients in the second group, and they also received rhinophototherapy twice a week, for 3 weeks. All patients were evaluated with visual analog scale (VAS), rhinoconjuntivitis quality of life questionnaire (RQLQ) and nasal symptom scale before, and 1 and 3 months after treatment. Both groups showed significant improvements in 1st and 3rd months ​​in VAS, RQLQ and nasal symptom scores when compared to pre-treatment values, however the 1st month’s results were significantly better than the third month’s results. First and 3rd month VAS, RQLQ and nasal symptom scores were significantly better in rhinophototherapy group. The researchers claimed that the rhinophototherapy applied in addition to medical treatment provided a significant improvement in the symptoms and quality of life of patients with persistent AR.

The therapeutic effect of UV light is mainly linked to its immunosuppressive and immunomodulating effects. The leading mechanism that explains the immunosuppressive effect of ultraviolet light is induction of apoptosis. It has been suggested that this effect of UV radiation induces DNA damage (983). Possible precancerous effect of UV radiation-induced DNA damage has raised doubts about the safety of phototherapy in long-term use. However, different opinions were suggested in the current studies that investigated the mechanism of action of phototherapy in patients with AR. In an animal study, Yurttaş et al. (984). examined the nasal epithelial and connective tissue cells of the rabbits that underwent 3 weeks of phototherapy (Rhino-light 5% UVB, 25% UVA and 70% visible light), using the TUNNEL method. They showed that phototherapy did not induce apoptosis (983).

Kitamura et al. (985) investigated the mechanism of action of phototherapy in AR, and applied UVB radiation at different wavelengths. They showed that low-dose 310 nm narrow band-UVB radiation suppressed the H1R (H1 receptor) gene upregulation in HeLa cells but did not cause apoptosis.

Histamine is one of the main mediators in allergic reactions, and it shows its effect through H1 receptors. H1R is directly related to the occurrence of symptoms in allergic reactions, and it is considered as a rate-limiting receptor. Increased H1R mRNA expression has been shown in patients with AR. Therefore, it was supposed that treatment methods that could decrease H1R gene expression in the nasal mucosa might be beneficial in treatment of AR. In an experimental study, Kitamura et al. demonstrated that nasal low-dose 310 nm narrow band-UVB application suppressed the upregulation of the H1R gene, but it did not induce apoptosis. This effect was not observed at wavelengths longer or shorter than 310 nm. According to the results of that study, the authors suggested that 310 nm narrow-band UVB phototherapy might lead to improvement of AR symptoms, independent of apoptosis (985).

Yıldırım et al. (986) investigated the effect of phototherapy on nasal flora in a prospective single-blind study on 31 patients with perennial AR unresponsive to medical treatment. Nasal cultures and symptom scores of the patients were collected and analyzed before and after phototherapy. All symptoms improved significantly. Pre- and post-treatment nasal cultures were similar with regard to aerobic bacterial proliferation. The authors suggested that phototherapy did not have any significant effect on the aerobic bacterial flora in patients with AR.

National literature on the use of phototherapy in AR treatment includes Demirbaş et al.’s (987) study, in which 6 sessions of phototherapy were applied in two weeks to 24 patients unresponsive to pharmacotherapy. VAS, SNOT-20 and acoustic rhinometry were performed before and one month after treatment. There were statistically significant improvements in VAS and SNOT-20 scores after treatment, however no improvement was detected on acoustic rhinometry results. The authors concluded that phototherapy was an effective method of improving symptoms in AR patients resistant to antiallergic medications, and it had a positive effect on quality of life. However, the effect of phototherapy on nasal congestion could not be demonstrated with an objective method, i.e. acoustic rhinometry.

Yaz et al. (988) investigated the long-term effect of rhinophototherapy on the quality of life, and included 100 AR patients that were followed-up for at least one year into the study. This is the only study in the national and international literature that investigated the long-term effects of rhinophototherapy. RQLQ was used to evaluate the patients before, and 1, 3, and 12 months after treatment. Rhinolight III device (Rhinolight Ltd. Szeged 6721, Hungary) was used to administer phototherapy, 3 times a week, for 2 weeks. Rhinophototherapy provided a significant improvement in quality of life at the end of the first month, this improvement decreased in the third month, and almost disappeared in the 12^th^ month. The authors stated that nasal symptoms, restricted activities and sleep parameters improved the most in the short-term, however, ocular and non-allergic symptoms improved minimally. The authors concluded that phototherapy improved AR symptoms and examination findings, and affected quality of life scores favorably in the short-term, however these favorable effects decreased gradually in the long-term.

In a retrospective study, Akdağ et al. (989) investigated the short-term effects of rhinophototherapy on the symptoms of the AR patients resistant to medical treatment. They analyzed the symptom scores of 40 patients before and 2 months after rhinophototherapy. Statistically significant improvements were observed in all nasal symptom scores after treatment. The improvement was more pronounced in palate itching and sneezing, while rhinorrhea and congestion improved mildly. The authors stated that there was no gender or age difference for the effect of phototherapy on the symptoms. The researchers claimed that phototherapy might be a good alternative in AR patients resistant to medical therapy.

When the results of studies on the effectiveness and safety of phototherapy in AR treatment are assessed, one may say that it improves AR symptoms and quality of life in the short term. One-session phototherapy was well tolerated without any obvious adverse effects except mild nasal dryness, which resolved in a few days (979).

Almost all of the studies in the literature compared improvement in symptoms and quality of life before and after a single-session phototherapy. However, no information was given about the long-term results. Only one study in the literature followed up patients for one year after phototherapy, and Ayaz et al. (988) reported that improvement in symptom scores and quality of life decreased in the 3rd month, and went back to the pre-treatment level after one year.

Absence of multicenter, randomized, double-blind, controlled studies is an important shortfall for rhinophototherapy (979).

The measurement methods used in the vast majority of studies in the literature are not based on objective data such as nasal airflow and inflammatory mediators. Therefore, the mechanism of action of phototherapy in AR treatment is not fully known. In the prospective, randomized double-blind single study related to the subject, objective data including nasal mucociliary clearance measurement, objective smell tests, nasal inspiratory peak flow rate, and nasal epithelial ICAM-1 expression were investigated (980). However, no relationship was found between improvement in symptoms and improvement in objective tests in that study. Therefore, the data in the study failed to explain the mechanism of action of phototherapy. The studies that investigated the mechanism of action of phototherapy and its long-term safety were animal studies or in vitro experimental research. In some previous studies, it was claimed that phototherapy induced apoptosis by producing DNA damage. Therefore, DNA damage that may occur in long-term use of phototherapy is the most important drawback regarding the safety of this method. In one of the recent studies on the subject, Yurttaş et al. (984) claimed that phototherapy did not induce apoptosis in animals. Kitamura et al. (985) investigated UV radiation at different wavelengths, and claimed that 310 nm narrow-band UVB suppressed allergic inflammation, independent of apoptosis..

In conclusion, results of short-term rhinophototherapy are promising. However, there is not enough data on its long-term efficacy and safety. There is a need for multicenter, randomized controlled studies on long-term follow-up of the patients.

**Keywords:** Phototherapy, allergic rhinitis, rhinophototherapy, endonasal phototherapy.

### 8.3.4. Botulinum toxin in treatment of allergic rhinitis

**Method: **The international literature was searched with keywords “nasal secretion, Botulinum toxin, allergic rhinitis treatment” in Pubmed, Scopus, Google academic and Thomson Reuters databases. All international publications were included in the study between 2013 and 2018. In the literature review, 15 articles were identified in the international literature published between 2013 and 2018. After reviewing the abstracts of the articles, the articles that were not directly related to the keywords of “botox in allergic rhinitis” were eliminated, and four research articles and two meta-analyzes were taken into consideration. Two research articles published before 2013 were also taken into consideration since they were directly related to the subject. At the end, two meta-analyzes and six international publications have been included in this report. National literature was scanned in Ulakbim and Google academic databases with the keywords “ nazal sekresyon, Botulinum toksini, alerjik rinit tedavisi” without any restrictions on the date of publication, and one clinical research article was found. It was not included in this report since its publication date was very old.

Botulinum toxin has been used in AR treatment. Botulinum toxin is a toxin produced by *Clostridium botulinum*, an anaerobic bacterium (233).

Parasympathetic system has the dominant effect on nasal mucosal secretions. Acetyl choline is the main transmitter. Mucosal secretion and rhinorrhea appears with increased parasympathetic activity in patients at risk. When used for treatment of AR, botox inhibits release of acetyl choline from the preganglionic nerves in sphenopalatine ganglion as well as the cholinergic nerves in the nasal mucosa, and reduces neuropeptide release from the trigeminal and parasympathetic nerve endings. Thus, it reduces the parasympathetic activity in the nasal cavity, stopping nasal congestion and increased secretion resulting from parasympathetic activity (990).

Botulinum toxin can be applied by two methods in the treatment of AR:

### 8.3.4.1. Topical application

Topical application can be done in two different ways. The pads impregnated with 10-40 U botulinum toxin (the amount used in different studies varies in this range) are placed into the nasal cavity, and left there for about 30 minutes. Gel form (991) or nasal drops may be applied. Although gel and drop forms have been used in the literature, neither gel nor nasal drop forms of botulinum toxin have been marketed in Turkey.

About 40-200 U of botox may be used for infiltration. The amount of botox units is controversial. Nasal septum, inferior turbinate and middle turbinate were mostly preferred for infiltration.

### 8.3.4.2. Infiltration

Since the nasal septal blood flow is richer than the turbinate blood flow, the effect of the nasal septal injections lasts longer (992).The level of evidence of this publication, which describes the use of botulinum toxin in AR, is III, and its grade of recommendation is C.

The most commonly used method for dilution in topical and infiltration is 100 U botox diluted with 2.5 mL of saline, 1 mL of this solution contains 4 U botox. One study carried out on this subject suggested injection of 12.5 U of botox to bilateral posterior nasal walls, and reported an improvement in AR symptoms for 2-4 weeks (993).

The effect of botox infiltration usually becomes evident on the 3rd day after injection. The maximum effect occurs in the 2nd week. The beneficial effect ends after 3-6 months. The toxic dose is 2500-3000 U (994).

Dryness and epistaxis have been reported as adverse effects.

A review on this subject included 16 studies conducted between 1998 and 2015. In those studies, botox injection dose was 8-60 U, and botox-impregnated sponges contained 20-100 IU of botox. The injection site was generally inferior turbinate, middle turbinate and nasal septum. Rare adverse effects included epistaxis and nasal dryness. The maximum duration of action was reported as 20 weeks (992).

Animal experiments with botulinum toxin applications showed that nasal secretions and allergy findings decreased in 3 days after botox applications (233).

In conclusion, nasal botox application is an expensive, short-term effective, but easy and safe alternative treatment for AR. The level of evidence is IIb and grade of recommendation is B.

**Keywords:** Nasal secretion, botulinum toxin, allergic rhinitis treatment.

## 9. Tables for level of evidence

Category of evidence

Strength of recommendation

**National publications**

No articles were found matching ART-KLVZ-2.1 criteria.

**Appendix-2. Table of the references.**

The level of evidence and grade of recommendation columns were added to this table.

In addition, it was stated whether the related pharmaceutical company had financial support in the studies.

Some articles were deemed necessary to be included, but their full text could not be found. This was stated in an additional column, and these studies were included in the review due to the small number of publications on the subject.

## Figures and Tables

**Table 3-1:**
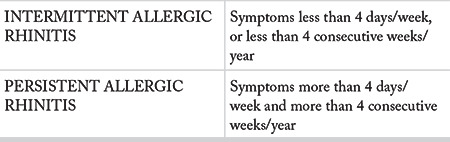
Classification of allergic rhinitis in relation with the duration of the symptoms

**Table 3-2:**
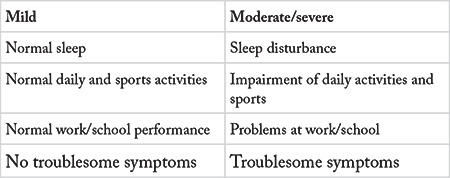
Classification of allergic rhinitis in relation with the severity of the symptoms

**Table 6-1:**
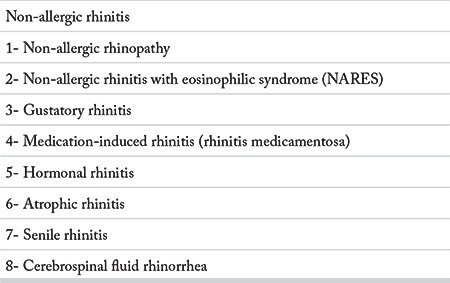
Classification of non-allergic rhinitis.

**Table 6.8.1:**
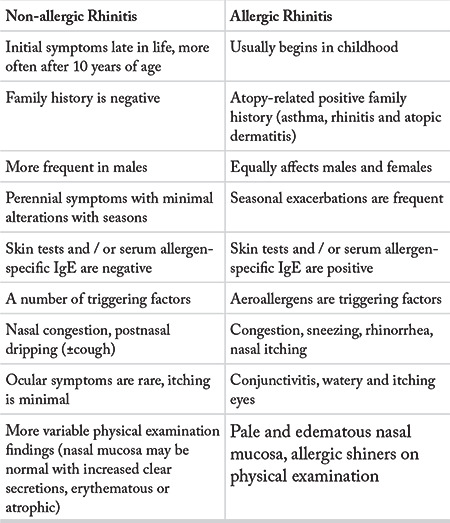
Differentiating features of allergic and non-allergic rhinitis

**Table 6.8.2:**
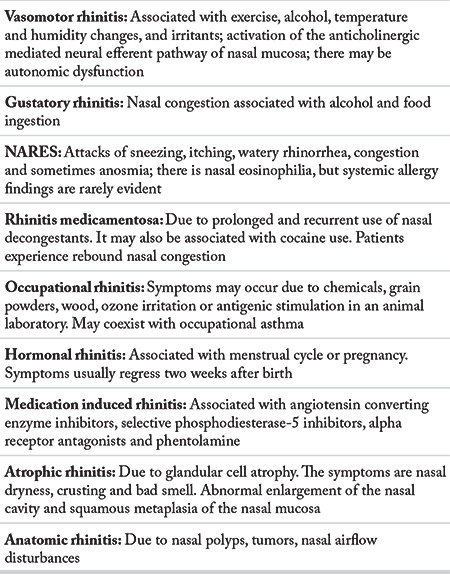
Classification of non-allergic rhinitis

**Table 7.2.2.1.1:**
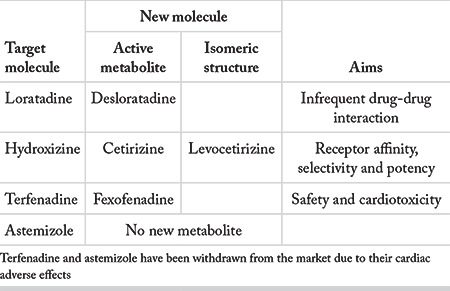
Development stages of H1 antihistamines

**Table 7.2.2.1.2:**
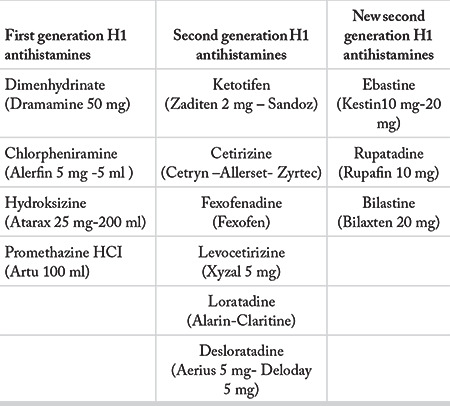
The most frequently used antihistamines and their trade names

**Table 7.2.2.1.3:**
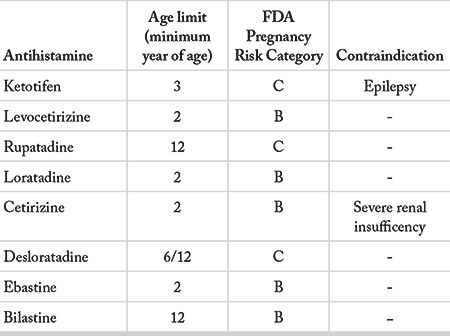
The second generation antihistamines registrated in Turkey

**Table 7.2.2.2.1:**
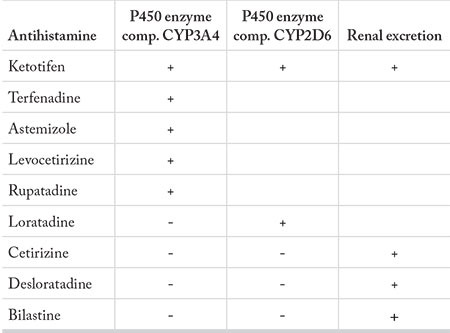
The relation of H1 antihistamines with cytochrome P450 enzyme complex metabolism

**Table 7.2.2.2.2:**
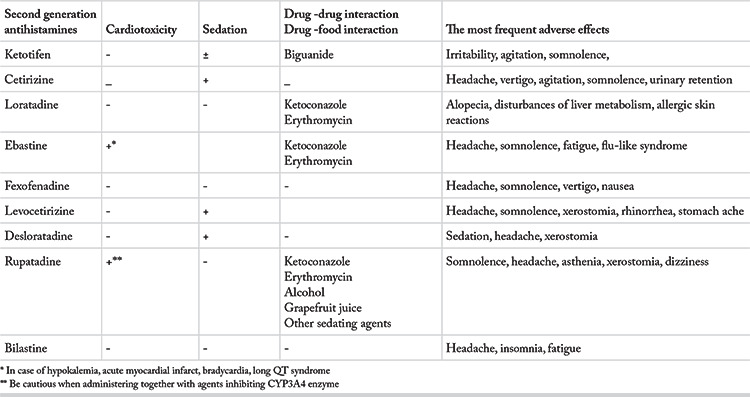
The adverse effects of second generation antihistamines

**Table 7.3.3.1.1:**
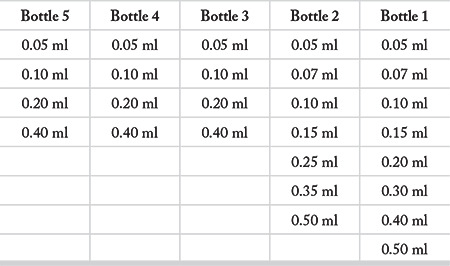
Conventional subcutaneous immunotherapy scheme.

**Table 7.3.3.1.2:**
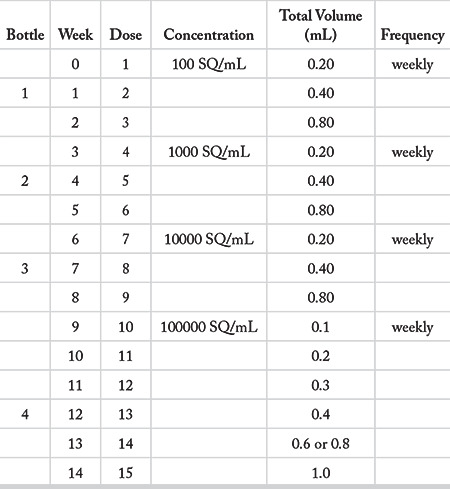
Conventional immunotherapy treatment scheme

**Table 7.3.3.1.3:**
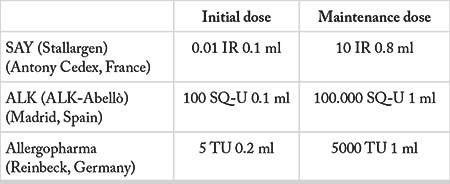
The types of allergen extracts by companies

**Table 7.3.3.2.1:**
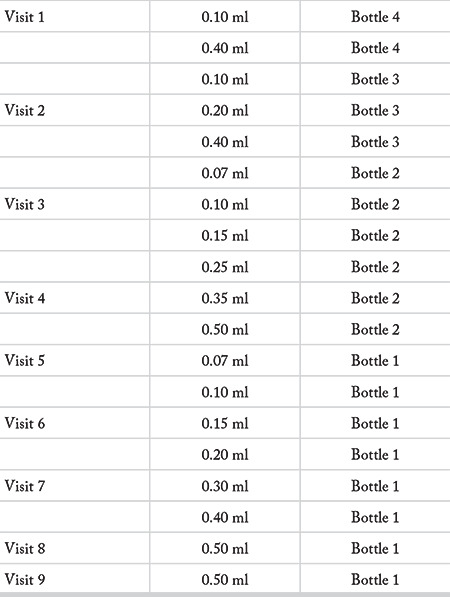
Subcutaneous immunotherapy cluster protocol scheme

**Table 7.3.3.2.2:**
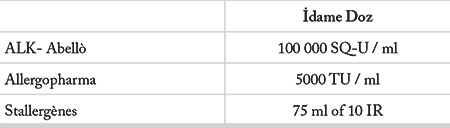
Maintenance dose

**Table 7.3.5.1.1:**
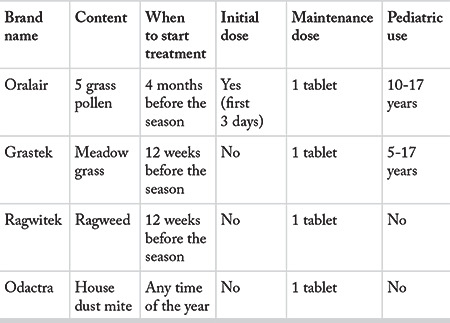
Treatment initiation and dosage schemes for SLIT tablets.

**Table 8.1.1.3.3.1:**
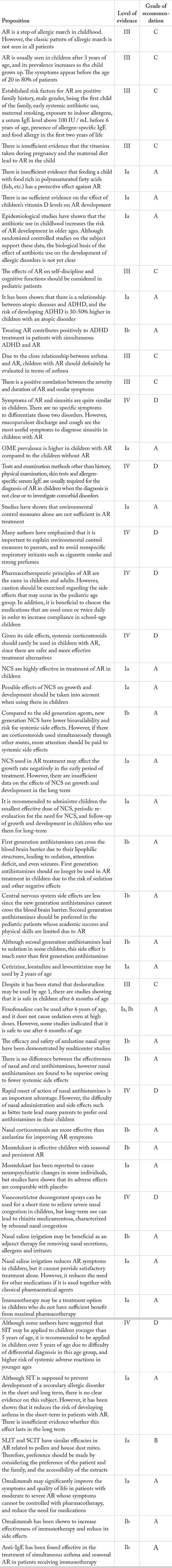
Level of evidence and grade of recommendation for general keystone propositions for immunotherapy for children.

**Table 8.1.3.1.1:**
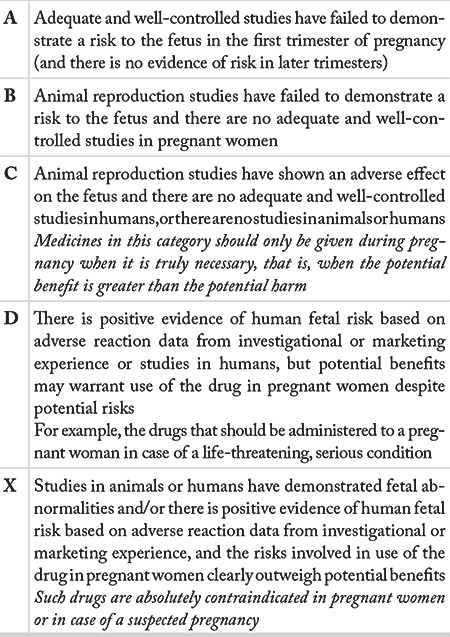
FDA pregnancy risk categories

**Table 8.1.3.1.2:**
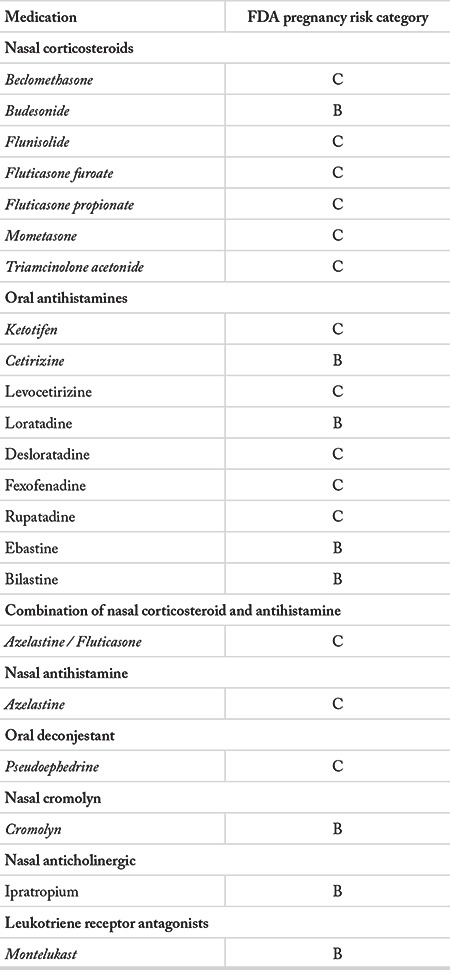
FDA pregnancy risk categories of drugs used in the treatment of allergic rhinitis

**Table 8.3.2.1:**
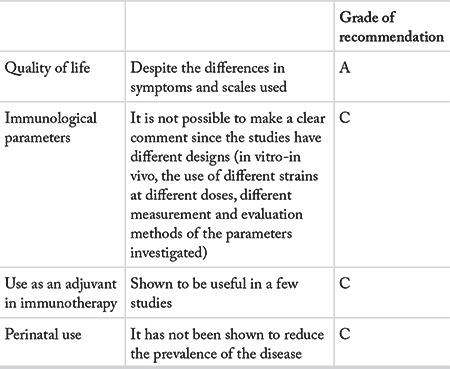
Use of Probiotics in Treatment of Allergic Rhinitis

**Category of evidence:**
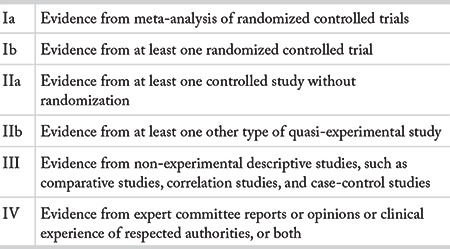


**Strength of recommendation:**
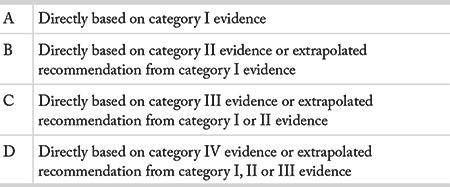


**Table 6.7:**
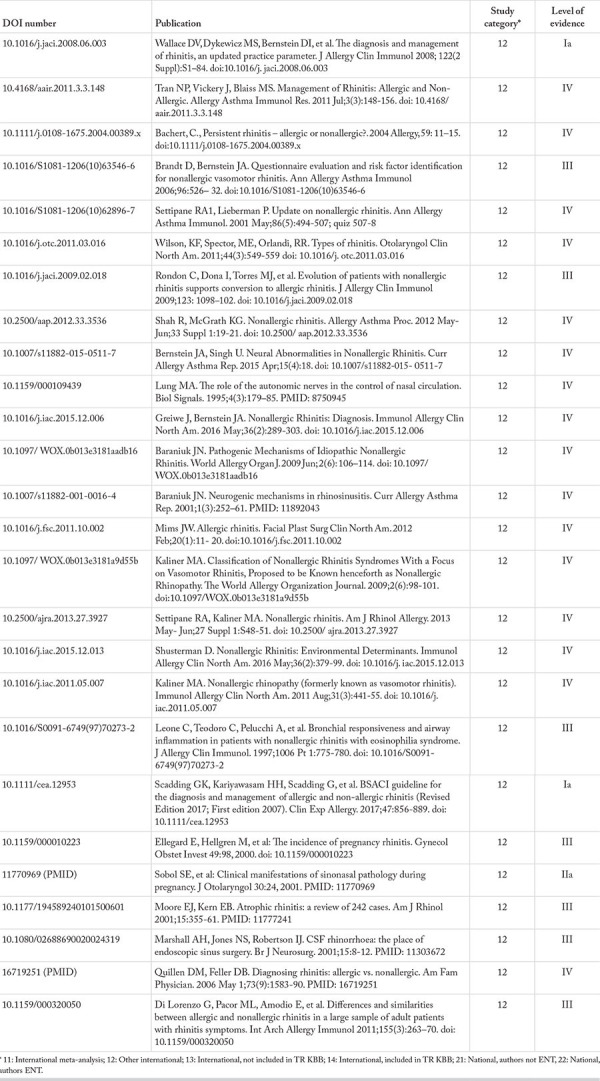
Differential diagnosis of allergic rhinitis.

**Table 6.8:**
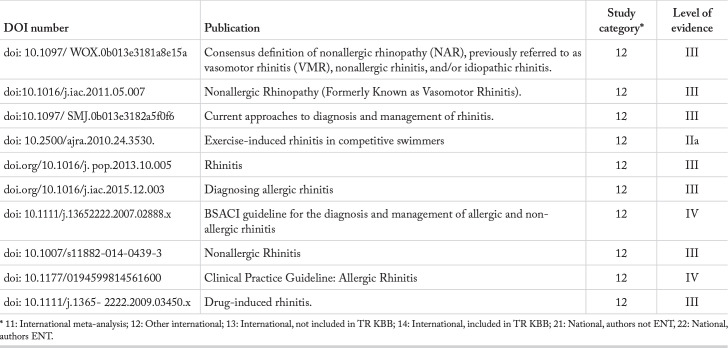
Differential diagnosis of allergic rhinitis and comorbid conditions.

**Table 6.8.1.1.2:**
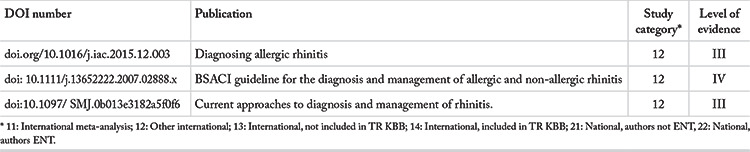
Physical examination.

**Table 6.8.1.1.3:**
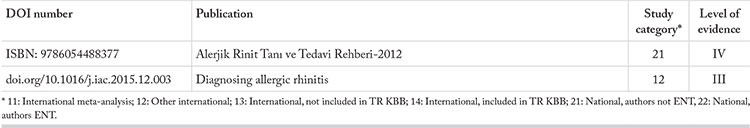
Diagnostic methods.

**Table 6.8.1.1.4:**
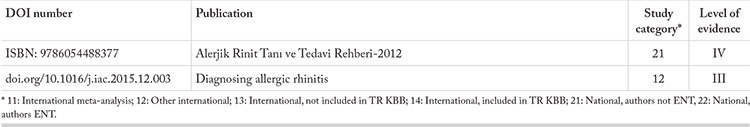
Further diagnostic workup.

**Table 6.8.1.1.5:**
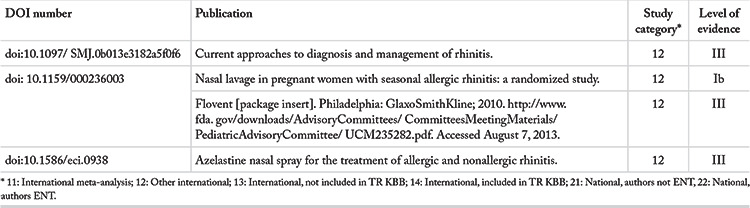
Treatment.

**Table 6.8.1.2:**
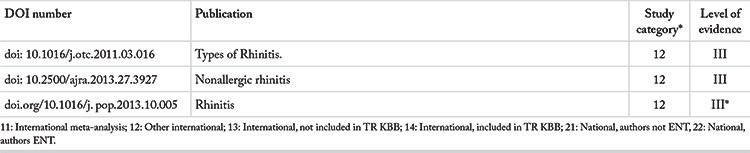
Disorders included in differential diagnosis of allergic rhinitis, except for nonallergic rhinitis.

**Table 6.8.1.3:**
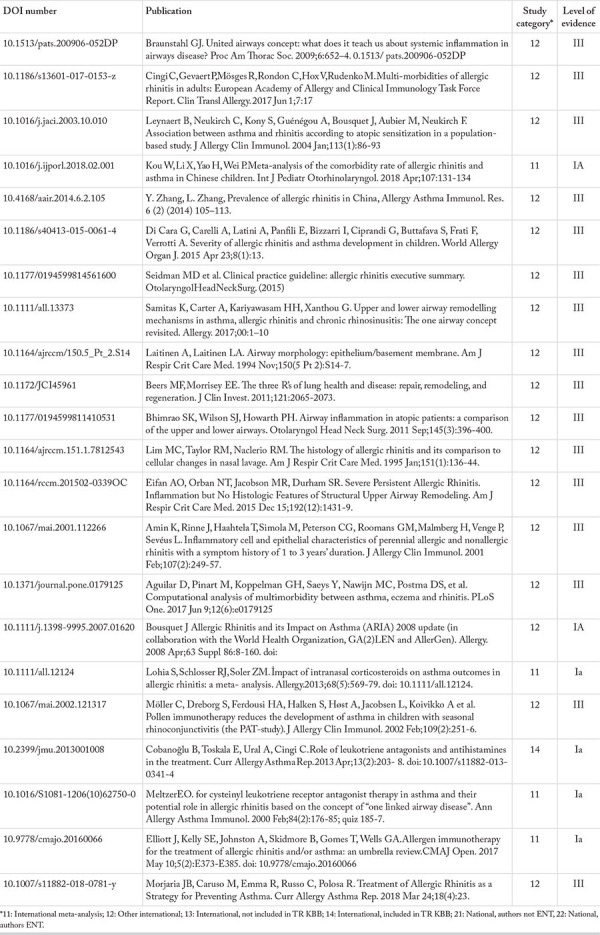
Bronchial asthma.

**Table 6.8.2.2:**
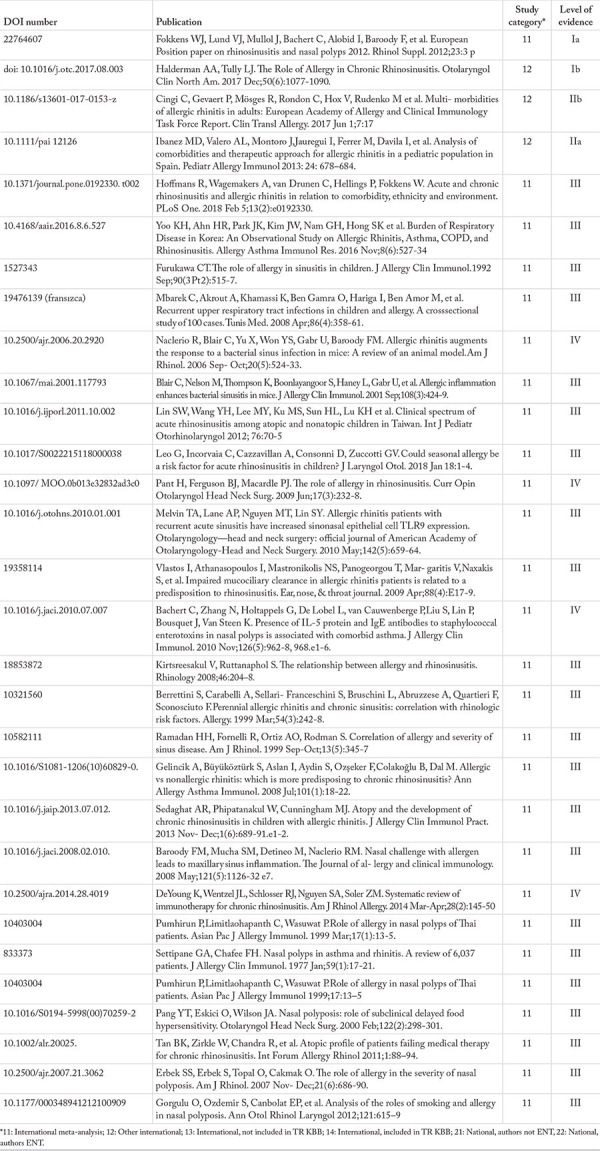
Rhinosinusitis.

**Table 6.8.2.3:**
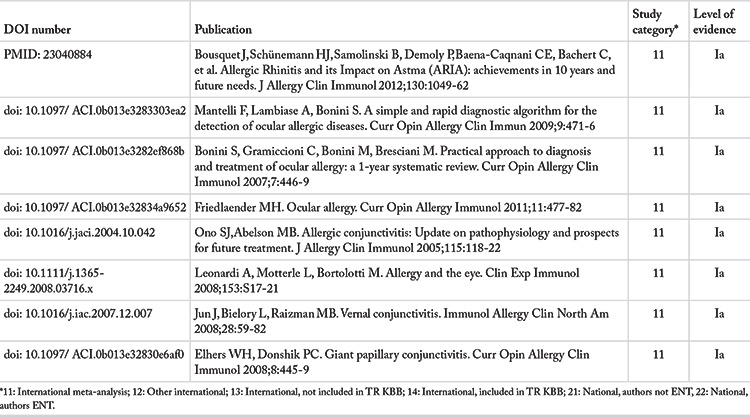
Conjunctivitis.

**Table 6.8.2.4:**
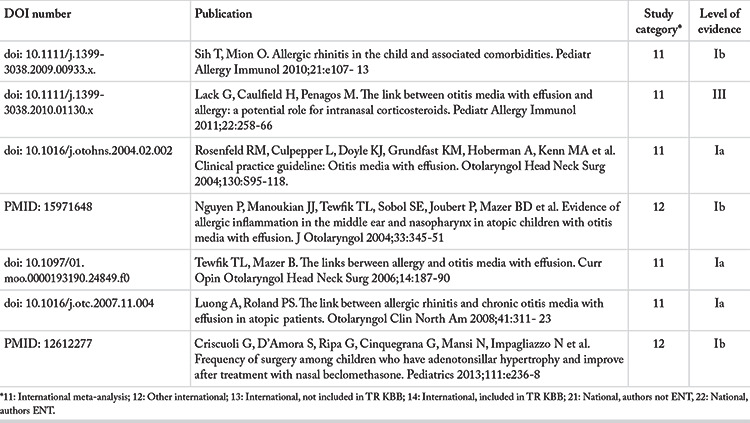
Otitis media.

**Table 6.8.2.5:**
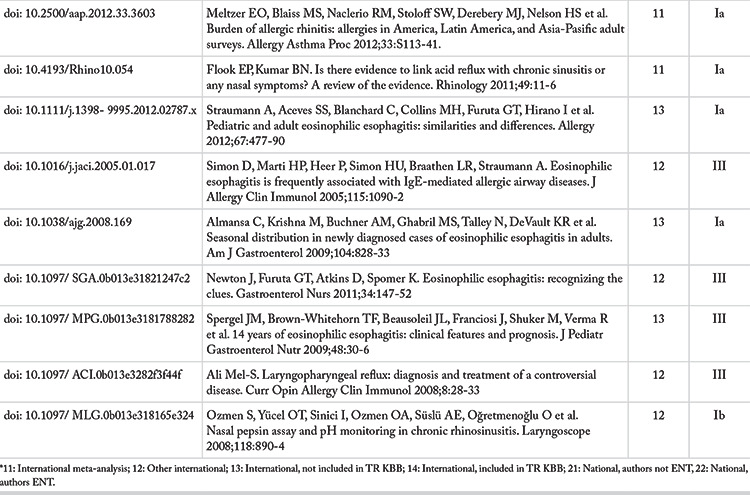
Gastroesophageal and laryngopharyngeal reflux.

**Table 6.8.2.6:**
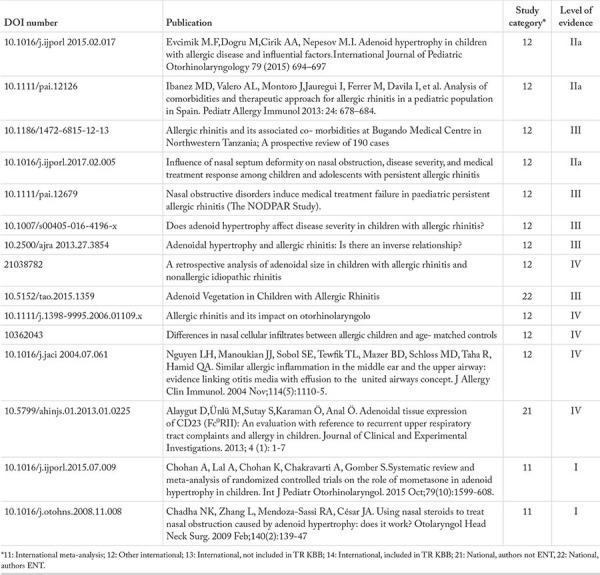
Adenoidal hypertrophy.

**Table 6.8.2.7:**
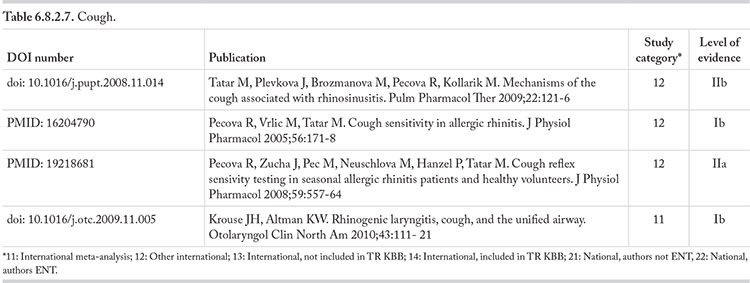
Cough.

**Table 6.8.2.7:**
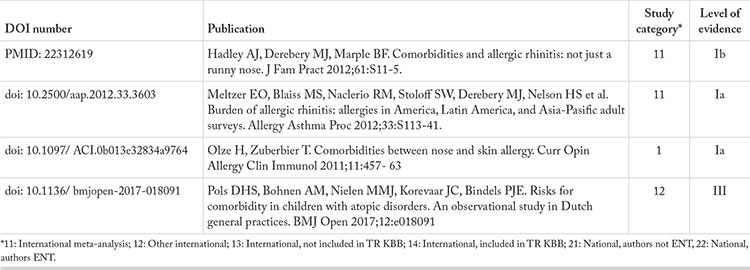
Skin rash.

**Table 6.8.2.9:**
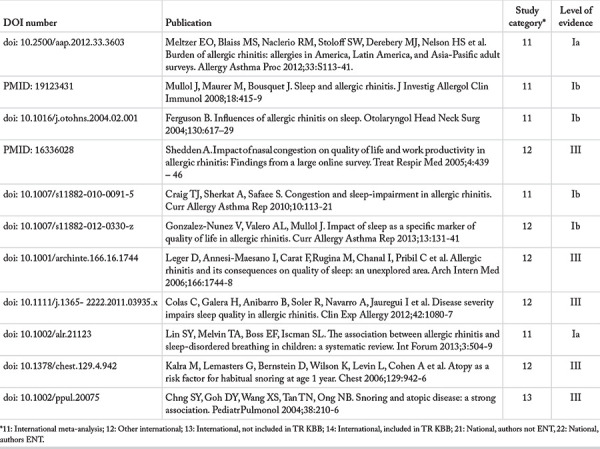
Sleep disorders.

**Table 6.8.2.10:**
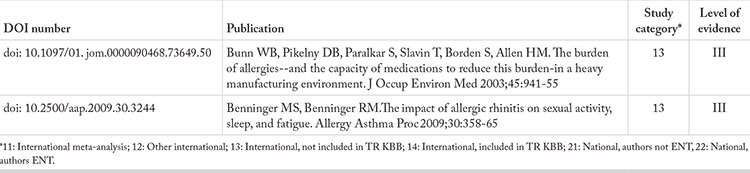
Cognitive disorders and learning disability.

**Table 6.8.2.11:**
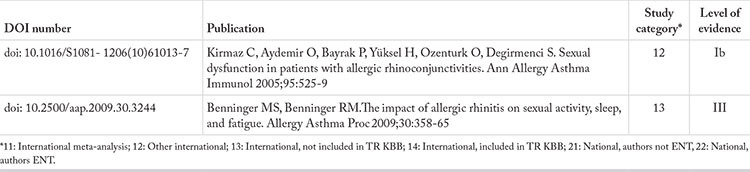
Sexual dysfunction.

**Table 7.1.1:**
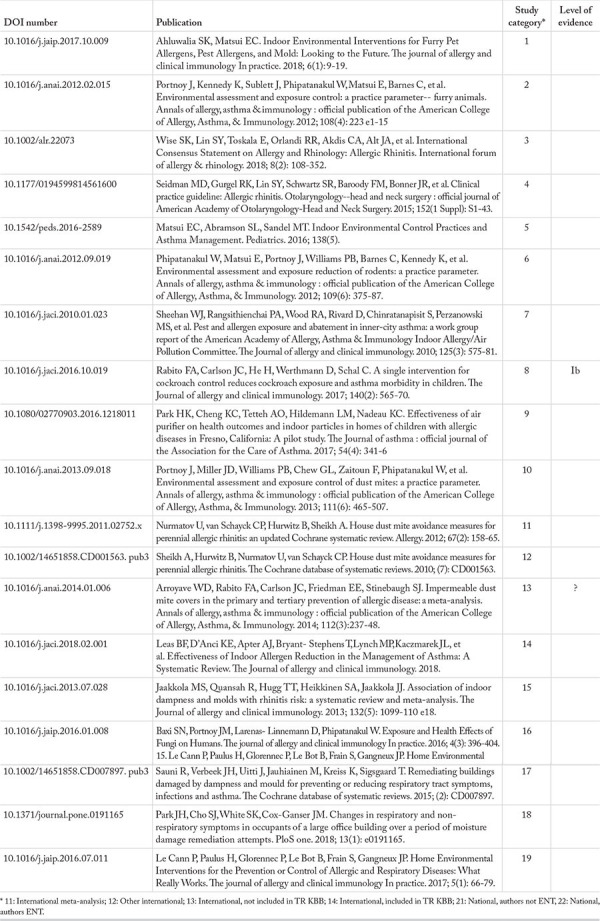
Control of indoors: methods for avoiding indoor allergens.

**Table 7.2.2:**
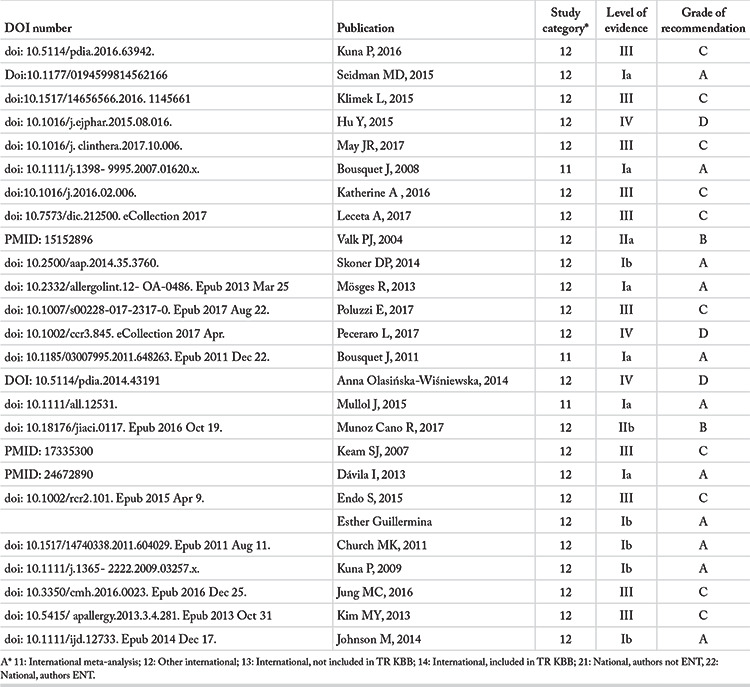
Oral antihistamines.

**Table 7.2.5.1:**
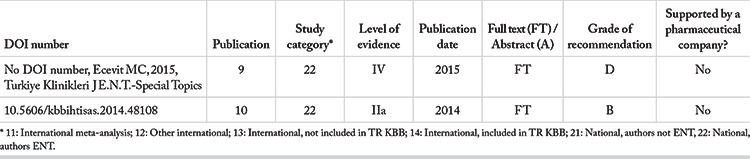
Combinations of oral antihistamine and leukotriene receptor antagonist (international).

**Table 7.2.5.1:**
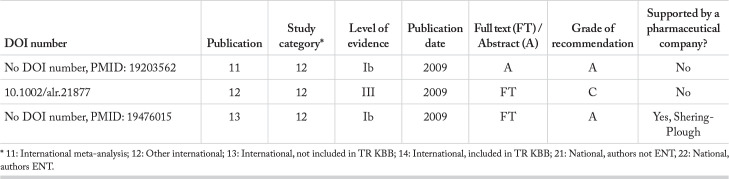
Combinations of oral antihistamine and leukotriene receptor antagonist (national).

**Table 7.2.5.2:**
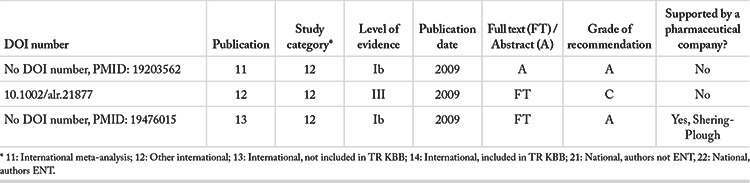
Combinations of oral antihistamine and decongestant.

**Table 7.2.5.3:**
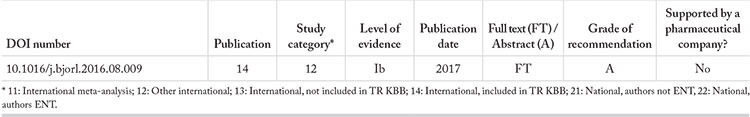
Combinations of antihistamine and corticosteroid.

**Table 7.2.5.4:**
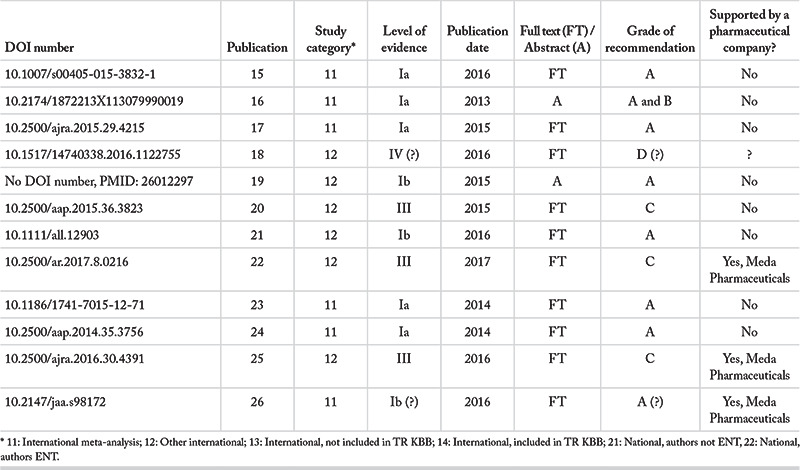
Combination of nasal corticosteroid and antihistamine.

**Table 7.2.5.5:**
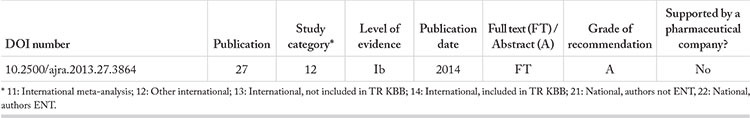
Combinations of nasal corticosteroid and decongestant.

**Table 7.2:**
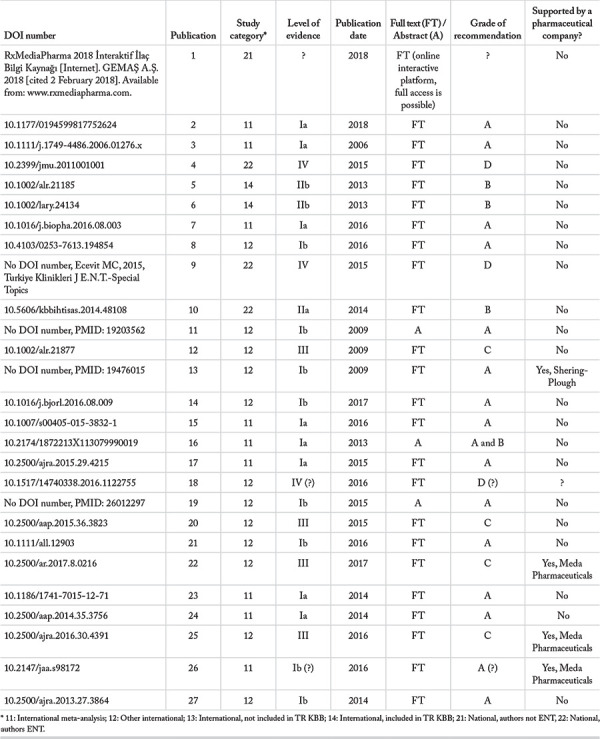
Classification of all publications included in the review.

**Table 7.2.4:**
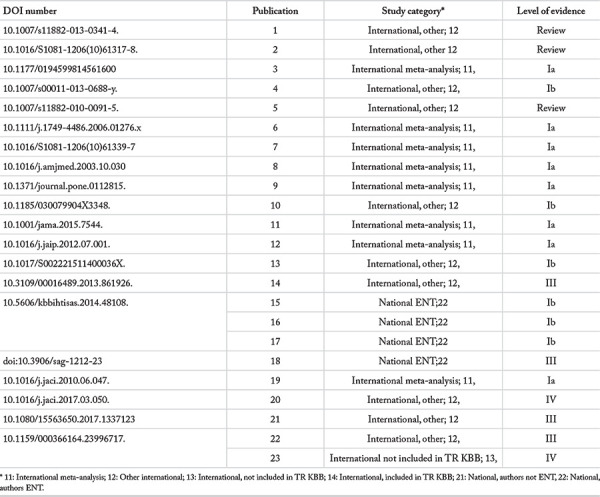
Antileukotriens.

**Table 7.2.6:**
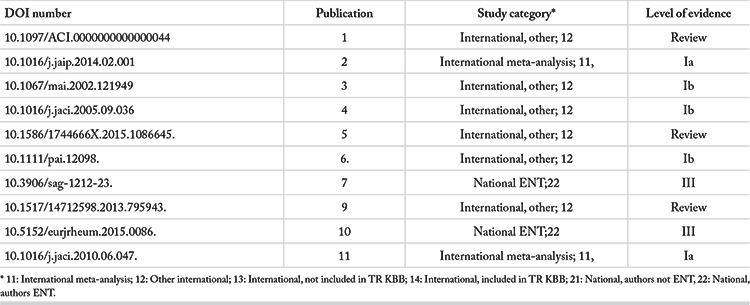
Anti-IgE.

**Table 7.2.7:**
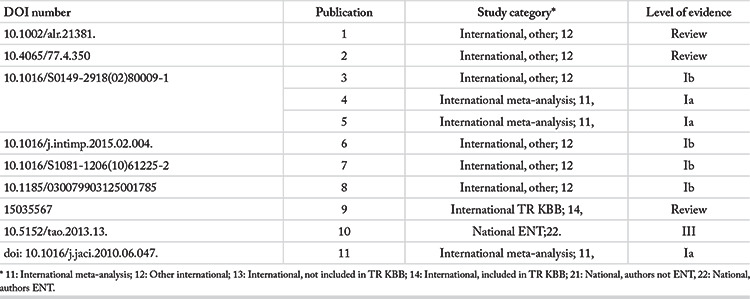
Cromolyns.

**Table 7.2.9:**
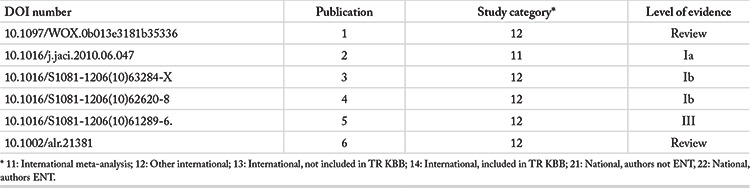
Anticholinergics.

**Table 7.2.3:**
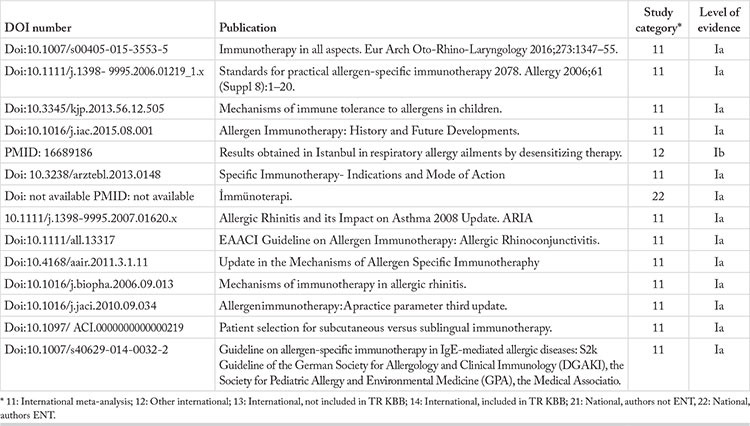
Immunotherapy.

**Table 7.3.2.1:**
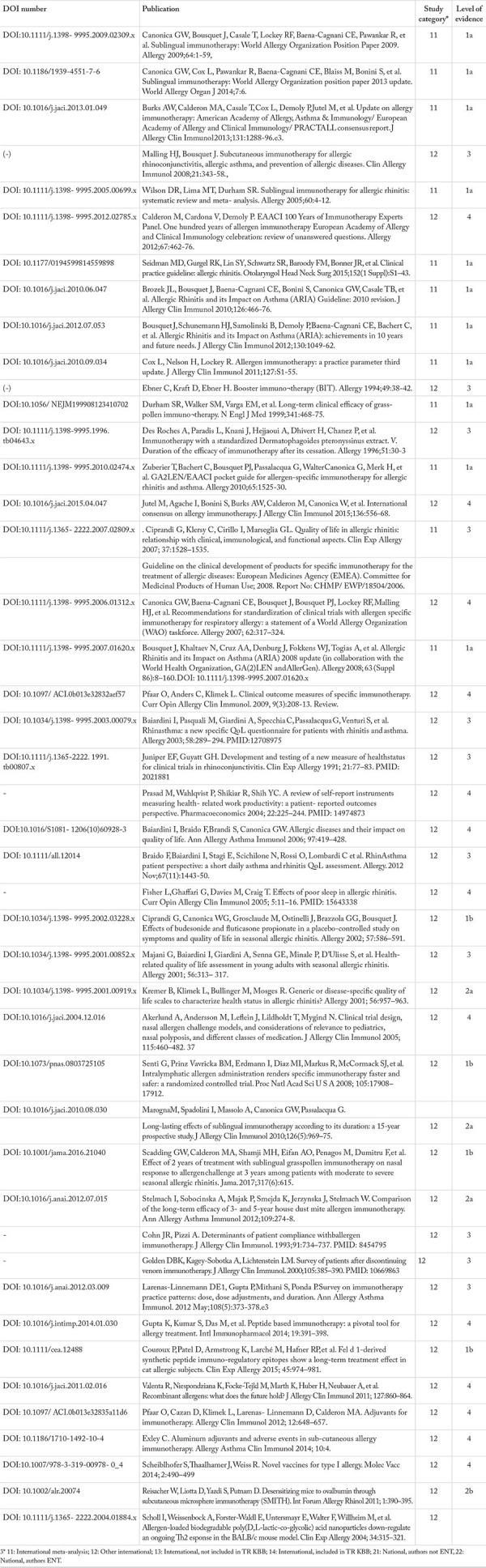
Treatment process.

**Table 7.3.3.1:**
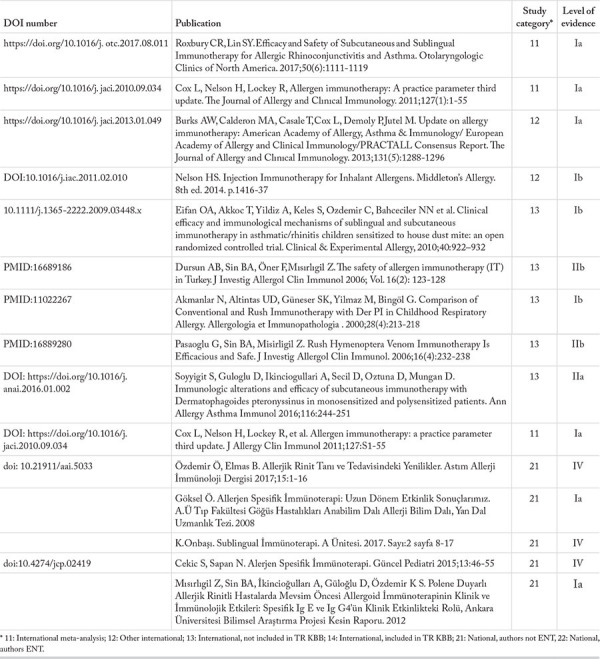
Initiation of treatment and the dose scheme.

**Table 7.3.3.2:**
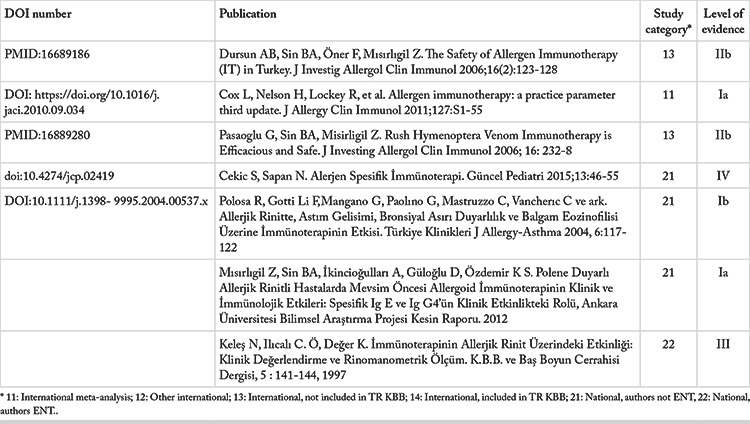
Maintenance and dose scheme.

**Table 7.3.3.3:**
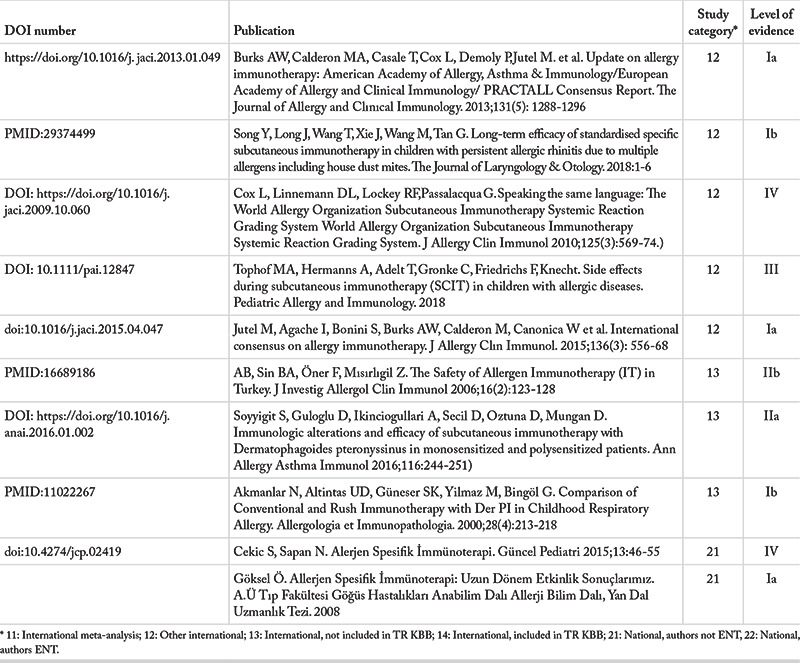
Adverse effects.

**Table 7.3.4:**
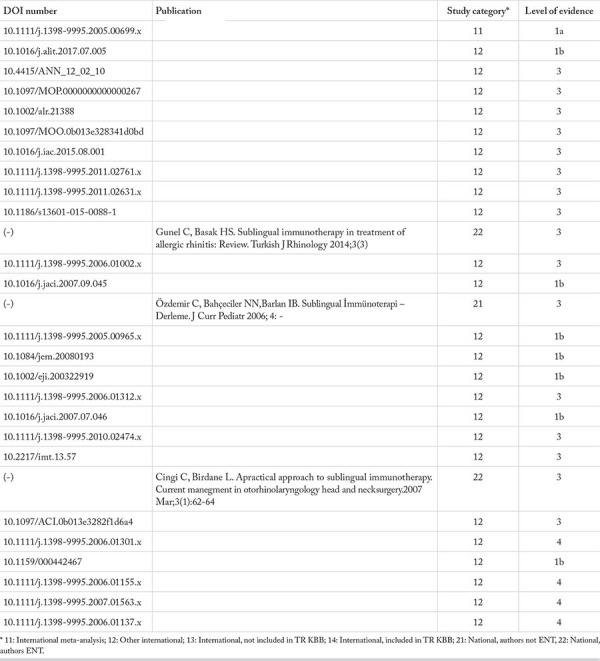
Sublingual immunotherapy.

**Table 7.3.5:**
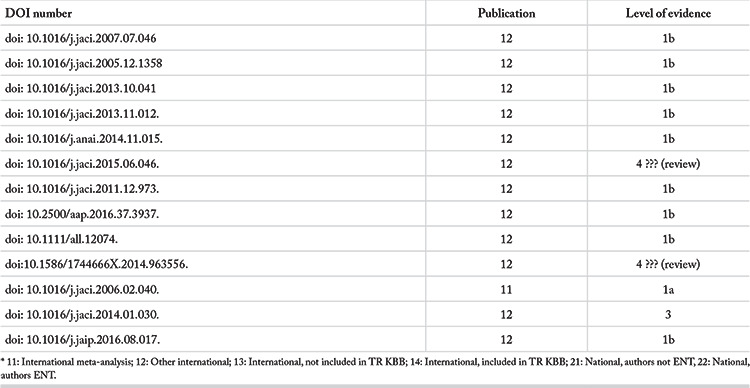
Oral immunotherapy.

**Table 7.3.7:**
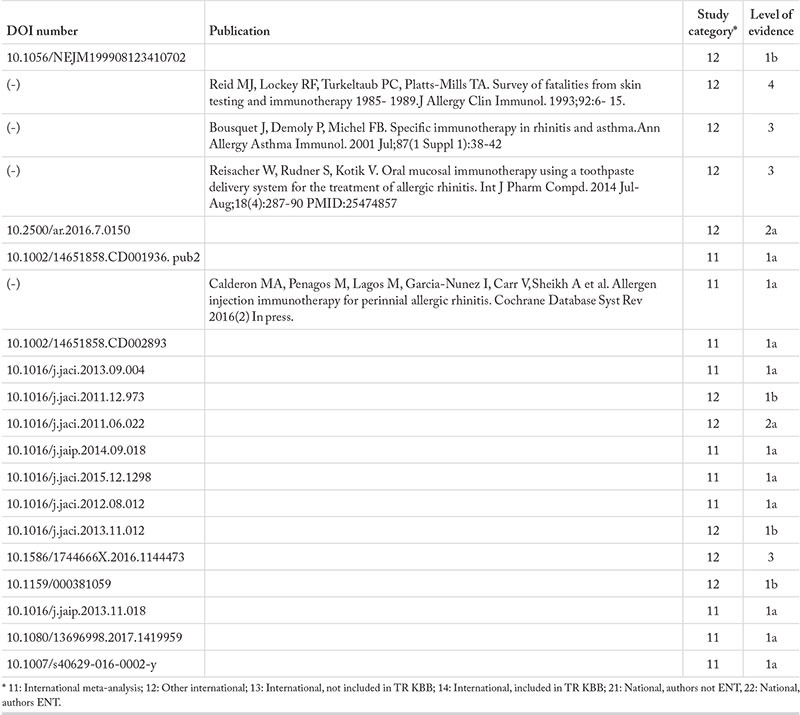
Comparison of immunotherapy methods.

**Table 8:**
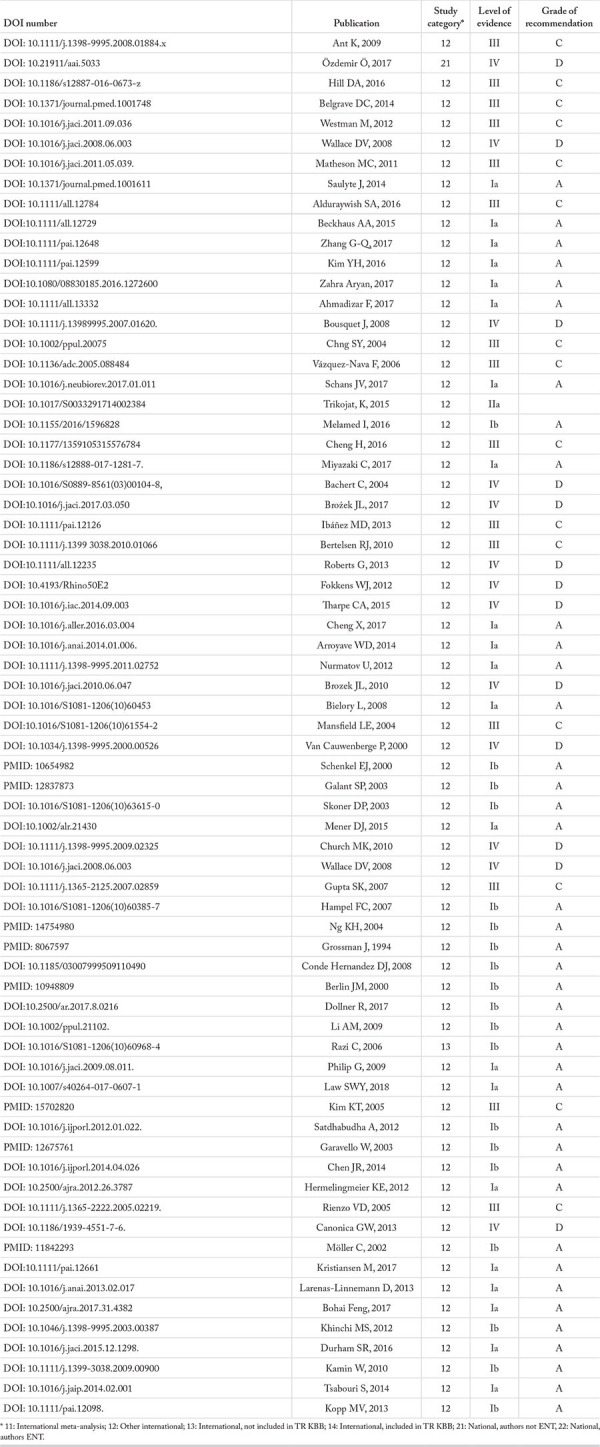
Special conditions in treatment of allergic rhinitis.

**Table 8.1.2:**
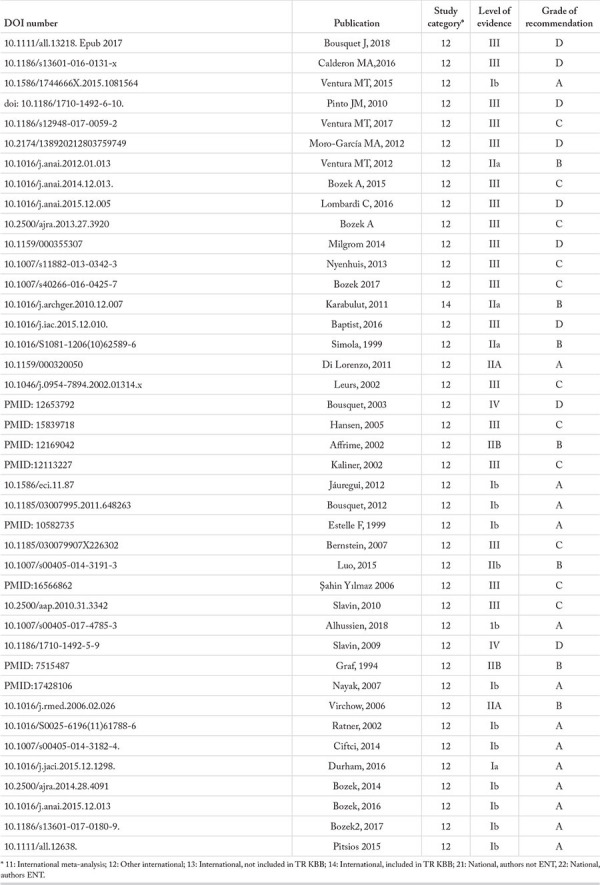
Treatment of allergic rhinitis in the elderly.

**Table 8.1.3:**
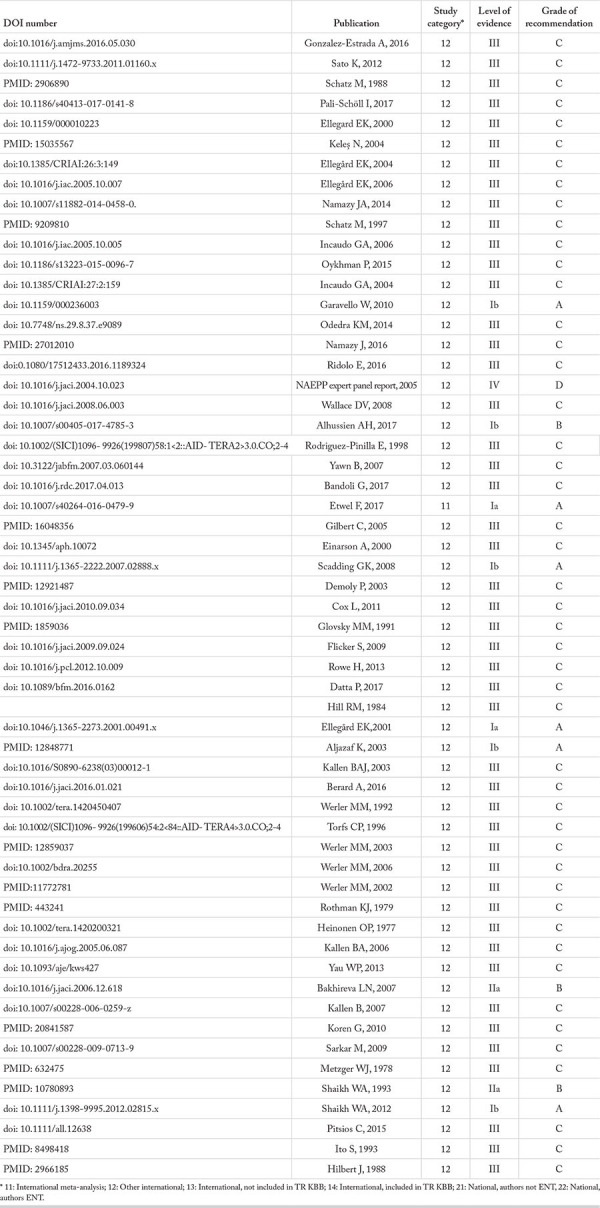
Treatment of allergic rhinitis during pregnancy and lactation.

**Table 8.1.4:**
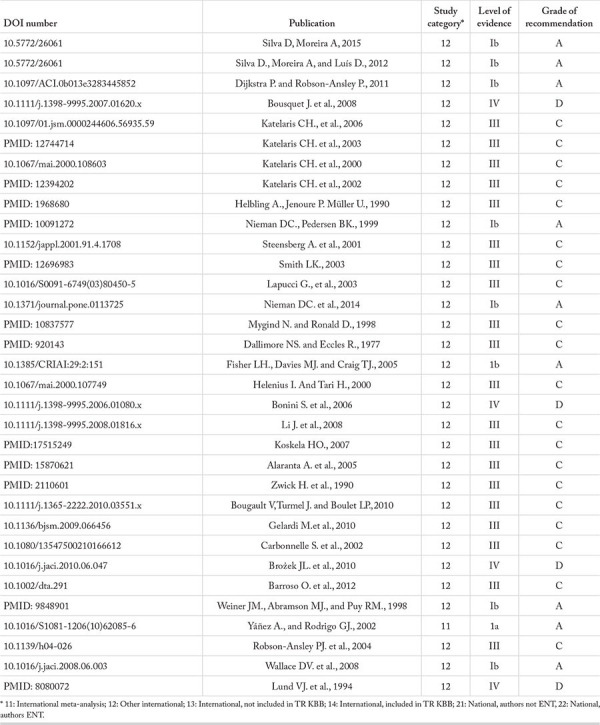
Allergic rhinitis and its treatment in athletes.

**Table 8.1.5:**
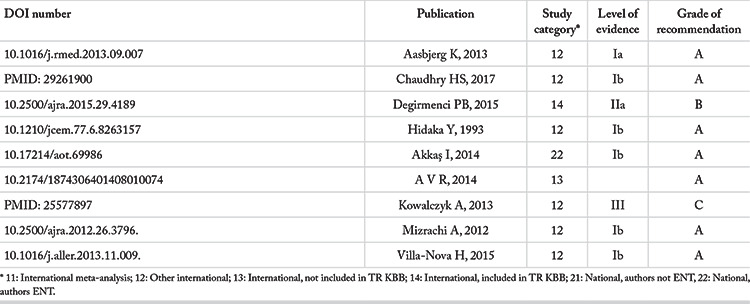
Treatment of allergic rhinitis in patients with comorbid endocrine disorders.

**Table 8.1.6:**
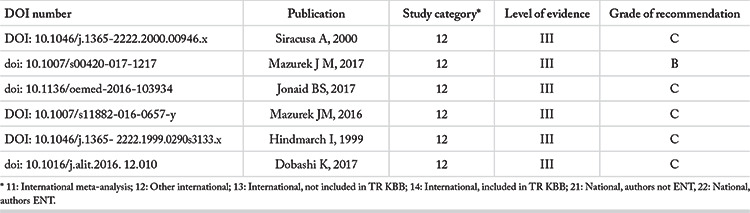
Special occupations (heavy and dangerous jobs).

**Table 8.1.7:**
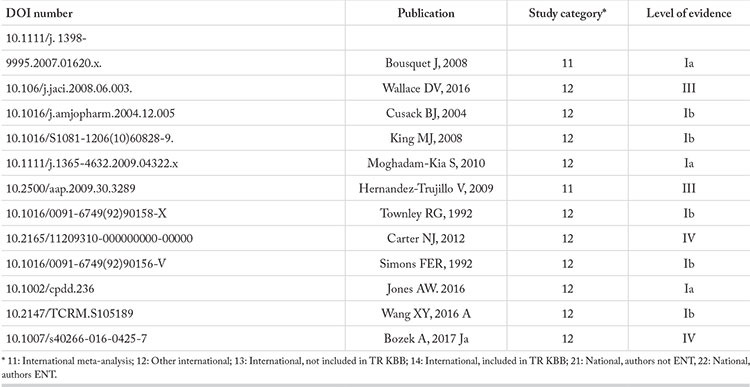
Treatment of allergic rhinitis in patients with other chronic conditions.

**Table 8.2.1:**
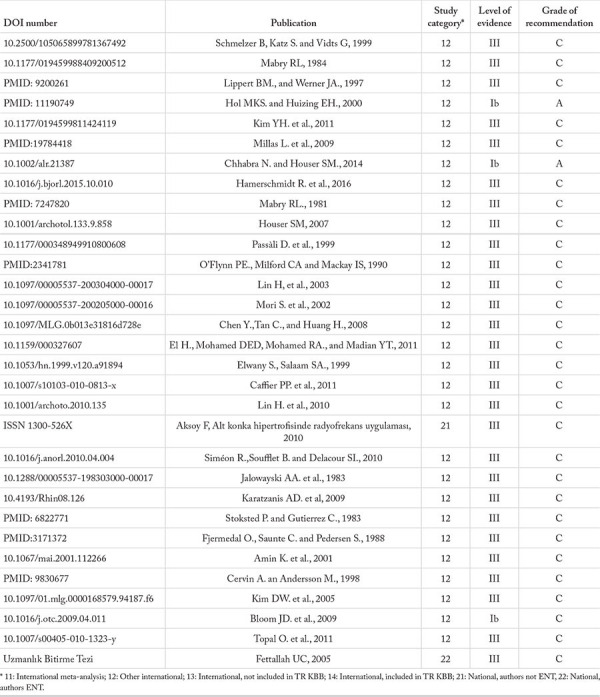
Inferior turbinate surgery and septoplasty in patients with allergic rhinitis.

**Table 8.2.2:**
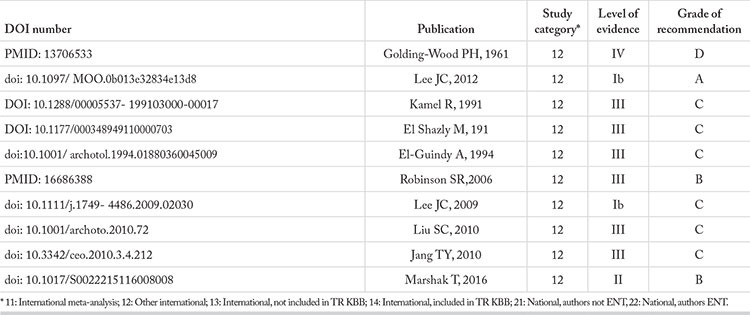
Vidian neurectomy in allergic rhinitis.

**Table 8.3:**
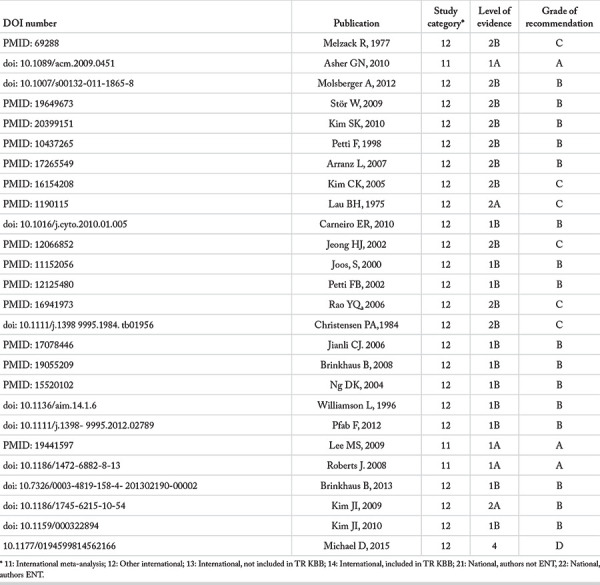
Other treatment methods in allergic rhinitis.

**Table 8.3.2:**
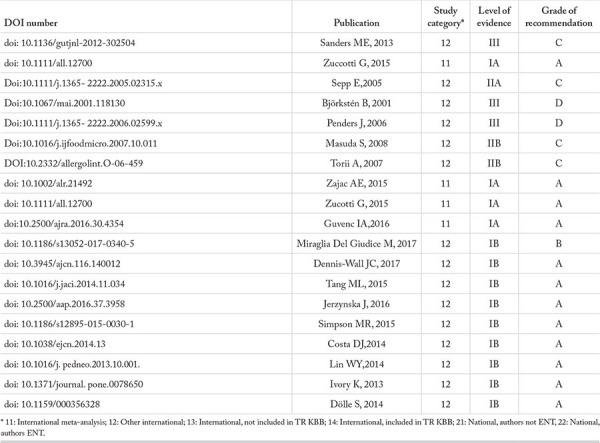
Probiotic treatment in allergic rhinitis.

**Table 8.3.3.3:**
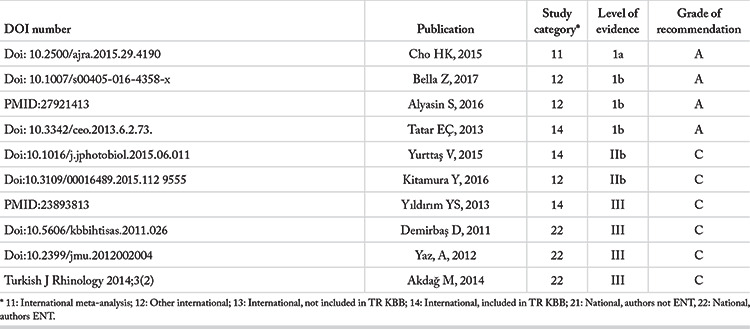
Phototherapy in allergic rhinitis.

**Recommendation:**
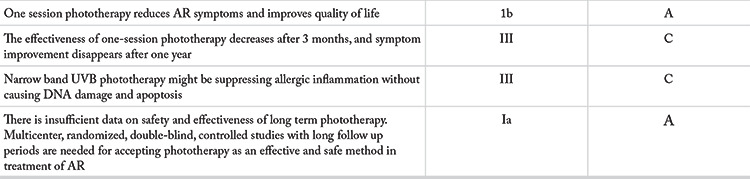


**Table 8.3.4:**
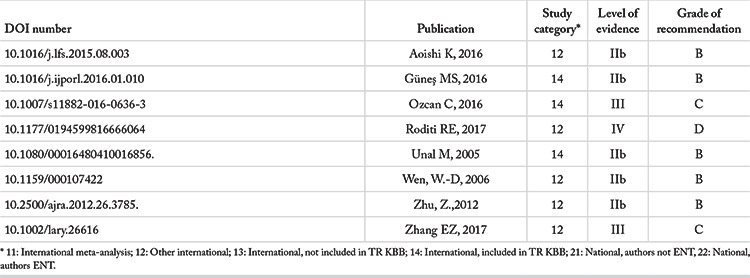
Botulinum toxin in treatment of allergic rhinitis.

**Table 7.3.1.1:**
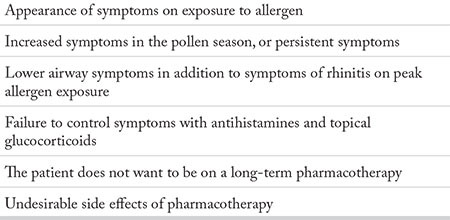
Indications of SIT (38).

**Table 7.3.1.2:**
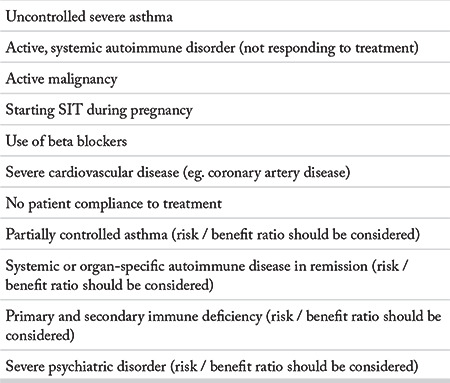
Contraindications of SIT (628).
